# On the Design of the Metal–Support Interface in Methanol Electrocatalytic Oxidation

**DOI:** 10.1021/acs.cgd.3c01466

**Published:** 2024-10-11

**Authors:** Bartłomiej M. Szyja, Joanna Zasada

**Affiliations:** Faculty of Chemistry, Wrocław University of Science and Technology, Gdańska 7/9, 50-344 Wrocław, Poland

## Abstract

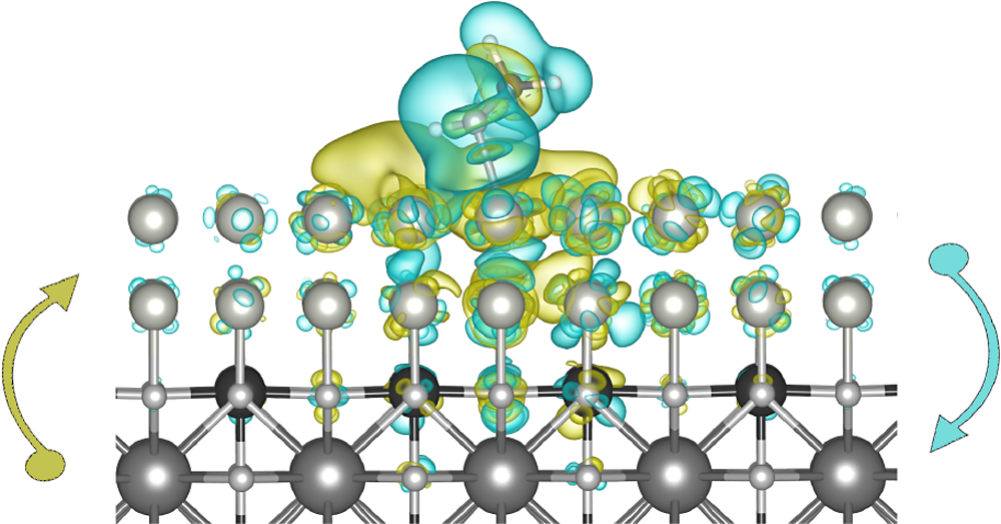

In this work, we present a theoretical investigation of the SrTiO_3_ perovskite-supported Pd catalyst in the methanol electro-oxidation reaction. In order to determine the metal–support interactions, we designed a system consisting of a Pd (100) double layer supported on one of the two possible terminations of the (100) perovskite surface. These terminations are characterized by different reducibilities of the layers directly interacting with the Pd bilayer and result in the difference in the stability of the surface-bound intermediates. Despite the fact that the Pd surface is identical in terms of geometry, we observed significant differences in the overpotential required for the reaction; in the case of TiO_2_ termination, the overpotential has been determined to be 0.68 V, while in the case of SrO termination, it amounts to as much as 1.35 V. We further investigate the charge transfers within the components of the system and the geometries of the intermediates to unravel the role of the electron structure on the overall efficiency of the process.

## Abstract 2

This work investigates a SrTiO_3_ perovskite-supported Pd catalyst for methanol electro-oxidation. A Pd (100) double layer is supported on two surface terminations (TiO_2_ and SrO), leading to different reaction overpotentials. Despite identical Pd geometry, TiO_2_ termination shows a lower overpotential compared to SrO. The study analyzes charge transfers and intermediate structures to understand the impact of the electronic structure on reaction efficiency.

## Introduction

In the era of increasing global energy consumption and decreasing deposits of fossil fuels, the matter of sustainability of energy generation has become one of the most important issues.^1^ While switching to zero-emission energy is considered by many to be the fundamental solution, it is not possible to be achieved overnight.^2−5^ Some CO_2_ emitters, such as automotive transport or small-scale household energy generation, are less dependent on fossil fuels, and in those cases, carbon neutrality can be easily achieved. On the other hand, as of 2023, the aviation sector still awaits new environmentally friendly propulsion technologies to be developed.^6,7^ The diversification of the energy sources can be considered either as an intermediate step toward or a complete alternative to zero emission,^8^ as it will allow for the reduction of CO_2_ emissions to the level that can be handled by Nature and effectively close the carbon cycle.

Out of the proposed solutions, the fuel cell concept deserves particular attention.^9−11^ It concerns the spatial separation of the oxidation–reduction half-reactions and the constant delivery of the reactants (fuel) to the half-cells. Many types of fuel cells have been proposed either to generate electricity or combine heat and power systems.^12,13^

The direct methanol fuel cell (DMFC) is a promising technology that allows to mitigate the issues related to hydrogen fuel storage, inherent in hydrogen-based fuel cells.^14^ Instead of molecular H_2_, it is based on the direct conversion of methanol, which does not require compression and high-pressure tanks and can be carried out at ambient temperature.^15^

Methanol oxidation reaction (MOR), which takes place on the anode of the DMFC, requires an efficient catalyst that—among others—is resistant to poisoning with CO, which is one of the intermediates of the process.^16,17^ The electronic structure of many noble metals makes them highly efficient catalysts in MORs.^18−20^ However, scarcity and the inherent high price of these metals are the main factors that increase the demand to search for an alternative. Most of the works have focused on Pt-based systems, and so far, Pt-containing intermetallic compounds have been reported as the most active.

As is the case for virtually all other catalytic systems, the role of the support cannot be neglected. A support material is commonly used for the actual catalyst in order to decrease the precious metal load and increase the specific surface area. It can also serve the purpose of a cocatalyst,^21^ which is the subject of our particular interest within the scope of this work, act as a catalyst promoter, and influence the properties of the metal phase by means of the metal–support interactions (MSI).

The role of different supporting oxides has been investigated with respect to the efficiency of the MOR.^21^ Scoffield et al.^22^ investigated the SrTiO_3_ and SrRuO_3_ perovskite systems as the supports for the Pt nanoparticles, as well as their respective oxides—TiO_2_ and RuO_2_. SrRuO_3_ has been found to better promote MOR than SrTiO_3_, which confirmed the relevance of the B site metal. In addition, the authors concluded that the presence of the Sr cation also plays a role, which they ascribed to the binding of hydroxyl species.

The reports on the activity of Pd-based systems in MOR are limited. Ji et al. synthesized Cu_2_O nanorods decorated with Pd and examined their catalytic activity in the MOR.^23^ They concluded a strong interaction between palladium and the support, which allowed the resulting catalyst to be twice as active in the alkaline medium as the commercial Pd black and Pd/C catalysts. Furthermore, the catalyst showed a high tolerance to CO poisoning. Siwal et al.^24^ developed the synthesis procedure to yield a metal–polymer nanocomposite, which shows excellent stability toward the electrocatalytic methanol oxidation in alkaline media. Shi et al. examined Pt nanoparticles deposited on different titanium oxide supports with respect to the influence of the electronic structure of the support on electrocatalytic activity in the MOR.^25^ The electronic properties of the support can be tailored by changing the number of oxygen vacancies or doping with fluorine. The authors also reported strong metal–support interactions, which were confirmed by charge transfer between the components of the system.

A theoretical study focused on the scaling relations of CO oxidation—which is one of the crucial steps in MOR—occurring on transition metal nanorods on SrTiO_3_ perovskite has been reported by Zhang et al.^26^ The authors claim that on a more reactive system such as Pd/SrTiO_3_, the oxidation of CO to CO_2_ involves the interface sites, via either the dissociative or associative pathways, and leads to high barriers due to strong O binding. Moreover, they predict that doping SrTiO_3_ with fluorine can enhance the catalytic performance of CO oxidation.

A similar catalytic system has been investigated experimentally by Chen et al.^27^ The authors synthesized SrTiO_3_ nanopolyhedra in a controlled way to achieve a uniform distribution of Pd nanoparticles. Based on the TOF and DRIFTS, the authors concluded that the CO oxidation activity is mostly dominated by the Pd size due to CO coverage effects.

Yoon et al. investigated the catalytic systems consisting of supported Pt nanoparticles with specific metal–support interactions as the main focus.^28^ The authors claim that the increased charge transfer between the reactants and the catalyst is responsible for the higher binding energies. Interestingly, the surface orientation of the supporting TiO_2_ was found to play an important role, which supports the assumptions of the present work.

## Aim and Scope of the Work

While Pd on its own is not a highly active MOR catalyst, we consider that the characteristics of the interaction with the supporting material can influence its properties. In order to determine the specific factors responsible for the catalytic effect, we investigated a system consisting of SrTiO_3_ perovskite as a supporting material for a thin Pd overlayer. We propose that the interface formed in such a way has unique characteristics due to the components influencing one other. Especially, the Pd bilayer is expected to show different properties compared to the pure Pd surface.^29,30^

Perovskite materials are suitable from this point of view, as they exhibit high thermal and chemical stability, and are catalytically active in MOR.^31^ Hence, in this work, we investigate the influence of the perovskite support on the alteration of the electron structure of the Pd catalyst, which is conceptually similar to the metal–support interactions.^32^ Similar modifications of the Pd catalytic properties supported on SrTiO_3_ have been reported for the CO catalytic oxidation by Pd nanoclusters.^26,27^ Our work builds on these results but uses a different model, wherein the perovskite is used as a support, fully covered with the Pd active phase, so it has no direct contact with the reaction intermediates. We propose that the role of SrTiO_3_ is not inert though it acts as a cocatalyst or a promoter.

In a broader scope, this study aims to investigate the role of the system components in the methanol electro-oxidation reaction, which will allow for the design of better catalytic systems. Perovskites seem to fit perfectly in this role, as they exhibit tunable properties,^33,34^ and upon modifying their structure, morphology, or simply composition, the promoting effect can be optimized for a given reaction. We intend to use the strength of computational modeling to accomplish this task, as this tool allows us to gain insights into the structure of the interface, whose intricacies are beyond the reach of experimental techniques.

## Computational Methods

### Model Description

We focus our analysis on the Pd/SrTiO_3_ interface and its role in the process efficiency. Despite a truly remarkable improvement in the synthesis of nanostructured material containing platinum group metals,^35,36^ the atomistic insights into the intricacies of such interfaces can only be gained from the quantum-chemical analysis.

All results presented in this work have been obtained using the DFT method as implemented in VASP code ver. 5.4.4.^37,38^ We used the Perdew–Burke–Ernzerhof (PBE) functional^39,40^ with an energy cutoff of 500 eV and the projector-augmented wave method^38,41^ to describe the electron–ion interactions. The Brillouin zone (BZ) was sampled by a 2 × 2 × 1 k-point grid generated with the Monkhorst–Pack scheme.^42^

The models were fully periodic and consisted of five atomic layers of a SrTiO_3_ (100) surface. In order to determine the influence of the chemical character of the support, we considered both possible terminations of the perovskite: SrO and TiO_2_. On top of a given perovskite surface, two layers of a palladium (100) surface were placed. The best match at the Pd and SrTiO_3_ interface was obtained by making use of the algorithm of Therrien et al.^43^

A vacuum slab of 16 Å thickness was created on top of the Pd layer in order to prevent the interaction across the periodic boxes. In total, the periodic box was tetragonal, with the cell lengths *a* = *b* = 15.780 Å, *c* = 32.000 Å, and angles α = β = γ = 90°.

For comparison purposes, the Pt (100) surface was constructed, and the MOR reaction was simulated with the same methodology. The functional, k-point sampling, ZPE correction, and solvent effects were calculated in the same way as for perovskite-containing systems.

### Computational Details

All systems were relaxed to reach forces smaller than 0.01 eV/Å, with the exception of three bottom layers of the perovskite, which were fixed to mimic the properties of the bulk. The transition state (TS) structures have been determined by means of the climbing image nudged elastic band (CI-NEB) method,^44^ as implemented in VTST Tools.^45^ The minimum energy path consisted of 16 images. The optimization was continued until the forces acting on the atoms were less than 0.05 eV/Å.

The geometries of the optimized systems were used to calculate zero-point energies (ZPE) using the finite displacement method with displacements of 0.0015 Å. The solvent effect was taken into account using the implicit solvation model that describes the effect of electrostatics, cavitation, and dispersion on the interaction between the solute and solvent, as implemented in the VASPsol package.^46,47^

Charge distribution was calculated with the DDEC6 method,^48−51^ using the Chargemol software.

### Electrocatalytic Process Description

Methanol oxidation half-reaction can be written as
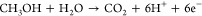
1

During each of its six steps, one electron and one proton are transferred to the electrode and the solvent, respectively. It has to be stressed that a stoichiometric amount of water is required for the process to occur, and the water molecule undergoes splitting, similar to the one in the oxygen evolution reaction (OER). The difference with the OER is that no O–O bond and rather a C–O bond is being formed. This leads to the unfavorable thermodynamics of the methanol electro-oxidation reaction, and the oxygen reduction reaction occurring on the cathode is the main driving force for the DMFC.^52^

**Figure 1:**
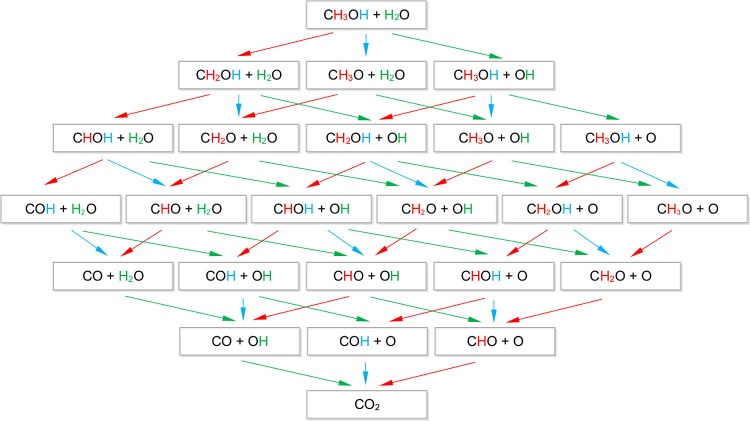
Network of all possible elementary steps of the methanol oxidation half-reaction. Red, blue, and green arrows represent the dissociation of the hydrogen atom bound to carbon, oxygen, and water, respectively.

The possible intermediates present in the mechanism are listed in Figure 1. All intermediates consisting of one system have been considered as separate models (for example, CH_3_OH + H_2_O were simulated as two separate systems), in order to avoid the interaction between them in a single periodic box. In the case of single intermediates, the energy of the empty surface has been added. Whenever possible, we also considered the possibility of dissociation of OH or O from the intermediate; for instance, COOH was considered as an alternative for CO + OH, and HCOO as an alternative for CHO + O.

In order to determine the energy changes within the system, we used the computational hydrogen electrode (CHE) methodology, as developed by No̷rskov et al.^53^ This method relies on two effects: one is that the free energy of a proton and an electron is equal to the free energy of half of the hydrogen molecule, due to the equilibrium of the proton/electron recombination reaction in standard conditions. In electrochemical reactions, each reaction step can be described by
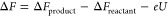
2where *U* is the potential applied, and *e* is the number of electrons transferred in the particular step, which is the second assumption of the method: the free energy of the intermediate is proportional to the applied potential.

To determine the optimal reaction pathway, we compared the relative free energies of each possible step in the reaction network, as shown in Figure 1.

## Results and Discussion

### Reaction Pathways

**Figure 2:**
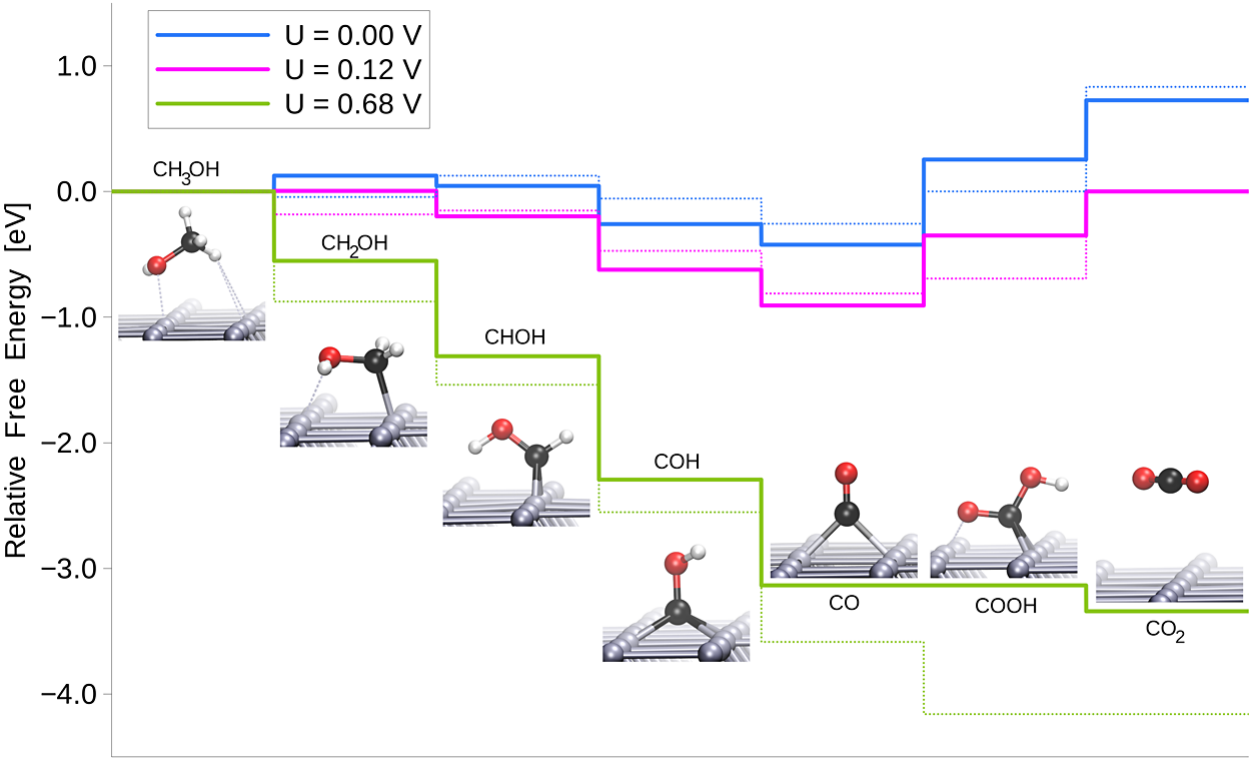
Energy profile of the methanol oxidation reaction occurring on Pd over the TiO_2_-terminated perovskite surface. The blue line represents the free energies with no external potential, the pink line represents the external potential equal to reaction potential, and the green line represents the potential required to make all consecutive reaction steps downhill. The dotted lines represent the same profiles for the Pt surface for the sake of comparison.

**Figure 3:**
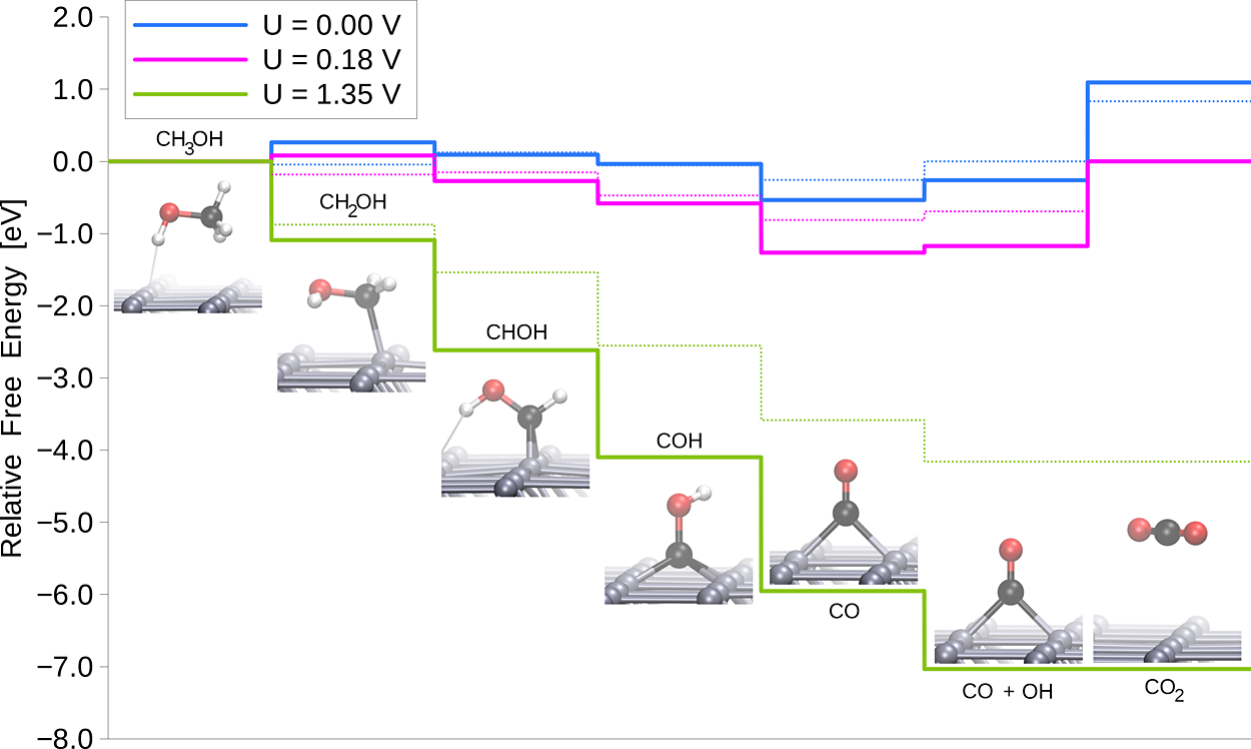
Energy profile of the methanol oxidation reaction occurring on Pd over the SrO-terminated perovskite surface. The blue line represents the free energies with no external potential, the pink line with the external potential equal to reaction potential, and the green line with the potential required to make all consecutive reaction steps downhill. The dotted lines represent the same profiles for the Pt surface for the sake of comparison.

We begin the analysis with a description of the reaction mechanism. The energy profiles for both the investigated systems are shown in Figures 2 and 3. As we mentioned before, a stoichiometric amount of water is needed for the reaction, and water molecules need to undergo splitting in the process. This is unfavorable from a thermodynamics point of view and results in preferred pathways, wherein the hydrogen atoms dissociate from methanol first. This can be observed for both investigated systems, with TiO_2_ and SrO terminations, implying that the effect of the water molecule dissociation is dominating the stability of the system. Compared to this, the effect of the metal–support interactions is minor. This observation coincides with the work of Mekazni et al.,^54^ where the DFT analysis revealed the importance of coadsorbed OH.

Along with the observation that the direct bond cleavage has more influence on the system stability than the metal–support interaction, the first bond that is cleaved is the one between C and H. The dissociation of the hydrogen leaves an unsaturated bond on the C atom, and a stronger interaction of the intermediate with the Pd overlayer is observed. However, this interaction does not compensate for the energy of the C–H bond, and the first step in the energy profile, the formation of the CH_2_OH intermediate, leads uphill. The subsequent steps to the CHOH, COH, and CO intermediates lead to the stabilization of the system and the corresponding decrease of potential energy.

This holds for both SrTiO_3_ terminations and is different than the results reported in the literature for Pt/Pd alloys,^55^ which assume the first step to be the dissociation of the oxygen-bound proton. On the other hand, Sheng et al.^56^ reported the same reaction pathway for a similar system consisting of two Pt monolayers supported on tungsten carbide. This difference has been attributed to the presence of OH surface species by Mekazni et al.^54^; however, our simulations consider only a single species, and we conclude that the factor playing the key role is the interaction with the surface.

Upon the formation of surface-bound CO species, the only H atoms that can participate in the process come from water molecules, which makes the subsequent steps thermodynamically unfavorable. This is also the step that makes the most significant difference for the systems with different terminations of the perovskite surface. In the case of the TiO_2_ termination, the most stable intermediate is a carboxyl group (−COOH), which means that the formation of the C–O bond is favored. To the best of our knowledge, this step has not been investigated computationally, as other mechanistic studies end at the carbonyl intermediate.^54−57^

Subsequently, CO_2_ is formed, which requires the cleavage of the Pd–C bonds and again is an unfavorable process. Thus, for the TiO_2_ termination of the perovskite, the optimal pathway leads via the following intermediates: CH_3_OH → CH_2_OH → CHOH → COH → CO → COOH → CO_2_. This pathway is mostly consistent with the one reported by Sheng et al.,^56^ with the exception of the CHO intermediate, which is more stable in their report, whereas COH has been determined to be more stable in our work.

The limiting step is CO → COOH, which involves water molecule dissociation. This step defines the reaction overpotential as η = 0.56 V. Together with the potential required to overcome the Δ*F* of the reaction, the onset potential amounts to η = 0.68 V. The value is within the range of 0.5 < η < 0.7 V reported for Pt-based catalysts in the literature^58^ and slightly smaller than the one calculated for the (110) surface of Pt (η = 0.83 V) using the same level of theory as a benchmark for this study.

Contrary to that, the most stable intermediate for the SrO-terminated perovskite surface consists of CO and OH separately bound to the Pd overlayer. This means that the hydroxyl bound to the Pd atom is slightly less stable than the one bound to the C atom of the CO intermediate, and the system is more stable despite the weakened C–Pd bond, which will be discussed in more detail in the following section. The C–O bond is formed in the last stage of the reaction, in the same step as the dissociation of the resulting CO_2_ from the surface—in agreement with the mechanism reported by Anderson et al.^59^ This complex process is the most unfavorable one of the whole mechanism and defines the reaction potential to η = 1.17 V, which is significantly more than that for the TiO_2_-terminated perovskite support.

Thus, the most optimal pathway leads via the following intermediates: CH_3_OH → CH_2_OH → CHOH → COH → CO + OH → CO_2_. The limiting step is CO + OH → CO_2_. The onset potential of the reaction amounts to η = 1.35 V.

The change of the most stable intermediate (CO + OH vs COOH) and the resulting change of the reaction potential are very significant, considering that the intermediates are interacting with the same Pd metal overlayer, and the composition of the whole investigated system remains unchanged. The change of the most stable intermediates suggests a different reaction mechanism is possible with respect to the OH formation at the support instead of the metal site, as reported by Scofield et al.^22^ Moreover, a similar effect has been described by Anderson et al.^59^ for the Pt catalysts alloyed with other metals based on the theoretical simulations. The authors claim that the interaction with the heteroatom is sufficient to facilitate the dissociation of hydrogen from water, yielding heteroatom-bound hydroxyl and Pt-bound hydrogen.

Interestingly, neither of the pathways is consistent with the mechanisms reported in the literature for Pd-based MOR reactions in alkaline media leading via formate.^60^ This implies that in alkaline media, different bonds are activated, and the C–H bond is cleaved last.

The comparison of the stability of the given intermediates for the supported Pd systems and the reference Pt (100) surface is shown in Figures 2 and 3 as dotted lines. Concerning the bias that needs to be applied for the reaction, the TiO_2_-terminated surface is characterized by better performance in terms of the overpotential. The reason is the slightly less stable −COOH intermediate, which makes the subsequent potential limiting step easier and therefore allows for a smaller overpotential (η = 0.56 V) of the reaction.

This is the opposite case for the SrO-terminated system, wherein the CO intermediate is significantly more stable due to a strong interaction with the Pd overlayer. This makes the following step significantly more difficult and results in a much higher (η = 1.17 V) overpotential.

### C–O Bond Formation

**Figure 4:**
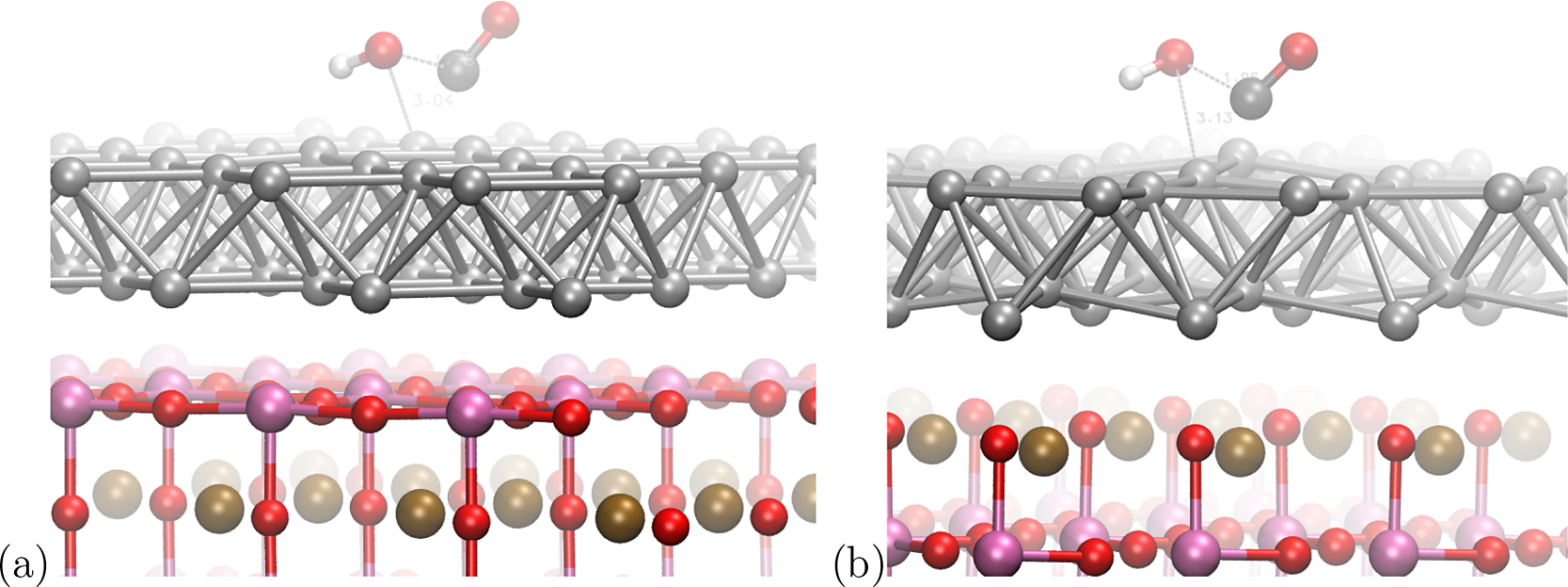
Transition state structures of the −COOH formation for the Ti-terminated (a) and Sr-terminated (b) systems. Pd atoms are shown in silver, Ti atoms in pink, Sr atoms in bronze, O atoms in red, C atoms in black, and H atoms in white colors.

**Figure 5:**
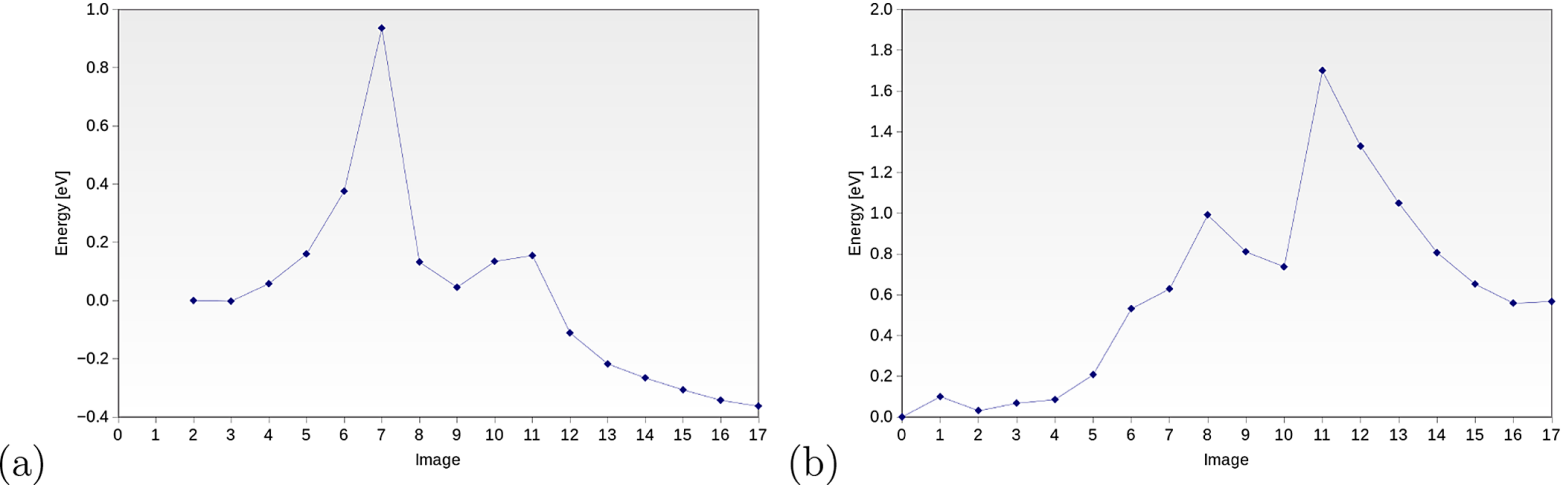
Energy profiles for −COOH formation for the Ti-terminated (a) and Sr-terminated (b) systems.

In this section, we present an analysis of the barrier heights accompanying the formation of the C–O bond. This is the step in the whole pathway wherein the CO and OH species recombine on the surface of the catalyst and is the only step not involving charge transfer. This step is crucial to complete the analysis. Figures 4 and 5 show the transition state structures and corresponding energy profiles of the COOH intermediate formation on the Ti- and Sr-terminated systems. It can be noted that the CO + OH → COOH reaction is significantly preferred kinetically on the Ti-terminated underlayer, with an activation energy of 0.93 eV. Contrary to that, the same reaction is accompanied by a prohibitive barrier of 1.70 eV, when taking place on the Sr-terminated surface.

It can also be noted that the dissociation of Pd–O and the formation of the C–O bond do not occur simultaneously but are two-step processes. The first maximum in the curves represents the dissociation of the −OH intermediate from the Pd overlayer and is a limiting step for the TiO_2_-terminated system. The second maximum is the formation of the C–O bond, which is a limiting step in the SrO-terminated system.

In addition, the thermodynamics of this step are favored in the TiO_2_-terminated system, where the COOH intermediate is more stable (Δ*G* = −0.36 eV). Negative Δ*G* means that the activation energy of the reverse process is higher and amounts to 1.29 eV. This is not the case for SrO termination, for which the COOH intermediate is less stable than dissociated CO and OH by Δ*G* = 0.56 eV. It implies that the formation of the C–O bond, which is a necessary step, is much more difficult not only from the thermodynamic but also from a kinetic point of view. Such a high barrier implies that the process can only take place on the system with the TiO_2_ termination of the Pd–SrTiO_3_ interface.

### Metal–Support Interactions

**Figure 6:**
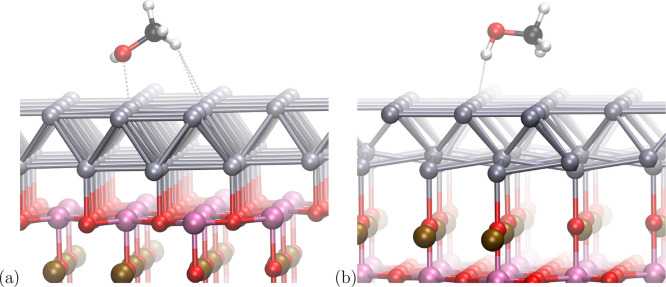
Methanol molecule interacting with the palladium overlayer supported on TiO_2_-terminated (a) and SrO-terminated (b) perovskite surfaces. Pd atoms are shown in silver, Ti – pink, Sr – bronze, O – red, C – black, and H – white colors.

As demonstrated experimentally on the Pt/CNT system,^61^ the interactions of the active phase with the support can have a significant influence on the efficiency of the MOR process. In order to explain the differences in the stability of the particular intermediates with Pd, we carried out an analysis of the interactions between the supporting perovskite and Pd overlayers. The geometries of the interfaces of differently terminated perovskite surfaces with Pd are shown in Figure 6 on the example of the system containing the methanol molecule.

The most striking difference is the distortion of the interface between the Sr-terminated perovskite and the Pd overlayer. The oxygen atoms from the SrO layer form a strong bond with the Pd atoms from the metal overlayer. The Pd atoms are attracted toward the perovskite, and the bottom metal layer is split into two types of atoms, Pd interacting with O, whose distance is *r* = 2.17 Å, and Pd being repelled from Sr, whose distance is significantly greater and amounts to *r* = 2.85 Å.

This effect is not visible for the TiO_2_-terminated surface; however, the distances between the Ti cations and the O anions are similar to those in the case of the SrO termination. This is due to the different structure of the TiO_2_ layer, where the Ti cation is not located directly below the Pd as is the case for the SrO termination. The Ti–Pd distance amounts to *r* = 2.85 Å and is the same as that of Sr–Pd in the SrO-terminated perovskite. The distance of O–Pd for the TiO_2_ termination is *r* = 2.12 Å (compared to *r* = 2.17 Å in the case of the SrO termination), which is not consistent with the bond orders (BOs) between the Pd and interface O atoms gathered in Table 1. Although the differences between the BOs are minor, slightly stronger binding is observed for the SrO-terminated surface (0.65–0.70) vs the TiO_2_-terminated one (0.61–0.64).

**Table 1:** Bond Orders Calculated for the Selected Bonds within the System with Different Terminations

	C–Pd	O–Pd	H_C_ – Pd	H_O_ – Pd	SBO C	SBO O	Pd–O_surf_
Sr termination							
CH_3_OH	0.0673	0.0367	0.2117	0.0940	3.9559	2.2684	0.6551
CH_2_OH	0.8027	0.2617	0.0337	0.0699	3.9201	2.3853	0.6752
CHOH	0.7908	0.0480	0.0290	0.0855	3.9797	2.3714	0.6652
COH	0.6452	0.0647		0.0384	3.9734	2.3724	0.7078
CO	0.8941	0.0435			4.1401	2.3121	0.6700
CO_2_	0.0624	0.0397			4.1741	2.1758	0.6492
Ti termination							
CH_3_OH	0.0369	0.4159	0.0382	0.0820	3.8502	2.4645	0.6282
CH_2_OH	0.7811	0.3110	0.0378	0.1039	3.8759	2.4114	0.6225
CHOH	0.7901	0.0511	0.0266	0.0829	3.9587	2.3631	0.6260
COH	0.6124	0.0651		0.0336	3.9458	2.3651	0.6438
CO	0.9091	0.0437			4.1373	2.3012	0.6268
COOH	0.6612	0.0645		0.0233	4.1459	2.4138	0.6294
CO_2_	0.0638	0.0406			4.1606	2.1664	0.6144

This, however, can be explained by the charge distribution within the system, as shown in Table 2. The Ti ions, which are formally on a +4 oxidation state, are characterized by the biggest values of the charges, especially in the inside layers—approximately 2.2. The Sr ions are formally on a +2 oxidation state, and their charges in the inside layers are approximately 1.5. The interface layers in these two terminations, however, behave differently—the Ti is reduced to a significantly greater degree than Sr, and their charges are 1.8 and 1.3, respectively. This means, that on average, the charge on the Ti atom was reduced by 0.37, and on Sr only by 0.17. At the same time, the interface Pd atoms in the case of TiO_2_ termination remain neutral with charges of −0.015, but in the case of SrO termination, the charges on the interfacial Pd vary from −0.16 to +0.07. The accumulation of positive charge on some Pd atoms leads to electrostatic repulsion with the interfacial Sr cations and distortion of the interfacial layer of Pd mentioned above.

**Table 2:** DDEC6 Charges Calculated for The SrTiO_3_/Pd Interface

layer	TiO_2_ termination	SrO termination
	charge on the Ti atom	charge on the Sr atom
1 (bottom)	2.134	1.465
2	2.204	1.500
3	2.195	1.504
4 (interface)	1.834	1.328
	charge on the Pd atom	charge on the Pd atom
5 (interface)	–0.015...–0.014	–0.162...0.069
6 (top)	–0.043...–0.030	–0.025...–0.024

Another important observation is the different orientation of the methanol molecule with respect to the surface. While in the case of the TiO_2_ termination, the hydroxyl group of the methanol is oriented laterally to the Pd surface, for the SrO termination, the hydroxyl group is oriented perpendicularly, with the hydrogen atom pointing toward the Pd, which is shown in Figure 6. This observation is reflected in the bond orders gathered in Table 1, which shows that the bond order between the oxygen atom and surface palladium atom is 0.0367 and 0.4159 for the SrO and TiO_2_ terminations, respectively.

This implied that the change of the character of the surface Pd atom is significant enough to influence the bonding of the intermediate with the surface. In the case of the TiO_2_ termination, the interaction with the lone pair of the electrons on the O atom is preferred, and in the case of the SrO termination, one with the partially positively charged H atom. Despite the fact that the partial charges borne by the surface Pd atoms are almost identical (see Table 2), the distribution of charge density is different.

**Figure 7:**
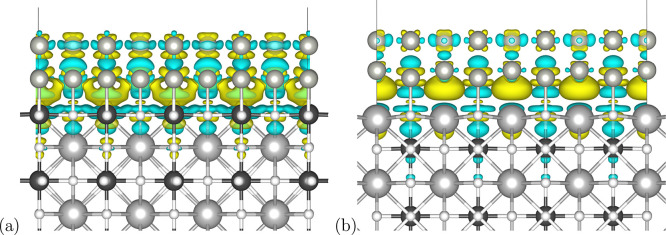
Charge density difference plots calculated with respect to the isolated TiO_2_-terminated (a) and SrO-terminated (b) perovskite nanoslabs and Pd overlayers. The blue and yellow colors denote charge depletion and accumulation, respectively. Isosurfaces are drawn for a 0.002 value.

Figure 7 shows the charge density difference for the Pd overlayer on the differently terminated perovskite surfaces, calculated with respect to the isolated perovskite and Pd slabs. Significant charge redistribution can be observed on the palladium atoms. It can be explained by the dipole moment induced by the interaction of the Pd with the O anions and Sr or Ti cations. In the case of the TiO_2_-terminated perovskite, the bigger volume is affected, and it can be expected that the charge redistribution occurs to a greater extent.

## Conclusions

An important conclusion is that the geometry of the metal–support interface is significantly more distorted in the case of the SrO-terminated system. It is a result of the charge transfer between the Pd overlayer and the perovskite support, leading to the formation of the partially positively charged Pd atoms at the interface with the support.

The charge difference density plots reveal that the promoting role of the support is a result of the distribution of electron density on the Pd overlayer. While the partial charge on the top Pd layer is almost identical in the systems with SrO and TiO_2_ terminations, the bond orders between the Pd atoms and the O atoms of the intermediates are greater in the case of the TiO_2_-terminated perovskite. Contrary to that, stronger binding of the carbon-bound H atom to Pd in the SrO-terminated system results in easier dissociation of this atom from the reactive species.

The limiting step is the formation of the surface-bound CO and OH species in the case of the SrO-terminated system, which leads to an excessive stabilization of this system and makes the final step of CO_2_ formation difficult. On the other hand, the most stable intermediate in the case of TiO_2_ termination is carboxyl, which is easily converted to CO_2_ and requires a lower overpotential.

Importantly, the computational model presented in this work does not consider the kinetic barriers of proton transfers, as they are currently inaccessible even with the state-of-the-art method. A consequence of the separation of the redox half-reactions is that the positive and negative charge transfers are also separated. Protons migrate through the solution via the Grothuss mechanism,^62^ which, in the implicit solvent approach, implies a change in the composition of the system and thus the inability to construct the NEB. The explicit solvent method, on the other hand, is dynamic by nature, and isolating the elementary step of the process for the investigation is virtually impossible.

One way to tackle this issue is to estimate the transition state energy by making an assumption that the reaction involves the surface hydrogen atom.^63^ The kinetic barriers of a similar process have been determined to be approximately 0.4 eV, which is below the overpotentials determined in the present work −0.68 and 1.35 eV. Thus, we conclude that the kinetics of the process will not play a significant role, as in the process conditions, the barriers can be crossed with ease; hence, the assumptions of the computational procedure are justified.

Since pure Pd is not the optimal catalyst for methanol electro-oxidation, in this work, we explored the possibility of increasing its activity by utilizing specially crafted supports. The concept of a cocatalyst is not new, and with respect to this, the results obtained in this work shed light on the cocatalytic effect of the support on the activity of the Pd overlayer in the methanol electrocatalytic oxidation. We observed significant differences in the overpotential calculated for the differently terminated but otherwise identical systems. We conclude that this is related to the reducibility of Ti in the interfacial layer of the support, and the support is able to play an important role in the efficiency of the catalytic process, despite the fact that it is not directly interacting with the intermediates.

More importantly, however, we demonstrated an important effect of the underlying perovskite despite it being indirect and not involving any change to the chemical composition of the system. This opens the way for further investigations of the role of the supporting material in the process, such as composition, substitutions, or vacancies, in order to improve the efficiency of the methanol electro-oxidation. Although significant efforts are needed, perhaps even the application of abundant elements instead of noble metals without sacrificing too much efficiency will become possible.

## Supporting Information Available

Geometries of the optimized structures and DDEC6 charges (PDF)
