# Supplementary material for: On the Design of the Metal–Support Interface in Methanol Electrocatalytic Oxidation
Source: Cryst Growth Des. 2024 Oct 11;24(21):8709–18. doi: 10.1021/acs.cgd.3c01466 (PMC11544584; doi:10.1021/acs.cgd.3c01466)
Supplement: Supplementary file 1 — cg3c01466_si_001.pdf [file cg3c01466_si_001.pdf]

On the Design of Metal-Support Interface in Methanol Electrocatalytic Oxidation

Bartłomiej M. Szyja* and Joanna Zasada

Faculty of Chemistry, Wrocław University of Science and Technology, Gdańska 7/9, 50-344 Wrocław, Poland

E-mail: b.m.szyja@pwr.edu.pl

Contents

1 Coordinates and DDEC6 charges in xyz format 3

1.1 SrO-Pd interface 3

1.1.1 CH_2O 3

1.1.2 CHO 8

1.1.3 HCOO 12

1.1.4 CH_3O 17

1.1.5 COOH 22

1.1.6 CO 27

1.1.7 COHOH 32

1.1.8 CH_2OH 37

1.1.9 CH_3OH 42

1.1.10 $CH_2O(OH)$ 47

1.1.11 CH(OH)(OH) 52

1.1.12 COH 56
1.1.13 CHOH 61
1.1.14 CO_2 66
1.2 TiO_2−Pd interface 71
1.2.1 CH_2O 71
1.2.2 CHO 76
1.2.3 HCOO 81
1.2.4 CH_3O 86
1.2.5 COOH 92
1.2.6 CO 97
1.2.7 CH_2OH 102
1.2.8 CH_3OH 107
1.2.9 $CH_2O(OH)$ 112
1.2.10 CH(OH)(OH) 117
1.2.11 COH 122
1.2.12 CHOH 127
1.2.13 CO_2 133

1 Coordinates and DDEC6 charges in xyz format

1.1 SrO-Pd interface

1.1.1 CH_2O

```
      340
jmolscript: load "" {1 1 1} spacegroup "x,y,z" unitcell [{     15.780520      0.000000      0.000000 }, {      0.000000     15.780520      0.000000 }, {      0.000000      0.000000     32.000000 }]
O        0.000000      1.972565      1.972576        1.922884
O        0.000000      5.917695      1.972576        1.922956
O        0.000000      9.862825      1.972576        1.922967
O        0.000000     13.807955      1.972576        1.922844
O        1.951893      5.918247     11.807680        1.945410
O        1.958299      1.977189     11.804480        1.944063
O        1.951372      9.865713     11.807392        1.944846
O        1.957558     13.807671     11.805664        1.943877
O        1.989434      0.000852      9.816160        1.982803
O        1.943371      3.946566      9.813440        1.983153
O        1.942519     11.835816      9.813632        1.983322
O        1.990839      7.891033      9.817184        1.982939
O        1.967026      1.972218      7.866432        1.901482
O        1.971271      5.920283      7.868320        1.899770
O        1.971334      9.861783      7.867840        1.900578
O        1.966032     13.809801      7.866880        1.901258
O        2.116862      3.946014      5.905344        1.919392
O        1.820788      0.000458      5.903808        1.919534
O        1.820078      7.890702      5.904064        1.919658
O        2.116436     11.835437      5.905504        1.919505
O        1.972565      0.000000      1.972576        1.922868
O        1.972565      1.972565      3.945120        1.915089
O        1.972565      1.972565      0.000000        1.520373
O        1.972565      3.945130      1.972576        1.922907
O        1.972565      5.917695      3.945120        1.914581
O        1.972565      5.917695      0.000000        1.520361
O        1.972565      7.890260      1.972576        1.922887
O        1.972565      9.862825      0.000000        1.520417
O        1.972565      9.862825      3.945120        1.914681
O        1.972565     11.835390      1.972576        1.922941
O        1.972565     13.807955      0.000000        1.520401
O        1.972565     13.807955      3.945120        1.914940
O        3.938139      1.996709      9.814880        1.983140
O        3.937445     13.785610      9.814976        1.983160
O        3.939449      5.895334      9.825600        1.983048
O        3.938045      9.887017      9.823392        1.983337
O        3.941879      1.825364      5.904544        1.919669
O        3.941753     13.956339      5.904960        1.919739
O        3.942163      6.067215      5.903904        1.920009
O        3.941769      9.714283      5.902976        1.920218
O        3.945130      1.972565      1.972576        1.922894
O        3.945130      5.917695      1.972576        1.922830
O        3.945130      9.862825      1.972576        1.922782
O        3.945130     13.807955      1.972576        1.922905
O        5.903556      1.969977     11.806528        1.945459
O        5.919904      5.933476     11.814464        1.942555
O        5.902767     13.811758     11.807520        1.944064
O        5.911525      9.859527     11.814048        1.942337
O        5.888848      0.000726      9.812832        1.982898
O        5.932718     11.835295      9.820800        1.983335
O        5.933397      3.948034      9.823328        1.982813
O        5.887002      7.891854      9.813216        1.984373
O        5.906728      9.866044      7.869248        1.900722
O        5.912172     13.805146      7.867904        1.900496
```

O	5.911967	1.977899	7.867808	1.900635
O	5.908842	5.917016	7.870304	1.899968
O	6.061534	0.000584	5.907520	1.919009
O	5.765965	11.835327	5.904160	1.920086
O	5.766155	3.946550	5.904256	1.919972
O	6.062118	7.890686	5.906944	1.918935
O	5.917695	1.972565	3.945120	1.914505
O	5.917695	3.945130	1.972576	1.922819
O	5.917695	5.917695	3.945120	1.915203
O	5.917695	5.917695	0.000000	1.520287
O	5.917695	7.890260	1.972576	1.922967
O	5.917695	9.862825	0.000000	1.520376
O	5.917695	9.862825	3.945120	1.915026
O	5.917695	11.835390	1.972576	1.922830
O	5.917695	13.807955	0.000000	1.520413
O	5.917695	13.807955	3.945120	1.914733
O	5.917695	0.000000	1.972576	1.922944
O	5.917695	1.972565	0.000000	1.520367
O	7.882401	13.829921	9.816384	1.983047
O	7.882733	1.952161	9.815392	1.983253
O	7.881107	9.841269	9.809920	1.984601
O	7.883411	5.940514	9.811168	1.984710
O	7.886520	13.660502	5.904288	1.919880
O	7.886678	2.121154	5.904160	1.919736
O	7.886552	10.010846	5.907744	1.918934
O	7.886709	5.770621	5.907584	1.918938
O	7.890260	1.972565	1.972576	1.922910
O	7.890260	5.917695	1.972576	1.923018
O	7.890260	9.862825	1.972576	1.923038
O	7.890260	13.807955	1.972576	1.922912
O	9.825362	9.849001	11.816832	1.941596
O	9.848260	13.815577	11.807808	1.944365
O	9.847060	1.967563	11.808288	1.945213
O	9.837481	5.932166	11.815904	1.943338
O	9.833394	11.834080	9.826272	1.983078
O	9.880436	7.891049	9.812064	1.984298
O	9.878842	0.000647	9.811104	1.982311
O	9.834152	3.947450	9.824576	1.983714
O	9.859558	5.915659	7.870208	1.900112
O	9.859527	9.866739	7.870752	1.899401
O	9.855692	1.978356	7.868352	1.900383
O	9.854793	13.802353	7.868384	1.900032
O	9.710417	7.890781	5.907872	1.919146
O	10.006633	11.834980	5.903584	1.920216
O	10.006570	3.946314	5.903840	1.920104
O	9.711095	0.000347	5.908320	1.919254
O	9.862825	1.972565	3.945120	1.914654
O	9.862825	5.917695	3.945120	1.915464
O	9.862825	5.917695	0.000000	1.520218
O	9.862825	7.890260	1.972576	1.923007
O	9.862825	9.862825	0.000000	1.520292
O	9.862825	9.862825	3.945120	1.915469
O	9.862825	11.835390	1.972576	1.922806
O	9.862825	13.807955	0.000000	1.520375
O	9.862825	13.807955	3.945120	1.914502
O	9.862825	0.000000	1.972576	1.922983
O	9.862825	1.972565	0.000000	1.520320
O	9.862825	3.945130	1.972576	1.922785
O	-0.003708	13.659729	5.904640	1.919817
O	-0.003614	2.121801	5.904448	1.919749
O	-0.004150	10.011367	5.908576	1.919118
O	-0.003582	5.769942	5.908064	1.919090
O	-0.007874	13.831563	9.814848	1.983030
O	-0.007448	1.950015	9.813504	1.983106
O	-0.008064	9.838854	9.809696	1.982790
O	-0.007638	5.942376	9.812064	1.982828
O	11.824549	9.886496	9.828320	1.982650
O	11.826285	5.894829	9.823680	1.983859
O	11.827058	1.996835	9.814816	1.983034
O	11.826932	13.784852	9.814208	1.983430
O	11.830624	9.713904	5.903296	1.920654
O	11.831098	6.067421	5.903904	1.920234

129	O	11.831098	1.824922	5.905088	1.919958
130	O	11.831145	13.956671	5.905376	1.919862
131	O	11.835390	1.972565	1.972576	1.922918
132	O	11.835390	5.917695	1.972576	1.922825
133	O	11.835390	9.862825	1.972576	1.922800
134	O	11.835390	13.807955	1.972576	1.922912
135	O	13.797287	5.922224	11.808192	1.944560
136	O	13.800822	9.862588	11.806592	1.945168
137	O	13.792459	1.973386	11.803040	1.943967
138	O	13.790707	13.809154	11.804128	1.943738
139	O	13.775652	7.890781	9.813952	1.983556
140	O	13.824525	3.946582	9.816320	1.982612
141	O	13.823862	11.835295	9.815168	1.982589
142	O	13.777972	0.000552	9.812768	1.983133
143	O	13.796972	5.917758	7.868224	1.900481
144	O	13.793721	9.863867	7.868000	1.900369
145	O	13.800649	1.974727	7.866144	1.901475
146	O	13.801548	13.807450	7.866368	1.901577
147	O	13.952347	7.890544	5.905696	1.919342
148	O	13.655342	3.946061	5.904096	1.919744
149	O	13.655294	11.835406	5.904288	1.919678
150	O	13.951810	0.000442	5.905024	1.919497
151	O	13.807955	5.917695	3.945120	1.914679
152	O	13.807955	7.890260	1.972576	1.922959
153	O	13.807955	9.862825	0.000000	1.520387
154	O	13.807955	9.862825	3.945120	1.914304
155	O	13.807955	11.835390	1.972576	1.922902
156	O	13.807955	13.807955	0.000000	1.520394
157	O	13.807955	13.807955	3.945120	1.915008
158	O	13.807955	0.000000	1.972576	1.922904
159	O	13.807955	1.972565	3.945120	1.914720
160	O	13.807955	1.972565	0.000000	1.520371
161	O	13.807955	3.945130	1.972576	1.922880
162	O	13.807955	5.917695	0.000000	1.520324
163	Pd	1.907707	3.952563	16.156224	2.955138
164	Pd	1.907707	0.007417	16.156736	2.957362
165	Pd	1.907944	11.841734	16.156416	2.959086
166	Pd	1.908023	7.896872	16.156672	2.964084
167	Pd	1.913104	1.980850	13.985568	3.082730
168	Pd	1.913104	13.814504	13.985888	3.084961
169	Pd	1.913151	5.925427	13.985600	3.086181
170	Pd	1.913514	9.869974	13.985664	3.083443
171	Pd	3.879246	0.007654	14.412608	3.483576
172	Pd	3.879373	3.955040	14.413024	3.487305
173	Pd	3.879814	11.842980	14.413984	3.486140
174	Pd	3.879846	7.897330	14.413184	3.483432
175	Pd	3.885290	9.869863	16.156672	2.966701
176	Pd	3.885637	5.925759	16.158432	2.973890
177	Pd	3.885653	13.814883	16.156928	2.959352
178	Pd	3.885764	1.981702	16.155904	2.956101
179	Pd	5.851685	0.007433	16.158688	2.957231
180	Pd	5.851874	3.952594	16.158144	2.971163
181	Pd	5.851906	11.841434	16.158688	2.963507
182	Pd	5.852095	7.897156	16.158560	3.215945
183	Pd	5.857240	1.980818	13.985280	3.084101
184	Pd	5.857366	13.814504	13.985664	3.082442
185	Pd	5.857650	5.925443	13.985344	3.097894
186	Pd	5.857729	9.869832	13.985472	3.086472
187	Pd	7.821836	0.007780	14.412480	3.482747
188	Pd	7.821899	11.842775	14.413728	3.480679
189	Pd	7.822230	3.954456	14.412928	3.485456
190	Pd	7.822309	7.897393	14.412800	3.439717
191	Pd	7.829395	5.925569	16.158176	3.076805
192	Pd	7.829505	13.814914	16.156640	2.966874
193	Pd	7.829552	1.981449	16.156128	2.960902
194	Pd	7.829789	9.869753	16.156736	3.332456
195	Pd	9.795647	3.952357	16.157216	2.932468
196	Pd	9.795805	11.841955	16.156800	2.972213
197	Pd	9.795821	0.007417	16.157056	2.956625
198	Pd	9.795900	7.896856	16.157152	3.181766
199	Pd	9.801960	5.925317	13.985600	3.100659
200	Pd	9.802007	1.980597	13.985568	3.084866

201	Pd	9.802023	13.814662	13.985792	3.082624
202	Pd	9.802307	9.869895	13.985600	3.095557
203	Pd	11.767045	3.954535	14.413856	3.489569
204	Pd	11.767360	0.007606	14.413312	3.483589
205	Pd	11.767439	7.897298	14.413920	3.490297
206	Pd	11.767486	11.842744	14.414624	3.488570
207	Pd	11.774193	5.925554	16.158144	2.927539
208	Pd	11.774304	9.869816	16.156480	2.970570
209	Pd	11.774383	1.981386	16.156064	2.958946
210	Pd	11.774777	13.814804	16.156672	2.956455
211	Pd	13.740414	3.952421	16.156992	2.957108
212	Pd	13.740856	11.841797	16.156992	2.954858
213	Pd	13.740856	0.007669	16.157312	2.956732
214	Pd	13.740935	7.897172	16.156896	2.960137
215	Pd	13.746900	5.925333	13.985824	3.084179
216	Pd	13.746916	1.980597	13.985888	3.081549
217	Pd	13.747011	13.814567	13.986144	3.083295
218	Pd	13.747358	9.869737	13.985824	3.081378
219	Pd	15.712695	0.007638	14.412032	3.480726
220	Pd	15.712727	3.954693	14.412576	3.482647
221	Pd	15.713137	11.842965	14.413312	3.483161
222	Pd	15.713437	7.897345	14.412704	3.483435
223	Pd	15.720586	5.925664	16.158688	2.954660
224	Pd	15.721043	9.870005	16.156928	2.957156
225	Pd	15.721185	13.815135	16.157216	2.955496
226	Pd	15.721359	1.981513	16.156416	2.957468
227	Sr	0.000000	0.000000	0.000000	1.317693
228	Sr	0.000000	0.000000	3.945120	1.582872
229	Sr	0.000000	3.945130	0.000000	1.317677
230	Sr	0.000000	3.945130	3.945120	1.582601
231	Sr	0.000000	7.890260	0.000000	1.317717
232	Sr	0.000000	7.890260	3.945120	1.582669
233	Sr	0.000000	11.835390	0.000000	1.317732
234	Sr	0.000000	11.835390	3.945120	1.582415
235	Sr	3.946045	7.890812	11.562208	1.817340
236	Sr	3.941611	3.942163	11.564416	1.817954
237	Sr	3.944341	-0.000252	11.561056	1.819870
238	Sr	3.941169	11.837331	11.563424	1.818736
239	Sr	3.942873	7.890607	7.827136	1.585507
240	Sr	3.943157	3.944451	7.828288	1.584423
241	Sr	3.943047	11.835437	7.827968	1.584529
242	Sr	3.943599	-0.000174	7.827168	1.584968
243	Sr	3.945130	0.000000	0.000000	1.317713
244	Sr	3.945130	0.000000	3.945120	1.582751
245	Sr	3.945130	3.945130	0.000000	1.317696
246	Sr	3.945130	3.945130	3.945120	1.582836
247	Sr	3.945130	7.890260	0.000000	1.317767
248	Sr	3.945130	7.890260	3.945120	1.583202
249	Sr	3.945130	11.835390	0.000000	1.317792
250	Sr	3.945130	11.835390	3.945120	1.582764
251	Sr	7.889013	3.949691	11.557824	1.819040
252	Sr	7.888145	7.891159	11.568768	1.824494
253	Sr	7.889581	0.000663	11.562432	1.820348
254	Sr	7.885147	11.833133	11.562144	1.818792
255	Sr	7.888051	7.890165	7.827936	1.584706
256	Sr	7.888603	3.944120	7.827008	1.585208
257	Sr	7.888130	-0.000174	7.828160	1.584974
258	Sr	7.888067	11.836226	7.827808	1.585265
259	Sr	7.890260	0.000000	0.000000	1.317726
260	Sr	7.890260	0.000000	3.945120	1.582017
261	Sr	7.890260	3.945130	0.000000	1.317668
262	Sr	7.890260	3.945130	3.945120	1.582643
263	Sr	7.890260	7.890260	0.000000	1.317751
264	Sr	7.890260	7.890260	3.945120	1.582395
265	Sr	7.890260	11.835390	0.000000	1.317789
266	Sr	7.890260	11.835390	3.945120	1.582633
267	Sr	-0.001783	7.890118	7.827296	1.584467
268	Sr	-0.001262	3.944593	7.827712	1.585083
269	Sr	-0.001752	11.835753	7.827488	1.585036
270	Sr	-0.002099	-0.000142	7.826912	1.585153
271	Sr	-0.001262	11.834333	11.561088	1.819959
272	Sr	-0.000426	3.945146	11.561312	1.819784

Sr	-0.001499	-0.000063	11.560992	1.817839
Sr	-0.002146	7.890292	11.562528	1.820995
Sr	11.830009	7.887656	11.558432	1.817796
Sr	11.833986	-0.000395	11.561312	1.820170
Sr	11.836826	11.838956	11.564256	1.817272
Sr	11.836779	3.942716	11.564256	1.817447
Sr	11.834033	-0.000316	7.827040	1.584587
Sr	11.834080	3.944262	7.827968	1.584824
Sr	11.834080	11.835485	7.828064	1.584716
Sr	11.834333	7.890150	7.826528	1.585756
Sr	11.835390	0.000000	0.000000	1.317682
Sr	11.835390	0.000000	3.945120	1.582779
Sr	11.835390	3.945130	0.000000	1.317635
Sr	11.835390	3.945130	3.945120	1.582913
Sr	11.835390	7.890260	0.000000	1.317682
Sr	11.835390	7.890260	3.945120	1.583236
Sr	11.835390	11.835390	0.000000	1.317732
Sr	11.835390	11.835390	3.945120	1.582926
Ti	1.968904	9.862604	9.852064	2.646763
Ti	1.969614	5.918074	9.852736	2.645622
Ti	1.969330	13.807971	9.851104	2.646198
Ti	1.969693	1.972581	9.850688	2.647062
Ti	1.973464	9.862068	5.902304	2.606092
Ti	1.973322	5.917474	5.902368	2.605755
Ti	1.972991	13.807845	5.901952	2.606438
Ti	1.972960	1.971760	5.901760	2.606281
Ti	1.972565	1.972565	1.972576	2.636952
Ti	1.972565	5.917695	1.972576	2.636921
Ti	1.972565	9.862825	1.972576	2.636927
Ti	1.972565	13.807955	1.972576	2.636948
Ti	5.915359	5.919257	9.851392	2.645841
Ti	5.914570	9.862541	9.851136	2.645444
Ti	5.914981	1.972723	9.852544	2.646256
Ti	5.914649	13.808129	9.852160	2.646057
Ti	5.917695	1.972565	1.972576	2.636940
Ti	5.917695	5.917695	1.972576	2.636905
Ti	5.917695	9.862825	1.972576	2.636910
Ti	5.917695	13.807955	1.972576	2.636933
Ti	5.917537	5.916511	5.901632	2.605731
Ti	5.917900	9.862683	5.901856	2.606057
Ti	5.918168	1.972265	5.902400	2.606195
Ti	5.918090	13.806771	5.902240	2.605927
Ti	9.860142	1.972391	9.852352	2.645505
Ti	9.859006	5.918721	9.850784	2.644833
Ti	9.859464	13.807908	9.852928	2.645859
Ti	9.857539	9.862494	9.851424	2.644588
Ti	9.862825	5.917695	1.972576	2.636899
Ti	9.862825	9.862825	1.972576	2.636894
Ti	9.862825	13.807955	1.972576	2.636924
Ti	9.862825	1.972565	1.972576	2.636944
Ti	9.863425	1.971792	5.902272	2.606130
Ti	9.863362	13.807229	5.902496	2.606156
Ti	9.863740	5.917016	5.901504	2.605644
Ti	9.863882	9.862336	5.901472	2.605784
Ti	13.804815	13.808144	9.850912	2.646773
Ti	13.805178	1.972549	9.851296	2.646628
Ti	13.804562	9.862494	9.852960	2.645524
Ti	13.805225	5.917995	9.851968	2.645857
Ti	13.808460	13.806992	5.901920	2.606386
Ti	13.808586	1.972391	5.902176	2.606489
Ti	13.808350	9.862683	5.902624	2.606238
Ti	13.808160	5.916653	5.902208	2.605859
Ti	13.807955	5.917695	1.972576	2.636938
Ti	13.807955	9.862825	1.972576	2.636928
Ti	13.807955	13.807955	1.972576	2.636951
Ti	13.807955	1.972565	1.972576	2.636953
C	7.527734	8.469295	17.713696	3.934799
H	6.459235	8.125832	17.966144	1.058679
H	7.812778	9.261635	18.438912	0.970609
O	8.405131	7.435244	17.663744	2.434857

1.1.2 CHO

```
       339
jmolscript: load "" {1 1 1} spacegroup "x,y,z" unitcell [{     15.780520      0.000000      0.000000 }, {      0.000000     15.780520      0.000000 }, {      0.000000      0.000000     32.000000 }]
O        1.972565      1.972565      3.945120        1.917054
O        1.970545      1.974790      7.860992        1.905691
O        1.978372      1.963665     11.793760        1.946959
O        1.972565      1.972565      0.000000        1.520210
O        1.972565      3.945130      1.972576        1.922812
O        2.103827      3.945240      5.901024        1.922994
O        1.951309      3.945035      9.809536        1.983679
O        1.972565      5.917695      3.945120        1.917040
O        1.968651      5.916196      7.863104        1.904852
O        1.978420      5.920362     11.798496        1.955669
O        1.972565      5.917695      0.000000        1.520133
O        1.972565      7.890260      1.972576        1.922942
O        1.840245      7.889581      5.902432        1.922466
O        1.991833      7.889723      9.806336        1.984114
O        1.968494      9.862573      7.863392        1.902960
O        1.974743      9.857617     11.799712        1.946383
O        1.972565      9.862825      0.000000        1.520262
O        1.972565      9.862825      3.945120        1.916169
O        1.972565      0.000000      1.972576        1.922917
O        1.840671     -0.000284      5.903360        1.922173
O        1.992527     -0.000789      9.800608        1.983734
O        1.972565     11.835390      1.972576        1.922850
O        2.104317     11.834317      5.900960        1.923161
O        1.950283     11.834080      9.809216        1.983594
O        1.972597     13.809202     11.794784        1.945645
O        1.972565     13.807955      0.000000        1.520239
O        1.972565     13.807955      3.945120        1.916999
O        1.970592     13.803789      7.861088        1.905855
O        3.945130      1.972565      1.972576        1.922842
O        3.945083      1.840877      5.902432        1.922521
O        3.945240      1.994027      9.801696        1.984038
O        3.945004      6.048957      5.904768        1.921678
O        3.944436      5.896691      9.802176        1.983709
O        3.945130      5.917695      1.972576        1.922856
O        3.944420      9.730048      5.902976        1.922608
O        3.943615      9.883150      9.806304        1.983764
O        3.945130      9.862825      1.972576        1.922858
O        3.944609     13.786793      9.803840        1.983506
O        3.945130     13.807955      1.972576        1.922890
O        3.945240     13.939154      5.902976        1.922452
O        5.917695      1.972565      3.945120        1.917191
O        5.919983      1.971855      7.861024        1.905267
O        5.914444      1.970482     11.795072        1.944868
O        5.917695      3.945130      1.972576        1.922854
O        5.786133      3.945098      5.901920        1.922541
O        5.938241      3.944862      9.806240        1.983943
O        5.917695      5.917695      3.945120        1.917091
O        5.921735      5.916953      7.862752        1.904681
O        5.907311      5.915628     11.797248        1.953844
O        5.917695      5.917695      0.000000        1.520164
O        5.917695      7.890260      1.972576        1.922871
O        6.049336      7.889771      5.901888        1.922484
O        5.896817      7.889345      9.805664        1.985093
O        5.916401      9.861720      7.861952        1.905526
O        5.914618      9.857996     11.796352        1.955121
O        5.917695      9.862825      0.000000        1.520222
O        5.917695      9.862825      3.945120        1.917970
O        6.049778     -0.000347      5.901856        1.922660
O        5.897165     -0.000442      9.805888        1.983930
O        5.917695     11.835390      1.972576        1.922889
O        5.785817     11.834569      5.902304        1.922313
O        5.937452     11.834759      9.806912        1.983849
O        5.913923     13.805462     11.796832        1.952199
O        5.917695     13.807955      0.000000        1.520203
```

O	5.917695	13.807955	3.945120	1.917702
O	5.919589	13.806977	7.862304	1.904501
O	5.917695	1.972565	0.000000	1.520247
O	5.917695	0.000000	1.972576	1.922868
O	7.890260	1.972565	1.972576	1.922837
O	7.890875	2.104743	5.901600	1.922960
O	7.890607	1.953518	9.808256	1.983688
O	7.891286	5.785675	5.902752	1.922522
O	7.891002	5.936868	9.815552	1.983475
O	7.890260	5.917695	1.972576	1.922817
O	7.889913	9.993582	5.902208	1.922568
O	7.888650	9.843194	9.805632	1.985792
O	7.890260	9.862825	1.972576	1.922826
O	7.889771	13.829432	9.808960	1.983900
O	7.890260	13.807955	1.972576	1.922819
O	7.890402	13.675383	5.900544	1.922838
O	9.862825	1.972565	3.945120	1.916339
O	9.862888	1.977457	7.863392	1.903328
O	9.863440	1.965180	11.799104	1.950147
O	9.995886	3.945714	5.901344	1.922945
O	9.844015	3.946676	9.817408	1.983853
O	9.862825	5.917695	3.945120	1.916909
O	9.863014	5.914791	7.867072	1.902096
O	9.870747	5.928221	11.807680	1.950633
O	9.862825	5.917695	0.000000	1.520131
O	9.862825	7.890260	1.972576	1.922949
O	9.731484	7.889361	5.903872	1.922188
O	9.881525	7.890228	9.805536	1.985298
O	9.861468	9.863188	7.861664	1.905481
O	9.859432	9.860458	11.796096	1.948311
O	9.862825	9.862825	0.000000	1.520273
O	9.862825	9.862825	3.945120	1.916764
O	9.883008	0.000694	9.804160	1.984015
O	9.862825	11.835390	1.972576	1.922794
O	9.994797	11.834822	5.901696	1.922676
O	9.840937	11.836037	9.807520	1.985250
O	9.865792	13.810922	11.796448	1.953543
O	9.862825	13.807955	0.000000	1.520221
O	9.862825	13.807955	3.945120	1.917604
O	9.860821	13.806566	7.862112	1.905864
O	9.862825	1.972565	0.000000	1.520248
O	9.862825	3.945130	1.972576	1.922825
O	9.862825	0.000000	1.972576	1.922936
O	9.731342	0.000347	5.904320	1.921986
O	0.000000	1.972565	1.972576	1.922897
O	-0.000805	2.104222	5.901664	1.922762
O	-0.000537	1.952839	9.808928	1.983544
O	-0.001278	5.785454	5.902848	1.922495
O	-0.000868	5.938652	9.813280	1.984023
O	0.000000	5.917695	1.972576	1.922845
O	-0.001089	9.993945	5.902208	1.922768
O	-0.002588	9.840511	9.816384	1.983167
O	0.000000	9.862825	1.972576	1.922891
O	-0.000552	13.828422	9.807168	1.984058
O	0.000000	13.807955	1.972576	1.922841
O	-0.000537	13.675162	5.900768	1.922636
O	11.835390	1.972565	1.972576	1.922930
O	11.835579	1.840419	5.902432	1.922567
O	11.835090	1.993979	9.807840	1.984018
O	11.835690	6.049715	5.904800	1.921614
O	11.835769	5.896628	9.813184	1.982803
O	11.835390	5.917695	1.972576	1.922968
O	11.835737	9.730127	5.903808	1.922079
O	11.833922	9.884523	9.806048	1.985342
O	11.835390	9.862825	1.972576	1.922928
O	11.835295	13.787866	9.804128	1.984613
O	11.835390	13.807955	1.972576	1.922926
O	11.835248	13.938933	5.902912	1.921900
O	13.807750	1.975090	7.863232	1.904221
O	13.803899	1.965558	11.798880	1.954691
O	13.675493	3.945745	5.903392	1.922243
O	13.827270	3.945162	9.812544	1.984359

O	13.807955	5.917695	3.945120	1.917601
O	13.807971	5.917237	7.867744	1.902550
O	13.808318	5.913813	11.807840	1.961251
O	13.807955	7.890260	1.972576	1.922942
O	13.939580	7.889629	5.904384	1.921592
O	13.787409	7.889029	9.814112	1.982857
O	13.808965	9.860931	7.866784	1.902063
O	13.794447	9.853925	11.806976	1.951372
O	13.807955	9.862825	0.000000	1.520126
O	13.807955	9.862825	3.945120	1.917063
O	13.787283	0.000079	9.803328	1.983826
O	13.807955	11.835390	1.972576	1.922830
O	13.675509	11.833828	5.902880	1.922650
O	13.828549	11.834001	9.814144	1.983376
O	13.806835	13.814425	11.797088	1.953143
O	13.807955	13.807955	0.000000	1.520160
O	13.807955	13.807955	3.945120	1.917060
O	13.809281	13.804041	7.862592	1.904787
O	13.807955	1.972565	3.945120	1.917001
O	13.807955	1.972565	0.000000	1.520128
O	13.807955	3.945130	1.972576	1.922842
O	13.807955	5.917695	0.000000	1.519983
O	13.807955	0.000000	1.972576	1.922855
O	13.939091	-0.000331	5.904224	1.921884
Pd	2.005972	1.923772	13.974176	3.111791
Pd	1.996914	3.893654	16.179200	2.953222
Pd	1.949683	5.911130	13.967872	3.081646
Pd	1.941635	7.896620	16.129792	2.968012
Pd	1.932814	9.856907	13.978656	3.073472
Pd	1.986894	-0.013887	16.137120	2.995250
Pd	1.982617	11.839288	16.162464	2.961390
Pd	1.989024	13.817644	13.976288	3.103199
Pd	3.960642	1.942061	16.141568	2.993829
Pd	3.934305	3.938455	14.399456	3.475664
Pd	3.920576	5.911651	16.142752	2.949768
Pd	3.926667	7.884358	14.399040	3.467402
Pd	3.939417	9.848938	16.148352	2.940552
Pd	3.962852	15.749653	14.396064	3.533944
Pd	3.943015	11.809542	14.396960	3.471930
Pd	3.939891	13.790328	16.146528	2.985083
Pd	5.893125	1.943387	13.976256	3.104076
Pd	5.890000	3.943110	16.165824	2.936740
Pd	5.905954	5.894245	13.967456	3.090874
Pd	5.921877	7.868167	16.116800	2.976859
Pd	5.918153	9.853451	13.965536	3.087473
Pd	5.915533	15.752510	16.153920	2.979725
Pd	5.909031	11.828888	16.145600	2.960792
Pd	5.916196	13.791449	13.969472	3.107722
Pd	7.852497	1.945580	16.156384	2.965355
Pd	7.833908	3.904448	14.398944	3.513778
Pd	7.885763	5.898727	16.140128	2.967338
Pd	7.903263	7.858352	14.378432	3.458559
Pd	7.860419	9.846997	16.117760	2.965716
Pd	7.889045	15.758648	14.397760	3.474200
Pd	7.878756	11.833323	14.394496	3.490875
Pd	7.891002	13.796956	16.151616	2.953674
Pd	9.851416	1.993206	13.975520	3.072108
Pd	9.806899	3.899319	16.154112	2.980124
Pd	9.797194	5.852884	13.969280	3.096630
Pd	9.872483	7.898971	16.174240	3.317577
Pd	9.856008	9.854019	13.971456	3.048702
Pd	9.858201	0.007811	16.138080	2.944149
Pd	9.863456	11.838341	16.117952	2.967315
Pd	9.857712	13.797761	13.966368	3.084657
Pd	0.018479	1.933035	16.172320	2.959585
Pd	0.065915	3.869652	14.388640	3.511187
Pd	0.055547	5.860175	16.118464	2.976264
Pd	15.710944	7.888382	14.423808	3.526429
Pd	0.008174	9.901251	16.151776	2.981527
Pd	-0.013555	-0.010399	14.399296	3.473377
Pd	0.026685	11.878155	14.400640	3.515645
Pd	0.005681	13.818607	16.171008	2.935286

211	Pd	11.829646	1.987383	16.131232	2.965088
212	Pd	11.815459	3.995170	14.423520	3.524714
213	Pd	11.834711	5.834342	16.197056	2.998364
214	Pd	11.884341	7.825655	14.364320	3.201473
215	Pd	11.822955	9.837308	16.174048	3.311186
216	Pd	11.834901	0.005255	14.398976	3.467000
217	Pd	11.849592	11.805486	14.375872	3.456821
218	Pd	11.826663	13.788987	16.114432	2.980642
219	Pd	13.805746	1.984148	13.969184	3.079641
220	Pd	13.850341	3.894743	16.114400	2.976169
221	Pd	13.867779	5.839960	13.960672	3.112326
222	Pd	13.873886	7.857799	16.197824	2.997614
223	Pd	13.861293	9.915327	13.968896	3.095789
224	Pd	13.799733	-0.008332	16.150048	2.943738
225	Pd	13.809249	11.844811	16.137760	2.968644
226	Pd	13.813021	13.801974	13.967808	3.094555
227	Sr	0.000000	0.000000	0.000000	1.317477
228	Sr	3.945130	0.000000	0.000000	1.317477
229	Sr	3.945130	3.945130	0.000000	1.317452
230	Sr	3.945130	3.945130	3.945120	1.581732
231	Sr	3.944672	3.945430	7.819936	1.586128
232	Sr	3.945083	3.945035	11.551008	1.823021
233	Sr	3.945130	7.890260	0.000000	1.317497
234	Sr	3.945130	7.890260	3.945120	1.581579
235	Sr	3.943931	7.891491	7.820832	1.586141
236	Sr	3.945525	7.890260	11.552416	1.824561
237	Sr	3.945130	0.000000	3.945120	1.581598
238	Sr	3.945004	0.000694	7.819136	1.586695
239	Sr	3.943441	0.001704	11.545440	1.828572
240	Sr	3.945130	11.835390	0.000000	1.317515
241	Sr	3.945130	11.835390	3.945120	1.581841
242	Sr	3.943773	11.836384	7.820384	1.585673
243	Sr	3.944641	11.837552	11.551648	1.823640
244	Sr	7.890260	0.000000	0.000000	1.317521
245	Sr	7.890260	3.945130	0.000000	1.317493
246	Sr	7.890260	3.945130	3.945120	1.581896
247	Sr	7.890165	3.945098	7.821472	1.584771
248	Sr	7.888603	3.944783	11.551968	1.824626
249	Sr	7.890260	7.890260	0.000000	1.317555
250	Sr	7.890260	7.890260	3.945120	1.581690
251	Sr	7.889676	7.890923	7.819584	1.585734
252	Sr	7.886094	7.895578	11.542976	1.831370
253	Sr	7.890260	0.000000	3.945120	1.581762
254	Sr	7.889692	0.002446	7.820864	1.586177
255	Sr	7.889850	0.002509	11.551904	1.823044
256	Sr	7.890260	11.835390	0.000000	1.317568
257	Sr	7.890260	11.835390	3.945120	1.582026
258	Sr	7.889487	11.836400	7.819552	1.585981
259	Sr	7.890181	11.835674	11.548416	1.825293
260	Sr	0.000000	3.945130	0.000000	1.317428
261	Sr	0.000000	3.945130	3.945120	1.582011
262	Sr	-0.000079	3.945209	7.820608	1.585004
263	Sr	-0.000205	3.945919	11.547584	1.829468
264	Sr	0.000000	7.890260	0.000000	1.317464
265	Sr	0.000000	7.890260	3.945120	1.581734
266	Sr	0.000615	7.891664	7.821984	1.584917
267	Sr	0.000600	7.888445	11.555808	1.811866
268	Sr	0.000000	0.000000	3.945120	1.581518
269	Sr	0.000773	0.001105	7.820864	1.586442
270	Sr	0.001357	0.001278	11.551776	1.822846
271	Sr	0.000000	11.835390	0.000000	1.317501
272	Sr	0.000000	11.835390	3.945120	1.581853
273	Sr	0.000584	11.836005	7.821440	1.584512
274	Sr	0.000805	11.837710	11.552544	1.823835
275	Sr	11.835390	0.000000	0.000000	1.317496
276	Sr	11.835390	3.945130	0.000000	1.317445
277	Sr	11.835390	3.945130	3.945120	1.581881
278	Sr	11.834711	3.945477	7.821952	1.584886
279	Sr	11.838735	3.945477	11.555904	1.811990
280	Sr	11.835390	7.890260	0.000000	1.317510
281	Sr	11.835390	7.890260	3.945120	1.581476
282	Sr	11.834601	7.891570	7.822336	1.584672

```
Sr     11.828683      7.897645     11.560288        1.834738
Sr     11.835390      0.000000      3.945120        1.581641
Sr     11.835106      0.001436      7.820384        1.586036
Sr     11.835264     -0.000300     11.552096        1.824265
Sr     11.835390     11.835390      0.000000        1.317553
Sr     11.835390     11.835390      3.945120        1.581838
Sr     11.834932     11.836700      7.819264        1.585788
Sr     11.831540     11.840676     11.541184        1.832469
Ti      1.972565      1.972565      1.972576        2.637061
Ti      1.972328      1.973891      5.899584        2.610193
Ti      1.972833      1.972896      9.843008        2.646890
Ti      1.971871      5.918784      5.899584        2.609379
Ti      1.972644      5.918610      9.843520        2.646975
Ti      1.972565      5.917695      1.972576        2.637031
Ti      1.972518      9.864024      5.900480        2.609665
Ti      1.971650      9.862699      9.847584        2.646052
Ti      1.972565      9.862825      1.972576        2.637059
Ti      1.972170     13.808413      9.843328        2.646910
Ti      1.972565     13.807955      1.972576        2.637066
Ti      1.972186     13.808697      5.899584        2.610065
Ti      5.917695      1.972565      1.972576        2.637063
Ti      5.917695      1.973954      5.899552        2.610388
Ti      5.917316      1.973322      9.843648        2.646849
Ti      5.917569      5.918879      5.899584        2.609202
Ti      5.916212      5.917632      9.843232        2.647113
Ti      5.917695      5.917695      1.972576        2.637019
Ti      5.917427      9.864087      5.898784        2.610218
Ti      5.915328      9.863961      9.841408        2.647760
Ti      5.917695      9.862825      1.972576        2.637050
Ti      5.917206     13.808870      9.842976        2.646564
Ti      5.917695     13.807955      1.972576        2.637053
Ti      5.917837     13.809359      5.899040        2.609785
Ti      9.862651      1.974127      5.900256        2.609423
Ti      9.863377      1.973891      9.846688        2.646446
Ti      9.862430      5.918452      5.899648        2.608421
Ti      9.863314      5.919021      9.846464        2.646912
Ti      9.862825      5.917695      1.972576        2.637009
Ti      9.862857      9.864040      5.899840        2.610069
Ti      9.861673      9.863788      9.842816        2.647709
Ti      9.862825      9.862825      1.972576        2.637036
Ti      9.861594     13.810685      9.841504        2.647222
Ti      9.862825     13.807955      1.972576        2.637038
Ti      9.862399     13.809202      5.899072        2.609711
Ti      9.862825      1.972565      1.972576        2.637051
Ti     13.807592      1.973954      5.899552        2.609348
Ti     13.807213      1.972802      9.844160        2.647127
Ti     13.807781      5.918910      5.899040        2.608637
Ti     13.806582      5.919241      9.844448        2.646582
Ti     13.807955      5.917695      1.972576        2.636994
Ti     13.808239      9.864135      5.899488        2.608580
Ti     13.806677      9.862541      9.846080        2.646425
Ti     13.807955      9.862825      1.972576        2.637002
Ti     13.808586     13.809912      9.843168        2.646785
Ti     13.807955     13.807955      1.972576        2.637023
Ti     13.808350     13.809154      5.899552        2.609263
Ti     13.807955      1.972565      1.972576        2.637028
C      11.360018      8.351114     17.465312        4.019775
H      10.952028      8.760619     18.414336        0.955592
O      12.279343      7.425066     17.641440        2.477396
```

1.1.3 HCOO

```
      340
jmolscript: load "" {1 1 1} spacegroup "x,y,z" unitcell [{      15.780520       0.000000      0.000000 }, {      0.000000     15.780520      0.000000    }, {      0.000000      0.000000     32.000000 }]
O       0.000000      1.972565      1.972576        1.922841
```

O	0.000000	5.917695	1.972576	1.922797
O	0.000000	9.862825	1.972576	1.922822
O	0.000000	13.807955	1.972576	1.922727
O	1.937469	5.914791	11.790304	1.964041
O	1.949983	9.869532	11.792608	1.957460
O	1.959909	1.970372	11.788512	1.950161
O	1.967768	-0.000694	9.799872	1.982555
O	1.966426	13.806251	11.790688	1.953858
O	1.968762	13.808018	7.858816	1.908685
O	1.969014	11.835390	9.798720	1.983158
O	1.968667	3.944988	9.798784	1.982644
O	1.970119	1.971618	7.858272	1.908688
O	1.969456	7.890765	9.804096	1.983325
O	1.969740	3.946014	5.901152	1.928440
O	1.970040	11.834648	5.901120	1.928419
O	1.972013	7.890165	5.899584	1.928681
O	1.971902	-0.000237	5.899872	1.929042
O	1.972565	0.000000	1.972576	1.922728
O	1.972565	1.972565	3.945120	1.918579
O	1.972565	1.972565	0.000000	1.520022
O	1.972565	3.945130	1.972576	1.922783
O	1.972565	5.917695	3.945120	1.918841
O	1.972565	5.917695	0.000000	1.519888
O	1.972565	7.890260	1.972576	1.922795
O	1.972565	9.862825	0.000000	1.519980
O	1.972565	9.862825	3.945120	1.918449
O	1.972565	11.835390	1.972576	1.922819
O	1.972565	13.807955	0.000000	1.520041
O	1.972565	13.807955	3.945120	1.918681
O	1.975311	5.920125	7.859904	1.909822
O	1.975705	9.860868	7.860800	1.908177
O	3.940096	1.972391	9.801792	1.983325
O	3.940254	13.807608	9.800544	1.982772
O	3.940585	5.917805	9.811744	1.981596
O	3.942179	9.862809	9.813792	1.981814
O	3.943221	1.974364	5.898976	1.929403
O	3.943552	13.806061	5.900576	1.928566
O	3.944593	9.863204	5.901120	1.928945
O	3.945067	5.917095	5.901920	1.928256
O	3.945130	1.972565	1.972576	1.922752
O	3.945130	5.917695	1.972576	1.922714
O	3.945130	9.862825	1.972576	1.922722
O	3.945130	13.807955	1.972576	1.922804
O	5.908258	13.812452	11.792096	1.956557
O	5.911635	1.974474	7.858016	1.909480
O	5.912645	0.000473	9.800672	1.981763
O	5.913134	11.833165	9.812288	1.981796
O	5.913955	13.803663	7.860512	1.907631
O	5.914444	9.861294	7.865344	1.904352
O	5.912551	5.916038	11.802176	1.978003
O	5.914618	1.980802	11.788896	1.951167
O	5.914776	-0.000647	5.902560	1.928572
O	5.914997	7.890150	5.902304	1.927499
O	5.915943	3.946850	9.801984	1.983187
O	5.916685	5.920015	7.865792	1.905844
O	5.917143	7.888808	9.808640	1.982072
O	5.917553	11.834080	5.901856	1.928465
O	5.917506	3.946740	5.902016	1.928093
O	5.917695	1.972565	3.945120	1.917431
O	5.917695	3.945130	1.972576	1.922746
O	5.917695	5.917695	3.945120	1.918963
O	5.917695	5.917695	0.000000	1.519832
O	5.917695	7.890260	1.972576	1.922858
O	5.917695	9.862825	0.000000	1.520015
O	5.917695	9.862825	3.945120	1.917463
O	5.917695	11.835390	1.972576	1.922788
O	5.917695	13.807955	0.000000	1.520044
O	5.917695	13.807955	3.945120	1.917893
O	5.917695	0.000000	1.972576	1.922763
O	5.917695	1.972565	0.000000	1.520038
O	5.923723	9.844741	11.802816	1.961367
O	7.884516	13.807703	9.799808	1.984221

O	7.885084	1.972265	9.795520	1.984536
O	7.885983	5.917995	9.817920	1.976405
O	7.886599	9.864198	9.809472	1.981046
O	7.888524	5.919415	5.903392	1.927440
O	7.888508	13.808634	5.900256	1.928530
O	7.888619	1.971760	5.901088	1.928624
O	7.888587	9.861342	5.903616	1.927686
O	7.890260	1.972565	1.972576	1.922866
O	7.890260	5.917695	1.972576	1.922913
O	7.890260	9.862825	1.972576	1.922929
O	7.890260	13.807955	1.972576	1.922805
O	9.842689	1.969220	11.792096	1.954707
O	9.848465	13.824477	11.791488	1.965812
O	9.848370	9.845435	11.808256	1.959837
O	9.848765	5.934706	11.813696	1.963705
O	9.854351	7.890023	9.809856	1.978974
O	9.855850	3.949059	9.817760	1.980258
O	9.857286	5.917174	7.869376	1.902685
O	9.857318	13.800175	7.860992	1.909200
O	9.858612	11.833765	9.816960	1.981493
O	9.858864	0.001531	9.794144	1.980955
O	9.859132	9.863646	7.866848	1.903627
O	9.859243	3.947339	5.900608	1.929211
O	9.860821	7.890181	5.905248	1.927634
O	9.859432	11.834096	5.901184	1.928720
O	9.861184	0.000000	5.907072	1.927358
O	9.861310	1.983343	7.860128	1.908474
O	9.862825	1.972565	3.945120	1.917012
O	9.862825	5.917695	3.945120	1.917652
O	9.862825	5.917695	0.000000	1.519865
O	9.862825	7.890260	1.972576	1.922981
O	9.862825	9.862825	0.000000	1.519963
O	9.862825	9.862825	3.945120	1.917371
O	9.862825	11.835390	1.972576	1.922687
O	9.862825	13.807955	0.000000	1.519947
O	9.862825	13.807955	3.945120	1.918476
O	9.862825	0.000000	1.972576	1.922883
O	9.862825	1.972565	0.000000	1.520005
O	9.862825	3.945130	1.972576	1.922676
O	11.828036	5.918863	9.815328	1.981113
O	11.828368	9.860947	9.810592	1.983234
O	11.829094	1.973969	9.805536	1.982648
O	11.829109	13.807766	9.800672	1.983476
O	11.830877	5.917884	5.901024	1.928986
O	11.831824	9.862778	5.900736	1.928985
O	11.832597	13.807024	5.900864	1.928458
O	11.832976	1.973654	5.898816	1.929218
O	11.835390	1.972565	1.972576	1.922768
O	11.835390	5.917695	1.972576	1.922734
O	11.835390	9.862825	1.972576	1.922722
O	11.835390	13.807955	1.972576	1.922809
O	13.793800	5.916259	7.859200	1.908802
O	13.796656	13.813320	11.788000	1.949940
O	13.797161	9.863377	7.859328	1.909573
O	13.800601	1.965606	11.789760	1.949834
O	13.800301	1.975090	7.858752	1.908673
O	13.802132	3.945035	9.803584	1.982269
O	13.802621	7.890181	9.799424	1.983307
O	13.802763	0.000110	9.795360	1.983081
O	13.802242	11.835358	9.800704	1.982786
O	13.803773	13.806393	7.858048	1.908863
O	13.806708	9.863141	11.789952	1.956427
O	13.806282	3.945682	5.899232	1.929103
O	13.804894	0.000047	5.901024	1.928455
O	13.806124	11.834917	5.899584	1.928808
O	13.805020	7.890165	5.900672	1.928681
O	13.807955	5.917695	3.945120	1.916911
O	13.807955	7.890260	1.972576	1.922878
O	13.807955	9.862825	0.000000	1.520066
O	13.807955	9.862825	3.945120	1.917616
O	13.807955	11.835390	1.972576	1.922784
O	13.807955	13.807955	0.000000	1.520072

O	13.807955	13.807955	3.945120	1.918421
O	13.807955	0.000000	1.972576	1.922805
O	13.807955	1.972565	3.945120	1.918666
O	13.807955	1.972565	0.000000	1.520073
O	13.807955	3.945130	1.972576	1.922754
O	13.807955	5.917695	0.000000	1.520051
O	13.815056	5.919573	11.791520	1.946998
O	-0.006123	5.917774	9.787040	1.981924
O	-0.005618	1.973291	9.797152	1.982063
O	-0.004987	13.807071	9.800736	1.981820
O	-0.005144	9.862557	9.793248	1.981897
O	-0.002162	1.971965	5.901312	1.928338
O	-0.001957	5.919021	5.905664	1.927801
O	-0.001799	13.808239	5.900256	1.928730
O	-0.001625	9.861705	5.905024	1.927893
Pd	1.896582	7.922626	16.159072	2.950091
Pd	1.899627	9.886590	13.960512	3.116816
Pd	1.900117	3.921096	16.144736	2.973291
Pd	1.911368	1.953597	13.966432	3.110492
Pd	1.920900	5.916732	13.952288	3.121961
Pd	1.921768	11.827910	16.124704	2.959494
Pd	1.948563	-0.000063	16.137728	2.983920
Pd	1.951482	13.803158	13.963616	3.100383
Pd	3.811579	7.918649	14.364416	3.515940
Pd	3.820669	5.920173	16.109216	2.955368
Pd	3.834382	3.922737	14.375936	3.517052
Pd	3.900771	11.822513	14.396352	3.501898
Pd	3.901623	1.967720	16.156512	2.959050
Pd	3.917404	13.812531	16.117728	2.965402
Pd	3.918540	9.854161	16.146624	2.940501
Pd	3.921901	0.014518	14.390784	3.478041
Pd	5.774708	5.910341	13.940064	3.140859
Pd	5.800162	3.953083	16.112832	2.995543
Pd	5.842296	7.837695	16.001408	2.944147
Pd	5.855788	9.863867	13.962112	3.060494
Pd	5.872626	11.838404	16.143776	2.966320
Pd	5.876413	2.009444	13.969728	3.050193
Pd	5.884619	13.801059	13.964288	3.079710
Pd	5.890426	0.015528	16.136224	2.931284
Pd	7.811136	5.772372	16.406368	3.046927
Pd	7.830278	2.051562	16.070048	2.971472
Pd	7.837711	11.784671	14.389792	3.522347
Pd	7.838453	4.164243	14.423456	3.469616
Pd	7.843849	9.847265	16.113632	2.903535
Pd	7.847132	13.797114	16.105824	2.976890
Pd	7.848647	0.020846	14.388288	3.475237
Pd	7.974907	7.684545	14.497440	3.288248
Pd	9.786211	0.028026	16.156576	2.915859
Pd	9.801723	13.794242	13.952608	3.093635
Pd	9.804563	2.016372	13.967008	3.061971
Pd	9.834799	11.855589	16.105760	2.916049
Pd	9.853088	9.888910	13.965248	3.084207
Pd	9.865050	3.929933	16.096512	3.014968
Pd	9.974204	5.849239	13.958848	3.198509
Pd	9.993503	7.951962	16.224000	3.055885
Pd	11.747366	0.025028	14.394176	3.462823
Pd	11.760764	13.833488	16.130976	2.983156
Pd	11.763478	7.913079	14.401120	3.537109
Pd	11.803182	1.952445	16.182592	2.935237
Pd	11.821677	11.880775	14.374784	3.499922
Pd	11.853143	5.878843	16.116960	3.004007
Pd	11.877524	9.929229	16.138656	2.953467
Pd	11.880301	3.866953	14.388320	3.534152
Pd	13.755074	5.913403	13.972000	3.080996
Pd	13.757662	7.887451	16.132480	2.974809
Pd	13.760819	9.882614	13.962688	3.087992
Pd	13.761118	15.761473	16.112288	2.993138
Pd	13.772259	11.906087	16.159104	2.976079
Pd	13.771849	13.844566	13.967328	3.108892
Pd	13.795694	3.914279	16.183872	2.942919
Pd	13.808697	1.918643	13.968416	3.121223
Pd	15.701886	9.886228	16.129856	2.963639

220	Pd	15.716925	3.940569	14.394592	3.472908
221	Pd	15.722164	11.864063	14.391232	3.489722
222	Pd	15.726093	7.893022	14.392928	3.471704
223	Pd	15.742883	1.944649	16.132384	2.995596
224	Pd	15.745740	5.918800	16.144416	2.946087
225	Pd	15.760368	15.752809	14.391776	3.536377
226	Pd	-0.009453	13.816492	16.161696	2.966004
227	Sr	0.000000	0.000000	0.000000	1.317193
228	Sr	0.000000	0.000000	3.945120	1.574668
229	Sr	0.000000	3.945130	0.000000	1.317170
230	Sr	0.000000	3.945130	3.945120	1.574253
231	Sr	0.000000	7.890260	0.000000	1.317207
232	Sr	0.000000	7.890260	3.945120	1.574032
233	Sr	0.000000	11.835390	0.000000	1.317219
234	Sr	0.000000	11.835390	3.945120	1.574415
235	Sr	0.000442	3.947355	11.545120	1.822971
236	Sr	3.940538	11.839493	11.546208	1.821020
237	Sr	3.942716	0.000063	11.545280	1.821754
238	Sr	3.943236	0.000552	7.810688	1.578144
239	Sr	3.943820	7.890449	7.809760	1.577785
240	Sr	3.944167	11.835074	7.811904	1.577081
241	Sr	3.944467	3.944436	7.810016	1.577561
242	Sr	3.945130	0.000000	0.000000	1.317180
243	Sr	3.945130	0.000000	3.945120	1.574641
244	Sr	3.945130	3.945130	0.000000	1.317131
245	Sr	3.945130	3.945130	3.945120	1.574543
246	Sr	3.945130	7.890260	0.000000	1.317211
247	Sr	3.945130	7.890260	3.945120	1.574865
248	Sr	3.945130	11.835390	0.000000	1.317254
249	Sr	3.945130	11.835390	3.945120	1.574589
250	Sr	3.945635	3.941201	11.535904	1.832738
251	Sr	3.945430	7.890781	11.532992	1.839206
252	Sr	7.883411	7.901748	11.606144	1.767623
253	Sr	7.885715	3.932111	11.556256	1.803775
254	Sr	7.887798	11.842665	11.543584	1.827697
255	Sr	7.888272	0.000395	7.810848	1.578123
256	Sr	7.888714	11.835848	7.811808	1.577726
257	Sr	7.889755	-0.001042	11.543616	1.828333
258	Sr	7.889361	7.890875	7.820864	1.577309
259	Sr	7.889471	3.943410	7.813248	1.578597
260	Sr	7.890260	0.000000	0.000000	1.317183
261	Sr	7.890260	0.000000	3.945120	1.574071
262	Sr	7.890260	3.945130	0.000000	1.317135
263	Sr	7.890260	3.945130	3.945120	1.574477
264	Sr	7.890260	7.890260	0.000000	1.317239
265	Sr	7.890260	7.890260	3.945120	1.574344
266	Sr	7.890260	11.835390	0.000000	1.317264
267	Sr	7.890260	11.835390	3.945120	1.574518
268	Sr	11.833386	3.945872	7.812608	1.577224
269	Sr	11.834017	7.889471	7.812576	1.578079
270	Sr	11.833780	11.834159	7.811168	1.577296
271	Sr	11.834522	0.001057	7.811104	1.577757
272	Sr	11.835374	7.890765	11.544032	1.822560
273	Sr	11.835642	-0.001752	11.546432	1.820654
274	Sr	11.835390	0.000000	0.000000	1.317180
275	Sr	11.835390	0.000000	3.945120	1.574250
276	Sr	11.835390	3.945130	0.000000	1.317156
277	Sr	11.835390	3.945130	3.945120	1.574530
278	Sr	11.835390	7.890260	0.000000	1.317221
279	Sr	11.835390	7.890260	3.945120	1.574397
280	Sr	11.835390	11.835390	0.000000	1.317229
281	Sr	11.835390	11.835390	3.945120	1.574631
282	Sr	11.836605	11.835232	11.539104	1.829988
283	Sr	11.836558	3.942937	11.543104	1.826841
284	Sr	-0.002604	0.000899	11.540416	1.827153
285	Sr	-0.002872	3.944499	7.810752	1.578611
286	Sr	-0.002478	11.835863	7.810080	1.578311
287	Sr	-0.002430	7.889865	7.810944	1.578130
288	Sr	-0.002115	0.000110	7.809120	1.578375
289	Sr	-0.001404	11.833544	11.543200	1.824906
290	Sr	-0.000836	7.888351	11.545024	1.826144
291	Ti	1.968999	5.917080	9.836128	2.648574

```
Ti       1.969267       1.972028       9.838368        2.650054
Ti       1.969204       9.863724       9.838464        2.649560
Ti       1.969551      13.808097       9.838016        2.650352
Ti       1.972186      13.807892       5.898336        2.621660
Ti       1.972265       1.972407       5.898432        2.621575
Ti       1.972407       9.862872       5.898560        2.621020
Ti       1.972328       5.917632       5.898240        2.621119
Ti       1.972565       1.972565       1.972576        2.637405
Ti       1.972565       5.917695       1.972576        2.637353
Ti       1.972565       9.862825       1.972576        2.637370
Ti       1.972565      13.807955       1.972576        2.637384
Ti       5.914271      13.808634       9.840224        2.649862
Ti       5.916196       1.972581       9.839584        2.651662
Ti       5.917111      13.807781       5.899008        2.620976
Ti       5.917285       1.972644       5.899424        2.621605
Ti       5.917679       9.862541       5.899392        2.619825
Ti       5.917695       1.972565       1.972576        2.637392
Ti       5.917695       5.917695       1.972576        2.637311
Ti       5.917695       9.862825       1.972576        2.637308
Ti       5.917695      13.807955       1.972576        2.637363
Ti       5.918184       5.917916       5.898016        2.619649
Ti       5.918894       9.860852       9.844064        2.648886
Ti       5.920993       5.919163       9.839936        2.648504
Ti       9.855676       5.920551       9.846848        2.645320
Ti       9.857302       9.859117       9.844960        2.647245
Ti       9.859637       1.972991       9.841632        2.649571
Ti       9.860205      13.809123       9.837792        2.650104
Ti       9.862146       9.862241       5.899520        2.619026
Ti       9.862225       5.917443       5.899360        2.618735
Ti       9.862241      13.807292       5.898528        2.620529
Ti       9.862525       1.973212       5.899872        2.620711
Ti       9.862825       5.917695       1.972576        2.637288
Ti       9.862825       9.862825       1.972576        2.637289
Ti       9.862825      13.807955       1.972576        2.637350
Ti       9.862825       1.972565       1.972576        2.637380
Ti      13.805272      13.808286       9.838592        2.650699
Ti      13.805493       1.972139       9.839168        2.650549
Ti      13.805777       5.917711       9.841568        2.649013
Ti      13.805888       9.861926       9.839392        2.650085
Ti      13.806677       5.917521       5.899968        2.620809
Ti      13.806740       9.862557       5.899168        2.621231
Ti      13.806882       1.972865       5.898336        2.621933
Ti      13.807245      13.807734       5.898592        2.621840
Ti      13.807955       5.917695       1.972576        2.637378
Ti      13.807955       9.862825       1.972576        2.637385
Ti      13.807955      13.807955       1.972576        2.637404
Ti      13.807955       1.972565       1.972576        2.637425
C        8.098011       7.744338      18.632544        4.167494
H        7.790527       8.125200      19.627616        0.935806
O        7.698258       6.581771      18.343712        2.460289
O        8.820127       8.548339      17.948608        2.520281
```

1.1.4 CH_3O

```
       341
jmolscript: load "" {1 1 1} spacegroup "x,y,z" unitcell [{      15.780520
       0.000000      0.000000 }, {      0.000000     15.780520      0.000000
   }, {      0.000000      0.000000     32.000000 }]
O        1.972565       1.972565       3.945120        1.917745
O        1.976068       1.973054       7.858816        1.908071
O        1.971050       1.966994      11.790176        1.949766
O        1.972565       1.972565       0.000000        1.520465
O        1.972565       3.945130       1.972576        1.922742
O        1.981150       3.945540       5.901024        1.929138
O        1.972723       3.944294       9.803808        1.982512
O        1.972565       5.917695       3.945120        1.918078
O        1.980439       5.918137       7.861824        1.908244
```

O	1.953676	5.917790	11.793408	1.963608
O	1.972565	5.917695	0.000000	1.520380
O	1.972565	7.890260	1.972576	1.922781
O	1.965195	7.889787	5.901120	1.928926
O	1.975595	7.890118	9.805312	1.982633
O	1.975279	9.860695	7.862560	1.906603
O	1.967594	9.868616	11.796160	1.960150
O	1.972565	9.862825	0.000000	1.520388
O	1.972565	9.862825	3.945120	1.918258
O	1.972565	0.000000	1.972576	1.922739
O	1.965069	-0.000505	5.900832	1.929413
O	1.975216	-0.001562	9.799296	1.982214
O	1.972565	11.835390	1.972576	1.922781
O	1.981465	11.834033	5.901120	1.929087
O	1.971602	11.834033	9.800064	1.983007
O	1.974474	13.804894	11.790208	1.949558
O	1.972565	13.807955	0.000000	1.520451
O	1.972565	13.807955	3.945120	1.917656
O	1.973575	13.806361	7.858368	1.909090
O	3.945130	1.972565	1.972576	1.922717
O	3.945714	1.964312	5.899040	1.929963
O	3.946077	1.973464	9.803456	1.982652
O	3.946534	5.925696	5.900736	1.929689
O	3.946961	5.914365	9.815872	1.981905
O	3.945130	5.917695	1.972576	1.922724
O	3.946029	9.853609	5.902336	1.928521
O	3.947197	9.863204	9.811360	1.982334
O	3.945130	9.862825	1.972576	1.922745
O	3.945714	13.804673	9.800256	1.983237
O	3.945130	13.807955	1.972576	1.922769
O	3.946077	13.814914	5.900928	1.929118
O	5.917695	1.972565	3.945120	1.916618
O	5.916196	1.974080	7.860032	1.906062
O	5.918689	1.978562	11.794400	1.942797
O	5.917695	3.945130	1.972576	1.922694
O	5.910578	3.945509	5.901376	1.929525
O	5.922003	3.945603	9.805472	1.982947
O	5.917695	5.917695	3.945120	1.916978
O	5.917521	5.916922	7.864576	1.904708
O	5.931535	5.917711	11.803264	1.953839
O	5.917695	5.917695	0.000000	1.520476
O	5.917695	7.890260	1.972576	1.922811
O	5.926437	7.890102	5.903040	1.928546
O	5.917679	7.887546	9.805216	1.983582
O	5.917821	9.861626	7.864320	1.906459
O	5.934359	9.845687	11.799072	1.970233
O	5.917695	9.862825	0.000000	1.520374
O	5.917695	9.862825	3.945120	1.918847
O	5.926374	-0.001152	5.903072	1.928835
O	5.916322	-0.000600	9.803584	1.981219
O	5.917695	11.835390	1.972576	1.922745
O	5.910073	11.834096	5.902272	1.928568
O	5.919273	11.832486	9.810752	1.982469
O	5.911793	13.811742	11.795872	1.959906
O	5.917695	13.807955	0.000000	1.520385
O	5.917695	13.807955	3.945120	1.918312
O	5.918910	13.804562	7.862304	1.907001
O	5.917695	1.972565	0.000000	1.520522
O	5.917695	0.000000	1.972576	1.922768
O	7.890260	1.972565	1.972576	1.922818
O	7.890655	1.979761	5.901376	1.928891
O	7.889771	1.969093	9.800160	1.983094
O	7.890213	5.909394	5.903456	1.928706
O	7.892154	5.918926	9.810176	1.979227
O	7.890260	5.917695	1.972576	1.922879
O	7.890039	9.870368	5.903040	1.928333
O	7.892217	9.859101	9.804640	1.983197
O	7.890260	9.862825	1.972576	1.922815
O	7.889518	13.807008	9.804320	1.982925
O	7.890260	13.807955	1.972576	1.922796
O	7.890323	13.798534	5.901056	1.928936
O	9.862825	1.972565	3.945120	1.918343

O	9.864245	1.976763	7.859968	1.909145
O	9.855692	1.961566	11.792032	1.962904
O	9.870763	3.945461	5.900672	1.929614
O	9.859779	3.944088	9.808160	1.982799
O	9.862825	5.917695	3.945120	1.916496
O	9.863346	5.916401	7.864320	1.903875
O	9.866297	5.913561	11.802272	1.954224
O	9.862825	5.917695	0.000000	1.520467
O	9.862825	7.890260	1.972576	1.922865
O	9.854019	7.889976	5.903360	1.928850
O	9.864308	7.887562	9.809408	1.979770
O	9.862983	9.862494	7.864224	1.905179
O	9.862225	9.847486	11.802464	1.954449
O	9.862825	9.862825	0.000000	1.520469
O	9.862825	9.862825	3.945120	1.917156
O	9.864861	-0.000978	9.792896	1.982403
O	9.862825	11.835390	1.972576	1.922709
O	9.870842	11.833717	5.900640	1.929484
O	9.862525	11.832802	9.815200	1.981983
O	9.861515	13.825440	11.792736	1.964384
O	9.862825	13.807955	0.000000	1.520371
O	9.862825	13.807955	3.945120	1.918310
O	9.861815	13.799875	7.861440	1.908555
O	9.862825	1.972565	0.000000	1.520389
O	9.862825	3.945130	1.972576	1.922653
O	9.862825	0.000000	1.972576	1.922824
O	9.854351	-0.001089	5.904832	1.927724
O	0.000000	1.972565	1.972576	1.922825
O	0.000174	1.980266	5.901824	1.928823
O	0.000994	1.970545	9.796256	1.982460
O	0.000789	5.909205	5.905088	1.927684
O	0.000284	5.917821	9.792800	1.982231
O	0.000000	5.917695	1.972576	1.922809
O	0.000931	9.870210	5.902784	1.928912
O	-0.000268	9.860032	9.804352	1.981687
O	0.000000	9.862825	1.972576	1.922724
O	0.000710	13.808018	9.799264	1.981826
O	0.000000	13.807955	1.972576	1.922722
O	0.000126	13.798897	5.900704	1.929509
O	11.835390	1.972565	1.972576	1.922714
O	11.835090	1.963807	5.899584	1.929317
O	11.835280	1.973812	9.805440	1.982004
O	11.834790	5.925743	5.900864	1.929527
O	11.835800	5.916496	9.808384	1.982617
O	11.835390	5.917695	1.972576	1.922673
O	11.834743	9.853104	5.901696	1.929586
O	11.834222	9.860994	9.804672	1.982990
O	11.835390	9.862825	1.972576	1.922714
O	11.835390	13.804010	9.803040	1.982466
O	11.835390	13.807955	1.972576	1.922741
O	11.834759	13.815467	5.900832	1.929271
O	13.805619	1.974680	7.859104	1.908238
O	13.816555	1.962907	11.789536	1.950618
O	13.800096	3.945240	5.899616	1.929357
O	13.809154	3.944451	9.805440	1.982030
O	13.807955	5.917695	3.945120	1.918103
O	13.803237	5.916038	7.860064	1.909344
O	13.818922	5.923502	11.792288	1.962453
O	13.807955	7.890260	1.972576	1.922816
O	13.816839	7.889439	5.901184	1.929143
O	13.807308	7.889771	9.800672	1.983256
O	13.806645	9.863330	7.860032	1.906076
O	13.799796	9.861089	11.794464	1.942479
O	13.807955	9.862825	0.000000	1.520530
O	13.807955	9.862825	3.945120	1.916518
O	13.805967	-0.001720	9.795872	1.982422
O	13.807955	11.835390	1.972576	1.922734
O	13.799591	11.834522	5.899136	1.929906
O	13.809628	11.833717	9.803168	1.982675
O	13.811632	13.808397	11.789696	1.950194
O	13.807955	13.807955	0.000000	1.520475
O	13.807955	13.807955	3.945120	1.917740

156	O	13.807119	13.803884	7.858592	1.908575
157	O	13.807955	1.972565	3.945120	1.918194
158	O	13.807955	1.972565	0.000000	1.520407
159	O	13.807955	3.945130	1.972576	1.922706
160	O	13.807955	5.917695	0.000000	1.520403
161	O	13.807955	0.000000	1.972576	1.922813
162	O	13.816271	-0.000473	5.901984	1.928920
163	Pd	1.952035	1.963176	13.969344	3.103553
164	Pd	1.942598	3.946487	16.144832	2.973774
165	Pd	1.959183	5.921703	13.955968	3.106630
166	Pd	1.960714	7.918539	16.143712	2.958047
167	Pd	1.965101	9.867086	13.960704	3.106513
168	Pd	1.983012	15.755161	16.157344	2.969213
169	Pd	1.972265	11.822324	16.125376	2.963183
170	Pd	1.987651	13.788403	13.968704	3.098999
171	Pd	3.917293	1.970435	16.157600	2.944752
172	Pd	3.898562	3.928276	14.393248	3.506907
173	Pd	3.876358	5.921877	16.118912	3.005502
174	Pd	3.909671	7.903453	14.369248	3.499590
175	Pd	3.962347	9.859527	16.145024	2.961319
176	Pd	3.943962	0.010431	14.397728	3.482455
177	Pd	3.950969	11.830672	14.394464	3.506355
178	Pd	3.960611	13.804941	16.128800	2.960556
179	Pd	5.900510	1.995289	13.979808	3.062611
180	Pd	5.865556	3.971752	16.150368	2.975365
181	Pd	5.860001	5.893677	13.967456	3.101243
182	Pd	5.894198	7.853917	16.055456	2.957078
183	Pd	5.900289	9.876680	13.949504	3.060597
184	Pd	5.910941	0.022329	16.140736	2.962690
185	Pd	5.921593	11.812066	16.146080	2.958718
186	Pd	5.914065	13.813699	13.960544	3.107104
187	Pd	7.865201	2.008213	16.109152	2.972054
188	Pd	7.871765	4.037762	14.417888	3.507612
189	Pd	7.959063	5.786496	16.275520	3.068185
190	Pd	7.994538	7.778487	14.443136	3.274938
191	Pd	7.928228	9.880010	16.054016	2.956640
192	Pd	7.885573	0.009374	14.395744	3.477075
193	Pd	7.874953	11.865199	14.368256	3.499448
194	Pd	7.860766	13.816256	16.140640	2.961046
195	Pd	9.859464	1.985631	13.957184	3.078596
196	Pd	9.924874	3.864239	16.088608	2.991289
197	Pd	9.944189	5.833143	13.967168	3.127691
198	Pd	9.988659	7.814498	16.274752	3.069867
199	Pd	9.883545	9.915043	13.966368	3.101629
200	Pd	9.851400	0.023560	16.142560	2.930936
201	Pd	9.855313	11.901353	16.118688	3.007492
202	Pd	9.855566	13.817865	13.954912	3.107962
203	Pd	0.021256	1.945423	16.124544	3.004144
204	Pd	-0.015481	3.948886	14.393536	3.477530
205	Pd	15.754293	5.927116	16.140576	2.932363
206	Pd	-0.009879	7.896004	14.396256	3.477438
207	Pd	15.756060	9.869642	16.142464	2.961563
208	Pd	0.035238	15.740043	14.392672	3.526873
209	Pd	15.762073	11.833165	14.397504	3.482695
210	Pd	0.015938	13.792664	16.156992	2.970906
211	Pd	11.875709	1.936301	16.186464	2.958096
212	Pd	11.931730	3.849611	14.377344	3.500921
213	Pd	11.917038	5.853563	16.091328	2.989003
214	Pd	11.739508	7.903121	14.418176	3.509071
215	Pd	11.802282	9.909046	16.150048	2.975358
216	Pd	11.826300	0.012419	14.392256	3.477726
217	Pd	11.847715	11.876293	14.392896	3.506847
218	Pd	11.830372	13.834498	16.142976	2.976695
219	Pd	13.866232	1.911621	13.966656	3.125154
220	Pd	13.842798	3.900534	16.188800	2.955770
221	Pd	13.795157	5.920551	13.957472	3.078333
222	Pd	13.770887	7.914641	16.108864	2.972753
223	Pd	13.780986	9.874834	13.980064	3.063474
224	Pd	13.829353	15.758096	16.123744	3.003775
225	Pd	13.802858	11.858224	16.159136	2.945050
226	Pd	13.810953	13.825045	13.968384	3.104442
227	Sr	0.000000	0.000000	0.000000	1.317653

228	Sr	3.945130	0.000000	0.000000	1.317689
229	Sr	3.945130	3.945130	0.000000	1.317722
230	Sr	3.945130	3.945130	3.945120	1.574155
231	Sr	3.946377	3.944025	7.812448	1.577144
232	Sr	3.947466	3.940554	11.546464	1.824106
233	Sr	3.945130	7.890260	0.000000	1.317777
234	Sr	3.945130	7.890260	3.945120	1.574314
235	Sr	3.944704	7.888540	7.810496	1.577558
236	Sr	3.946866	7.889645	11.536832	1.836176
237	Sr	3.945130	0.000000	3.945120	1.574089
238	Sr	3.944783	0.000568	7.811360	1.578150
239	Sr	3.943804	-0.001783	11.547808	1.821019
240	Sr	3.945130	11.835390	0.000000	1.317740
241	Sr	3.945130	11.835390	3.945120	1.574229
242	Sr	3.944672	11.835090	7.811040	1.577348
243	Sr	3.942479	11.837299	11.544832	1.823388
244	Sr	7.890260	0.000000	0.000000	1.317696
245	Sr	7.890260	3.945130	0.000000	1.317736
246	Sr	7.890260	3.945130	3.945120	1.574001
247	Sr	7.890528	3.944152	7.813152	1.577882
248	Sr	7.890071	3.939165	11.550528	1.811130
249	Sr	7.890260	7.890260	0.000000	1.317829
250	Sr	7.890260	7.890260	3.945120	1.573994
251	Sr	7.891254	7.888666	7.817472	1.577651
252	Sr	7.884200	7.896336	11.584256	1.793179
253	Sr	7.890260	0.000000	3.945120	1.573937
254	Sr	7.889771	-0.000205	7.811264	1.578111
255	Sr	7.892248	-0.001894	11.546720	1.824381
256	Sr	7.890260	11.835390	0.000000	1.317775
257	Sr	7.890260	11.835390	3.945120	1.574264
258	Sr	7.891365	11.835358	7.810336	1.577777
259	Sr	7.890307	11.833291	11.535968	1.836772
260	Sr	0.000000	3.945130	0.000000	1.317651
261	Sr	0.000000	3.945130	3.945120	1.574134
262	Sr	-0.000268	3.943741	7.811072	1.578186
263	Sr	0.000584	3.944499	11.545088	1.823863
264	Sr	0.000000	7.890260	0.000000	1.317699
265	Sr	0.000000	7.890260	3.945120	1.573982
266	Sr	-0.000174	7.890086	7.811648	1.578060
267	Sr	0.001089	7.887341	11.547040	1.824308
268	Sr	0.000000	0.000000	3.945120	1.574102
269	Sr	-0.000189	-0.000252	7.810112	1.578714
270	Sr	-0.001105	0.000742	11.541152	1.826618
271	Sr	0.000000	11.835390	0.000000	1.317695
272	Sr	0.000000	11.835390	3.945120	1.574031
273	Sr	-0.001057	11.835106	7.811456	1.577987
274	Sr	0.001404	11.836321	11.547776	1.820881
275	Sr	11.835390	0.000000	0.000000	1.317644
276	Sr	11.835390	3.945130	0.000000	1.317648
277	Sr	11.835390	3.945130	3.945120	1.574222
278	Sr	11.834569	3.945540	7.810784	1.577480
279	Sr	11.833039	3.946392	11.539328	1.830255
280	Sr	11.835390	7.890260	0.000000	1.317744
281	Sr	11.835390	7.890260	3.945120	1.573907
282	Sr	11.836069	7.889361	7.813056	1.578038
283	Sr	11.840377	7.890086	11.550496	1.811012
284	Sr	11.835390	0.000000	3.945120	1.574182
285	Sr	11.836321	-0.000142	7.810656	1.578344
286	Sr	11.835437	-0.001247	11.544256	1.824120
287	Sr	11.835390	11.835390	0.000000	1.317732
288	Sr	11.835390	11.835390	3.945120	1.574160
289	Sr	11.836305	11.833591	7.812128	1.577262
290	Sr	11.839209	11.832565	11.545920	1.823990
291	Ti	1.972565	1.972565	1.972576	2.637362
292	Ti	1.972881	1.972297	5.899168	2.621129
293	Ti	1.973070	1.971476	9.840160	2.649694
294	Ti	1.972960	5.917001	5.898816	2.620326
295	Ti	1.972297	5.915880	9.839584	2.649847
296	Ti	1.972565	5.917695	1.972576	2.637319
297	Ti	1.972391	9.862620	5.898720	2.620116
298	Ti	1.971902	9.863030	9.840128	2.648537
299	Ti	1.972565	9.862825	1.972576	2.637314

```
Ti       1.972423     13.807560      9.838880        2.649748
Ti       1.972565     13.807955      1.972576        2.637348
Ti       1.972344     13.807813      5.899200        2.621077
Ti       5.917695      1.972565      1.972576        2.637340
Ti       5.917364      1.972723      5.900320        2.620750
Ti       5.918121      1.971618      9.843680        2.648321
Ti       5.917979      5.917064      5.899872        2.619982
Ti       5.921845      5.917016      9.843968        2.648642
Ti       5.917695      5.917695      1.972576        2.637307
Ti       5.918200      9.861910      5.898112        2.620054
Ti       5.920393      9.859401      9.838624        2.650766
Ti       5.917695      9.862825      1.972576        2.637279
Ti       5.916764     13.807860      9.839648        2.648662
Ti       5.917695     13.807955      1.972576        2.637321
Ti       5.917427     13.807829      5.898688        2.620374
Ti       9.862509      1.972928      5.898624        2.620638
Ti       9.862115      1.971161      9.838112        2.650502
Ti       9.862557      5.917585      5.900384        2.619682
Ti       9.860632      5.919241      9.846016        2.647770
Ti       9.862825      5.917695      1.972576        2.637297
Ti       9.863141      9.862178      5.899712        2.620075
Ti       9.862983      9.858012      9.843200        2.648800
Ti       9.862825      9.862825      1.972576        2.637301
Ti       9.863930     13.807703      9.838880        2.649707
Ti       9.862825     13.807955      1.972576        2.637315
Ti       9.863062     13.807324      5.898656        2.620511
Ti       9.862825      1.972565      1.972576        2.637350
Ti      13.807608      1.972675      5.898752        2.621100
Ti      13.808507      1.971350      9.839392        2.650316
Ti      13.807355      5.917600      5.898848        2.620830
Ti      13.808712      5.917553      9.838336        2.650223
Ti      13.807955      5.917695      1.972576        2.637341
Ti      13.807687      9.862651      5.900416        2.620595
Ti      13.808286      9.861610      9.843776        2.648475
Ti      13.807955      9.862825      1.972576        2.637338
Ti      13.808428     13.806771      9.839616        2.649883
Ti      13.807955     13.807955      1.972576        2.637368
Ti      13.807923     13.807276      5.899200        2.621569
Ti      13.807955      1.972565      1.972576        2.637368
C        7.819863      7.954802     18.380352        3.912478
H        7.205054      8.562368     17.612032        1.167005
H        7.091229      7.406840     18.999584        0.967293
H        8.367652      8.690033     18.991840        0.967926
O        8.711115      7.064308     17.761120        2.474832
```

1.1.5 COOH

```
       340
jmolscript: load "" {1 1 1} spacegroup "x,y,z" unitcell [{     15.780520      0.000000      0.000000 }, {      0.000000     15.780520      0.000000 }, {      0.000000      0.000000     32.000000 }]
O        1.972565      1.972565      3.945120        1.918833
O        1.965716      1.975043      7.859072        1.909371
O        1.966931      1.971744     11.791648        1.953118
O        1.972565      1.972565      0.000000        1.519315
O        1.972565      3.945130      1.972576        1.922834
O        1.971918      3.945872      5.900672        1.928725
O        1.970372      3.945982      9.803648        1.982619
O        1.972565      5.917695      3.945120        1.918422
O        1.975832      5.918942      7.862496        1.906683
O        1.957400      5.919888     11.796000        1.963125
O        1.972565      5.917695      0.000000        1.519239
O        1.972565      7.890260      1.972576        1.922906
O        1.971082      7.889723      5.900544        1.928817
O        1.970924      7.890512      9.805536        1.981953
O        1.969945      9.861421      7.859840        1.907352
O        1.966000      9.868853     11.792896        1.951105
```

O	1.972565	9.862825	0.000000	1.519370
O	1.972565	9.862825	3.945120	1.917910
O	1.972565	0.000000	1.972576	1.922826
O	1.971239	0.000379	5.900704	1.928692
O	1.968020	0.000316	9.798688	1.983127
O	1.972565	11.835390	1.972576	1.922820
O	1.971744	11.834885	5.900576	1.928838
O	1.968620	11.835548	9.799456	1.982477
O	1.961724	13.810464	11.789184	1.954073
O	1.972565	13.807955	0.000000	1.519396
O	1.972565	13.807955	3.945120	1.919138
O	1.968541	13.808050	7.858240	1.909493
O	3.945130	1.972565	1.972576	1.922886
O	3.943804	1.973007	5.902304	1.927694
O	3.939670	1.971902	9.794720	1.982890
O	3.945351	5.917932	5.901792	1.928533
O	3.943347	5.917742	9.811840	1.981412
O	3.945130	5.917695	1.972576	1.922763
O	3.944373	9.861594	5.901120	1.928365
O	3.941074	9.862336	9.801216	1.982339
O	3.945130	9.862825	1.972576	1.922816
O	3.940112	13.807529	9.799136	1.982540
O	3.945130	13.807955	1.972576	1.922815
O	3.943883	13.807608	5.900672	1.928781
O	5.917695	1.972565	3.945120	1.919010
O	5.917632	1.973827	7.859520	1.908890
O	5.896723	1.970798	11.788416	1.960588
O	5.917695	3.945130	1.972576	1.922831
O	5.917158	3.945651	5.901440	1.928280
O	5.913923	3.945682	9.804320	1.983227
O	5.917695	5.917695	3.945120	1.918436
O	5.916511	5.918121	7.864128	1.904950
O	5.919478	5.923455	11.799872	1.949674
O	5.917695	5.917695	0.000000	1.519018
O	5.917695	7.890260	1.972576	1.922867
O	5.917663	7.889613	5.901376	1.928320
O	5.915186	7.889881	9.803264	1.983339
O	5.915407	9.861783	7.860448	1.908642
O	5.907848	9.865034	11.792448	1.965699
O	5.917695	9.862825	0.000000	1.519214
O	5.917695	9.862825	3.945120	1.919474
O	5.916180	0.000095	5.901408	1.928538
O	5.915091	-0.001136	9.800064	1.982761
O	5.917695	11.835390	1.972576	1.922814
O	5.916732	11.834412	5.901024	1.928493
O	5.913876	11.834680	9.800800	1.982804
O	5.903729	13.806093	11.792576	1.951751
O	5.917695	13.807955	0.000000	1.519349
O	5.917695	13.807955	3.945120	1.918027
O	5.915801	13.806929	7.859744	1.907277
O	5.917695	1.972565	0.000000	1.519270
O	5.917695	0.000000	1.972576	1.922825
O	7.890260	1.972565	1.972576	1.922823
O	7.888903	1.972407	5.899328	1.929363
O	7.886157	1.972139	9.807520	1.983154
O	7.890623	5.917269	5.906208	1.927481
O	7.891333	5.914965	9.810336	1.981724
O	7.890260	5.917695	1.972576	1.923029
O	7.889345	9.861705	5.901472	1.928283
O	7.886346	9.861010	9.803552	1.983284
O	7.890260	9.862825	1.972576	1.922878
O	7.885479	13.805414	9.805600	1.981952
O	7.890260	13.807955	1.972576	1.922895
O	7.889203	13.807324	5.900192	1.928895
O	9.862825	1.972565	3.945120	1.915072
O	9.857570	1.976337	7.860896	1.903998
O	9.858896	1.955917	11.799904	1.918054
O	9.861263	3.946282	5.901280	1.928979
O	9.862699	3.941785	9.814144	1.981554
O	9.862825	5.917695	3.945120	1.919924
O	9.865713	5.912377	7.870304	1.904302
O	9.869390	5.902640	11.814304	1.973146

O	9.862825	5.917695	0.000000	1.518781
O	9.862825	7.890260	1.972576	1.923009
O	9.860821	7.888398	5.905824	1.927445
O	9.861973	7.885037	9.811424	1.982050
O	9.859164	9.860269	7.864224	1.904894
O	9.850343	9.854114	11.800544	1.948708
O	9.862825	9.862825	0.000000	1.519027
O	9.862825	9.862825	3.945120	1.918208
O	9.858154	-0.001925	9.797664	1.982250
O	9.862825	11.835390	1.972576	1.922776
O	9.861389	11.833417	5.901824	1.928311
O	9.858596	11.832723	9.811616	1.981382
O	9.853499	13.815451	11.796064	1.962564
O	9.862825	13.807955	0.000000	1.519225
O	9.862825	13.807955	3.945120	1.918453
O	9.857507	13.801438	7.862368	1.907293
O	9.862825	1.972565	0.000000	1.519285
O	9.862825	3.945130	1.972576	1.922789
O	9.862825	0.000000	1.972576	1.922919
O	9.861294	-0.000994	5.904608	1.928694
O	0.000000	1.972565	1.972576	1.922792
O	-0.001846	1.973086	5.900000	1.928499
O	-0.004292	1.972628	9.803008	1.982583
O	-0.000552	5.916969	5.904864	1.928664
O	-0.002446	5.918074	9.797728	1.982075
O	0.000000	5.917695	1.972576	1.922906
O	-0.001594	9.863093	5.901376	1.928487
O	-0.003424	9.861215	9.800736	1.983029
O	0.000000	9.862825	1.972576	1.922791
O	-0.005097	13.807923	9.799008	1.983229
O	0.000000	13.807955	1.972576	1.922786
O	-0.001846	13.807292	5.900576	1.928625
O	11.835390	1.972565	1.972576	1.922925
O	11.833922	1.972313	5.901600	1.928528
O	11.831224	1.971192	9.802688	1.982212
O	11.832613	5.918153	5.900896	1.928920
O	11.834491	5.914223	9.815008	1.981259
O	11.835390	5.917695	1.972576	1.922798
O	11.833133	9.861136	5.901568	1.928345
O	11.830482	9.862541	9.804512	1.983567
O	11.835390	9.862825	1.972576	1.922866
O	11.829930	13.805998	9.802848	1.982572
O	11.835390	13.807955	1.972576	1.922870
O	11.832912	13.807482	5.900960	1.928544
O	13.805099	1.972186	7.860608	1.908432
O	13.797350	1.975185	11.791296	1.973069
O	13.806014	3.944909	5.901632	1.928581
O	13.805004	3.944909	9.803136	1.981871
O	13.807955	5.917695	3.945120	1.915116
O	13.800459	5.919636	7.861120	1.904027
O	13.817202	5.914428	11.800224	1.918617
O	13.807955	7.890260	1.972576	1.922841
O	13.807071	7.890102	5.899328	1.929186
O	13.804562	7.890039	9.807808	1.983020
O	13.803584	9.859511	7.859648	1.908701
O	13.801311	9.875686	11.789152	1.959698
O	13.807955	9.862825	0.000000	1.519287
O	13.807955	9.862825	3.945120	1.918872
O	13.803931	-0.000252	9.803168	1.982597
O	13.807955	11.835390	1.972576	1.922897
O	13.805399	11.834980	5.902304	1.927790
O	13.804420	11.836053	9.795200	1.982874
O	13.800096	13.805288	11.791904	1.953112
O	13.807955	13.807955	0.000000	1.519304
O	13.807955	13.807955	3.945120	1.918882
O	13.802479	13.811190	7.859136	1.908785
O	13.807955	1.972565	3.945120	1.919604
O	13.807955	1.972565	0.000000	1.519272
O	13.807955	3.945130	1.972576	1.922896
O	13.807955	5.917695	0.000000	1.519290
O	13.807955	0.000000	1.972576	1.922793
O	13.806330	0.000363	5.900064	1.928493

163	Pd	1.935355	1.974285	13.964544	3.102850
164	Pd	1.928190	3.955561	16.140256	2.960473
165	Pd	1.938337	5.920977	13.958144	3.112688
166	Pd	1.928679	7.904652	16.148896	2.954730
167	Pd	1.933161	9.880278	13.968096	3.099036
168	Pd	1.929232	-0.001941	16.143008	2.964675
169	Pd	1.930179	11.864000	16.139072	2.970891
170	Pd	1.936948	13.813273	13.962816	3.094656
171	Pd	3.904053	1.969393	16.141792	2.932599
172	Pd	3.889946	3.943000	14.391808	3.494990
173	Pd	3.893496	5.922129	16.136192	2.964422
174	Pd	3.879089	7.909039	14.398112	3.486665
175	Pd	3.912669	9.877596	16.148608	2.951394
176	Pd	3.895074	0.007543	14.388800	3.510443
177	Pd	3.908866	11.841513	14.396544	3.498588
178	Pd	3.892928	13.818560	16.140672	2.970191
179	Pd	5.854920	1.951798	13.955616	3.096567
180	Pd	5.877849	3.966371	16.122464	2.953348
181	Pd	5.843590	5.914176	13.964608	3.065692
182	Pd	5.871979	7.896541	16.120064	2.996523
183	Pd	5.861753	9.890788	13.952160	3.101490
184	Pd	5.863852	-0.010242	16.124640	3.002803
185	Pd	5.873604	11.836432	16.149280	2.950327
186	Pd	5.873336	13.819001	13.967488	3.100057
187	Pd	7.786661	1.964264	16.128928	3.020719
188	Pd	7.842745	3.924173	14.364256	3.438855
189	Pd	7.868578	5.917585	16.148448	3.290449
190	Pd	7.817307	7.932946	14.365024	3.424768
191	Pd	7.860561	9.878069	16.120672	2.994735
192	Pd	7.864333	-0.004734	14.394528	3.477486
193	Pd	7.841009	11.869681	14.397824	3.486935
194	Pd	7.845080	13.821290	16.146944	2.958831
195	Pd	9.824021	2.000670	14.002432	3.097338
196	Pd	9.767984	3.819706	16.227872	2.881515
197	Pd	9.782045	5.966015	13.945728	3.039038
198	Pd	9.828313	7.873690	16.154112	3.286307
199	Pd	9.838397	9.909788	13.966368	3.066972
200	Pd	9.824857	0.010021	16.149792	2.911146
201	Pd	9.827855	11.864710	16.136512	2.964188
202	Pd	9.832605	13.814883	13.957760	3.113686
203	Pd	15.749401	1.978972	16.133344	2.970706
204	Pd	15.747160	3.956823	14.386976	3.477971
205	Pd	15.740248	5.925522	16.147776	2.914224
206	Pd	15.754892	7.888856	14.395776	3.478983
207	Pd	-0.017564	9.886985	16.128544	2.998503
208	Pd	15.736050	0.014281	14.389024	3.495179
209	Pd	15.737108	11.852733	14.390176	3.509115
210	Pd	15.749275	13.821495	16.142304	2.965711
211	Pd	11.834333	1.977804	16.107936	3.008753
212	Pd	11.739429	4.016269	14.438784	3.428922
213	Pd	11.935139	5.980786	16.233856	2.871878
214	Pd	11.819089	7.900202	14.367136	3.436530
215	Pd	11.778896	9.868459	16.122848	2.951299
216	Pd	11.790273	0.002730	14.386080	3.478150
217	Pd	11.806890	11.857735	14.393792	3.495585
218	Pd	11.800389	13.822268	16.141184	2.961730
219	Pd	13.781665	1.970971	13.946400	3.081008
220	Pd	13.774784	3.912922	16.110496	3.006568
221	Pd	13.756195	5.931014	14.002880	3.097596
222	Pd	13.783700	7.968153	16.128576	3.022972
223	Pd	13.796672	9.894607	13.958016	3.095538
224	Pd	13.769719	0.007196	16.131072	2.972011
225	Pd	13.777688	11.845900	16.145792	2.930230
226	Pd	13.777388	13.818575	13.965248	3.102543
227	Sr	0.000000	0.000000	0.000000	1.316423
228	Sr	3.945130	0.000000	0.000000	1.316427
229	Sr	3.945130	3.945130	0.000000	1.316299
230	Sr	3.945130	3.945130	3.945120	1.574725
231	Sr	3.943946	3.943915	7.811072	1.577482
232	Sr	3.944751	3.939512	11.544576	1.825142
233	Sr	3.945130	7.890260	0.000000	1.316335
234	Sr	3.945130	7.890260	3.945120	1.574810

Sr	3.943394	7.889865	7.811104	1.577615
Sr	3.947308	7.892785	11.547136	1.821897
Sr	3.945130	0.000000	3.945120	1.574695
Sr	3.943883	-0.000726	7.809408	1.578293
Sr	3.942984	-0.000789	11.540160	1.828391
Sr	3.945130	11.835390	0.000000	1.316459
Sr	3.945130	11.835390	3.945120	1.574735
Sr	3.943426	11.835358	7.810176	1.578503
Sr	3.942211	11.836731	11.545248	1.823549
Sr	7.890260	0.000000	0.000000	1.316342
Sr	7.890260	3.945130	0.000000	1.316125
Sr	7.890260	3.945130	3.945120	1.574564
Sr	7.890292	3.942163	7.811680	1.577259
Sr	7.887341	3.942037	11.542816	1.836528
Sr	7.890260	7.890260	0.000000	1.316130
Sr	7.890260	7.890260	3.945120	1.574603
Sr	7.889850	7.889218	7.811424	1.576543
Sr	7.888461	7.890339	11.541568	1.836803
Sr	7.890260	0.000000	3.945120	1.574327
Sr	7.890449	0.000552	7.812608	1.577790
Sr	7.889581	-0.000363	11.547584	1.821821
Sr	7.890260	11.835390	0.000000	1.316332
Sr	7.890260	11.835390	3.945120	1.574752
Sr	7.888998	11.835311	7.811136	1.577986
Sr	7.885857	11.831634	11.546560	1.822364
Sr	0.000000	3.945130	0.000000	1.316330
Sr	0.000000	3.945130	3.945120	1.574449
Sr	-0.001578	3.945272	7.811424	1.577961
Sr	0.001310	3.945162	11.543776	1.828742
Sr	0.000000	7.890260	0.000000	1.316353
Sr	0.000000	7.890260	3.945120	1.574202
Sr	-0.002304	7.888477	7.812896	1.577459
Sr	-0.000899	7.889203	11.548352	1.821852
Sr	0.000000	0.000000	3.945120	1.574985
Sr	-0.001389	-0.000331	7.809472	1.578325
Sr	0.000521	-0.002446	11.541536	1.824620
Sr	0.000000	11.835390	0.000000	1.316435
Sr	0.000000	11.835390	3.945120	1.574705
Sr	-0.001105	11.834790	7.809760	1.578104
Sr	-0.001199	11.835469	11.541440	1.827261
Sr	11.835390	0.000000	0.000000	1.316322
Sr	11.835390	3.945130	0.000000	1.316137
Sr	11.835390	3.945130	3.945120	1.574433
Sr	11.835800	3.943378	7.816832	1.577640
Sr	11.842633	3.935835	11.575136	1.801068
Sr	11.835390	7.890260	0.000000	1.316135
Sr	11.835390	7.890260	3.945120	1.574575
Sr	11.836936	7.888871	7.812064	1.577438
Sr	11.837568	7.892201	11.544928	1.835656
Sr	11.835390	0.000000	3.945120	1.574392
Sr	11.833686	-0.000126	7.811232	1.577558
Sr	11.833844	-0.003172	11.543424	1.829629
Sr	11.835390	11.835390	0.000000	1.316311
Sr	11.835390	11.835390	3.945120	1.574583
Sr	11.834917	11.834790	7.811072	1.577894
Sr	11.838862	11.834759	11.545472	1.824176
Ti	1.972565	1.972565	1.972576	2.637463
Ti	1.972170	1.972249	5.898240	2.621753
Ti	1.970087	1.971855	9.837760	2.649432
Ti	1.971871	5.917127	5.898560	2.620619
Ti	1.970876	5.917837	9.840288	2.649343
Ti	1.972565	5.917695	1.972576	2.637452
Ti	1.972170	9.861862	5.899040	2.621317
Ti	1.970545	9.862367	9.840384	2.649225
Ti	1.972565	9.862825	1.972576	2.637455
Ti	1.970135	13.807229	9.837088	2.650612
Ti	1.972565	13.807955	1.972576	2.637458
Ti	1.972565	13.807182	5.897760	2.621800
Ti	5.917695	1.972565	1.972576	2.637486
Ti	5.918389	1.971855	5.897856	2.621281
Ti	5.915407	1.970766	9.837632	2.650593
Ti	5.917111	5.916985	5.898688	2.620100

```
Ti       5.916527       5.917016       9.841088        2.647135
Ti       5.917695       5.917695       1.972576        2.637418
Ti       5.917569       9.862273       5.897472        2.621127
Ti       5.914602       9.863220       9.836416        2.650042
Ti       5.917695       9.862825       1.972576        2.637456
Ti       5.915423      13.807182       9.840128        2.649278
Ti       5.917695      13.807955       1.972576        2.637449
Ti       5.917995      13.807576       5.898912        2.621141
Ti       9.863014       1.972691       5.901984        2.621112
Ti       9.863125       1.971334       9.849120        2.647407
Ti       9.863519       5.916433       5.897024        2.618815
Ti       9.866076       5.912472       9.840608        2.644709
Ti       9.862825       5.917695       1.972576        2.637405
Ti       9.862825       9.862730       5.898784        2.619476
Ti       9.861184       9.861515       9.841408        2.647296
Ti       9.862825       9.862825       1.972576        2.637413
Ti       9.859937      13.807040       9.840032        2.648584
Ti       9.862825      13.807955       1.972576        2.637455
Ti       9.862604      13.807939       5.898560        2.620500
Ti       9.862825       1.972565       1.972576        2.637485
Ti      13.807513       1.972376       5.897344        2.621210
Ti      13.805477       1.972360       9.837664        2.650313
Ti      13.807119       5.916922       5.901984        2.620693
Ti      13.806677       5.915075       9.849856        2.646649
Ti      13.807955       5.917695       1.972576        2.637485
Ti      13.807955       9.861499       5.898048        2.621547
Ti      13.807071       9.862494       9.838400        2.651057
Ti      13.807955       9.862825       1.972576        2.637489
Ti      13.805793      13.807403       9.838112        2.649992
Ti      13.807955      13.807955       1.972576        2.637466
Ti      13.807608      13.807671       5.898176        2.621636
Ti      13.807955       1.972565       1.972576        2.637478
C        9.322610       6.416928      17.529280        4.172486
H        8.124222       7.609493      18.378848        0.948104
O        8.788487       6.946538      18.677088        2.277623
O       10.222779       5.515670      17.730208        2.377450
```

1.1.6 CO

```
       338
jmolscript: load "" {1 1 1} spacegroup "x,y,z" unitcell [{      15.780520
       0.000000      0.000000 }, {      0.000000    15.780520      0.000000
    }, {      0.000000      0.000000    32.000000 }]
O        1.972565       1.972565       3.945120        1.918754
O        1.968762       1.973212       7.858976        1.908561
O        1.960698       1.972628      11.791552        1.952542
O        1.972565       1.972565       0.000000        1.519134
O        1.972565       3.945130       1.972576        1.922856
O        1.969835       3.945982       5.901088        1.928475
O        1.969062       3.945935       9.801344        1.982021
O        1.972565       5.917695       3.945120        1.918882
O        1.977820       5.919636       7.861088        1.908009
O        1.947174       5.918026      11.792864        1.960979
O        1.972565       5.917695       0.000000        1.518963
O        1.972565       7.890260       1.972576        1.922876
O        1.970214       7.890165       5.900096        1.928380
O        1.968841       7.890970       9.804256        1.982335
O        1.966852       9.861531       7.859744        1.908240
O        1.962671       9.869121      11.792064        1.955261
O        1.972565       9.862825       0.000000        1.519014
O        1.972565       9.862825       3.945120        1.919022
O        1.972565       0.000000       1.972576        1.922846
O        1.970955       0.000442       5.900064        1.928937
O        1.966916       0.000552       9.800288        1.982817
O        1.972565      11.835390       1.972576        1.922855
O        1.970987      11.835059       5.901024        1.928805
O        1.967168      11.836226       9.798592        1.983145
```

O	1.962513	13.809186	11.790912	1.954561
O	1.972565	13.807955	0.000000	1.519106
O	1.972565	13.807955	3.945120	1.919228
O	1.967231	13.809249	7.858784	1.909621
O	3.945130	1.972565	1.972576	1.922862
O	3.943363	1.974033	5.900512	1.928987
O	3.939323	1.972155	9.800832	1.982466
O	3.943726	5.917632	5.898656	1.929721
O	3.940948	5.918342	9.815392	1.981796
O	3.945130	5.917695	1.972576	1.922700
O	3.943394	9.862462	5.902048	1.928042
O	3.939433	9.863267	9.799872	1.982586
O	3.945130	9.862825	1.972576	1.922881
O	3.939086	13.808744	9.798560	1.983369
O	3.945130	13.807955	1.972576	1.922848
O	3.943426	13.807639	5.901056	1.928812
O	5.917695	1.972565	3.945120	1.918527
O	5.913434	1.973449	7.859520	1.907677
O	5.905434	1.977173	11.791808	1.950723
O	5.917695	3.945130	1.972576	1.922894
O	5.917064	3.945745	5.901312	1.928815
O	5.912061	3.946377	9.802336	1.983194
O	5.917695	5.917695	3.945120	1.917925
O	5.909252	5.919399	7.862528	1.906149
O	5.923550	5.918531	11.799648	1.941530
O	5.917695	5.917695	0.000000	1.518797
O	5.917695	7.890260	1.972576	1.922871
O	5.916022	7.890418	5.900480	1.928935
O	5.912835	7.890023	9.803968	1.983486
O	5.915533	9.861152	7.860256	1.909721
O	5.907974	9.864703	11.789568	1.975501
O	5.917695	9.862825	0.000000	1.518803
O	5.917695	9.862825	3.945120	1.920020
O	5.915217	0.000363	5.900640	1.928746
O	5.913576	0.000710	9.802144	1.982594
O	5.917695	11.835390	1.972576	1.922886
O	5.915722	11.835106	5.901984	1.928121
O	5.912235	11.836005	9.799744	1.982916
O	5.902388	13.809075	11.792064	1.954721
O	5.917695	13.807955	0.000000	1.519009
O	5.917695	13.807955	3.945120	1.919032
O	5.915154	13.809722	7.859840	1.908197
O	5.917695	1.972565	0.000000	1.519052
O	5.917695	0.000000	1.972576	1.922836
O	7.890260	1.972565	1.972576	1.922875
O	7.888493	1.972218	5.900704	1.928672
O	7.884342	1.973638	9.801728	1.982550
O	7.888887	5.918389	5.906208	1.927898
O	7.886015	5.916859	9.800384	1.982110
O	7.890260	5.917695	1.972576	1.923111
O	7.888272	9.862935	5.900672	1.928598
O	7.885447	9.863283	9.803904	1.983298
O	7.890260	9.862825	1.972576	1.922879
O	7.884358	13.806724	9.804512	1.982178
O	7.890260	13.807955	1.972576	1.922873
O	7.888445	13.808034	5.900000	1.928744
O	9.862825	1.972565	3.945120	1.918600
O	9.858548	1.983154	7.861024	1.908395
O	9.850753	1.956721	11.793696	1.957205
O	9.860205	3.946156	5.898784	1.929638
O	9.858170	3.946282	9.817504	1.983274
O	9.862825	5.917695	3.945120	1.919081
O	9.865239	5.912124	7.866784	1.905097
O	9.841221	5.929231	11.809344	1.955707
O	9.862825	5.917695	0.000000	1.518605
O	9.862825	7.890260	1.972576	1.923104
O	9.859606	7.889771	5.906080	1.927972
O	9.858959	7.889297	9.801248	1.981971
O	9.857681	9.867449	7.862656	1.905523
O	9.853199	9.848701	11.800320	1.942016
O	9.862825	9.862825	0.000000	1.518795
O	9.862825	9.862825	3.945120	1.917898

99	O	9.857554	0.000032	9.790464	1.981729
100	O	9.862825	11.835390	1.972576	1.922709
101	O	9.861247	11.834775	5.898624	1.929892
102	O	9.857791	11.834601	9.815968	1.982264
103	O	9.853925	13.825030	11.793376	1.960480
104	O	9.862825	13.807955	0.000000	1.518959
105	O	9.862825	13.807955	3.945120	1.918656
106	O	9.856765	13.798487	7.861152	1.908322
107	O	9.862825	1.972565	0.000000	1.518924
108	O	9.862825	3.945130	1.972576	1.922718
109	O	9.862825	0.000000	1.972576	1.923047
110	O	9.860332	0.000268	5.907488	1.927340
111	O	0.000000	1.972565	1.972576	1.922868
112	O	-0.002146	1.972896	5.900736	1.929016
113	O	-0.005350	1.973543	9.798368	1.982433
114	O	-0.002178	5.917790	5.907456	1.927497
115	O	-0.005160	5.918200	9.790400	1.981602
116	O	0.000000	5.917695	1.972576	1.923030
117	O	-0.002146	9.863440	5.900608	1.929021
118	O	-0.005933	9.862462	9.802496	1.982458
119	O	0.000000	9.862825	1.972576	1.922798
120	O	-0.005918	13.808460	9.800576	1.982468
121	O	0.000000	13.807955	1.972576	1.922809
122	O	-0.002272	13.807545	5.900128	1.929069
123	O	11.835390	1.972565	1.972576	1.922892
124	O	11.832644	1.972770	5.900448	1.928960
125	O	11.829583	1.973165	9.803264	1.982593
126	O	11.832407	5.918642	5.898496	1.929399
127	O	11.829236	5.918011	9.818432	1.983406
128	O	11.835390	5.917695	1.972576	1.922745
129	O	11.832912	9.861121	5.901408	1.928960
130	O	11.829141	9.863567	9.802720	1.983729
131	O	11.835390	9.862825	1.972576	1.922923
132	O	11.829488	13.806835	9.801184	1.982034
133	O	11.835390	13.807955	1.972576	1.922892
134	O	11.832581	13.808902	5.901216	1.928675
135	O	13.802369	1.974143	7.859040	1.908130
136	O	13.802669	1.968336	11.790720	1.949862
137	O	13.805477	3.945809	5.900448	1.928674
138	O	13.802384	3.945745	9.803488	1.982299
139	O	13.807955	5.917695	3.945120	1.918506
140	O	13.793169	5.917774	7.861280	1.908380
141	O	13.815640	5.920662	11.793856	1.957701
142	O	13.807955	7.890260	1.972576	1.922900
143	O	13.806598	7.890118	5.900864	1.928730
144	O	13.802353	7.890939	9.802048	1.982559
145	O	13.803347	9.863220	7.859712	1.907197
146	O	13.793958	9.866376	11.792224	1.951179
147	O	13.807955	9.862825	0.000000	1.519041
148	O	13.807955	9.862825	3.945120	1.918647
149	O	13.802369	-0.000063	9.798336	1.982538
150	O	13.807955	11.835390	1.972576	1.922868
151	O	13.804373	11.835153	5.900544	1.928644
152	O	13.803426	11.836053	9.801056	1.982603
153	O	13.798360	13.810795	11.791744	1.952773
154	O	13.807955	13.807955	0.000000	1.519122
155	O	13.807955	13.807955	3.945120	1.918823
156	O	13.803473	13.807671	7.859168	1.908228
157	O	13.807955	1.972565	3.945120	1.918544
158	O	13.807955	1.972565	0.000000	1.519122
159	O	13.807955	3.945130	1.972576	1.922868
160	O	13.807955	5.917695	0.000000	1.518916
161	O	13.807955	0.000000	1.972576	1.922860
162	O	13.805777	0.000284	5.900768	1.928844
163	Pd	1.920805	1.975248	13.965088	3.100732
164	Pd	1.917554	3.952058	16.129696	2.970526
165	Pd	1.935686	5.923266	13.957920	3.105480
166	Pd	1.921026	7.910112	16.144480	2.952022
167	Pd	1.921436	9.884129	13.962624	3.099857
168	Pd	1.933776	-0.004324	16.154528	2.961067
169	Pd	1.929721	11.846957	16.127008	2.962680
170	Pd	1.944318	13.805146	13.962432	3.096376

171	Pd	3.892281	1.975405	16.156352	2.932590
172	Pd	3.883381	3.940569	14.392224	3.499897
173	Pd	3.877795	5.927952	16.127552	2.951737
174	Pd	3.865738	7.910128	14.387552	3.498088
175	Pd	3.911991	9.873540	16.141856	2.951034
176	Pd	3.892518	0.021035	14.391168	3.497117
177	Pd	3.914326	11.832360	14.389152	3.495490
178	Pd	3.903138	13.817123	16.127776	2.961966
179	Pd	5.861027	1.981181	13.967136	3.084220
180	Pd	5.859496	3.958922	16.132736	2.952808
181	Pd	5.839897	5.919731	13.973824	3.073261
182	Pd	5.871663	7.906656	16.084128	2.918315
183	Pd	5.873320	9.877043	13.941312	3.089040
184	Pd	5.870006	0.015339	16.122784	2.974334
185	Pd	5.873951	11.837221	16.142304	2.949894
186	Pd	5.865241	13.828470	13.962912	3.100181
187	Pd	7.834697	2.000165	16.117088	2.965582
188	Pd	7.837758	3.980826	14.387392	3.451843
189	Pd	7.876168	5.894435	16.237152	3.401426
190	Pd	7.876310	7.867789	14.385024	3.348494
191	Pd	7.844339	9.873840	16.083136	2.918400
192	Pd	7.856269	0.011883	14.392256	3.473937
193	Pd	7.837017	11.882858	14.389504	3.499177
194	Pd	7.839463	13.827539	16.146464	2.952240
195	Pd	9.827808	1.998934	13.960768	3.096839
196	Pd	9.854414	3.901686	16.104704	2.938083
197	Pd	9.866344	5.880990	13.963872	3.069327
198	Pd	9.847565	7.861855	16.240672	3.394496
199	Pd	9.825630	9.910782	13.974912	3.074875
200	Pd	9.818624	0.016001	16.141408	2.940208
201	Pd	9.817582	11.873279	16.131776	2.950158
202	Pd	9.827193	13.815435	13.958880	3.105073
203	Pd	15.753030	1.965416	16.134144	2.980129
204	Pd	15.721169	3.954441	14.391648	3.477601
205	Pd	15.733936	5.927053	16.140416	2.936707
206	Pd	15.734188	7.888714	14.392384	3.473780
207	Pd	15.734583	9.876459	16.121216	2.974377
208	Pd	15.754798	-0.012167	14.396000	3.512335
209	Pd	15.724625	11.854248	14.391648	3.497486
210	Pd	15.751010	13.811632	16.156640	2.959755
211	Pd	11.818379	1.960288	16.170944	2.953156
212	Pd	11.852433	3.891255	14.379808	3.500665
213	Pd	11.851171	5.890347	16.103872	2.941784
214	Pd	11.758555	7.907950	14.388960	3.450387
215	Pd	11.785729	9.885139	16.134336	2.947942
216	Pd	11.790999	0.025470	14.392224	3.479072
217	Pd	11.802851	11.860560	14.394624	3.500635
218	Pd	11.796491	13.829511	16.129856	2.972588
219	Pd	13.807671	1.941288	13.968384	3.120953
220	Pd	13.785657	3.926399	16.168704	2.954642
221	Pd	13.747737	5.920820	13.960032	3.094671
222	Pd	13.745149	7.915351	16.114112	2.967237
223	Pd	13.767241	9.888200	13.967904	3.082351
224	Pd	13.779298	-0.003456	16.135616	2.978388
225	Pd	13.769482	11.856441	16.156032	2.932302
226	Pd	13.772039	13.826623	13.965664	3.100789
227	Sr	0.000000	0.000000	0.000000	1.316202
228	Sr	3.945130	0.000000	0.000000	1.316131
229	Sr	3.945130	3.945130	0.000000	1.315998
230	Sr	3.945130	3.945130	3.945120	1.574849
231	Sr	3.943568	3.944499	7.811200	1.577833
232	Sr	3.943852	3.941232	11.545472	1.824044
233	Sr	3.945130	7.890260	0.000000	1.315921
234	Sr	3.945130	7.890260	3.945120	1.575019
235	Sr	3.942873	7.889518	7.810048	1.578262
236	Sr	3.945177	7.894158	11.542400	1.824674
237	Sr	3.945130	0.000000	3.945120	1.574889
238	Sr	3.942826	0.000363	7.809888	1.578429
239	Sr	3.942558	-0.001831	11.543136	1.824872
240	Sr	3.945130	11.835390	0.000000	1.316053
241	Sr	3.945130	11.835390	3.945120	1.574948
242	Sr	3.942384	11.836037	7.809056	1.578259

Sr	3.940980	11.837315	11.540864	1.826697
Sr	7.890260	0.000000	0.000000	1.316029
Sr	7.890260	3.945130	0.000000	1.315824
Sr	7.890260	3.945130	3.945120	1.574628
Sr	7.887909	3.944625	7.811488	1.577692
Sr	7.883932	3.943315	11.544864	1.825022
Sr	7.890260	7.890260	0.000000	1.315723
Sr	7.890260	7.890260	3.945120	1.574554
Sr	7.888556	7.889865	7.812256	1.577759
Sr	7.887293	7.891191	11.553216	1.827732
Sr	7.890260	0.000000	3.945120	1.574568
Sr	7.887798	-0.000110	7.810624	1.578241
Sr	7.889865	-0.001215	11.545312	1.824536
Sr	7.890260	11.835390	0.000000	1.315921
Sr	7.890260	11.835390	3.945120	1.574980
Sr	7.888887	11.835532	7.810272	1.578564
Sr	7.883916	11.833149	11.543040	1.824936
Sr	0.000000	3.945130	0.000000	1.316093
Sr	0.000000	3.945130	3.945120	1.574508
Sr	-0.002399	3.944073	7.810528	1.578079
Sr	-0.000284	3.945998	11.544480	1.825127
Sr	0.000000	7.890260	0.000000	1.316030
Sr	0.000000	7.890260	3.945120	1.574432
Sr	-0.002130	7.890560	7.810624	1.577950
Sr	-0.000694	7.888571	11.545280	1.824345
Sr	0.000000	0.000000	3.945120	1.574998
Sr	-0.002604	0.000316	7.809920	1.578745
Sr	-0.002383	0.000395	11.543520	1.824187
Sr	0.000000	11.835390	0.000000	1.316132
Sr	0.000000	11.835390	3.945120	1.575006
Sr	-0.002620	11.835579	7.809888	1.578182
Sr	-0.000221	11.835832	11.543392	1.824178
Sr	11.835390	0.000000	0.000000	1.316088
Sr	11.835390	3.945130	0.000000	1.315902
Sr	11.835390	3.945130	3.945120	1.575118
Sr	11.833844	3.944467	7.811136	1.577756
Sr	11.834680	3.943552	11.541856	1.828840
Sr	11.835390	7.890260	0.000000	1.315827
Sr	11.835390	7.890260	3.945120	1.574741
Sr	11.833654	7.890497	7.811648	1.577686
Sr	11.835611	7.894663	11.545920	1.823388
Sr	11.835390	0.000000	3.945120	1.574579
Sr	11.834143	0.000142	7.810560	1.577525
Sr	11.832439	-0.001878	11.544544	1.824996
Sr	11.835390	11.835390	0.000000	1.316009
Sr	11.835390	11.835390	3.945120	1.574732
Sr	11.833717	11.834790	7.811520	1.577951
Sr	11.837157	11.834775	11.546336	1.823008
Ti	1.972565	1.972565	1.972576	2.637497
Ti	1.971650	1.972344	5.898240	2.621539
Ti	1.969093	1.972281	9.838464	2.649705
Ti	1.972376	5.917316	5.898304	2.620854
Ti	1.969661	5.917411	9.838816	2.649071
Ti	1.972565	5.917695	1.972576	2.637481
Ti	1.971445	9.862667	5.897920	2.621547
Ti	1.969235	9.863251	9.838208	2.649806
Ti	1.972565	9.862825	1.972576	2.637497
Ti	1.968683	13.808665	9.836736	2.649875
Ti	1.972565	13.807955	1.972576	2.637472
Ti	1.971381	13.808081	5.897728	2.621550
Ti	5.917695	1.972565	1.972576	2.637524
Ti	5.916859	1.972423	5.898464	2.621679
Ti	5.914271	1.971776	9.839776	2.649898
Ti	5.916575	5.917411	5.899008	2.620617
Ti	5.915549	5.917853	9.842112	2.647056
Ti	5.917695	5.917695	1.972576	2.637493
Ti	5.917285	9.862225	5.896864	2.621173
Ti	5.914918	9.862573	9.834656	2.651165
Ti	5.917695	9.862825	1.972576	2.637500
Ti	5.914081	13.808002	9.838144	2.649777
Ti	5.917695	13.807955	1.972576	2.637488
Ti	5.916843	13.807908	5.898016	2.621412

```
Ti       9.861926       1.973038       5.898496        2.621066
Ti       9.859480       1.971255       9.839520        2.649985
Ti       9.862509       5.916985       5.897984        2.619512
Ti       9.859180       5.918405       9.841440        2.646516
Ti       9.862825       5.917695       1.972576        2.637464
Ti       9.862020       9.862904       5.899040        2.620380
Ti       9.859606       9.862020       9.842592        2.647701
Ti       9.862825       9.862825       1.972576        2.637485
Ti       9.859874      13.807592       9.839264        2.649551
Ti       9.862825      13.807955       1.972576        2.637478
Ti       9.862099      13.806945       5.898368        2.620887
Ti       9.862825       1.972565       1.972576        2.637489
Ti      13.807103       1.972328       5.898464        2.621716
Ti      13.805477       1.971823       9.839904        2.650227
Ti      13.806298       5.917490       5.898496        2.621083
Ti      13.806077       5.917869       9.839712        2.649214
Ti      13.807955       5.917695       1.972576        2.637493
Ti      13.806945       9.862604       5.898400        2.621607
Ti      13.805414       9.863220       9.840192        2.650116
Ti      13.807955       9.862825       1.972576        2.637516
Ti      13.804846      13.808113       9.838720        2.649789
Ti      13.807955      13.807955       1.972576        2.637492
Ti      13.807024      13.807734       5.898176        2.621386
Ti      13.807955       1.972565       1.972576        2.637509
C        8.749178       6.988072      17.632736        4.140142
O        8.708796       7.026308      18.815488        2.312125
```

1.1.7 COHOH

```
       341
jmolscript: load "" {1 1 1} spacegroup "x,y,z" unitcell [{      15.780520
       0.000000      0.000000 }, {       0.000000    15.780520      0.000000
    }, {      0.000000      0.000000     32.000000 }]
O        1.972565       1.972565       3.945120        1.918224
O        1.968304       1.971460       7.860896        1.907517
O        1.965969       1.974901      11.794816        1.954002
O        1.972565       1.972565       0.000000        1.519233
O        1.972565       3.945130       1.972576        1.922852
O        1.971271       3.945367       5.902368        1.928179
O        1.968462       3.945020       9.800960        1.982397
O        1.972565       5.917695       3.945120        1.918099
O        1.968304       5.919857       7.861280        1.906987
O        1.964896       5.913134      11.794880        1.952811
O        1.972565       5.917695       0.000000        1.519138
O        1.972565       7.890260       1.972576        1.922866
O        1.970940       7.890718       5.901248        1.928609
O        1.969835       7.890465       9.807168        1.981944
O        1.974885       9.861910       7.863360        1.905226
O        1.954907       9.864924      11.797024        1.959019
O        1.972565       9.862825       0.000000        1.519072
O        1.972565       9.862825       3.945120        1.917775
O        1.972565       0.000000       1.972576        1.922853
O        1.971129      -0.000079       5.901152        1.928764
O        1.968604       0.000205       9.804640        1.982118
O        1.972565      11.835390       1.972576        1.922908
O        1.970813      11.834964       5.902272        1.928135
O        1.969945      11.835437       9.805056        1.981886
O        1.964848      13.809375      11.795776        1.950680
O        1.972565      13.807955       0.000000        1.519243
O        1.972565      13.807955       3.945120        1.917568
O        1.970119      13.808129       7.861248        1.906561
O        3.945130       1.972565       1.972576        1.922872
O        3.943726       1.972770       5.902304        1.928126
O        3.940601       1.972391       9.801664        1.982354
O        3.943804       5.918200       5.902112        1.927629
O        3.940617       5.917537       9.802048        1.982506
O        3.945130       5.917695       1.972576        1.922860
```

O	3.944120	9.862888	5.901280	1.928894
O	3.942558	9.863220	9.814560	1.981685
O	3.945130	9.862825	1.972576	1.922802
O	3.940853	13.808223	9.804032	1.982189
O	3.945130	13.807955	1.972576	1.922859
O	3.943710	13.807434	5.901440	1.928506
O	5.917695	1.972565	3.945120	1.918022
O	5.916180	1.971239	7.861312	1.906800
O	5.905954	1.973607	11.795040	1.951910
O	5.917695	3.945130	1.972576	1.922828
O	5.916275	3.945367	5.902272	1.927739
O	5.913592	3.944925	9.801696	1.982774
O	5.917695	5.917695	3.945120	1.919395
O	5.915502	5.918768	7.860736	1.909534
O	5.910420	5.917348	11.789856	1.975642
O	5.917695	5.917695	0.000000	1.518945
O	5.917695	7.890260	1.972576	1.922876
O	5.916559	7.890560	5.901856	1.927970
O	5.914192	7.891017	9.803872	1.983564
O	5.913371	9.863598	7.863616	1.904808
O	5.924354	9.860647	11.801216	1.943566
O	5.917695	9.862825	0.000000	1.518937
O	5.917695	9.862825	3.945120	1.917281
O	5.916117	-0.000110	5.901536	1.928560
O	5.914492	0.000110	9.804960	1.982496
O	5.917695	11.835390	1.972576	1.922900
O	5.916859	11.835138	5.901664	1.928875
O	5.914239	11.834932	9.806656	1.982931
O	5.907737	13.806314	11.795488	1.948131
O	5.917695	13.807955	0.000000	1.519166
O	5.917695	13.807955	3.945120	1.917181
O	5.914271	13.807056	7.861472	1.906131
O	5.917695	1.972565	0.000000	1.519144
O	5.917695	0.000000	1.972576	1.922874
O	7.890260	1.972565	1.972576	1.922841
O	7.889029	1.972817	5.901152	1.928350
O	7.886015	1.973875	9.806848	1.981852
O	7.888950	5.917932	5.901888	1.927839
O	7.886883	5.918042	9.803808	1.983300
O	7.890260	5.917695	1.972576	1.922881
O	7.889313	9.862904	5.904288	1.927905
O	7.887956	9.863456	9.804544	1.982485
O	7.890260	9.862825	1.972576	1.922991
O	7.885794	13.808634	9.804352	1.982691
O	7.890260	13.807955	1.972576	1.922903
O	7.889187	13.808176	5.902112	1.927979
O	9.862825	1.972565	3.945120	1.917868
O	9.858249	1.977725	7.863040	1.905293
O	9.856607	1.963160	11.796640	1.959067
O	9.861452	3.945777	5.901568	1.928825
O	9.859558	3.946740	9.813760	1.981822
O	9.862825	5.917695	3.945120	1.917223
O	9.860300	5.917411	7.863680	1.904390
O	9.853325	5.930446	11.801376	1.943459
O	9.862825	5.917695	0.000000	1.518942
O	9.862825	7.890260	1.972576	1.922971
O	9.860805	7.890891	5.903840	1.928107
O	9.859858	7.892296	9.806304	1.982494
O	9.862525	9.864640	7.865632	1.904126
O	9.852236	9.862841	11.807936	1.950507
O	9.862825	9.862825	0.000000	1.518798
O	9.862825	9.862825	3.945120	1.917882
O	9.858817	0.000615	9.800000	1.981758
O	9.862825	11.835390	1.972576	1.922834
O	9.861184	11.835011	5.901792	1.928548
O	9.859195	11.835295	9.810080	1.983389
O	9.852757	13.814425	11.795040	1.961665
O	9.862825	13.807955	0.000000	1.519024
O	9.862825	13.807955	3.945120	1.918110
O	9.860379	13.804610	7.862400	1.906699
O	9.862825	1.972565	0.000000	1.519077
O	9.862825	3.945130	1.972576	1.922798

O	9.862825	0.000000	1.972576	1.922938
O	9.861136	0.000316	5.904768	1.927901
O	0.000000	1.972565	1.972576	1.922858
O	-0.001625	1.972817	5.901152	1.928396
O	-0.004198	1.972360	9.804448	1.982164
O	-0.001594	5.917695	5.901440	1.928376
O	-0.004245	5.918437	9.804608	1.982478
O	0.000000	5.917695	1.972576	1.922849
O	-0.001310	9.863014	5.905408	1.927629
O	-0.003724	9.863156	9.799872	1.981632
O	0.000000	9.862825	1.972576	1.922936
O	-0.003551	13.807482	9.802208	1.981976
O	0.000000	13.807955	1.972576	1.922866
O	-0.001562	13.807876	5.901920	1.928304
O	11.835390	1.972565	1.972576	1.922902
O	11.833354	1.972455	5.902272	1.928118
O	11.830987	1.973733	9.804320	1.981913
O	11.833559	5.918768	5.901632	1.928536
O	11.830640	5.918184	9.806112	1.983335
O	11.835390	5.917695	1.972576	1.922900
O	11.833433	9.862667	5.901248	1.928389
O	11.830829	9.862872	9.811584	1.983420
O	11.835390	9.862825	1.972576	1.922816
O	11.831429	13.808207	9.806752	1.981881
O	11.835390	13.807955	1.972576	1.922872
O	11.833465	13.808034	5.901536	1.928240
O	13.804941	1.973922	7.861088	1.906903
O	13.800759	1.972644	11.795232	1.951717
O	13.805730	3.945319	5.901120	1.928769
O	13.804562	3.945256	9.804192	1.982289
O	13.807955	5.917695	3.945120	1.917318
O	13.803757	5.917095	7.861216	1.906299
O	13.797698	5.916874	11.795008	1.949600
O	13.807955	7.890260	1.972576	1.922916
O	13.806756	7.890860	5.902432	1.928048
O	13.804925	7.890228	9.803232	1.982528
O	13.800412	9.864719	7.862528	1.906645
O	13.807056	9.859069	11.795360	1.960945
O	13.807955	9.862825	0.000000	1.519029
O	13.807955	9.862825	3.945120	1.917976
O	13.803742	0.000805	9.801440	1.981960
O	13.807955	11.835390	1.972576	1.922863
O	13.806203	11.835138	5.901120	1.928683
O	13.804168	11.835785	9.808000	1.981734
O	13.806535	13.815356	11.795296	1.948254
O	13.807955	13.807955	0.000000	1.519217
O	13.807955	13.807955	3.945120	1.917316
O	13.803884	13.806456	7.861504	1.906119
O	13.807955	1.972565	3.945120	1.917790
O	13.807955	1.972565	0.000000	1.519238
O	13.807955	3.945130	1.972576	1.922815
O	13.807955	5.917695	0.000000	1.519163
O	13.807955	0.000000	1.972576	1.922908
O	13.806188	0.000016	5.902176	1.928098
Pd	1.960824	1.992701	13.965248	3.093372
Pd	1.944996	3.947213	16.127712	2.962817
Pd	1.926502	5.896802	13.967072	3.107873
Pd	1.929831	7.878945	16.161984	2.940502
Pd	1.942203	9.876475	13.962528	3.108494
Pd	1.949399	0.017390	16.162848	2.954431
Pd	1.932893	11.835721	16.130144	2.969115
Pd	1.933240	13.811742	13.969664	3.095172
Pd	3.917135	1.980329	16.128736	2.962219
Pd	3.934163	3.968880	14.395328	3.490739
Pd	3.930391	5.928394	16.149312	2.951032
Pd	3.849121	7.878882	14.380928	3.477866
Pd	3.897252	9.852599	16.129152	2.950775
Pd	3.903801	-0.011583	14.397152	3.500992
Pd	3.909103	11.845332	14.402624	3.500482
Pd	3.905679	13.812642	16.160736	2.929662
Pd	5.867734	1.957242	13.967936	3.106474
Pd	5.894924	3.964603	16.149312	2.950075

181	Pd	5.900368	5.927763	13.943104	3.067867
182	Pd	5.890126	7.880571	16.062816	3.037664
183	Pd	5.852174	9.864466	13.974560	3.068117
184	Pd	5.878922	-0.006107	16.129312	2.973048
185	Pd	5.879601	11.817542	16.150048	2.926446
186	Pd	5.873273	13.812484	13.972384	3.083207
187	Pd	7.848426	1.961029	16.163456	2.940113
188	Pd	7.847747	3.881187	14.384224	3.482388
189	Pd	7.854864	5.921325	16.065888	3.031871
190	Pd	7.911879	7.942067	14.390016	3.367519
191	Pd	7.915667	9.893455	16.254784	3.264052
192	Pd	7.866573	0.008001	14.400416	3.480149
193	Pd	7.838405	11.807411	14.388448	3.428683
194	Pd	7.852229	13.789381	16.113760	2.960250
195	Pd	9.846571	1.970229	13.962016	3.108329
196	Pd	9.822143	3.921933	16.131520	2.950973
197	Pd	9.832116	5.878291	13.975040	3.072405
198	Pd	9.856781	7.954282	16.261920	3.255916
199	Pd	9.873209	9.906095	13.970240	3.071520
200	Pd	9.833631	-0.009042	16.146368	2.944867
201	Pd	9.868459	11.881580	16.091328	3.049770
202	Pd	9.839217	13.810953	13.958688	3.087018
203	Pd	15.761662	1.984053	16.163392	2.954765
204	Pd	15.732168	3.937398	14.397152	3.501526
205	Pd	15.742710	5.912424	16.128512	2.972419
206	Pd	15.756234	7.900486	14.399840	3.479581
207	Pd	15.741526	9.865287	16.146784	2.942311
208	Pd	-0.005492	0.029999	14.400736	3.506754
209	Pd	15.732595	11.841718	14.399904	3.491876
210	Pd	-0.013350	13.829211	16.138048	2.974397
211	Pd	11.806307	1.964044	16.128352	2.973106
212	Pd	11.811088	3.942021	14.401056	3.499982
213	Pd	11.783630	5.912819	16.148800	2.926392
214	Pd	11.764693	7.872728	14.388960	3.425891
215	Pd	11.850381	9.901898	16.092256	3.048389
216	Pd	11.809841	-0.017516	14.398592	3.493243
217	Pd	11.876151	11.908422	14.376224	3.472940
218	Pd	11.822781	13.827633	16.178656	2.950989
219	Pd	13.779818	1.963207	13.968640	3.097108
220	Pd	13.776205	3.939307	16.161440	2.929643
221	Pd	13.780292	5.904408	13.971328	3.082206
222	Pd	13.753812	7.881470	16.108992	2.962197
223	Pd	13.780087	9.869074	13.959040	3.085191
224	Pd	13.795094	0.015607	16.139424	2.974289
225	Pd	13.795520	11.854611	16.177856	2.950149
226	Pd	13.823294	13.852645	13.972480	3.124975
227	Sr	0.000000	0.000000	0.000000	1.316342
228	Sr	3.945130	0.000000	0.000000	1.316276
229	Sr	3.945130	3.945130	0.000000	1.316200
230	Sr	3.945130	3.945130	3.945120	1.574409
231	Sr	3.943157	3.944499	7.810880	1.577466
232	Sr	3.942132	3.943552	11.545248	1.824470
233	Sr	3.945130	7.890260	0.000000	1.316077
234	Sr	3.945130	7.890260	3.945120	1.574618
235	Sr	3.943441	7.890986	7.811200	1.577564
236	Sr	3.947323	7.886457	11.542752	1.829280
237	Sr	3.945130	0.000000	3.945120	1.574396
238	Sr	3.943662	-0.000663	7.812000	1.577674
239	Sr	3.942873	0.001168	11.547168	1.823964
240	Sr	3.945130	11.835390	0.000000	1.316153
241	Sr	3.945130	11.835390	3.945120	1.574431
242	Sr	3.944325	11.836037	7.813472	1.577143
243	Sr	3.944088	11.838404	11.551168	1.820624
244	Sr	7.890260	0.000000	0.000000	1.316175
245	Sr	7.890260	3.945130	0.000000	1.316071
246	Sr	7.890260	3.945130	3.945120	1.574561
247	Sr	7.889455	3.944830	7.811296	1.577577
248	Sr	7.885084	3.948460	11.543584	1.828521
249	Sr	7.890260	7.890260	0.000000	1.315890
250	Sr	7.890260	7.890260	3.945120	1.574294
251	Sr	7.889234	7.890686	7.813184	1.577387
252	Sr	7.886126	7.887909	11.553472	1.825157

Sr	7.890260	0.000000	3.945120	1.574246
Sr	7.888303	0.000189	7.812544	1.577492
Sr	7.888682	0.001262	11.549920	1.821313
Sr	7.890260	11.835390	0.000000	1.315985
Sr	7.890260	11.835390	3.945120	1.574283
Sr	7.888556	11.835974	7.812832	1.577429
Sr	7.888477	11.837662	11.547392	1.825084
Sr	0.000000	3.945130	0.000000	1.316288
Sr	0.000000	3.945130	3.945120	1.574492
Sr	-0.002051	3.945020	7.811808	1.577567
Sr	0.000252	3.944215	11.546912	1.824199
Sr	0.000000	7.890260	0.000000	1.316176
Sr	0.000000	7.890260	3.945120	1.574191
Sr	-0.001168	7.889865	7.812416	1.577400
Sr	-0.000142	7.889944	11.549504	1.821452
Sr	0.000000	0.000000	3.945120	1.574401
Sr	-0.001831	-0.000521	7.812128	1.577845
Sr	-0.002020	-0.000789	11.547648	1.822491
Sr	0.000000	11.835390	0.000000	1.316221
Sr	0.000000	11.835390	3.945120	1.574211
Sr	-0.001404	11.836211	7.812576	1.577463
Sr	-0.000095	11.835327	11.549024	1.822725
Sr	11.835390	0.000000	0.000000	1.316227
Sr	11.835390	3.945130	0.000000	1.316146
Sr	11.835390	3.945130	3.945120	1.574388
Sr	11.834554	3.945698	7.813184	1.577428
Sr	11.837047	3.945240	11.550144	1.821111
Sr	11.835390	7.890260	0.000000	1.315983
Sr	11.835390	7.890260	3.945120	1.574386
Sr	11.834459	7.889976	7.812896	1.577330
Sr	11.837804	7.889076	11.547680	1.824156
Sr	11.835390	0.000000	3.945120	1.574226
Sr	11.834680	0.000047	7.812288	1.577234
Sr	11.834080	0.001404	11.548160	1.823198
Sr	11.835390	11.835390	0.000000	1.316056
Sr	11.835390	11.835390	3.945120	1.574597
Sr	11.834364	11.835879	7.812096	1.577324
Sr	11.831634	11.833465	11.542304	1.832029
Ti	1.972565	1.972565	1.972576	2.637462
Ti	1.971744	1.972139	5.898752	2.620935
Ti	1.969851	1.972123	9.839968	2.648443
Ti	1.971871	5.917727	5.898816	2.620904
Ti	1.970072	5.917206	9.840576	2.648208
Ti	1.972565	5.917695	1.972576	2.637480
Ti	1.972486	9.862983	5.899136	2.619952
Ti	1.970640	9.863235	9.842368	2.648525
Ti	1.972565	9.862825	1.972576	2.637446
Ti	1.970293	13.808002	9.841696	2.647782
Ti	1.972565	13.807955	1.972576	2.637466
Ti	1.972202	13.807860	5.899232	2.620639
Ti	5.917695	1.972565	1.972576	2.637471
Ti	5.917222	1.972281	5.898912	2.620617
Ti	5.914997	1.972407	9.840832	2.649056
Ti	5.917506	5.917947	5.897536	2.620761
Ti	5.915644	5.917742	9.835648	2.651348
Ti	5.917695	5.917695	1.972576	2.637488
Ti	5.917111	9.863220	5.899616	2.620324
Ti	5.916732	9.863456	9.843840	2.647306
Ti	5.917695	9.862825	1.972576	2.637469
Ti	5.915123	13.808618	9.843040	2.648552
Ti	5.917695	13.807955	1.972576	2.637485
Ti	5.917095	13.807860	5.899680	2.620737
Ti	9.862446	1.972896	5.899008	2.620019
Ti	9.860710	1.973007	9.842048	2.648049
Ti	9.862715	5.917679	5.899616	2.619872
Ti	9.861121	5.918957	9.843968	2.647604
Ti	9.862825	5.917695	1.972576	2.637475
Ti	9.862573	9.862935	5.899008	2.619247
Ti	9.859985	9.862115	9.842848	2.646099
Ti	9.862825	9.862825	1.972576	2.637449
Ti	9.860221	13.809596	9.840640	2.649339
Ti	9.862825	13.807955	1.972576	2.637459

```
Ti      9.862304     13.807797      5.898848        2.620125
Ti      9.862825      1.972565      1.972576        2.637457
Ti     13.807403      1.972691      5.899104        2.620771
Ti     13.805793      1.972581      9.841248        2.647772
Ti     13.807371      5.917569      5.899520        2.620641
Ti     13.806314      5.917364      9.842432        2.648406
Ti     13.807955      5.917695      1.972576        2.637482
Ti     13.807198      9.862888      5.898944        2.620194
Ti     13.807245      9.862588      9.840864        2.648112
Ti     13.807955      9.862825      1.972576        2.637463
Ti     13.806235     13.808539      9.842752        2.647730
Ti     13.807955     13.807955      1.972576        2.637469
Ti     13.807497     13.807876      5.899488        2.620524
Ti     13.807955      1.972565      1.972576        2.637469
C       8.834882      8.879367     17.793344        4.093479
H       7.502343      7.550758     17.942048        1.021636
H      10.158268     10.205894     18.004928        1.013505
O       8.053809      8.102571     18.567200        2.335174
O       9.594556      9.642703     18.605088        2.328588
```

1.1.8 CH_2OH

```
       341
jmolscript: load "" {1 1 1} spacegroup "x,y,z" unitcell [{      15.780520
       0.000000      0.000000 }, {      0.000000    15.780520      0.000000
    }, {      0.000000      0.000000    32.000000 }]
O       0.000000      1.972565      1.972576        1.922774
O       0.000000      5.917695      1.972576        1.922782
O       0.000000      9.862825      1.972576        1.922739
O       0.000000     13.807955      1.972576        1.922745
O       1.959688      5.919841     11.793216        1.953714
O       1.958820      9.865208     11.794176        1.963130
O       1.963207      1.972881     11.792480        1.954324
O       1.961124     13.808018     11.792832        1.959599
O       1.967137     11.835721      9.802304        1.982228
O       1.967089      0.000694      9.801088        1.982584
O       1.967689      3.946298      9.802432        1.982121
O       1.967421      7.891254      9.803392        1.982251
O       1.967752      1.973654      7.859232        1.908404
O       1.969661      5.918973      7.860192        1.907307
O       1.970308      9.863204      7.861440        1.907574
O       1.969030     13.807497      7.860128        1.908069
O       1.969661      3.945588      5.900832        1.928760
O       1.971381      0.000205      5.901344        1.928563
O       1.971050      7.890860      5.900832        1.928726
O       1.969456     11.835311      5.900960        1.928547
O       1.972565      0.000000      1.972576        1.922772
O       1.972565      1.972565      3.945120        1.918273
O       1.972565      1.972565      0.000000        1.520457
O       1.972565      3.945130      1.972576        1.922719
O       1.972565      5.917695      3.945120        1.917818
O       1.972565      5.917695      0.000000        1.520453
O       1.972565      7.890260      1.972576        1.922762
O       1.972565      9.862825      0.000000        1.520385
O       1.972565      9.862825      3.945120        1.918838
O       1.972565     11.835390      1.972576        1.922766
O       1.972565     13.807955      0.000000        1.520447
O       1.972565     13.807955      3.945120        1.918439
O       3.939228     13.809438      9.803104        1.982326
O       3.939039      1.974001      9.799936        1.983015
O       3.938976      5.918500      9.804448        1.982099
O       3.939307      9.863267      9.807136        1.982336
O       3.943000     13.807355      5.900416        1.928917
O       3.943000      1.973685      5.900512        1.928681
O       3.943094      5.917758      5.900512        1.928886
O       3.942873      9.863835      5.900544        1.929139
O       3.945130      1.972565      1.972576        1.922727
```

O	3.945130	5.917695	1.972576	1.922742
O	3.945130	9.862825	1.972576	1.922687
O	3.945130	13.807955	1.972576	1.922706
O	5.903619	1.972754	11.792640	1.954464
O	5.913229	9.866802	11.796640	1.935878
O	5.907737	5.921656	11.793280	1.954474
O	5.914365	13.814078	11.786976	1.963819
O	5.911241	11.836605	9.801696	1.983437
O	5.912267	0.002320	9.798208	1.982518
O	5.911856	7.891964	9.804320	1.982142
O	5.912409	3.946629	9.803424	1.982583
O	5.912882	9.863456	7.860416	1.905838
O	5.912677	5.918957	7.860512	1.907172
O	5.912614	13.808034	7.858304	1.909664
O	5.913639	1.975958	7.859488	1.908576
O	5.915075	0.000631	5.901312	1.927943
O	5.916575	11.835721	5.900224	1.928945
O	5.916448	3.945856	5.900000	1.928879
O	5.915328	7.890702	5.900800	1.929032
O	5.917695	1.972565	3.945120	1.918626
O	5.917695	3.945130	1.972576	1.922703
O	5.917695	5.917695	3.945120	1.917683
O	5.917695	5.917695	0.000000	1.520510
O	5.917695	7.890260	1.972576	1.922818
O	5.917695	9.862825	0.000000	1.520558
O	5.917695	9.862825	3.945120	1.916448
O	5.917695	11.835390	1.972576	1.922736
O	5.917695	13.807955	0.000000	1.520531
O	5.917695	13.807955	3.945120	1.918931
O	5.917695	0.000000	1.972576	1.922794
O	5.917695	1.972565	0.000000	1.520475
O	7.883806	1.974127	9.802560	1.982505
O	7.884358	5.919668	9.803296	1.982329
O	7.885194	13.809344	9.798304	1.983434
O	7.884279	9.863377	9.802752	1.981743
O	7.888303	1.972313	5.900384	1.928750
O	7.888493	5.919147	5.901568	1.928528
O	7.888382	13.808997	5.900480	1.928651
O	7.888840	9.862667	5.901984	1.928693
O	7.890260	1.972565	1.972576	1.922721
O	7.890260	5.917695	1.972576	1.922792
O	7.890260	9.862825	1.972576	1.922774
O	7.890260	13.807955	1.972576	1.922716
O	9.843510	9.874834	11.802432	1.966161
O	9.852505	13.804909	11.793216	1.962989
O	9.853073	1.987241	11.791392	1.959354
O	9.853436	5.909110	11.793792	1.968217
O	9.857034	3.947087	9.793504	1.981931
O	9.856576	0.001499	9.808096	1.982052
O	9.857018	11.836416	9.799648	1.981397
O	9.856497	7.892643	9.813696	1.982105
O	9.858833	5.927447	7.861216	1.908702
O	9.857854	9.859527	7.863840	1.907122
O	9.857712	1.968288	7.859968	1.908511
O	9.858927	13.811411	7.859936	1.908623
O	9.861705	7.891680	5.898880	1.929232
O	9.859558	3.946250	5.905056	1.927926
O	9.859622	11.835122	5.903840	1.927666
O	9.861973	-0.000032	5.899008	1.929216
O	9.862825	1.972565	3.945120	1.918457
O	9.862825	5.917695	3.945120	1.918279
O	9.862825	5.917695	0.000000	1.520425
O	9.862825	7.890260	1.972576	1.922643
O	9.862825	9.862825	0.000000	1.520389
O	9.862825	9.862825	3.945120	1.919251
O	9.862825	11.835390	1.972576	1.922849
O	9.862825	13.807955	0.000000	1.520502
O	9.862825	13.807955	3.945120	1.918791
O	9.862825	0.000000	1.972576	1.922622
O	9.862825	1.972565	0.000000	1.520451
O	9.862825	3.945130	1.972576	1.922817
O	11.827358	9.863220	9.805024	1.983224

O	11.828873	13.809754	9.798816	1.983242
O	11.829614	5.919115	9.803104	1.981995
O	11.829346	1.973180	9.801536	1.981904
O	11.832597	9.864592	5.901408	1.928471
O	11.832881	5.917253	5.900512	1.928735
O	11.833212	13.806519	5.900192	1.929008
O	11.833039	1.974396	5.900864	1.928908
O	11.835390	1.972565	1.972576	1.922783
O	11.835390	5.917695	1.972576	1.922766
O	11.835390	9.862825	1.972576	1.922727
O	11.835390	13.807955	1.972576	1.922735
O	13.801469	5.919999	11.792384	1.950019
O	13.795220	9.864245	11.791008	1.966585
O	13.799102	1.973133	11.792064	1.953482
O	13.792632	13.809091	11.788736	1.955019
O	13.802779	7.890812	9.800800	1.982603
O	13.803521	11.835753	9.798784	1.983571
O	13.802921	0.001452	9.799840	1.982565
O	13.801627	3.946029	9.802112	1.982731
O	13.800270	9.862336	7.860032	1.909305
O	13.802432	5.917553	7.859520	1.907930
O	13.803678	1.974664	7.859456	1.907785
O	13.803521	13.808870	7.858176	1.909394
O	13.804483	7.890749	5.900480	1.928616
O	13.806992	3.945635	5.899552	1.929241
O	13.807008	11.835201	5.900672	1.928577
O	13.804546	0.000442	5.900640	1.928737
O	13.807955	5.917695	3.945120	1.918216
O	13.807955	7.890260	1.972576	1.922754
O	13.807955	9.862825	0.000000	1.520437
O	13.807955	9.862825	3.945120	1.919193
O	13.807955	11.835390	1.972576	1.922717
O	13.807955	13.807955	0.000000	1.520541
O	13.807955	13.807955	3.945120	1.918769
O	13.807955	0.000000	1.972576	1.922774
O	13.807955	1.972565	3.945120	1.918328
O	13.807955	1.972565	0.000000	1.520506
O	13.807955	3.945130	1.972576	1.922683
O	13.807955	5.917695	0.000000	1.520505
O	-0.006296	9.862778	9.800800	1.981445
O	-0.005681	5.917853	9.799616	1.982109
O	-0.005839	1.974206	9.801152	1.982180
O	-0.005965	13.809028	9.800992	1.982620
O	-0.002020	5.919257	5.901408	1.928684
O	-0.002351	9.862068	5.902432	1.927781
O	-0.001941	1.971902	5.900256	1.928833
O	-0.001783	13.809170	5.900672	1.928763
Pd	1.933414	0.014045	16.105696	2.981579
Pd	1.932625	3.922785	16.165792	2.944162
Pd	1.927180	7.945176	16.112512	2.974276
Pd	1.932167	11.815112	16.141312	2.947709
Pd	1.945785	1.962418	13.964512	3.083684
Pd	1.924766	13.826829	13.958464	3.098653
Pd	1.922841	5.939677	13.965856	3.094879
Pd	1.940026	9.864371	13.956992	3.103807
Pd	3.885259	11.855936	14.386752	3.488910
Pd	3.878994	3.977385	14.397920	3.494674
Pd	3.933452	15.760936	14.393856	3.493336
Pd	3.917372	7.892974	14.397952	3.493475
Pd	3.889156	13.808018	16.153472	2.953161
Pd	3.863971	5.933523	16.159328	2.946547
Pd	3.950085	9.882614	16.126080	2.947690
Pd	3.926146	1.975705	16.116256	2.962401
Pd	5.907911	11.895782	16.072960	2.961280
Pd	5.863694	3.987516	16.124064	2.968487
Pd	5.886434	15.744162	16.159296	2.928653
Pd	5.874677	7.853902	16.154080	2.943454
Pd	5.894403	13.806124	13.952640	3.074553
Pd	5.847835	2.002485	13.963744	3.108985
Pd	5.880816	5.924859	13.966688	3.084160
Pd	5.874519	9.878542	13.984320	3.094100
Pd	7.930832	11.782841	14.386080	3.397993

188	Pd	7.820131	0.066436	14.382720	3.484003
189	Pd	7.835044	7.951094	14.408512	3.485230
190	Pd	7.846485	3.933673	14.391296	3.488374
191	Pd	7.863654	9.858470	16.225952	2.926472
192	Pd	7.804477	1.994074	16.166048	2.949690
193	Pd	7.912463	13.810985	16.067360	2.996199
194	Pd	7.883001	5.933476	16.104000	2.997744
195	Pd	9.818545	3.920938	16.140832	2.952029
196	Pd	9.815625	0.076756	16.114368	2.981499
197	Pd	9.844551	11.832423	16.301248	3.422964
198	Pd	9.854414	7.870219	16.109024	3.093122
199	Pd	9.809140	5.922666	13.951296	3.085224
200	Pd	9.835036	1.983359	13.958656	3.105252
201	Pd	9.816588	13.873239	13.950272	3.100723
202	Pd	9.879742	9.815831	13.949088	3.128064
203	Pd	11.794945	3.971405	14.395200	3.484495
204	Pd	11.817227	0.026495	14.389696	3.490503
205	Pd	11.841466	7.833387	14.379072	3.487911
206	Pd	11.714937	11.867851	14.368192	3.433940
207	Pd	11.728335	13.825361	16.125920	2.979438
208	Pd	11.778959	5.904755	16.172800	2.957041
209	Pd	11.795197	9.859322	16.076192	3.032083
210	Pd	11.836984	1.994437	16.125760	2.980651
211	Pd	13.787093	3.974797	16.128320	2.980808
212	Pd	13.725644	11.870439	16.074624	2.988513
213	Pd	13.765569	7.872333	16.166624	2.936234
214	Pd	13.762775	15.753204	16.166272	2.922332
215	Pd	13.803268	5.901867	13.969184	3.114867
216	Pd	13.782627	1.997782	13.965600	3.102716
217	Pd	13.776536	13.819112	13.961344	3.082087
218	Pd	13.760140	9.873903	13.950496	3.080929
219	Pd	15.710360	0.009500	14.394336	3.499188
220	Pd	-0.013524	3.941769	14.394592	3.502540
221	Pd	15.716214	7.920117	14.398976	3.494739
222	Pd	15.751626	11.829756	14.392576	3.490305
223	Pd	-0.003819	5.928489	16.127168	2.968959
224	Pd	15.691644	9.881556	16.156512	2.939728
225	Pd	15.754230	13.808933	16.124608	2.965025
226	Pd	15.729707	1.973307	16.147392	2.948161
227	Sr	0.000000	0.000000	0.000000	1.317763
228	Sr	0.000000	0.000000	3.945120	1.574789
229	Sr	0.000000	3.945130	0.000000	1.317717
230	Sr	0.000000	3.945130	3.945120	1.574669
231	Sr	0.000000	7.890260	0.000000	1.317732
232	Sr	0.000000	7.890260	3.945120	1.574719
233	Sr	0.000000	11.835390	0.000000	1.317765
234	Sr	0.000000	11.835390	3.945120	1.574704
235	Sr	3.942021	7.890039	11.548736	1.821401
236	Sr	3.943599	3.943694	11.547360	1.821510
237	Sr	3.941532	0.003014	11.544096	1.823948
238	Sr	3.942495	11.835879	11.543264	1.825539
239	Sr	3.942889	3.946203	7.811200	1.577983
240	Sr	3.942700	0.000410	7.810208	1.578381
241	Sr	3.943536	7.890386	7.812320	1.577761
242	Sr	3.943315	11.834948	7.810912	1.577844
243	Sr	3.945130	0.000000	0.000000	1.317715
244	Sr	3.945130	0.000000	3.945120	1.574755
245	Sr	3.945130	3.945130	0.000000	1.317669
246	Sr	3.945130	3.945130	3.945120	1.574697
247	Sr	3.945130	7.890260	0.000000	1.317696
248	Sr	3.945130	7.890260	3.945120	1.574611
249	Sr	3.945130	11.835390	0.000000	1.317733
250	Sr	3.945130	11.835390	3.945120	1.574784
251	Sr	7.882386	11.839809	11.549344	1.824434
252	Sr	7.883459	7.888982	11.553056	1.817108
253	Sr	7.884248	0.000095	11.541792	1.829060
254	Sr	7.889029	3.944672	11.544256	1.825657
255	Sr	7.886141	7.890576	7.812768	1.578172
256	Sr	7.886378	11.835295	7.811712	1.578296
257	Sr	7.887404	0.000836	7.810016	1.578300
258	Sr	7.886867	3.945714	7.810816	1.578200
259	Sr	7.890260	0.000000	0.000000	1.317794

260	Sr	7.890260	0.000000	3.945120	1.574913
261	Sr	7.890260	3.945130	0.000000	1.317716
262	Sr	7.890260	3.945130	3.945120	1.574626
263	Sr	7.890260	7.890260	0.000000	1.317754
264	Sr	7.890260	7.890260	3.945120	1.574707
265	Sr	7.890260	11.835390	0.000000	1.317816
266	Sr	7.890260	11.835390	3.945120	1.574683
267	Sr	11.835311	7.891570	11.538848	1.830497
268	Sr	11.831319	3.944593	11.545472	1.823157
269	Sr	11.835500	-0.000458	11.543808	1.824527
270	Sr	11.840093	11.837315	11.537344	1.834204
271	Sr	11.833291	3.945682	7.811232	1.577757
272	Sr	11.832976	0.000568	7.810112	1.578381
273	Sr	11.833433	7.889818	7.809600	1.578166
274	Sr	11.833149	11.835958	7.808928	1.578232
275	Sr	11.835390	0.000000	0.000000	1.317826
276	Sr	11.835390	0.000000	3.945120	1.574761
277	Sr	11.835390	3.945130	0.000000	1.317746
278	Sr	11.835390	3.945130	3.945120	1.574588
279	Sr	11.835390	7.890260	0.000000	1.317786
280	Sr	11.835390	7.890260	3.945120	1.574839
281	Sr	11.835390	11.835390	0.000000	1.317852
282	Sr	11.835390	11.835390	3.945120	1.574810
283	Sr	-0.001878	7.888224	11.546432	1.821508
284	Sr	-0.000694	0.001515	11.544160	1.823183
285	Sr	-0.002840	3.945114	11.545248	1.823950
286	Sr	-0.002825	11.836226	11.543520	1.823943
287	Sr	-0.002146	3.945777	7.810688	1.578257
288	Sr	-0.002067	7.888824	7.811008	1.577797
289	Sr	-0.002115	0.000757	7.810048	1.578171
290	Sr	-0.001767	11.835990	7.810080	1.578037
291	Ti	1.969519	5.917616	9.840864	2.649610
292	Ti	1.968967	1.973607	9.838912	2.649628
293	Ti	1.969283	9.862667	9.838880	2.649707
294	Ti	1.969156	13.808634	9.838752	2.649647
295	Ti	1.971350	1.972849	5.898656	2.621736
296	Ti	1.971618	13.807908	5.898528	2.621253
297	Ti	1.971713	5.917869	5.899008	2.621241
298	Ti	1.972170	9.862541	5.898144	2.620742
299	Ti	1.972565	1.972565	1.972576	2.637309
300	Ti	1.972565	5.917695	1.972576	2.637313
301	Ti	1.972565	9.862825	1.972576	2.637287
302	Ti	1.972565	13.807955	1.972576	2.637298
303	Ti	5.914776	9.863283	9.844704	2.647533
304	Ti	5.913971	5.918516	9.841760	2.649174
305	Ti	5.913955	13.808949	9.837184	2.651824
306	Ti	5.913434	1.973827	9.838624	2.649836
307	Ti	5.916527	9.862588	5.900448	2.621486
308	Ti	5.916322	5.917979	5.899136	2.621387
309	Ti	5.916464	13.807592	5.897952	2.621995
310	Ti	5.916417	1.972912	5.898336	2.621775
311	Ti	5.917695	1.972565	1.972576	2.637324
312	Ti	5.917695	5.917695	1.972576	2.637332
313	Ti	5.917695	9.862825	1.972576	2.637301
314	Ti	5.917695	13.807955	1.972576	2.637316
315	Ti	9.858422	5.918594	9.838848	2.649659
316	Ti	9.858706	13.808160	9.837568	2.649136
317	Ti	9.859164	1.973670	9.838624	2.650060
318	Ti	9.857097	9.864466	9.837920	2.647969
319	Ti	9.862825	5.917695	1.972576	2.637283
320	Ti	9.862825	9.862825	1.972576	2.637249
321	Ti	9.862825	13.807955	1.972576	2.637276
322	Ti	9.862825	1.972565	1.972576	2.637289
323	Ti	9.861610	1.972313	5.898496	2.621024
324	Ti	9.861421	13.808255	5.898176	2.621321
325	Ti	9.861294	5.918184	5.898656	2.620984
326	Ti	9.861168	9.862241	5.897568	2.620412
327	Ti	13.804483	1.973259	9.839392	2.649577
328	Ti	13.804783	5.916969	9.840032	2.649563
329	Ti	13.805067	13.809470	9.837216	2.649922
330	Ti	13.805051	9.862604	9.836352	2.649763
331	Ti	13.806787	1.972896	5.898592	2.621459

```
Ti      13.806819      13.808271       5.898144        2.621962
Ti      13.806708       5.917269       5.898656        2.621527
Ti      13.807955       5.917695       1.972576        2.637323
Ti      13.807955       9.862825       1.972576        2.637277
Ti      13.807955      13.807955       1.972576        2.637314
Ti      13.807955       1.972565       1.972576        2.637318
Ti      13.807071       9.862699       5.897664        2.621216
C        9.549850      11.450124      18.262144        3.920140
H        8.928129      12.259933      18.662720        0.990066
H        9.471563       9.494445      18.192704        0.961158
H       10.574968      11.425570      18.661376        1.007127
O        8.862608      10.231821      18.451808        2.385337
```

1.1.9 CH_3OH

```
      342
jmolscript: load "" {1 1 1} spacegroup "x,y,z" unitcell [{      15.780520
       0.000000      0.000000 }, {       0.000000     15.780520      0.000000
   }, {       0.000000      0.000000     32.000000 }]
O        1.972565       1.972565       3.945120        1.917839
O        1.972234       1.970971       7.862656        1.904713
O        1.975532       1.968872      11.796960        1.955648
O        1.972565       1.972565       0.000000        1.519234
O        1.972565       3.945130       1.972576        1.922904
O        2.103954       3.944436       5.902752        1.922171
O        1.952161       3.943126       9.807168        1.984009
O        1.972565       5.917695       3.945120        1.917652
O        1.973354       5.916433       7.862880        1.904378
O        1.971097       5.913561      11.798144        1.953176
O        1.972565       5.917695       0.000000        1.519151
O        1.972565       7.890260       1.972576        1.922953
O        1.841192       7.889645       5.902656        1.922503
O        1.992922       7.888540       9.808096        1.984217
O        1.971681       9.861168       7.863648        1.904179
O        1.976147       9.858485      11.799744        1.955271
O        1.972565       9.862825       0.000000        1.519126
O        1.972565       9.862825       3.945120        1.917373
O        1.972565       0.000000       1.972576        1.922955
O        1.841839      -0.000978       5.902848        1.922448
O        1.993427      -0.002004       9.807136        1.983848
O        1.972565      11.835390       1.972576        1.922955
O        2.103827      11.834301       5.902848        1.922837
O        1.951735      11.833228       9.808256        1.984216
O        1.972060      13.804373      11.797792        1.953788
O        1.972565      13.807955       0.000000        1.519235
O        1.972565      13.807955       3.945120        1.917851
O        1.972960      13.805825       7.862688        1.904731
O        3.945130       1.972565       1.972576        1.922936
O        3.945288       1.840892       5.902656        1.922695
O        3.945461       1.991707       9.806112        1.984297
O        3.945335       6.048563       5.902560        1.922584
O        3.945446       5.895697       9.808736        1.983826
O        3.945130       5.917695       1.972576        1.922942
O        3.945114       9.730521       5.903456        1.922446
O        3.945761       9.881919       9.807616        1.984169
O        3.945130       9.862825       1.972576        1.922950
O        3.945067      13.785657       9.807008        1.984007
O        3.945130      13.807955       1.972576        1.922922
O        3.945193      13.938081       5.902272        1.922731
O        5.917695       1.972565       3.945120        1.917855
O        5.918484       1.971981       7.862112        1.905268
O        5.918863       1.968793      11.796960        1.953289
O        5.917695       3.945130       1.972576        1.922927
O        5.786890       3.944657       5.902528        1.922778
O        5.938478       3.943441       9.806912        1.983891
O        5.917695       5.917695       3.945120        1.917326
O        5.917805       5.916969       7.863168        1.904148
```

O	5.920393	5.912124	11.797824	1.956357
O	5.917695	5.917695	0.000000	1.519153
O	5.917695	7.890260	1.972576	1.922966
O	6.049478	7.889629	5.902464	1.922462
O	5.897307	7.888603	9.809120	1.983993
O	5.917963	9.861389	7.863488	1.903729
O	5.918326	9.859590	11.800416	1.947696
O	5.917695	9.862825	0.000000	1.519175
O	5.917695	9.862825	3.945120	1.917129
O	6.048926	-0.000710	5.902720	1.922533
O	5.897070	-0.001278	9.805120	1.984113
O	5.917695	11.835390	1.972576	1.922952
O	5.786496	11.834412	5.902400	1.922618
O	5.938699	11.833512	9.808992	1.983433
O	5.917158	13.808081	11.796352	1.955842
O	5.917695	13.807955	0.000000	1.519297
O	5.917695	13.807955	3.945120	1.917321
O	5.917443	13.805430	7.862528	1.904934
O	5.917695	1.972565	0.000000	1.519252
O	5.917695	0.000000	1.972576	1.922978
O	7.890260	1.972565	1.972576	1.922910
O	7.890718	2.103228	5.902208	1.922687
O	7.890954	1.950819	9.806528	1.984168
O	7.890797	5.785928	5.903264	1.922374
O	7.891128	5.937152	9.807872	1.983653
O	7.890260	5.917695	1.972576	1.922972
O	7.890686	9.993488	5.903008	1.922578
O	7.889692	9.840464	9.808096	1.983626
O	7.890260	9.862825	1.972576	1.922962
O	7.890213	13.827365	9.806464	1.984091
O	7.890260	13.807955	1.972576	1.922955
O	7.890591	13.675967	5.902560	1.922488
O	9.862825	1.972565	3.945120	1.917486
O	9.863535	1.972107	7.862528	1.905148
O	9.863772	1.967168	11.797408	1.953940
O	9.994419	3.944925	5.903264	1.922598
O	9.842594	3.943994	9.808160	1.983631
O	9.862825	5.917695	3.945120	1.917516
O	9.863930	5.918121	7.864096	1.904101
O	9.864151	5.914618	11.799264	1.957443
O	9.862825	5.917695	0.000000	1.518941
O	9.862825	7.890260	1.972576	1.922991
O	9.731784	7.889755	5.902784	1.922473
O	9.882866	7.889581	9.811648	1.983552
O	9.862825	9.861073	7.864416	1.903459
O	9.855140	9.865729	11.802560	1.948566
O	9.862825	9.862825	0.000000	1.518914
O	9.862825	9.862825	3.945120	1.917190
O	9.883434	-0.001310	9.805312	1.984163
O	9.862825	11.835390	1.972576	1.922983
O	9.994482	11.834396	5.902752	1.922588
O	9.841490	11.834001	9.807808	1.984346
O	9.862273	13.807482	11.796608	1.956116
O	9.862825	13.807955	0.000000	1.519127
O	9.862825	13.807955	3.945120	1.917373
O	9.863598	13.806093	7.862784	1.905250
O	9.862825	1.972565	0.000000	1.519133
O	9.862825	3.945130	1.972576	1.922953
O	9.862825	0.000000	1.972576	1.922984
O	9.732036	-0.000600	5.903008	1.922589
O	0.000000	1.972565	1.972576	1.922987
O	0.000079	2.102896	5.902304	1.922541
O	0.000458	1.950236	9.808064	1.983670
O	0.000063	5.785738	5.902880	1.922724
O	0.000300	5.936947	9.806496	1.984249
O	0.000000	5.917695	1.972576	1.922986
O	-0.000252	9.993219	5.903584	1.922010
O	0.000079	9.840748	9.810816	1.983197
O	0.000000	9.862825	1.972576	1.922974
O	0.000316	13.826734	9.806208	1.984307
O	0.000000	13.807955	1.972576	1.922972
O	-0.000000	13.676030	5.902816	1.922533

123	O	11.835390	1.972565	1.972576	1.922968
124	O	11.835642	1.840766	5.902400	1.922738
125	O	11.835753	1.991801	9.806784	1.984207
126	O	11.835579	6.048452	5.902752	1.922460
127	O	11.835816	5.896170	9.808768	1.983933
128	O	11.835390	5.917695	1.972576	1.922972
129	O	11.835469	9.730632	5.903456	1.922539
130	O	11.834159	9.881809	9.811264	1.983982
131	O	11.835390	9.862825	1.972576	1.922999
132	O	11.835674	13.785799	9.808288	1.983826
133	O	11.835390	13.807955	1.972576	1.922966
134	O	11.835469	13.938413	5.902368	1.922632
135	O	13.808776	1.971350	7.862816	1.904599
136	O	13.807718	1.968746	11.797376	1.954035
137	O	13.676945	3.944499	5.902784	1.922582
138	O	13.828691	3.943457	9.807424	1.983869
139	O	13.807955	5.917695	3.945120	1.917429
140	O	13.807545	5.916575	7.863200	1.904448
141	O	13.810369	5.914428	11.798176	1.953072
142	O	13.807955	7.890260	1.972576	1.922990
143	O	13.939423	7.889771	5.903200	1.922254
144	O	13.787156	7.888871	9.808128	1.984142
145	O	13.807497	9.861783	7.864832	1.903847
146	O	13.806614	9.858075	11.800192	1.958742
147	O	13.807955	9.862825	0.000000	1.518886
148	O	13.807955	9.862825	3.945120	1.917570
149	O	13.787598	-0.002020	9.806688	1.984018
150	O	13.807955	11.835390	1.972576	1.922966
151	O	13.676724	11.834301	5.902560	1.922510
152	O	13.827996	11.833197	9.809312	1.983895
153	O	13.811995	13.805225	11.798144	1.953218
154	O	13.807955	13.807955	0.000000	1.519087
155	O	13.807955	13.807955	3.945120	1.917480
156	O	13.807750	13.805209	7.863232	1.904475
157	O	13.807955	1.972565	3.945120	1.917556
158	O	13.807955	1.972565	0.000000	1.519127
159	O	13.807955	3.945130	1.972576	1.922920
160	O	13.807955	5.917695	0.000000	1.518969
161	O	13.807955	0.000000	1.972576	1.922994
162	O	13.939186	-0.000915	5.903200	1.922657
163	Pd	1.981276	1.966095	13.966208	3.094519
164	Pd	1.975090	3.893465	16.133312	2.968468
165	Pd	1.966221	5.901346	13.969312	3.099958
166	Pd	1.977441	7.916629	16.147872	2.951222
167	Pd	1.980724	9.852852	13.967552	3.096796
168	Pd	1.976652	0.033613	16.146880	2.955730
169	Pd	1.980234	11.784277	16.137056	2.960079
170	Pd	1.968604	13.794699	13.968288	3.097170
171	Pd	3.984739	1.962245	16.127776	2.969438
172	Pd	3.955971	3.948002	14.399136	3.488919
173	Pd	3.900203	5.901015	16.159072	2.951888
174	Pd	3.939496	7.859330	14.398368	3.489910
175	Pd	3.997853	9.847518	16.129216	2.958109
176	Pd	3.943789	15.755034	14.399136	3.488085
177	Pd	3.951647	11.832581	14.396608	3.487461
178	Pd	3.900455	13.797066	16.154368	2.954569
179	Pd	5.914933	1.959814	13.968096	3.091922
180	Pd	5.924386	3.972020	16.143936	2.943598
181	Pd	5.931708	5.912298	13.966528	3.091867
182	Pd	5.913087	7.812951	16.133760	2.978836
183	Pd	5.908747	9.843510	13.975424	3.097364
184	Pd	5.921230	15.735293	16.134240	2.957998
185	Pd	5.912330	11.885209	16.145984	2.960474
186	Pd	5.924780	13.799165	13.966528	3.091934
187	Pd	7.859093	1.958915	16.149888	2.946782
188	Pd	7.892248	3.924347	14.398944	3.491429
189	Pd	7.939259	5.911730	16.125216	2.954194
190	Pd	7.906561	7.909654	14.408448	3.479517
191	Pd	7.815460	9.840543	16.193280	2.957672
192	Pd	7.898213	-0.004703	14.396704	3.485471
193	Pd	7.884721	11.793651	14.406400	3.465411
194	Pd	7.923667	13.789571	16.126112	2.975471

195	Pd	9.872136	1.964975	13.967936	3.095104
196	Pd	9.866612	3.895264	16.139680	2.956661
197	Pd	9.860632	5.902546	13.965376	3.094034
198	Pd	9.883450	7.914294	16.145152	2.928937
199	Pd	9.893139	9.850642	13.974016	3.087096
200	Pd	9.865886	0.018384	16.142720	2.951602
201	Pd	9.883955	11.820225	16.118496	2.951884
202	Pd	9.861973	13.791433	13.965216	3.090326
203	Pd	15.743483	1.961108	16.153568	2.951013
204	Pd	-0.003835	3.921585	14.397504	3.490358
205	Pd	0.037431	5.901694	16.134112	2.956193
206	Pd	0.011346	7.885999	14.404032	3.495394
207	Pd	15.748375	9.849490	16.157920	2.947457
208	Pd	0.010762	-0.000426	14.398976	3.492444
209	Pd	0.002509	11.811498	14.402752	3.494955
210	Pd	0.041629	13.797114	16.130944	2.957568
211	Pd	11.872458	1.959041	16.127616	2.964425
212	Pd	11.841655	3.943726	14.400640	3.491253
213	Pd	11.802567	5.902672	16.155872	2.931122
214	Pd	11.845300	7.861776	14.396256	3.489091
215	Pd	11.868498	9.850863	16.129376	2.994716
216	Pd	11.835532	15.755697	14.397344	3.486916
217	Pd	11.860607	11.839446	14.392448	3.486071
218	Pd	11.814481	13.800238	16.155520	2.954471
219	Pd	13.804783	1.957526	13.967584	3.096530
220	Pd	13.811111	3.970016	16.146400	2.953354
221	Pd	13.817928	5.909726	13.969504	3.098501
222	Pd	13.812168	7.848836	16.138240	2.945063
223	Pd	13.815624	9.849822	13.963616	3.095367
224	Pd	13.814693	15.735782	16.135552	2.967328
225	Pd	13.814346	11.854390	16.149536	2.938001
226	Pd	13.823215	13.801106	13.968960	3.100063
227	Sr	0.000000	0.000000	0.000000	1.316366
228	Sr	3.945130	0.000000	0.000000	1.316462
229	Sr	3.945130	3.945130	0.000000	1.316369
230	Sr	3.945130	3.945130	3.945120	1.581878
231	Sr	3.945461	3.944878	7.820384	1.585664
232	Sr	3.944988	3.943457	11.550080	1.823998
233	Sr	3.945130	7.890260	0.000000	1.316312
234	Sr	3.945130	7.890260	3.945120	1.581790
235	Sr	3.945240	7.889850	7.820896	1.585359
236	Sr	3.944814	7.890733	11.550240	1.824706
237	Sr	3.945130	0.000000	3.945120	1.581861
238	Sr	3.945430	-0.000805	7.819872	1.586015
239	Sr	3.945887	-0.000047	11.550048	1.824363
240	Sr	3.945130	11.835390	0.000000	1.316402
241	Sr	3.945130	11.835390	3.945120	1.581788
242	Sr	3.945525	11.834727	7.820256	1.585627
243	Sr	3.944562	11.834822	11.549856	1.825616
244	Sr	7.890260	0.000000	0.000000	1.316380
245	Sr	7.890260	3.945130	0.000000	1.316232
246	Sr	7.890260	3.945130	3.945120	1.581806
247	Sr	7.890292	3.944641	7.820064	1.585716
248	Sr	7.889850	3.943757	11.550048	1.824491
249	Sr	7.890260	7.890260	0.000000	1.316111
250	Sr	7.890260	7.890260	3.945120	1.581780
251	Sr	7.890165	7.890102	7.821568	1.585068
252	Sr	7.888792	7.889061	11.555872	1.819510
253	Sr	7.890260	0.000000	3.945120	1.581724
254	Sr	7.889929	-0.000584	7.819712	1.586306
255	Sr	7.890071	0.000221	11.549376	1.825479
256	Sr	7.890260	11.835390	0.000000	1.316253
257	Sr	7.890260	11.835390	3.945120	1.581758
258	Sr	7.889992	11.834901	7.821600	1.585635
259	Sr	7.890276	11.836337	11.556032	1.820142
260	Sr	0.000000	3.945130	0.000000	1.316281
261	Sr	0.000000	3.945130	3.945120	1.581831
262	Sr	0.000379	3.944657	7.819872	1.585762
263	Sr	0.000300	3.944878	11.549280	1.825215
264	Sr	0.000000	7.890260	0.000000	1.316144
265	Sr	0.000000	7.890260	3.945120	1.581759
266	Sr	0.000110	7.889566	7.821056	1.585419

Sr	-0.000552	7.888003	11.551776	1.822296
Sr	0.000000	0.000000	3.945120	1.581791
Sr	0.000363	-0.000663	7.819872	1.585929
Sr	-0.000410	-0.001499	11.549664	1.824256
Sr	0.000000	11.835390	0.000000	1.316222
Sr	0.000000	11.835390	3.945120	1.581790
Sr	0.000426	11.835106	7.820608	1.585577
Sr	-0.000268	11.836668	11.551520	1.822634
Sr	11.835390	0.000000	0.000000	1.316260
Sr	11.835390	3.945130	0.000000	1.316122
Sr	11.835390	3.945130	3.945120	1.581765
Sr	11.835516	3.944909	7.820448	1.585644
Sr	11.835785	3.943804	11.550368	1.822938
Sr	11.835390	7.890260	0.000000	1.315926
Sr	11.835390	7.890260	3.945120	1.581765
Sr	11.834948	7.889708	7.820672	1.585078
Sr	11.835390	7.889929	11.549888	1.825787
Sr	11.835390	0.000000	3.945120	1.581818
Sr	11.835201	-0.000615	7.819584	1.585984
Sr	11.834901	0.000284	11.549152	1.824933
Sr	11.835390	11.835390	0.000000	1.316062
Sr	11.835390	11.835390	3.945120	1.581812
Sr	11.835153	11.834759	7.820416	1.585443
Sr	11.833102	11.835595	11.548512	1.827293
Ti	1.972565	1.972565	1.972576	2.637159
Ti	1.972691	1.972486	5.898912	2.609653
Ti	1.972975	1.971366	9.842400	2.646691
Ti	1.972533	5.917900	5.899072	2.609389
Ti	1.972691	5.916590	9.843328	2.646728
Ti	1.972565	5.917695	1.972576	2.637169
Ti	1.972802	9.862667	5.899264	2.609276
Ti	1.972297	9.861720	9.843488	2.646627
Ti	1.972565	9.862825	1.972576	2.637158
Ti	1.972723	13.806992	9.842752	2.646605
Ti	1.972565	13.807955	1.972576	2.637156
Ti	1.972644	13.807624	5.898912	2.609676
Ti	5.917695	1.972565	1.972576	2.637166
Ti	5.917648	1.972675	5.898880	2.609769
Ti	5.917884	1.971602	9.842112	2.646524
Ti	5.917490	5.917742	5.899328	2.609584
Ti	5.917979	5.916843	9.843808	2.647229
Ti	5.917695	5.917695	1.972576	2.637173
Ti	5.917979	9.862667	5.899584	2.609491
Ti	5.918074	9.862115	9.844896	2.646770
Ti	5.917695	9.862825	1.972576	2.637165
Ti	5.917758	13.807008	9.843360	2.647223
Ti	5.917695	13.807955	1.972576	2.637163
Ti	5.917632	13.807781	5.899328	2.609722
Ti	9.862857	1.972502	5.899136	2.609676
Ti	9.862983	1.971508	9.842208	2.646516
Ti	9.862762	5.917695	5.899168	2.609097
Ti	9.862383	5.917095	9.843552	2.647166
Ti	9.862825	5.917695	1.972576	2.637190
Ti	9.862573	9.862683	5.899424	2.609260
Ti	9.861073	9.862336	9.844768	2.646531
Ti	9.862825	9.862825	1.972576	2.637186
Ti	9.861989	13.807276	9.842336	2.646957
Ti	9.862825	13.807955	1.972576	2.637178
Ti	9.862730	13.807639	5.899264	2.609583
Ti	9.862825	1.972565	1.972576	2.637181
Ti	13.807955	1.972612	5.899040	2.609351
Ti	13.808239	1.971397	9.842528	2.646443
Ti	13.808113	5.917679	5.899232	2.609505
Ti	13.807987	5.916622	9.843136	2.646670
Ti	13.807955	5.917695	1.972576	2.637187
Ti	13.807781	9.862809	5.899040	2.608815
Ti	13.807671	9.861878	9.843296	2.646691
Ti	13.807955	9.862825	1.972576	2.637180
Ti	13.807892	13.807040	9.843104	2.646253
Ti	13.807955	13.807955	1.972576	2.637179
Ti	13.807923	13.807939	5.899168	2.609369
Ti	13.807955	1.972565	1.972576	2.637168

```
C        8.959438     10.340170     18.967840       3.955985
H        8.186271      9.878716     18.284832       1.130269
H        8.483529     10.447541     19.952384       0.958751
H       10.536732      9.443331     18.240128       1.079917
H        9.202631     11.347677     18.572672       1.059852
O       10.096598      9.515480     19.122976       2.268446
```

1.1.10 $CH_2O(OH)$

```
       342
jmolscript: load "" {1 1 1} spacegroup "x,y,z" unitcell [{      15.780520
       0.000000      0.000000 }, {      0.000000     15.780520      0.000000
    }, {      0.000000      0.000000     32.000000 }]
O        1.972565      1.972565      3.945120       1.917339
O        1.970987      1.974096      7.858688       1.908240
O        1.964485      1.971003     11.790304       1.949115
O        1.972565      1.972565      0.000000       1.521570
O        1.972565      3.945130      1.972576       1.922633
O        1.971050      3.946077      5.900096       1.928841
O        1.970182      3.945872      9.802880       1.982325
O        1.972565      5.917695      3.945120       1.916607
O        1.979714      5.918153      7.860448       1.908359
O        1.945265      5.921261     11.791232       1.954461
O        1.972565      5.917695      0.000000       1.521661
O        1.972565      7.890260      1.972576       1.922694
O        1.971650      7.890591      5.900672       1.928137
O        1.970624      7.891065      9.800768       1.983197
O        1.975027      9.863362      7.861376       1.907766
O        1.953755      9.864656     11.793056       1.960377
O        1.972565      9.862825      0.000000       1.521682
O        1.972565      9.862825      3.945120       1.917386
O        1.972565      0.000000      1.972576       1.922658
O        1.971934      0.000221      5.900608       1.928667
O        1.969677      0.000158      9.799104       1.982092
O        1.972565     11.835390      1.972576       1.922671
O        1.971413     11.834711      5.900192       1.928768
O        1.969298     11.835406      9.801600       1.982624
O        1.964217     13.809675     11.790336       1.948957
O        1.972565     13.807955      0.000000       1.521605
O        1.972565     13.807955      3.945120       1.917513
O        1.970166     13.807182      7.858656       1.908332
O        3.945130      1.972565      1.972576       1.922657
O        3.944120      1.973780      5.899616       1.928966
O        3.941658      1.973938      9.802176       1.981990
O        3.945745      5.918484      5.901024       1.928920
O        3.943946      5.917332      9.818016       1.981637
O        3.945130      5.917695      1.972576       1.922551
O        3.945177      9.862541      5.901984       1.927940
O        3.943299      9.862888      9.813600       1.982185
O        3.945130      9.862825      1.972576       1.922588
O        3.941422     13.807482      9.801184       1.982467
O        3.945130     13.807955      1.972576       1.922674
O        3.944515     13.807087      5.900384       1.928564
O        5.917695      1.972565      3.945120       1.915801
O        5.913545      1.980960      7.860128       1.906827
O        5.910026      1.971539     11.793152       1.941865
O        5.917695      3.945130      1.972576       1.922555
O        5.917679      3.947118      5.900736       1.928956
O        5.916275      3.948681      9.814176       1.982632
O        5.917695      5.917695      3.945120       1.915656
O        5.915833      5.917979      7.867584       1.902291
O        5.926169      5.932355     11.811296       1.945139
O        5.917695      5.917695      0.000000       1.521846
O        5.917695      7.890260      1.972576       1.922775
O        5.916306      7.890781      5.904224       1.927250
O        5.915975      7.890213      9.808416       1.982101
O        5.915107      9.863109      7.866912       1.905313
```

O	5.924133	9.849459	11.806272	1.969626
O	5.917695	9.862825	0.000000	1.521807
O	5.917695	9.862825	3.945120	1.917949
O	5.915817	0.000000	5.905664	1.927998
O	5.914208	0.001199	9.797248	1.981316
O	5.917695	11.835390	1.972576	1.922584
O	5.917190	11.834033	5.901856	1.927825
O	5.915123	11.833780	9.813792	1.982320
O	5.908527	13.819996	11.793184	1.960860
O	5.917695	13.807955	0.000000	1.521677
O	5.917695	13.807955	3.945120	1.917374
O	5.914697	13.802558	7.861440	1.907826
O	5.917695	1.972565	0.000000	1.521728
O	5.917695	0.000000	1.972576	1.922708
O	7.890260	1.972565	1.972576	1.922700
O	7.889061	1.972297	5.900736	1.928112
O	7.885731	1.973559	9.800096	1.983386
O	7.889203	5.919115	5.904832	1.927409
O	7.887988	5.919178	9.813856	1.978841
O	7.890260	5.917695	1.972576	1.922835
O	7.888429	9.862083	5.904032	1.927295
O	7.886915	9.861200	9.808736	1.981705
O	7.890260	9.862825	1.972576	1.922778
O	7.885968	13.807071	9.801216	1.983060
O	7.890260	13.807955	1.972576	1.922687
O	7.888556	13.808239	5.900352	1.928157
O	9.862825	1.972565	3.945120	1.917191
O	9.860316	1.982917	7.860384	1.909798
O	9.849554	1.956690	11.791936	1.960971
O	9.860048	3.947276	5.900736	1.928724
O	9.858454	3.946834	9.816064	1.982314
O	9.862825	5.917695	3.945120	1.916595
O	9.860821	5.917742	7.868992	1.902656
O	9.849396	5.924102	11.812096	1.963469
O	9.862825	5.917695	0.000000	1.521679
O	9.862825	7.890260	1.972576	1.922833
O	9.860774	7.890102	5.904864	1.927475
O	9.858833	7.889345	9.814208	1.979053
O	9.859764	9.862367	7.867424	1.902602
O	9.842515	9.848859	11.810656	1.947170
O	9.862825	9.862825	0.000000	1.521840
O	9.862825	9.862825	3.945120	1.915807
O	9.859432	0.000615	9.788128	1.981816
O	9.862825	11.835390	1.972576	1.922552
O	9.860000	11.833465	5.900736	1.928673
O	9.859969	11.833449	9.817760	1.981542
O	9.854777	13.830016	11.790496	1.955229
O	9.862825	13.807955	0.000000	1.521647
O	9.862825	13.807955	3.945120	1.916794
O	9.858911	13.797966	7.860064	1.909037
O	9.862825	1.972565	0.000000	1.521540
O	9.862825	3.945130	1.972576	1.922535
O	9.862825	0.000000	1.972576	1.922747
O	9.861436	0.000189	5.907200	1.927090
O	0.000000	1.972565	1.972576	1.922695
O	-0.001625	1.972802	5.901184	1.928410
O	-0.003724	1.973528	9.798336	1.981993
O	-0.001420	5.918484	5.907424	1.927312
O	-0.003929	5.918105	9.789184	1.981489
O	0.000000	5.917695	1.972576	1.922730
O	-0.001262	9.862462	5.905216	1.928001
O	-0.004829	9.862478	9.797376	1.981182
O	0.000000	9.862825	1.972576	1.922669
O	-0.003914	13.807718	9.799136	1.982060
O	0.000000	13.807955	1.972576	1.922620
O	-0.001341	13.808144	5.900256	1.928759
O	11.835390	1.972565	1.972576	1.922674
O	11.833197	1.973749	5.899904	1.928495
O	11.831035	1.973796	9.804704	1.982334
O	11.832029	5.918184	5.900736	1.928421
O	11.830403	5.919036	9.816832	1.982086
O	11.835390	5.917695	1.972576	1.922556

O	11.832108	9.862273	5.900832	1.928829
O	11.828494	9.861389	9.813504	1.982308
O	11.835390	9.862825	1.972576	1.922581
O	11.831319	13.807182	9.801152	1.982601
O	11.835390	13.807955	1.972576	1.922666
O	11.833118	13.807482	5.900192	1.928665
O	13.802905	1.975248	7.859712	1.908144
O	13.806266	1.967657	11.790432	1.953193
O	13.806314	3.945982	5.899808	1.928736
O	13.803821	3.946045	9.805600	1.981699
O	13.807955	5.917695	3.945120	1.917084
O	13.795110	5.917080	7.860768	1.909353
O	13.817707	5.925822	11.792800	1.960421
O	13.807955	7.890260	1.972576	1.922714
O	13.806014	7.890023	5.900608	1.928175
O	13.803615	7.891223	9.799936	1.983434
O	13.797240	9.863646	7.859872	1.907371
O	13.801927	9.863851	11.792864	1.942685
O	13.807955	9.862825	0.000000	1.521723
O	13.807955	9.862825	3.945120	1.915787
O	13.803584	-0.000063	9.798208	1.982118
O	13.807955	11.835390	1.972576	1.922656
O	13.806188	11.835011	5.899488	1.928976
O	13.803757	11.835216	9.801472	1.982153
O	13.802590	13.808823	11.789472	1.949880
O	13.807955	13.807955	0.000000	1.521552
O	13.807955	13.807955	3.945120	1.917573
O	13.804420	13.806756	7.858432	1.908656
O	13.807955	1.972565	3.945120	1.917840
O	13.807955	1.972565	0.000000	1.521436
O	13.807955	3.945130	1.972576	1.922645
O	13.807955	5.917695	0.000000	1.521542
O	13.807955	0.000000	1.972576	1.922692
O	13.805635	0.000473	5.901184	1.928430
Pd	1.920458	1.967389	13.970368	3.104841
Pd	1.912378	3.950054	16.145760	2.983445
Pd	1.939962	5.923802	13.965792	3.096899
Pd	1.933919	7.914073	16.144576	2.971536
Pd	1.936096	9.876144	13.960352	3.100408
Pd	1.946669	-0.004955	16.152288	2.977286
Pd	1.930699	11.837868	16.139744	2.980878
Pd	1.939852	13.816461	13.969920	3.109779
Pd	3.896684	1.971808	16.159488	2.956209
Pd	3.867001	3.922343	14.388256	3.508293
Pd	3.848096	5.909142	16.124896	3.034802
Pd	3.893039	7.909212	14.372128	3.510160
Pd	3.891760	9.897037	16.129408	3.033332
Pd	3.910287	0.016680	14.396352	3.479989
Pd	3.890308	11.866904	14.379360	3.493446
Pd	3.919076	13.824035	16.140064	2.979850
Pd	5.876318	1.995620	13.980000	3.070768
Pd	5.837246	3.954109	16.147520	3.000492
Pd	5.833569	5.884256	13.979552	3.090394
Pd	5.780625	7.884895	16.088992	3.021509
Pd	5.867355	9.890173	13.952032	3.061920
Pd	5.880485	0.020136	16.145440	2.964362
Pd	5.860333	11.862974	16.130496	3.026766
Pd	5.883988	13.823199	13.959712	3.101071
Pd	7.842003	2.003353	16.122208	2.972276
Pd	7.840125	4.017026	14.406336	3.516415
Pd	7.916961	5.776175	16.242496	3.044343
Pd	7.924157	7.828527	14.492960	3.235702
Pd	7.880302	9.970969	16.088128	3.011637
Pd	7.855732	0.015544	14.395392	3.469436
Pd	7.844654	11.862375	14.371872	3.510746
Pd	7.841514	13.824146	16.142144	2.974900
Pd	9.829654	1.996693	13.959168	3.083465
Pd	9.888705	3.873834	16.093888	2.985547
Pd	9.902797	5.849807	13.962528	3.105760
Pd	9.971979	7.836070	16.248256	3.037091
Pd	9.871883	9.925710	13.978400	3.094223
Pd	9.821543	0.023024	16.151744	2.932744

201	Pd	9.845340	11.915113	16.125376	3.034786
202	Pd	9.834246	13.817297	13.964864	3.097159
203	Pd	15.762562	1.968131	16.123904	3.010416
204	Pd	15.731932	3.968438	14.395200	3.473163
205	Pd	15.732610	5.936853	16.153376	2.931415
206	Pd	15.742000	7.900628	14.396928	3.469592
207	Pd	15.738070	9.874187	16.147744	2.962992
208	Pd	-0.009516	-0.017138	14.398048	3.543114
209	Pd	15.735893	11.840676	14.396544	3.480539
210	Pd	15.756960	13.804357	16.150176	2.981111
211	Pd	11.838404	1.949431	16.179392	2.970421
212	Pd	11.888681	3.871877	14.377568	3.509505
213	Pd	11.883899	5.868933	16.097920	2.983097
214	Pd	11.727940	7.911548	14.408480	3.516397
215	Pd	11.794708	9.917126	16.146688	3.000201
216	Pd	11.789847	0.020278	14.394080	3.475163
217	Pd	11.830956	11.887592	14.388544	3.509524
218	Pd	11.812240	13.842514	16.146624	2.985136
219	Pd	13.823846	1.933129	13.965696	3.125114
220	Pd	13.806598	3.920007	16.183680	2.967978
221	Pd	13.758878	5.933333	13.959392	3.084068
222	Pd	13.748999	7.920385	16.118400	2.976486
223	Pd	13.759351	9.878211	13.979456	3.068732
224	Pd	13.787945	-0.005397	16.126048	3.010165
225	Pd	13.780781	11.856015	16.160480	2.955899
226	Pd	13.789823	13.835666	13.969344	3.106241
227	Sr	0.000000	0.000000	0.000000	1.318860
228	Sr	3.945130	0.000000	0.000000	1.319033
229	Sr	3.945130	3.945130	0.000000	1.319151
230	Sr	3.945130	3.945130	3.945120	1.574002
231	Sr	3.945193	3.945225	7.814272	1.576996
232	Sr	3.943710	3.941390	11.546816	1.824601
233	Sr	3.945130	7.890260	0.000000	1.319392
234	Sr	3.945130	7.890260	3.945120	1.574225
235	Sr	3.943962	7.888714	7.811008	1.577541
236	Sr	3.945714	7.889361	11.536672	1.835584
237	Sr	3.945130	0.000000	3.945120	1.574071
238	Sr	3.943031	0.000931	7.811712	1.577966
239	Sr	3.943662	-0.000552	11.546848	1.821712
240	Sr	3.945130	11.835390	0.000000	1.319273
241	Sr	3.945130	11.835390	3.945120	1.574342
242	Sr	3.943568	11.835343	7.811552	1.576748
243	Sr	3.942921	11.836463	11.541344	1.828530
244	Sr	7.890260	0.000000	0.000000	1.319101
245	Sr	7.890260	3.945130	0.000000	1.319218
246	Sr	7.890260	3.945130	3.945120	1.573972
247	Sr	7.888477	3.944846	7.813344	1.577863
248	Sr	7.888587	3.940759	11.547904	1.819420
249	Sr	7.890260	7.890260	0.000000	1.319523
250	Sr	7.890260	7.890260	3.945120	1.573575
251	Sr	7.889692	7.889471	7.822400	1.576667
252	Sr	7.885163	7.893590	11.607776	1.774332
253	Sr	7.890260	0.000000	3.945120	1.573570
254	Sr	7.888792	0.000395	7.812224	1.577785
255	Sr	7.890797	-0.001452	11.547136	1.826222
256	Sr	7.890260	11.835390	0.000000	1.319384
257	Sr	7.890260	11.835390	3.945120	1.574168
258	Sr	7.890623	11.835280	7.810880	1.577698
259	Sr	7.890718	11.834017	11.536928	1.835336
260	Sr	0.000000	3.945130	0.000000	1.318923
261	Sr	0.000000	3.945130	3.945120	1.574001
262	Sr	-0.000978	3.944025	7.811648	1.578115
263	Sr	0.000047	3.945367	11.546272	1.822355
264	Sr	0.000000	7.890260	0.000000	1.319106
265	Sr	0.000000	7.890260	3.945120	1.573443
266	Sr	-0.001420	7.890355	7.812512	1.578015
267	Sr	0.000505	7.888666	11.548000	1.825280
268	Sr	0.000000	0.000000	3.945120	1.574266
269	Sr	-0.001010	-0.000158	7.809824	1.578325
270	Sr	-0.001957	0.000426	11.542720	1.824637
271	Sr	0.000000	11.835390	0.000000	1.319033
272	Sr	0.000000	11.835390	3.945120	1.574105

273	Sr	-0.002036	11.836132	7.811648	1.577873
274	Sr	-0.000631	11.835564	11.546656	1.821663
275	Sr	11.835390	0.000000	0.000000	1.318911
276	Sr	11.835390	3.945130	0.000000	1.318972
277	Sr	11.835390	3.945130	3.945120	1.574313
278	Sr	11.833654	3.945651	7.811872	1.577077
279	Sr	11.834191	3.944578	11.540320	1.830083
280	Sr	11.835390	7.890260	0.000000	1.319218
281	Sr	11.835390	7.890260	3.945120	1.574007
282	Sr	11.834664	7.890702	7.813568	1.578112
283	Sr	11.839051	7.891396	11.548736	1.818195
284	Sr	11.835390	0.000000	3.945120	1.573995
285	Sr	11.835295	-0.000300	7.811232	1.577961
286	Sr	11.833070	-0.001326	11.545344	1.823193
287	Sr	11.835390	11.835390	0.000000	1.319149
288	Sr	11.835390	11.835390	3.945120	1.574025
289	Sr	11.834112	11.834001	7.813440	1.576976
290	Sr	11.838341	11.836116	11.546048	1.824638
291	Ti	1.972565	1.972565	1.972576	2.637214
292	Ti	1.972754	1.972723	5.899520	2.621694
293	Ti	1.970577	1.972234	9.840480	2.649763
294	Ti	1.972817	5.917064	5.900288	2.621171
295	Ti	1.969993	5.917111	9.841184	2.649091
296	Ti	1.972565	5.917695	1.972576	2.637159
297	Ti	1.972344	9.862651	5.899424	2.620774
298	Ti	1.969725	9.862715	9.839872	2.649703
299	Ti	1.972565	9.862825	1.972576	2.637142
300	Ti	1.970024	13.808381	9.840000	2.649873
301	Ti	1.972565	13.807955	1.972576	2.637199
302	Ti	1.972139	13.807781	5.899392	2.621841
303	Ti	5.917695	1.972565	1.972576	2.637197
304	Ti	5.917095	1.972991	5.901152	2.621357
305	Ti	5.915849	1.972107	9.844352	2.649208
306	Ti	5.917695	5.917190	5.901312	2.619600
307	Ti	5.920614	5.919589	9.849024	2.646111
308	Ti	5.917695	5.917695	1.972576	2.637094
309	Ti	5.918074	9.861926	5.898944	2.619474
310	Ti	5.919746	9.858990	9.841216	2.647736
311	Ti	5.917695	9.862825	1.972576	2.637062
312	Ti	5.915580	13.808839	9.839744	2.649618
313	Ti	5.917695	13.807955	1.972576	2.637133
314	Ti	5.917190	13.807624	5.899456	2.620693
315	Ti	9.862415	1.973117	5.899648	2.620714
316	Ti	9.860000	1.971602	9.839232	2.650355
317	Ti	9.862178	5.917805	5.900352	2.618624
318	Ti	9.857901	5.920914	9.847200	2.647065
319	Ti	9.862825	5.917695	1.972576	2.637083
320	Ti	9.862809	9.862225	5.901184	2.619128
321	Ti	9.859101	9.858059	9.848672	2.646869
322	Ti	9.862825	9.862825	1.972576	2.637096
323	Ti	9.861484	13.808728	9.840384	2.650143
324	Ti	9.862825	13.807955	1.972576	2.637164
325	Ti	9.862762	13.807166	5.900096	2.620823
326	Ti	9.862825	1.972565	1.972576	2.637174
327	Ti	13.807560	1.972549	5.899040	2.621257
328	Ti	13.806487	1.971981	9.839872	2.649810
329	Ti	13.806992	5.917585	5.899744	2.620627
330	Ti	13.807213	5.918516	9.839872	2.649783
331	Ti	13.807955	5.917695	1.972576	2.637171
332	Ti	13.807198	9.862793	5.901120	2.621126
333	Ti	13.806519	9.862478	9.844032	2.648598
334	Ti	13.807955	9.862825	1.972576	2.637190
335	Ti	13.806282	13.807892	9.839744	2.650579
336	Ti	13.807955	13.807955	1.972576	2.637222
337	Ti	13.807434	13.807292	5.899296	2.621844
338	Ti	13.807955	1.972565	1.972576	2.637221
339	C	7.977763	7.749229	18.644576	3.975065
340	H	6.527912	9.207350	18.484256	0.873251
341	H	7.314886	7.104501	19.250304	0.954443
342	H	8.611588	8.401502	19.273120	0.953378
343	O	7.076569	8.664184	17.884992	2.580964
344	O	8.724655	7.016824	17.790368	2.540657

1.1.11 CH(OH)(OH)

```
  342
jmolscript: load "" {1 1 1} spacegroup "x,y,z" unitcell [{      15.780520
       0.000000      0.000000 }, {       0.000000     15.780520      0.000000
    }, {       0.000000      0.000000     32.000000 }]
O         1.972565      1.972565      3.945120        1.916833
O         1.974964      1.972076      7.862112        1.905540
O         1.986562      1.980660     11.797984        1.952710
O         1.972565      1.972565      0.000000        1.519970
O         1.972565      3.945130      1.972576        1.922897
O         1.978388      3.946014      5.903072        1.928330
O         1.974758      3.946456      9.804000        1.982144
O         1.972565      5.917695      3.945120        1.917141
O         1.982838      5.920599      7.863328        1.905896
O         1.969582      5.915139     11.797184        1.964393
O         1.972565      5.917695      0.000000        1.519903
O         1.972565      7.890260      1.972576        1.922769
O         1.968793      7.890008      5.900352        1.928827
O         1.977141      7.891144      9.808896        1.981205
O         1.973464      9.859764      7.860256        1.906938
O         1.971350      9.872814     11.793152        1.947586
O         1.972565      9.862825      0.000000        1.520007
O         1.972565      9.862825      3.945120        1.917595
O         1.972565      0.000000      1.972576        1.922785
O         1.969535      0.000899      5.899840        1.929082
O         1.975863      0.001862      9.808352        1.981903
O         1.972565     11.835390      1.972576        1.922850
O         1.978877     11.835832      5.903232        1.928495
O         1.973543     11.836810      9.796800        1.982154
O         1.974348     13.803600     11.792768        1.947506
O         1.972565     13.807955      0.000000        1.520000
O         1.972565     13.807955      3.945120        1.917288
O         1.973480     13.813115      7.859712        1.907558
O         3.945130      1.972565      1.972576        1.922845
O         3.946408      1.969125      5.901696        1.928539
O         3.948696      1.975974      9.805728        1.981800
O         3.946897      5.922729      5.899584        1.929072
O         3.949627      5.914997      9.820032        1.979996
O         3.945130      5.917695      1.972576        1.922708
O         3.946329      9.857901      5.901472        1.928515
O         3.946692      9.864924      9.802976        1.982105
O         3.945130      9.862825      1.972576        1.922827
O         3.947229     13.808728      9.801024        1.982387
O         3.945130     13.807955      1.972576        1.922823
O         3.946740     13.813068      5.901824        1.928677
O         5.917695      1.972565      3.945120        1.917841
O         5.918121      1.975358      7.863584        1.905432
O         5.921609      1.975958     11.799840        1.962893
O         5.917695      3.945130      1.972576        1.922837
O         5.914492      3.946124      5.902720        1.927715
O         5.919573      3.946361      9.814016        1.978106
O         5.917695      5.917695      3.945120        1.918852
O         5.912551      5.916732      7.867264        1.904465
O         5.937168      5.913387     11.809344        1.965911
O         5.917695      5.917695      0.000000        1.519686
O         5.917695      7.890260      1.972576        1.922776
O         5.923786      7.889108      5.902304        1.928539
O         5.917853      7.889723      9.811744        1.982331
O         5.918957      9.858170      7.861056        1.907778
O         5.919099      9.876365     11.793504        1.959645
O         5.917695      9.862825      0.000000        1.519870
O         5.917695      9.862825      3.945120        1.917971
O         5.923234      0.001215      5.902176        1.928855
O         5.919683      0.001184      9.808608        1.981578
O         5.917695     11.835390      1.972576        1.922790
O         5.913829     11.835627      5.904256        1.927939
O         5.920977     11.836163      9.795136        1.982341
O         5.916938     13.796230     11.793824        1.964455
O         5.917695     13.807955      0.000000        1.519827
```

O	5.917695	13.807955	3.945120	1.918417
O	5.921388	13.812531	7.861120	1.908067
O	5.917695	1.972565	0.000000	1.519768
O	5.917695	0.000000	1.972576	1.922780
O	7.890260	1.972565	1.972576	1.922774
O	7.890733	1.977331	5.901952	1.928012
O	7.891775	1.974522	9.801248	1.983102
O	7.889992	5.913261	5.905728	1.927299
O	7.891175	5.917143	9.797984	1.982257
O	7.890260	5.917695	1.972576	1.922964
O	7.890733	9.868143	5.901248	1.928523
O	7.892611	9.863283	9.802880	1.983108
O	7.890260	9.862825	1.972576	1.922815
O	7.892974	13.809565	9.806112	1.982098
O	7.890260	13.807955	1.972576	1.922809
O	7.890939	13.803805	5.900800	1.928490
O	9.862825	1.972565	3.945120	1.919081
O	9.864545	1.974175	7.858784	1.910364
O	9.860963	1.973812	11.785792	1.977530
O	9.868080	3.945635	5.900256	1.928589
O	9.865981	3.946708	9.802176	1.984430
O	9.862825	5.917695	3.945120	1.917513
O	9.867843	5.917585	7.860640	1.907140
O	9.849238	5.919273	11.795456	1.950204
O	9.862825	5.917695	0.000000	1.519828
O	9.862825	7.890260	1.972576	1.922830
O	9.858012	7.890434	5.901344	1.928505
O	9.866612	7.890844	9.800288	1.983939
O	9.864640	9.864514	7.859904	1.908077
O	9.870684	9.861010	11.791744	1.957042
O	9.862825	9.862825	0.000000	1.519944
O	9.862825	9.862825	3.945120	1.918309
O	9.866944	0.000252	9.799360	1.982959
O	9.862825	11.835390	1.972576	1.922752
O	9.868758	11.835690	5.900192	1.929027
O	9.864182	11.836163	9.803744	1.982746
O	9.871615	13.810006	11.794400	1.955141
O	9.862825	13.807955	0.000000	1.519873
O	9.862825	13.807955	3.945120	1.918603
O	9.863488	13.806645	7.860608	1.906987
O	9.862825	1.972565	0.000000	1.519793
O	9.862825	3.945130	1.972576	1.922750
O	9.862825	0.000000	1.972576	1.922824
O	9.859085	0.000252	5.901472	1.928073
O	0.000000	1.972565	1.972576	1.922814
O	0.001262	1.978009	5.901152	1.928831
O	0.003929	1.973149	9.808096	1.982412
O	0.001357	5.912977	5.906304	1.927555
O	0.002793	5.920378	9.799840	1.980746
O	0.000000	5.917695	1.972576	1.922929
O	0.000821	9.868380	5.900960	1.928960
O	0.001578	9.861720	9.802304	1.982786
O	0.000000	9.862825	1.972576	1.922770
O	0.002241	13.810496	9.802304	1.982806
O	0.000000	13.807955	1.972576	1.922775
O	0.000805	13.803615	5.900672	1.928823
O	11.835390	1.972565	1.972576	1.922781
O	11.836810	1.968494	5.901984	1.927985
O	11.838751	1.973780	9.802304	1.981534
O	11.836321	5.923029	5.899872	1.929178
O	11.836716	5.918184	9.812640	1.982157
O	11.835390	5.917695	1.972576	1.922649
O	11.836305	9.857602	5.901088	1.928532
O	11.838373	9.865602	9.801760	1.982538
O	11.835390	9.862825	1.972576	1.922803
O	11.838799	13.807308	9.801952	1.982223
O	11.835390	13.807955	1.972576	1.922814
O	11.836274	13.813510	5.901792	1.928775
O	13.812689	1.974396	7.861408	1.907043
O	13.812121	1.975800	11.795456	1.955105
O	13.804546	3.945903	5.901440	1.928411
O	13.811016	3.946613	9.803232	1.983193

139	O	13.807955	5.917695	3.945120	1.917858
140	O	13.804515	5.918484	7.862112	1.906744
141	O	13.819112	5.919510	11.795712	1.962293
142	O	13.807955	7.890260	1.972576	1.922839
143	O	13.814599	7.890386	5.902016	1.928480
144	O	13.808381	7.891302	9.803712	1.982023
145	O	13.809801	9.864892	7.861120	1.906508
146	O	13.810575	9.861279	11.796032	1.953527
147	O	13.807955	9.862825	0.000000	1.519945
148	O	13.807955	9.862825	3.945120	1.917511
149	O	13.810006	0.001799	9.802400	1.982808
150	O	13.807955	11.835390	1.972576	1.922778
151	O	13.803757	11.835895	5.901024	1.928776
152	O	13.812121	11.836652	9.807360	1.981143
153	O	13.812894	13.815829	11.795264	1.955398
154	O	13.807955	13.807955	0.000000	1.519916
155	O	13.807955	13.807955	3.945120	1.917748
156	O	13.810117	13.807624	7.860960	1.907151
157	O	13.807955	1.972565	3.945120	1.918079
158	O	13.807955	1.972565	0.000000	1.519825
159	O	13.807955	3.945130	1.972576	1.922784
160	O	13.807955	5.917695	0.000000	1.519812
161	O	13.807955	0.000000	1.972576	1.922866
162	O	13.813857	0.000489	5.902144	1.928680
163	Pd	2.023189	1.998319	13.972448	3.059982
164	Pd	2.050789	3.917198	16.087168	2.993609
165	Pd	2.034093	5.900699	13.959296	3.067985
166	Pd	1.964044	7.975854	16.190816	2.947409
167	Pd	1.927433	9.913465	13.972800	3.124577
168	Pd	1.984448	0.036248	16.180800	2.932047
169	Pd	1.950314	11.810204	16.140320	2.966111
170	Pd	1.968446	13.792711	13.972704	3.100549
171	Pd	4.000851	1.938211	16.143584	2.974190
172	Pd	4.149803	3.948744	14.454560	3.483010
173	Pd	3.884454	5.995414	16.109408	3.029021
174	Pd	3.878157	7.991066	14.386848	3.508955
175	Pd	3.988432	9.895522	16.192992	2.932749
176	Pd	3.923921	15.755429	14.393504	3.514712
177	Pd	3.944341	11.815080	14.400832	3.492811
178	Pd	3.912354	13.792269	16.135808	2.976079
179	Pd	5.926627	1.878734	13.956096	3.128745
180	Pd	5.864720	4.051412	16.368288	2.880554
181	Pd	5.864388	6.058363	13.949248	3.153546
182	Pd	5.918626	7.895389	16.087584	3.013742
183	Pd	5.924149	9.884744	13.958176	3.091340
184	Pd	5.930509	15.712837	16.120832	2.968393
185	Pd	5.925254	11.881516	16.152384	2.954199
186	Pd	5.898885	13.797997	13.953952	3.124963
187	Pd	7.862123	1.965953	16.036576	3.081705
188	Pd	7.723428	4.022896	14.410848	3.328374
189	Pd	7.936639	5.997150	16.241248	3.429564
190	Pd	7.922515	7.898482	14.362464	3.444698
191	Pd	7.882685	9.867449	16.103488	2.981007
192	Pd	7.902474	15.686515	14.372416	3.487038
193	Pd	7.894205	11.869744	14.400320	3.480663
194	Pd	7.962361	13.799639	16.181344	2.923098
195	Pd	9.851621	1.979335	13.940928	3.051689
196	Pd	9.818671	3.901402	16.024608	3.041776
197	Pd	9.881288	5.923644	13.968128	3.047188
198	Pd	9.880199	7.924614	16.135872	3.028923
199	Pd	9.896895	9.894828	13.961504	3.077249
200	Pd	9.858233	0.072054	16.137120	2.960563
201	Pd	9.894418	11.797217	16.122784	2.981442
202	Pd	9.910451	13.793074	13.963712	3.106718
203	Pd	0.001925	1.994216	16.132576	2.945765
204	Pd	0.025138	3.960800	14.394464	3.485303
205	Pd	0.088544	5.907737	16.159200	2.926282
206	Pd	0.039262	7.867552	14.400800	3.471079
207	Pd	15.729438	9.870936	16.124256	2.994019
208	Pd	0.043097	0.019331	14.395520	3.476541
209	Pd	15.751452	11.866541	14.398176	3.510085
210	Pd	0.038536	13.834435	16.149632	2.964784

211	Pd	11.863842	2.004836	16.155552	2.914940
212	Pd	11.881406	3.936482	14.377824	3.475183
213	Pd	11.794298	5.905386	16.133824	2.982718
214	Pd	11.861096	7.896714	14.397728	3.474151
215	Pd	11.896382	9.861563	16.158656	2.924933
216	Pd	11.831035	0.045984	14.397568	3.493908
217	Pd	11.883284	11.827626	14.402912	3.509398
218	Pd	11.819215	13.830537	16.117760	2.957229
219	Pd	13.840242	1.977473	13.965184	3.104141
220	Pd	13.830616	4.000062	16.141024	2.943837
221	Pd	13.825850	5.919636	13.959168	3.103436
222	Pd	13.830789	7.826870	16.124512	2.955971
223	Pd	13.832099	9.856797	13.967232	3.099775
224	Pd	13.823657	0.000347	16.115744	2.963003
225	Pd	13.818417	11.896855	16.177088	2.935530
226	Pd	13.805888	13.843903	13.965056	3.090926
227	Sr	0.000000	0.000000	0.000000	1.317089
228	Sr	3.945130	0.000000	0.000000	1.317070
229	Sr	3.945130	3.945130	0.000000	1.317029
230	Sr	3.945130	3.945130	3.945120	1.574486
231	Sr	3.944641	3.945020	7.815968	1.577791
232	Sr	3.940617	3.940412	11.568608	1.793930
233	Sr	3.945130	7.890260	0.000000	1.317102
234	Sr	3.945130	7.890260	3.945120	1.574405
235	Sr	3.945556	7.890812	7.812416	1.577045
236	Sr	3.947118	7.894032	11.542912	1.828631
237	Sr	3.945130	0.000000	3.945120	1.574346
238	Sr	3.945382	0.001404	7.812192	1.577495
239	Sr	3.946250	-0.000931	11.545280	1.826355
240	Sr	3.945130	11.835390	0.000000	1.317133
241	Sr	3.945130	11.835390	3.945120	1.574065
242	Sr	3.944783	11.835848	7.811776	1.578072
243	Sr	3.946093	11.837536	11.546976	1.823228
244	Sr	7.890260	0.000000	0.000000	1.317023
245	Sr	7.890260	3.945130	0.000000	1.316962
246	Sr	7.890260	3.945130	3.945120	1.574338
247	Sr	7.891980	3.945856	7.814368	1.578063
248	Sr	7.904573	3.943599	11.565856	1.809194
249	Sr	7.890260	7.890260	0.000000	1.317060
250	Sr	7.890260	7.890260	3.945120	1.574104
251	Sr	7.891538	7.890733	7.810240	1.577833
252	Sr	7.895152	7.891980	11.533760	1.841078
253	Sr	7.890260	0.000000	3.945120	1.574489
254	Sr	7.891112	0.000347	7.809888	1.577970
255	Sr	7.897046	0.003614	11.536832	1.835612
256	Sr	7.890260	11.835390	0.000000	1.317100
257	Sr	7.890260	11.835390	3.945120	1.574202
258	Sr	7.891854	11.835879	7.811520	1.578087
259	Sr	7.890497	11.835201	11.548320	1.819395
260	Sr	0.000000	3.945130	0.000000	1.317052
261	Sr	0.000000	3.945130	3.945120	1.573891
262	Sr	0.001689	3.945146	7.813024	1.577262
263	Sr	-0.001673	3.946566	11.547456	1.825938
264	Sr	0.000000	7.890260	0.000000	1.317099
265	Sr	0.000000	7.890260	3.945120	1.574028
266	Sr	0.002193	7.891049	7.813248	1.577354
267	Sr	0.000032	7.891538	11.550496	1.820497
268	Sr	0.000000	0.000000	3.945120	1.574209
269	Sr	0.002336	0.000789	7.812576	1.577457
270	Sr	-0.000047	-0.000899	11.549152	1.821863
271	Sr	0.000000	11.835390	0.000000	1.317125
272	Sr	0.000000	11.835390	3.945120	1.574028
273	Sr	0.001452	11.835800	7.811840	1.578227
274	Sr	0.004277	11.835942	11.546752	1.824342
275	Sr	11.835390	0.000000	0.000000	1.317027
276	Sr	11.835390	3.945130	0.000000	1.316965
277	Sr	11.835390	3.945130	3.945120	1.574406
278	Sr	11.837852	3.946140	7.810720	1.577951
279	Sr	11.837946	3.943094	11.541312	1.830601
280	Sr	11.835390	7.890260	0.000000	1.317049
281	Sr	11.835390	7.890260	3.945120	1.574094
282	Sr	11.836274	7.890939	7.812320	1.577754

```
Sr      11.834506       7.893116      11.548928        1.821917
Sr      11.835390       0.000000       3.945120        1.574296
Sr      11.837410       0.000095       7.811008        1.577650
Sr      11.837647      -0.001404      11.546080        1.821827
Sr      11.835390      11.835390       0.000000        1.317094
Sr      11.835390      11.835390       3.945120        1.574300
Sr      11.837599      11.835863       7.811360        1.577828
Sr      11.836637      11.836589      11.548384        1.821570
Ti       1.972565       1.972565       1.972576        2.637403
Ti       1.973259       1.972770       5.900064        2.620388
Ti       1.975484       1.974190       9.844704        2.649100
Ti       1.973827       5.917884       5.899776        2.620472
Ti       1.975074       5.917190       9.844128        2.647572
Ti       1.972565       5.917695       1.972576        2.637370
Ti       1.972944       9.862746       5.899328        2.620724
Ti       1.973117       9.864151       9.841568        2.648889
Ti       1.972565       9.862825       1.972576        2.637399
Ti       1.973859      13.808649       9.841408        2.648336
Ti       1.972565      13.807955       1.972576        2.637403
Ti       1.972991      13.808634       5.899584        2.621259
Ti       5.917695       1.972565       1.972576        2.637351
Ti       5.917853       1.973275       5.899104        2.619781
Ti       5.919683       1.975926       9.842080        2.647328
Ti       5.917490       5.917632       5.898144        2.619211
Ti       5.920094       5.914334       9.841728        2.646388
Ti       5.917695       5.917695       1.972576        2.637333
Ti       5.918184       9.863077       5.898944        2.620372
Ti       5.918752       9.864845       9.839200        2.649555
Ti       5.917695       9.862825       1.972576        2.637367
Ti       5.919115      13.808397       9.838432        2.649013
Ti       5.917695      13.807955       1.972576        2.637368
Ti       5.918026      13.808634       5.898560        2.620569
Ti       9.863298       1.973117       5.897824        2.620926
Ti       9.864719       1.974064       9.835488        2.652316
Ti       9.863882       5.917900       5.899360        2.620658
Ti       9.863567       5.917679       9.840896        2.648811
Ti       9.862825       5.917695       1.972576        2.637393
Ti       9.863788       9.863298       5.898656        2.620762
Ti       9.865792       9.864529       9.839008        2.650113
Ti       9.862825       9.862825       1.972576        2.637417
Ti       9.865571      13.807923       9.839424        2.648924
Ti       9.862825      13.807955       1.972576        2.637400
Ti       9.863646      13.808097       5.898432        2.620741
Ti       9.862825       1.972565       1.972576        2.637400
Ti      13.809154       1.972770       5.898816        2.620541
Ti      13.810653       1.972707       9.840320        2.648505
Ti      13.808602       5.918105       5.899072        2.620117
Ti      13.810022       5.919336       9.840704        2.648739
Ti      13.807955       5.917695       1.972576        2.637375
Ti      13.808902       9.863030       5.899360        2.620405
Ti      13.809691       9.863377       9.841600        2.647426
Ti      13.807955       9.862825       1.972576        2.637395
Ti      13.810417      13.808618       9.840576        2.648736
Ti      13.807955      13.807955       1.972576        2.637395
Ti      13.809012      13.808050       5.899104        2.620706
Ti      13.807955       1.972565       1.972576        2.637397
C        7.865753       5.416790      18.170176        3.987790
H        7.286766       6.230954      18.640864        0.956383
H        7.715917       3.479652      18.077408        0.943883
H        9.780246       5.730159      18.153664        1.003705
O        7.113748       4.233914      18.327072        2.382522
O        9.133812       5.226492      18.707392        2.285986
```

1.1.12 COH

```
       339
jmolscript: load "" {1 1 1} spacegroup "x,y,z" unitcell [{      15.780520
```

```
    0.000000     0.000000 }, {     0.000000    15.780520     0.000000
  }, {     0.000000     0.000000    32.000000 }]
O        1.972565      1.972565      3.945120       1.917566
O        1.967989      1.972944      7.860128       1.908084
O        1.961408      1.973528     11.793536       1.948743
O        1.972565      1.972565      0.000000       1.520329
O        1.972565      3.945130      1.972576       1.922745
O        1.970245      3.945430      5.901408       1.928573
O        1.967421      3.945525      9.802208       1.982428
O        1.972565      5.917695      3.945120       1.917324
O        1.967926      5.919036      7.860800       1.906987
O        1.961787      5.916906     11.795328       1.950819
O        1.972565      5.917695      0.000000       1.520285
O        1.972565      7.890260      1.972576       1.922808
O        1.970214      7.890670      5.901216       1.928501
O        1.967389      7.890812      9.805504       1.981521
O        1.967657      9.863141      7.862432       1.905199
O        1.963254      9.863693     11.796704       1.954953
O        1.972565      9.862825      0.000000       1.520388
O        1.972565      9.862825      3.945120       1.916811
O        1.972565      0.000000      1.972576       1.922799
O        1.970214     -0.000016      5.901408       1.928559
O        1.967421      0.000395      9.802272       1.982104
O        1.972565     11.835390      1.972576       1.922815
O        1.970245     11.835295      5.901216       1.928418
O        1.967421     11.835674      9.805536       1.981308
O        1.961882     13.810148     11.795136       1.951123
O        1.972565     13.807955      0.000000       1.520392
O        1.972565     13.807955      3.945120       1.917181
O        1.968099     13.807308      7.860736       1.906981
O        3.945130      1.972565      1.972576       1.922762
O        3.943063      1.972644      5.901408       1.928635
O        3.939323      1.972991      9.802336       1.982207
O        3.943142      5.918042      5.901376       1.928378
O        3.939054      5.917963      9.802624       1.982762
O        3.945130      5.917695      1.972576       1.922755
O        3.942826      9.862920      5.901568       1.928889
O        3.939986      9.863156      9.806336       1.982049
O        3.945130      9.862825      1.972576       1.922760
O        3.938960     13.808207      9.802528       1.982744
O        3.945130     13.807955      1.972576       1.922743
O        3.943110     13.808018      5.901184       1.928504
O        5.917695      1.972565      3.945120       1.917424
O        5.914113      1.973449      7.860672       1.907086
O        5.905970      1.973086     11.795296       1.950271
O        5.917695      3.945130      1.972576       1.922714
O        5.915502      3.945461      5.901024       1.928649
O        5.912472      3.945067      9.802688       1.982804
O        5.917695      5.917695      3.945120       1.918331
O        5.913718      5.917869      7.861280       1.907399
O        5.904061      5.914586     11.794560       1.957603
O        5.917695      5.917695      0.000000       1.520276
O        5.917695      7.890260      1.972576       1.922797
O        5.915628      7.890402      5.901600       1.928433
O        5.911951      7.890165      9.803840       1.982816
O        5.912393      9.863519      7.861024       1.905662
O        5.911951      9.862525     11.796064       1.933850
O        5.917695      9.862825      0.000000       1.520353
O        5.917695      9.862825      3.945120       1.916755
O        5.915612     -0.000047      5.901440       1.928426
O        5.912235      0.001010      9.801600       1.982626
O        5.917695     11.835390      1.972576       1.922778
O        5.915628     11.835548      5.901088       1.928626
O        5.912124     11.836163      9.804928       1.982602
O        5.903855     13.814204     11.794240       1.957342
O        5.917695     13.807955      0.000000       1.520401
O        5.917695     13.807955      3.945120       1.918225
O        5.913387     13.807576      7.861152       1.907623
O        5.917695      1.972565      0.000000       1.520345
O        5.917695      0.000000      1.972576       1.922797
O        7.890260      1.972565      1.972576       1.922788
O        7.888398      1.972754      5.901344       1.928286
```

O	7.884753	1.972849	9.805248	1.981263
O	7.888130	5.918105	5.901600	1.928264
O	7.884453	5.917269	9.804672	1.982841
O	7.890260	5.917695	1.972576	1.922798
O	7.888903	9.863014	5.901824	1.927688
O	7.885021	9.862746	9.800288	1.984271
O	7.890260	9.862825	1.972576	1.922714
O	7.884216	13.809549	9.803616	1.983408
O	7.890260	13.807955	1.972576	1.922783
O	7.888145	13.807766	5.901440	1.928358
O	9.862825	1.972565	3.945120	1.916962
O	9.858659	1.972407	7.862688	1.904791
O	9.852662	1.973622	11.796480	1.956621
O	9.860458	3.945130	5.901792	1.928841
O	9.857838	3.946345	9.807840	1.982329
O	9.862825	5.917695	3.945120	1.916631
O	9.858738	5.917443	7.861408	1.905119
O	9.853041	5.926185	11.797280	1.932896
O	9.862825	5.917695	0.000000	1.520286
O	9.862825	7.890260	1.972576	1.922701
O	9.860553	7.891112	5.901568	1.927810
O	9.857696	7.891428	9.801120	1.983927
O	9.858012	9.861799	7.861760	1.913748
O	9.852078	9.866139	11.793792	1.996448
O	9.862825	9.862825	0.000000	1.520057
O	9.862825	9.862825	3.945120	1.922881
O	9.857759	-0.000205	9.808512	1.982386
O	9.862825	11.835390	1.972576	1.922735
O	9.860474	11.834806	5.902656	1.927183
O	9.857665	11.834995	9.797440	1.985144
O	9.852978	13.799055	11.795296	1.939165
O	9.862825	13.807955	0.000000	1.520393
O	9.862825	13.807955	3.945120	1.917444
O	9.858548	13.810622	7.860832	1.906573
O	9.862825	1.972565	0.000000	1.520394
O	9.862825	3.945130	1.972576	1.922746
O	9.862825	0.000000	1.972576	1.922760
O	9.860505	0.000284	5.900992	1.928863
O	0.000000	1.972565	1.972576	1.922800
O	-0.002383	1.972660	5.901280	1.928643
O	-0.005870	1.972975	9.802336	1.982435
O	-0.002430	5.917995	5.901216	1.928440
O	-0.005523	5.917853	9.802976	1.982514
O	0.000000	5.917695	1.972576	1.922767
O	-0.002115	9.862920	5.901056	1.929028
O	-0.006233	9.863172	9.808064	1.982526
O	0.000000	9.862825	1.972576	1.922736
O	-0.005318	13.808302	9.802592	1.982427
O	0.000000	13.807955	1.972576	1.922747
O	-0.002336	13.808018	5.901056	1.928515
O	11.835390	1.972565	1.972576	1.922813
O	11.832912	1.972786	5.901216	1.928585
O	11.829551	1.972833	9.805536	1.981353
O	11.833181	5.917963	5.901408	1.928500
O	11.829930	5.917411	9.804832	1.982950
O	11.835390	5.917695	1.972576	1.922807
O	11.832329	9.863093	5.902240	1.927448
O	11.828810	9.862730	9.799840	1.984039
O	11.835390	9.862825	1.972576	1.922734
O	11.830119	13.809470	9.803552	1.983383
O	11.835390	13.807955	1.972576	1.922784
O	11.833133	13.807781	5.901344	1.928479
O	13.802700	1.973496	7.860928	1.906922
O	13.798929	1.972754	11.795680	1.949753
O	13.805509	3.945446	5.901152	1.928645
O	13.802858	3.945177	9.803936	1.982576
O	13.807955	5.917695	3.945120	1.918140
O	13.803237	5.917742	7.861504	1.907239
O	13.800570	5.916496	11.795296	1.956827
O	13.807955	7.890260	1.972576	1.922802
O	13.805162	7.890323	5.901696	1.928484
O	13.803694	7.890323	9.804032	1.982893

145	O	13.805115	9.863251	7.861088	1.905651
146	O	13.790723	9.862872	11.796192	1.933661
147	O	13.807955	9.862825	0.000000	1.520356
148	O	13.807955	9.862825	3.945120	1.916502
149	O	13.803110	0.000899	9.802496	1.982523
150	O	13.807955	11.835390	1.972576	1.922779
151	O	13.805320	11.835595	5.901344	1.928690
152	O	13.803505	11.836053	9.804416	1.982709
153	O	13.801059	13.812389	11.794432	1.955952
154	O	13.807955	13.807955	0.000000	1.520405
155	O	13.807955	13.807955	3.945120	1.918071
156	O	13.803615	13.807955	7.861120	1.907683
157	O	13.807955	1.972565	3.945120	1.917170
158	O	13.807955	1.972565	0.000000	1.520351
159	O	13.807955	3.945130	1.972576	1.922728
160	O	13.807955	5.917695	0.000000	1.520284
161	O	13.807955	0.000000	1.972576	1.922794
162	O	13.805335	0.000047	5.901440	1.928462
163	Pd	1.931646	1.980108	13.970752	3.096166
164	Pd	1.928774	3.933878	16.152544	2.957629
165	Pd	1.932703	5.911241	13.969952	3.097507
166	Pd	1.932104	7.898513	16.146624	2.959120
167	Pd	1.936301	9.868885	13.967840	3.097791
168	Pd	1.928885	0.019757	16.157856	2.956085
169	Pd	1.932088	11.832739	16.138240	2.964487
170	Pd	1.933682	13.816697	13.969504	3.094599
171	Pd	3.911186	1.977552	16.150912	2.958133
172	Pd	3.896873	3.946740	14.395392	3.498762
173	Pd	3.894159	5.911762	16.145024	2.956065
174	Pd	3.890324	7.890086	14.400736	3.487679
175	Pd	3.915510	9.865097	16.148352	2.955205
176	Pd	3.890009	0.009626	14.395328	3.499344
177	Pd	3.900234	11.842349	14.402496	3.487714
178	Pd	3.892186	13.823341	16.144608	2.959836
179	Pd	5.868176	1.974159	13.970336	3.094904
180	Pd	5.866976	3.957486	16.141408	2.953147
181	Pd	5.865035	5.904818	13.961024	3.101172
182	Pd	5.881037	7.877273	16.126496	2.956616
183	Pd	5.856956	9.861326	13.981696	3.082113
184	Pd	5.867087	-0.006312	16.136608	2.955320
185	Pd	5.878054	11.857483	16.140416	2.962270
186	Pd	5.858550	13.835366	13.960928	3.105743
187	Pd	7.834855	1.975974	16.147360	2.961115
188	Pd	7.844496	3.937618	14.400096	3.487083
189	Pd	7.873880	5.928473	16.126400	2.949454
190	Pd	7.820194	7.867820	14.359168	3.419693
191	Pd	7.875237	9.871552	16.164864	3.212303
192	Pd	7.847684	0.015591	14.399424	3.485752
193	Pd	7.793447	11.864268	14.349120	3.412471
194	Pd	7.878567	13.801659	16.123168	2.988530
195	Pd	9.826609	1.976794	13.966080	3.101103
196	Pd	9.821496	3.921680	16.145152	2.960801
197	Pd	9.822679	5.899863	13.983744	3.074663
198	Pd	9.816446	7.962251	16.181600	3.196994
199	Pd	9.823027	9.862825	13.914752	3.007888
200	Pd	9.821212	0.027016	16.149664	2.964626
201	Pd	9.816178	11.785413	16.131584	3.352539
202	Pd	9.821322	13.828186	13.975456	3.067756
203	Pd	15.733620	1.977047	16.152128	2.955986
204	Pd	15.738765	3.947071	14.396320	3.498191
205	Pd	15.753125	5.915075	16.143168	2.955643
206	Pd	15.750395	7.895404	14.402016	3.488597
207	Pd	15.739207	9.865460	16.153920	2.952444
208	Pd	15.746103	0.008553	14.396160	3.499085
209	Pd	15.742615	11.837489	14.402784	3.486901
210	Pd	15.755287	13.819538	16.143136	2.958389
211	Pd	11.816737	1.975090	16.144320	2.960921
212	Pd	11.797375	3.941406	14.401120	3.486195
213	Pd	11.774840	5.931519	16.127936	2.946291
214	Pd	11.813865	7.871749	14.361568	3.419095
215	Pd	11.757450	9.872735	16.172352	3.203746
216	Pd	11.792956	0.010620	14.399936	3.484250

Pd	11.839051	11.862769	14.350592	3.411923
Pd	11.769001	13.798439	16.126240	2.980914
Pd	13.778729	1.974427	13.970880	3.094137
Pd	13.773475	3.961447	16.145312	2.949788
Pd	13.783101	5.910105	13.962304	3.099452
Pd	13.758246	7.885841	16.124672	2.954202
Pd	13.789539	9.862809	13.983520	3.081246
Pd	13.773822	-0.011835	16.139872	2.952716
Pd	13.761055	11.849514	16.136512	2.959083
Pd	13.788861	13.830537	13.962208	3.103293
Sr	0.000000	0.000000	0.000000	1.317608
Sr	3.945130	0.000000	0.000000	1.317592
Sr	3.945130	3.945130	0.000000	1.317499
Sr	3.945130	3.945130	3.945120	1.574492
Sr	3.942384	3.944720	7.811712	1.577868
Sr	3.942447	3.944120	11.546304	1.824918
Sr	3.945130	7.890260	0.000000	1.317547
Sr	3.945130	7.890260	3.945120	1.574314
Sr	3.942132	7.890449	7.812704	1.577566
Sr	3.944420	7.888698	11.549888	1.820631
Sr	3.945130	0.000000	3.945120	1.574454
Sr	3.942353	0.000679	7.811648	1.577800
Sr	3.942510	0.001247	11.546176	1.825157
Sr	3.945130	11.835390	0.000000	1.317638
Sr	3.945130	11.835390	3.945120	1.574320
Sr	3.942179	11.835374	7.812832	1.577593
Sr	3.944641	11.836826	11.550784	1.819467
Sr	7.890260	0.000000	0.000000	1.317673
Sr	7.890260	3.945130	0.000000	1.317537
Sr	7.890260	3.945130	3.945120	1.574307
Sr	7.888130	3.944530	7.812736	1.577312
Sr	7.886331	3.946156	11.550144	1.820886
Sr	7.890260	7.890260	0.000000	1.317557
Sr	7.890260	7.890260	3.945120	1.574604
Sr	7.885968	7.887956	7.809536	1.578036
Sr	7.888540	7.890576	11.538208	1.836887
Sr	7.890260	0.000000	3.945120	1.574325
Sr	7.887830	0.001152	7.812416	1.577573
Sr	7.885857	-0.000978	11.549696	1.821119
Sr	7.890260	11.835390	0.000000	1.317685
Sr	7.890260	11.835390	3.945120	1.574665
Sr	7.885952	11.837520	7.808640	1.578073
Sr	7.891933	11.835185	11.534432	1.841734
Sr	0.000000	3.945130	0.000000	1.317517
Sr	0.000000	3.945130	3.945120	1.574506
Sr	-0.001594	3.944751	7.811936	1.577946
Sr	-0.000615	3.944199	11.547072	1.824552
Sr	0.000000	7.890260	0.000000	1.317550
Sr	0.000000	7.890260	3.945120	1.574253
Sr	-0.001247	7.890386	7.813056	1.577638
Sr	-0.002430	7.888209	11.550720	1.819993
Sr	0.000000	0.000000	3.945120	1.574440
Sr	-0.001546	0.000726	7.811872	1.577994
Sr	-0.000852	0.001120	11.546912	1.824845
Sr	0.000000	11.835390	0.000000	1.317633
Sr	0.000000	11.835390	3.945120	1.574305
Sr	-0.001341	11.835327	7.813056	1.577659
Sr	-0.002904	11.837489	11.551296	1.819145
Sr	11.835390	0.000000	0.000000	1.317671
Sr	11.835390	3.945130	0.000000	1.317536
Sr	11.835390	3.945130	3.945120	1.574221
Sr	11.833528	3.944515	7.812928	1.577523
Sr	11.835343	3.946172	11.550912	1.819983
Sr	11.835390	7.890260	0.000000	1.317549
Sr	11.835390	7.890260	3.945120	1.574535
Sr	11.835990	7.888019	7.809760	1.577990
Sr	11.834301	7.890765	11.539488	1.835905
Sr	11.835390	0.000000	3.945120	1.574317
Sr	11.833733	0.001168	7.812512	1.577612
Sr	11.835832	-0.001026	11.550464	1.820678
Sr	11.835390	11.835390	0.000000	1.317682
Sr	11.835390	11.835390	3.945120	1.574535

```
Sr     11.835990    11.837505     7.808800      1.578081
Sr     11.831350    11.835043    11.534976      1.842011
Ti      1.972565     1.972565     1.972576      2.637337
Ti      1.971555     1.972628     5.899200      2.621387
Ti      1.969204     1.972833     9.840704      2.649373
Ti      1.971618     5.917790     5.899520      2.620965
Ti      1.969362     5.917916     9.841888      2.648349
Ti      1.972565     5.917695     1.972576      2.637326
Ti      1.971618     9.862809     5.899936      2.620354
Ti      1.969519     9.863046     9.844512      2.647773
Ti      1.972565     9.862825     1.972576      2.637336
Ti      1.969362    13.807971     9.841984      2.648665
Ti      1.972565    13.807955     1.972576      2.637317
Ti      1.971587    13.807892     5.899616      2.621007
Ti      5.917695     1.972565     1.972576      2.637325
Ti      5.916653     1.972691     5.899424      2.621101
Ti      5.914539     1.972802     9.841728      2.648492
Ti      5.916306     5.917237     5.898496      2.620881
Ti      5.913324     5.916653     9.839936      2.649723
Ti      5.917695     5.917695     1.972576      2.637338
Ti      5.915817     9.862778     5.900064      2.620922
Ti      5.913229     9.862920     9.843232      2.647316
Ti      5.917695     9.862825     1.972576      2.637301
Ti      5.913229    13.809344     9.839840      2.649974
Ti      5.917695    13.807955     1.972576      2.637322
Ti      5.916164    13.808350     5.898560      2.620982
Ti      9.861910     1.972644     5.899808      2.620373
Ti      9.859858     1.972849     9.844448      2.647807
Ti      9.862036     5.916827     5.900288      2.620761
Ti      9.860000     5.916527     9.844032      2.647977
Ti      9.862825     5.917695     1.972576      2.637318
Ti      9.862068     9.862541     5.894048      2.620642
Ti      9.860032     9.862336     9.824608      2.649918
Ti      9.862825     9.862825     1.972576      2.637235
Ti      9.859953    13.810085     9.840896      2.648655
Ti      9.862825    13.807955     1.972576      2.637288
Ti      9.861973    13.809060     5.899424      2.620967
Ti      9.862825     1.972565     1.972576      2.637331
Ti     13.807040     1.972707     5.899616      2.620807
Ti     13.804815     1.972770     9.842176      2.647877
Ti     13.807560     5.917253     5.898688      2.620808
Ti     13.806109     5.916796     9.840416      2.648804
Ti     13.807955     5.917695     1.972576      2.637347
Ti     13.808239     9.862746     5.900416      2.620856
Ti     13.806661     9.862857     9.843616      2.648014
Ti     13.807955     9.862825     1.972576      2.637295
Ti     13.806251    13.809312     9.840000      2.648917
Ti     13.807955    13.807955     1.972576      2.637323
Ti     13.807671    13.808350     5.898752      2.620857
Ti     13.807955     1.972565     1.972576      2.637321
C       9.814521     9.876791    16.973888      3.973427
H       9.773381    10.808220    18.650688      0.868742
O       9.810229     9.874929    18.343808      2.372468
```

1.1.13 CHOH

```
      340
jmolscript: load "" {1 1 1} spacegroup "x,y,z" unitcell [{     15.780520
      0.000000     0.000000 }, {      0.000000    15.780520     0.000000
    }, {      0.000000     0.000000    32.000000 }]
O       1.972565     1.972565     3.945120      1.918251
O       1.967042     1.971871     7.860448      1.908164
O       1.964754     1.975469    11.793984      1.954480
O       1.972565     1.972565     0.000000      1.519838
O       1.972565     3.945130     1.972576      1.922798
O       1.970955     3.945461     5.901728      1.928513
O       1.967215     3.945509     9.800736      1.982547
```

O	1.972565	5.917695	3.945120	1.917755
O	1.966284	5.919178	7.860896	1.907855
O	1.963901	5.914602	11.794272	1.952960
O	1.972565	5.917695	0.000000	1.519807
O	1.972565	7.890260	1.972576	1.922811
O	1.970450	7.890702	5.901056	1.928630
O	1.969141	7.890670	9.805408	1.981865
O	1.975263	9.862352	7.862176	1.906415
O	1.951703	9.864750	11.795424	1.958720
O	1.972565	9.862825	0.000000	1.519794
O	1.972565	9.862825	3.945120	1.917716
O	1.972565	0.000000	1.972576	1.922802
O	1.970592	0.000268	5.900704	1.928898
O	1.967405	0.000742	9.804064	1.982375
O	1.972565	11.835390	1.972576	1.922838
O	1.970671	11.835327	5.901888	1.928277
O	1.969740	11.836005	9.803328	1.981977
O	1.963744	13.809123	11.794272	1.951833
O	1.972565	13.807955	0.000000	1.519878
O	1.972565	13.807955	3.945120	1.917972
O	1.969551	13.809107	7.860608	1.907241
O	3.945130	1.972565	1.972576	1.922818
O	3.943205	1.972865	5.901888	1.928322
O	3.939449	1.972502	9.800768	1.982333
O	3.943221	5.918437	5.902176	1.927782
O	3.939386	5.918200	9.800480	1.982353
O	3.945130	5.917695	1.972576	1.922812
O	3.943946	9.862951	5.900256	1.929060
O	3.941895	9.863298	9.814368	1.981715
O	3.945130	9.862825	1.972576	1.922736
O	3.940017	13.809312	9.802976	1.982069
O	3.945130	13.807955	1.972576	1.922791
O	3.943363	13.807829	5.900704	1.928678
O	5.917695	1.972565	3.945120	1.917869
O	5.914665	1.971019	7.860928	1.907469
O	5.904061	1.974711	11.794560	1.952570
O	5.917695	3.945130	1.972576	1.922787
O	5.915486	3.945493	5.902080	1.927893
O	5.912409	3.945414	9.800448	1.982608
O	5.917695	5.917695	3.945120	1.919124
O	5.914760	5.919352	7.860096	1.910074
O	5.907280	5.917474	11.788800	1.974048
O	5.917695	5.917695	0.000000	1.519691
O	5.917695	7.890260	1.972576	1.922793
O	5.916133	7.890686	5.901312	1.928371
O	5.913908	7.891412	9.803328	1.983498
O	5.912077	9.863156	7.863104	1.905617
O	5.923155	9.860805	11.800512	1.946524
O	5.917695	9.862825	0.000000	1.519741
O	5.917695	9.862825	3.945120	1.917880
O	5.915801	0.000110	5.900864	1.928881
O	5.913939	0.000395	9.805376	1.982147
O	5.917695	11.835390	1.972576	1.922842
O	5.916369	11.835280	5.901728	1.928615
O	5.913403	11.835232	9.804896	1.982662
O	5.905718	13.804183	11.795040	1.948089
O	5.917695	13.807955	0.000000	1.519866
O	5.917695	13.807955	3.945120	1.917493
O	5.913040	13.807797	7.860992	1.906491
O	5.917695	1.972565	0.000000	1.519807
O	5.917695	0.000000	1.972576	1.922802
O	7.890260	1.972565	1.972576	1.922787
O	7.888414	1.973354	5.900960	1.928557
O	7.884626	1.974853	9.805760	1.981788
O	7.888493	5.917869	5.901600	1.928057
O	7.885636	5.918926	9.803616	1.983772
O	7.890260	5.917695	1.972576	1.922810
O	7.888603	9.863235	5.904000	1.928127
O	7.887104	9.864277	9.803200	1.981910
O	7.890260	9.862825	1.972576	1.922931
O	7.884579	13.809296	9.803008	1.982708
O	7.890260	13.807955	1.972576	1.922820

O	7.888666	13.808397	5.901600	1.928201
O	9.862825	1.972565	3.945120	1.917636
O	9.857270	1.979950	7.862336	1.906663
O	9.854098	1.962907	11.796192	1.957243
O	9.861215	3.946187	5.900352	1.929053
O	9.858201	3.947828	9.814720	1.981849
O	9.862825	5.917695	3.945120	1.917550
O	9.858911	5.916796	7.863488	1.905116
O	9.850753	5.932844	11.801408	1.946622
O	9.862825	5.917695	0.000000	1.519748
O	9.862825	7.890260	1.972576	1.922913
O	9.860221	7.890733	5.903744	1.928294
O	9.858833	7.892958	9.804448	1.982333
O	9.862257	9.865823	7.862976	1.905408
O	9.854824	9.867165	11.801088	1.946368
O	9.862825	9.862825	0.000000	1.519777
O	9.862825	9.862825	3.945120	1.917260
O	9.857791	0.001988	9.797600	1.982065
O	9.862825	11.835390	1.972576	1.922730
O	9.861058	11.835642	5.900736	1.928881
O	9.858533	11.837299	9.807328	1.983588
O	9.850879	13.818386	11.793504	1.962694
O	9.862825	13.807955	0.000000	1.519822
O	9.862825	13.807955	3.945120	1.918292
O	9.859401	13.805982	7.861120	1.908038
O	9.862825	1.972565	0.000000	1.519798
O	9.862825	3.945130	1.972576	1.922733
O	9.862825	0.000000	1.972576	1.922899
O	9.860411	0.000978	5.904512	1.927916
O	0.000000	1.972565	1.972576	1.922801
O	-0.002004	1.973338	5.900736	1.928916
O	-0.005444	1.972912	9.804384	1.982418
O	-0.002146	5.917585	5.901024	1.928721
O	-0.005791	5.919241	9.805728	1.982504
O	0.000000	5.917695	1.972576	1.922790
O	-0.001326	9.863409	5.904608	1.927710
O	-0.004119	9.863362	9.797056	1.981974
O	0.000000	9.862825	1.972576	1.922878
O	-0.004513	13.808302	9.801376	1.982348
O	0.000000	13.807955	1.972576	1.922805
O	-0.001815	13.808097	5.901440	1.928487
O	11.835390	1.972565	1.972576	1.922846
O	11.833054	1.972439	5.902112	1.928339
O	11.829772	1.975137	9.803328	1.982123
O	11.833086	5.919226	5.901664	1.928503
O	11.829109	5.918926	9.805248	1.982738
O	11.835390	5.917695	1.972576	1.922841
O	11.833496	9.862730	5.900352	1.928752
O	11.831319	9.863724	9.808448	1.983767
O	11.835390	9.862825	1.972576	1.922725
O	11.830514	13.808918	9.804992	1.981780
O	11.835390	13.807955	1.972576	1.922802
O	11.833244	13.808760	5.901216	1.928276
O	13.804531	1.974553	7.860736	1.907136
O	13.797713	1.974285	11.794560	1.951934
O	13.805067	3.945588	5.900736	1.928755
O	13.803615	3.946093	9.803648	1.982206
O	13.807955	5.917695	3.945120	1.917442
O	13.802968	5.917443	7.861216	1.906400
O	13.792490	5.916827	11.795392	1.948186
O	13.807955	7.890260	1.972576	1.922828
O	13.806740	7.890875	5.901792	1.928303
O	13.803947	7.890591	9.803264	1.982753
O	13.800728	9.864119	7.861248	1.908019
O	13.808239	9.861357	11.793664	1.961762
O	13.807955	9.862825	0.000000	1.519828
O	13.807955	9.862825	3.945120	1.918265
O	13.802527	0.001625	9.801088	1.982204
O	13.807955	11.835390	1.972576	1.922800
O	13.805888	11.835485	5.901152	1.928502
O	13.803647	11.836637	9.805440	1.981682
O	13.803852	13.815498	11.793984	1.948068

154	O	13.807955	13.807955	0.000000	1.519902
155	O	13.807955	13.807955	3.945120	1.917603
156	O	13.803568	13.808018	7.860864	1.906505
157	O	13.807955	1.972565	3.945120	1.917839
158	O	13.807955	1.972565	0.000000	1.519879
159	O	13.807955	3.945130	1.972576	1.922771
160	O	13.807955	5.917695	0.000000	1.519871
161	O	13.807955	0.000000	1.972576	1.922838
162	O	13.806156	0.000458	5.901696	1.928487
163	Pd	1.955333	1.995115	13.963904	3.090098
164	Pd	1.938621	3.949975	16.122624	2.965274
165	Pd	1.917728	5.894466	13.966112	3.110665
166	Pd	1.919053	7.877162	16.166048	2.941821
167	Pd	1.932277	9.880057	13.962112	3.109243
168	Pd	1.940815	0.015402	16.158976	2.952091
169	Pd	1.922572	11.836400	16.125312	2.973814
170	Pd	1.927243	13.811506	13.967744	3.094437
171	Pd	3.912417	1.982286	16.124640	2.963914
172	Pd	3.929697	3.971973	14.394144	3.488298
173	Pd	3.926493	5.931692	16.151040	2.954955
174	Pd	3.833073	7.877604	14.379168	3.474495
175	Pd	3.880288	9.852078	16.126304	2.961793
176	Pd	3.895895	-0.013934	14.397120	3.501916
177	Pd	3.904953	11.847320	14.401888	3.497835
178	Pd	3.903596	13.808839	16.161824	2.918252
179	Pd	5.857713	1.956232	13.966880	3.110608
180	Pd	5.888990	3.968927	16.151584	2.953412
181	Pd	5.897480	5.935085	13.944128	3.065288
182	Pd	5.877676	7.885336	16.062688	3.032776
183	Pd	5.844331	9.862825	13.970272	3.072091
184	Pd	5.870511	-0.004403	16.130080	2.981452
185	Pd	5.877502	11.794029	16.150816	2.942434
186	Pd	5.868223	13.812658	13.971648	3.080820
187	Pd	7.837648	1.960730	16.166496	2.942962
188	Pd	7.835943	3.876879	14.382656	3.478062
189	Pd	7.849957	5.921687	16.064000	3.029616
190	Pd	7.901054	7.944403	14.393440	3.379982
191	Pd	7.920227	9.893976	16.266304	3.342038
192	Pd	7.857310	0.013540	14.399680	3.480501
193	Pd	7.833166	11.802346	14.387264	3.441653
194	Pd	7.835139	13.792127	16.106144	2.982851
195	Pd	9.841348	1.970577	13.963200	3.108292
196	Pd	9.810339	3.916141	16.130208	2.962273
197	Pd	9.821922	5.881068	13.971744	3.075495
198	Pd	9.846745	7.968626	16.271360	3.338023
199	Pd	9.862557	9.902750	13.973120	3.079932
200	Pd	9.824620	-0.011330	16.144704	2.943686
201	Pd	9.851242	11.893352	16.092704	2.980680
202	Pd	9.826924	13.804925	13.956800	3.090515
203	Pd	15.753504	1.984321	16.159904	2.952805
204	Pd	15.720885	3.937808	14.395680	3.500372
205	Pd	15.737124	5.912188	16.128192	2.981163
206	Pd	15.750774	7.900659	14.399392	3.478117
207	Pd	15.730433	9.866865	16.144544	2.940713
208	Pd	-0.014550	0.029841	14.396224	3.501282
209	Pd	15.718897	11.839872	14.394208	3.487678
210	Pd	15.760068	13.828249	16.132480	2.978794
211	Pd	11.797485	1.964470	16.126624	2.975480
212	Pd	11.802756	3.948775	14.404640	3.498996
213	Pd	11.750112	5.921798	16.153152	2.937501
214	Pd	11.753268	7.875821	14.388448	3.440813
215	Pd	11.853348	9.894654	16.091456	2.984023
216	Pd	11.797832	15.761899	14.395136	3.488848
217	Pd	11.856994	11.902205	14.383808	3.484822
218	Pd	11.819688	13.830237	16.177920	2.955671
219	Pd	13.770508	1.965306	13.968224	3.095256
220	Pd	13.764795	3.944641	16.162944	2.917137
221	Pd	13.771928	5.907517	13.972192	3.079382
222	Pd	13.748352	7.874274	16.104224	2.981255
223	Pd	13.763817	9.866644	13.956864	3.088021
224	Pd	13.785594	0.018826	16.134208	2.978381
225	Pd	13.786588	11.861081	16.176544	2.955623

226	Pd	13.810180	13.848685	13.971392	3.121556
227	Sr	0.000000	0.000000	0.000000	1.317059
228	Sr	3.945130	0.000000	0.000000	1.317007
229	Sr	3.945130	3.945130	0.000000	1.316947
230	Sr	3.945130	3.945130	3.945120	1.574417
231	Sr	3.942684	3.944735	7.811008	1.577659
232	Sr	3.942037	3.944294	11.544576	1.824640
233	Sr	3.945130	7.890260	0.000000	1.316927
234	Sr	3.945130	7.890260	3.945120	1.574657
235	Sr	3.943236	7.891238	7.810432	1.578117
236	Sr	3.947323	7.886773	11.542176	1.829584
237	Sr	3.945130	0.000000	3.945120	1.574604
238	Sr	3.942858	0.000032	7.811552	1.577935
239	Sr	3.942368	0.001578	11.546880	1.823512
240	Sr	3.945130	11.835390	0.000000	1.316988
241	Sr	3.945130	11.835390	3.945120	1.574517
242	Sr	3.943915	11.836763	7.812416	1.577438
243	Sr	3.943946	11.838625	11.549856	1.820469
244	Sr	7.890260	0.000000	0.000000	1.317007
245	Sr	7.890260	3.945130	0.000000	1.316920
246	Sr	7.890260	3.945130	3.945120	1.574584
247	Sr	7.889187	3.945351	7.810688	1.577914
248	Sr	7.884374	3.949154	11.542848	1.828768
249	Sr	7.890260	7.890260	0.000000	1.316906
250	Sr	7.890260	7.890260	3.945120	1.574294
251	Sr	7.889218	7.891191	7.812864	1.577459
252	Sr	7.887167	7.889361	11.552704	1.821831
253	Sr	7.890260	0.000000	3.945120	1.574416
254	Sr	7.888130	0.000821	7.811808	1.577782
255	Sr	7.889124	0.001783	11.548384	1.820959
256	Sr	7.890260	11.835390	0.000000	1.316984
257	Sr	7.890260	11.835390	3.945120	1.574347
258	Sr	7.888477	11.835958	7.811808	1.577950
259	Sr	7.888193	11.839288	11.545184	1.824752
260	Sr	0.000000	3.945130	0.000000	1.317021
261	Sr	0.000000	3.945130	3.945120	1.574598
262	Sr	-0.002272	3.945020	7.811616	1.577980
263	Sr	-0.000316	3.944562	11.546496	1.823733
264	Sr	0.000000	7.890260	0.000000	1.317009
265	Sr	0.000000	7.890260	3.945120	1.574286
266	Sr	-0.001657	7.890134	7.811840	1.577745
267	Sr	-0.000221	7.891459	11.548672	1.820814
268	Sr	0.000000	0.000000	3.945120	1.574511
269	Sr	-0.001799	0.000521	7.811616	1.577965
270	Sr	-0.003156	-0.000899	11.546176	1.824005
271	Sr	0.000000	11.835390	0.000000	1.317037
272	Sr	0.000000	11.835390	3.945120	1.574333
273	Sr	-0.001436	11.836905	7.811584	1.577802
274	Sr	0.000158	11.835374	11.546208	1.824688
275	Sr	11.835390	0.000000	0.000000	1.317044
276	Sr	11.835390	3.945130	0.000000	1.316982
277	Sr	11.835390	3.945130	3.945120	1.574353
278	Sr	11.834412	3.946156	7.812960	1.577472
279	Sr	11.836495	3.945667	11.551200	1.819165
280	Sr	11.835390	7.890260	0.000000	1.316980
281	Sr	11.835390	7.890260	3.945120	1.574406
282	Sr	11.833765	7.890544	7.812192	1.577790
283	Sr	11.837757	7.889929	11.546048	1.823366
284	Sr	11.835390	0.000000	3.945120	1.574303
285	Sr	11.834633	0.000884	7.811680	1.577618
286	Sr	11.832976	0.001862	11.546400	1.824873
287	Sr	11.835390	11.835390	0.000000	1.317037
288	Sr	11.835390	11.835390	3.945120	1.574592
289	Sr	11.833717	11.835816	7.811584	1.577598
290	Sr	11.832218	11.834285	11.542784	1.827434
291	Ti	1.972565	1.972565	1.972576	2.637382
292	Ti	1.971776	1.972723	5.898656	2.621094
293	Ti	1.969172	1.972628	9.839488	2.649252
294	Ti	1.971666	5.917979	5.899040	2.621080
295	Ti	1.969409	5.917853	9.840064	2.648624
296	Ti	1.972565	5.917695	1.972576	2.637390
297	Ti	1.972013	9.863393	5.899168	2.620539

```
Ti       1.970419       9.863425       9.840992        2.648651
Ti       1.972565       9.862825       1.972576        2.637367
Ti       1.969898      13.808507       9.840736        2.648925
Ti       1.972565      13.807955       1.972576        2.637385
Ti       1.972028      13.808444       5.898944        2.621122
Ti       5.917695       1.972565       1.972576        2.637383
Ti       5.917158       1.972628       5.899040        2.620902
Ti       5.914334       1.972912       9.840192        2.648846
Ti       5.917411       5.918232       5.897792        2.621211
Ti       5.915012       5.918405       9.835424        2.651315
Ti       5.917695       5.917695       1.972576        2.637394
Ti       5.916748       9.863409       5.899072        2.620658
Ti       5.916575       9.863803       9.842080        2.646939
Ti       5.917695       9.862825       1.972576        2.637378
Ti       5.914507      13.809123       9.842016        2.648562
Ti       5.917695      13.807955       1.972576        2.637382
Ti       5.917016      13.808413       5.899360        2.620970
Ti       9.862462       1.973023       5.899232        2.620546
Ti       9.860016       1.973938       9.841472        2.648020
Ti       9.862541       5.917648       5.899264        2.619770
Ti       9.860489       5.919731       9.842784        2.648156
Ti       9.862825       5.917695       1.972576        2.637373
Ti       9.862241       9.863046       5.899648        2.620265
Ti       9.860284       9.863472       9.842976        2.647052
Ti       9.862825       9.862825       1.972576        2.637359
Ti       9.859827      13.810227       9.838816        2.650396
Ti       9.862825      13.807955       1.972576        2.637365
Ti       9.862225      13.807829       5.898656        2.620616
Ti       9.862825       1.972565       1.972576        2.637374
Ti      13.807482       1.973023       5.899072        2.620953
Ti      13.804799       1.973291       9.840992        2.648824
Ti      13.807418       5.917853       5.899456        2.620714
Ti      13.805509       5.917821       9.842336        2.648793
Ti      13.807955       5.917695       1.972576        2.637383
Ti      13.806819       9.863125       5.898688        2.620846
Ti      13.806929       9.863141       9.839168        2.649449
Ti      13.807955       9.862825       1.972576        2.637374
Ti      13.805462      13.808933       9.841792        2.648083
Ti      13.807955      13.807955       1.972576        2.637385
Ti      13.807403      13.808350       5.899264        2.620863
Ti      13.807955       1.972565       1.972576        2.637382
C        8.805215       8.856233      17.748352        3.979761
H        7.457132       7.515615      17.969824        1.015673
H        9.443331       9.494776      18.382016        0.979191
O        8.032979       8.089521      18.553536        2.371450
```

1.1.14 CO_2

```
     339
jmolscript: load "" {1 1 1} spacegroup "x,y,z" unitcell [{      15.780520
      0.000000      0.000000 }, {       0.000000     15.780520      0.000000
   }, {       0.000000      0.000000     32.000000 }]
O        1.972565       1.972565       3.945120        1.917609
O        1.969567       1.973717       7.861760        1.906783
O        1.965274       1.973023      11.795584        1.952710
O        1.972565       1.972565       0.000000        1.519760
O        1.972565       3.945130       1.972576        1.922778
O        1.971287       3.945461       5.901632        1.928605
O        1.969014       3.946014       9.805216        1.981674
O        1.972565       5.917695       3.945120        1.917552
O        1.969756       5.918074       7.862048        1.906575
O        1.965101       5.918989      11.796352        1.952791
O        1.972565       5.917695       0.000000        1.519742
O        1.972565       7.890260       1.972576        1.922832
O        1.970908       7.890465       5.902080        1.928182
O        1.969093       7.890986       9.805248        1.981818
O        1.969646       9.863488       7.861984        1.906302
```

O	1.965322	9.864703	11.796256	1.952803
O	1.972565	9.862825	0.000000	1.519727
O	1.972565	9.862825	3.945120	1.917732
O	1.972565	0.000000	1.972576	1.922867
O	1.970892	0.000284	5.902208	1.928294
O	1.968983	0.000884	9.803616	1.982413
O	1.972565	11.835390	1.972576	1.922808
O	1.971318	11.835564	5.901664	1.928626
O	1.969030	11.836211	9.805152	1.981811
O	1.965416	13.810906	11.795616	1.953118
O	1.972565	13.807955	0.000000	1.519749
O	1.972565	13.807955	3.945120	1.917776
O	1.969677	13.807987	7.861696	1.906629
O	3.945130	1.972565	1.972576	1.922809
O	3.943757	1.972675	5.901696	1.928250
O	3.941374	1.973370	9.804768	1.981901
O	3.943773	5.918137	5.901760	1.928299
O	3.941311	5.918658	9.805056	1.982009
O	3.945130	5.917695	1.972576	1.922829
O	3.943741	9.862888	5.901728	1.928281
O	3.941280	9.863346	9.804768	1.981996
O	3.945130	9.862825	1.972576	1.922821
O	3.941343	13.808681	9.804704	1.982006
O	3.945130	13.807955	1.972576	1.922801
O	3.943757	13.808413	5.901568	1.928221
O	5.917695	1.972565	3.945120	1.917675
O	5.914712	1.972770	7.861888	1.906626
O	5.911051	1.975200	11.796032	1.952766
O	5.917695	3.945130	1.972576	1.922808
O	5.915975	3.945446	5.901984	1.928174
O	5.914050	3.946093	9.804128	1.982058
O	5.917695	5.917695	3.945120	1.917492
O	5.914523	5.918863	7.861984	1.906758
O	5.910152	5.918389	11.796384	1.950497
O	5.917695	5.917695	0.000000	1.519788
O	5.917695	7.890260	1.972576	1.922801
O	5.916401	7.890481	5.901472	1.928595
O	5.914334	7.890986	9.805568	1.981885
O	5.914350	9.862746	7.861824	1.906702
O	5.910073	9.865460	11.796064	1.951918
O	5.917695	9.862825	0.000000	1.519770
O	5.917695	9.862825	3.945120	1.917705
O	5.916369	0.000316	5.901504	1.928582
O	5.914034	0.000963	9.805312	1.981860
O	5.917695	11.835390	1.972576	1.922836
O	5.916006	11.835627	5.902144	1.928293
O	5.913955	11.836226	9.803904	1.982249
O	5.910909	13.809091	11.796000	1.952710
O	5.917695	13.807955	0.000000	1.519783
O	5.917695	13.807955	3.945120	1.917830
O	5.914570	13.809170	7.861792	1.906602
O	5.917695	1.972565	0.000000	1.519800
O	5.917695	0.000000	1.972576	1.922836
O	7.890260	1.972565	1.972576	1.922790
O	7.888745	1.973054	5.901760	1.928218
O	7.886268	1.973354	9.804640	1.981798
O	7.888745	5.917774	5.901760	1.928169
O	7.886157	5.918168	9.804480	1.982130
O	7.890260	5.917695	1.972576	1.922809
O	7.888745	9.863298	5.901664	1.928115
O	7.886062	9.863851	9.803776	1.982290
O	7.890260	9.862825	1.972576	1.922801
O	7.886236	13.808728	9.804416	1.981756
O	7.890260	13.807955	1.972576	1.922798
O	7.888761	13.808065	5.901728	1.928218
O	9.862825	1.972565	3.945120	1.917676
O	9.859748	1.973244	7.861920	1.905981
O	9.855503	1.975469	11.796288	1.951547
O	9.861563	3.945335	5.901344	1.928607
O	9.859385	3.946077	9.804736	1.981934
O	9.862825	5.917695	3.945120	1.917970
O	9.859874	5.917443	7.861280	1.907282

O	9.856245	5.921340	11.794176	1.955948
O	9.862825	5.917695	0.000000	1.519808
O	9.862825	7.890260	1.972576	1.922801
O	9.861168	7.890497	5.901792	1.928024
O	9.859306	7.891065	9.801856	1.983044
O	9.859842	9.864261	7.860992	1.907302
O	9.855787	9.863314	11.793408	1.957501
O	9.862825	9.862825	0.000000	1.519789
O	9.862825	9.862825	3.945120	1.918303
O	9.859353	0.000947	9.805248	1.981861
O	9.862825	11.835390	1.972576	1.922790
O	9.861563	11.835769	5.901408	1.928501
O	9.859369	11.836337	9.803968	1.981941
O	9.855408	13.808523	11.795936	1.951964
O	9.862825	13.807955	0.000000	1.519800
O	9.862825	13.807955	3.945120	1.917946
O	9.859811	13.808807	7.861760	1.905960
O	9.862825	1.972565	0.000000	1.519818
O	9.862825	3.945130	1.972576	1.922775
O	9.862825	0.000000	1.972576	1.922858
O	9.861136	0.000347	5.901824	1.928410
O	0.000000	1.972565	1.972576	1.922852
O	-0.001389	1.973086	5.901760	1.928320
O	-0.003992	1.973354	9.804768	1.982046
O	-0.001294	5.917727	5.901696	1.928460
O	-0.003803	5.918658	9.804672	1.982062
O	0.000000	5.917695	1.972576	1.922837
O	-0.001341	9.863267	5.901728	1.928323
O	-0.003819	9.863330	9.804672	1.982061
O	0.000000	9.862825	1.972576	1.922822
O	-0.003992	13.808618	9.804704	1.982156
O	0.000000	13.807955	1.972576	1.922811
O	-0.001373	13.808002	5.901600	1.928354
O	11.835390	1.972565	1.972576	1.922817
O	11.833970	1.972628	5.901952	1.928245
O	11.831319	1.973370	9.804576	1.982018
O	11.833970	5.918168	5.901824	1.928282
O	11.831650	5.918168	9.804000	1.982264
O	11.835390	5.917695	1.972576	1.922816
O	11.833986	9.862888	5.901728	1.928260
O	11.831666	9.863772	9.803808	1.982251
O	11.835390	9.862825	1.972576	1.922804
O	11.831366	13.808697	9.804448	1.981950
O	11.835390	13.807955	1.972576	1.922802
O	11.833986	13.808476	5.901792	1.928331
O	13.805115	1.972675	7.861856	1.906336
O	13.799844	1.974727	11.796064	1.953145
O	13.806298	3.945414	5.902016	1.928178
O	13.804515	3.946029	9.804064	1.982062
O	13.807955	5.917695	3.945120	1.917709
O	13.805399	5.918926	7.861792	1.906387
O	13.801343	5.918374	11.796128	1.951716
O	13.807955	7.890260	1.972576	1.922790
O	13.806740	7.890512	5.901344	1.928561
O	13.804294	7.891096	9.805536	1.981668
O	13.805383	9.862762	7.861760	1.906155
O	13.801422	9.865997	11.795968	1.952088
O	13.807955	9.862825	0.000000	1.519741
O	13.807955	9.862825	3.945120	1.917926
O	13.804420	0.000821	9.805216	1.981909
O	13.807955	11.835390	1.972576	1.922824
O	13.806314	11.835642	5.902080	1.928143
O	13.804483	11.836242	9.803680	1.982207
O	13.800049	13.808807	11.796128	1.952670
O	13.807955	13.807955	0.000000	1.519753
O	13.807955	13.807955	3.945120	1.917988
O	13.805051	13.809170	7.861760	1.906170
O	13.807955	1.972565	3.945120	1.917911
O	13.807955	1.972565	0.000000	1.519759
O	13.807955	3.945130	1.972576	1.922795
O	13.807955	5.917695	0.000000	1.519754
O	13.807955	0.000000	1.972576	1.922818

162	O	13.806756	0.000316	5.901472	1.928728
163	Pd	1.945580	1.970624	13.967648	3.096628
164	Pd	1.941351	3.946834	16.152000	2.949232
165	Pd	1.944665	5.929625	13.967840	3.092394
166	Pd	1.941635	7.896399	16.126240	2.968977
167	Pd	1.946117	9.863977	13.967712	3.092639
168	Pd	1.942156	0.006770	16.126560	2.969973
169	Pd	1.942677	11.847525	16.151904	2.950315
170	Pd	1.945707	13.823814	13.967392	3.096496
171	Pd	3.918414	1.978940	16.140288	2.959256
172	Pd	3.901607	3.949470	14.397344	3.492401
173	Pd	3.913490	5.921356	16.140128	2.957915
174	Pd	3.927266	7.896635	14.399712	3.491629
175	Pd	3.914184	9.873177	16.138432	2.959837
176	Pd	3.927693	0.007796	14.396672	3.488587
177	Pd	3.902491	11.846642	14.396960	3.491673
178	Pd	3.919282	13.817344	16.140480	2.957850
179	Pd	5.893109	1.987983	13.967584	3.093315
180	Pd	5.887728	3.952058	16.126656	2.965473
181	Pd	5.884367	5.913939	13.969536	3.101027
182	Pd	5.880453	7.897014	16.162784	2.896402
183	Pd	5.885029	9.878353	13.968256	3.100367
184	Pd	5.890347	0.008411	16.148896	2.951249
185	Pd	5.887570	11.841055	16.126336	2.966387
186	Pd	5.893772	13.807781	13.967840	3.093396
187	Pd	7.864838	1.983122	16.137600	2.961846
188	Pd	7.873596	3.958812	14.398752	3.490064
189	Pd	7.866889	5.930193	16.142944	2.931748
190	Pd	7.822893	7.895878	14.390592	3.485154
191	Pd	7.866037	9.863914	16.139936	2.939371
192	Pd	7.852513	0.008427	14.397472	3.491917
193	Pd	7.871639	11.837536	14.398016	3.490123
194	Pd	7.863465	13.813273	16.137152	2.960710
195	Pd	9.835540	1.974080	13.969248	3.094048
196	Pd	9.832242	3.960579	16.156480	2.959588
197	Pd	9.835540	5.931566	13.963552	3.090749
198	Pd	9.832305	7.899855	16.081952	3.034967
199	Pd	9.835020	9.862036	13.962144	3.089026
200	Pd	9.832069	0.006454	16.131136	2.967629
201	Pd	9.831548	11.834364	16.153312	2.958259
202	Pd	9.834751	13.820027	13.968704	3.093428
203	Pd	15.749227	1.977709	16.141504	2.957429
204	Pd	15.761725	3.947513	14.397120	3.491937
205	Pd	15.753756	5.921593	16.137408	2.960693
206	Pd	15.737045	7.898450	14.398784	3.489725
207	Pd	15.755161	9.874045	16.138816	2.960749
208	Pd	15.737060	0.008190	14.396864	3.488763
209	Pd	-0.017374	11.846547	14.397088	3.492300
210	Pd	15.750647	13.817597	16.140448	2.958277
211	Pd	11.804113	1.981876	16.137344	2.960803
212	Pd	11.790226	3.958465	14.399232	3.491577
213	Pd	11.804618	5.931645	16.141504	2.924418
214	Pd	11.845710	7.897740	14.390112	3.483693
215	Pd	11.803797	9.864466	16.141280	2.931594
216	Pd	11.811514	0.006423	14.397152	3.490938
217	Pd	11.791236	11.836716	14.398432	3.490650
218	Pd	11.804113	13.812326	16.137664	2.959828
219	Pd	13.777088	1.986294	13.967360	3.093321
220	Pd	13.776157	3.950637	16.125824	2.967121
221	Pd	13.785610	5.913955	13.968864	3.101711
222	Pd	13.785468	7.899081	16.163392	2.907629
223	Pd	13.786004	9.880767	13.968320	3.102165
224	Pd	13.773553	0.006186	16.148832	2.950151
225	Pd	13.776930	11.842144	16.126272	2.966270
226	Pd	13.778351	13.806645	13.968096	3.092674
227	Sr	0.000000	0.000000	0.000000	1.316945
228	Sr	3.945130	0.000000	0.000000	1.316937
229	Sr	3.945130	3.945130	0.000000	1.316912
230	Sr	3.945130	3.945130	3.945120	1.574330
231	Sr	3.943883	3.945240	7.812320	1.577544
232	Sr	3.944593	3.945398	11.547456	1.822904
233	Sr	3.945130	7.890260	0.000000	1.316896

234	Sr	3.945130	7.890260	3.945120	1.574327
235	Sr	3.943820	7.890339	7.812384	1.577673
236	Sr	3.944073	7.890512	11.548576	1.821154
237	Sr	3.945130	0.000000	3.945120	1.574435
238	Sr	3.943789	0.000174	7.812288	1.577608
239	Sr	3.943568	0.000047	11.547360	1.822846
240	Sr	3.945130	11.835390	0.000000	1.316917
241	Sr	3.945130	11.835390	3.945120	1.574411
242	Sr	3.943836	11.835611	7.812192	1.577541
243	Sr	3.944735	11.835311	11.547200	1.823145
244	Sr	7.890260	0.000000	0.000000	1.316987
245	Sr	7.890260	3.945130	0.000000	1.316968
246	Sr	7.890260	3.945130	3.945120	1.574332
247	Sr	7.888871	3.945146	7.812224	1.577560
248	Sr	7.888508	3.944893	11.547872	1.821890
249	Sr	7.890260	7.890260	0.000000	1.316963
250	Sr	7.890260	7.890260	3.945120	1.574391
251	Sr	7.888729	7.890276	7.811648	1.577565
252	Sr	7.891649	7.890544	11.544832	1.824817
253	Sr	7.890260	0.000000	3.945120	1.574431
254	Sr	7.888982	0.000237	7.812160	1.577367
255	Sr	7.889487	-0.000032	11.547488	1.822694
256	Sr	7.890260	11.835390	0.000000	1.316970
257	Sr	7.890260	11.835390	3.945120	1.574404
258	Sr	7.888824	11.835753	7.811904	1.577534
259	Sr	7.888556	11.835832	11.547200	1.822063
260	Sr	0.000000	3.945130	0.000000	1.316919
261	Sr	0.000000	3.945130	3.945120	1.574452
262	Sr	-0.001199	3.945240	7.812288	1.577674
263	Sr	-0.001483	3.945603	11.547200	1.822900
264	Sr	0.000000	7.890260	0.000000	1.316890
265	Sr	0.000000	7.890260	3.945120	1.574379
266	Sr	-0.001168	7.890386	7.812320	1.577546
267	Sr	-0.000947	7.890339	11.548256	1.821667
268	Sr	0.000000	0.000000	3.945120	1.574464
269	Sr	-0.001152	0.000158	7.812256	1.577492
270	Sr	-0.000505	-0.000032	11.547296	1.823081
271	Sr	0.000000	11.835390	0.000000	1.316906
272	Sr	0.000000	11.835390	3.945120	1.574501
273	Sr	-0.001215	11.835532	7.812256	1.577689
274	Sr	-0.001531	11.835264	11.547264	1.823132
275	Sr	11.835390	0.000000	0.000000	1.316976
276	Sr	11.835390	3.945130	0.000000	1.316954
277	Sr	11.835390	3.945130	3.945120	1.574402
278	Sr	11.834254	3.945177	7.812192	1.577497
279	Sr	11.835374	3.944972	11.548000	1.821868
280	Sr	11.835390	7.890260	0.000000	1.316944
281	Sr	11.835390	7.890260	3.945120	1.574472
282	Sr	11.834427	7.890323	7.811680	1.577661
283	Sr	11.831950	7.890339	11.544512	1.825296
284	Sr	11.835390	0.000000	3.945120	1.574488
285	Sr	11.834128	0.000237	7.812160	1.577478
286	Sr	11.834396	0.000032	11.547488	1.822835
287	Sr	11.835390	11.835390	0.000000	1.316958
288	Sr	11.835390	11.835390	3.945120	1.574493
289	Sr	11.834396	11.835674	7.812000	1.577415
290	Sr	11.835153	11.835911	11.547584	1.822162
291	Ti	1.972565	1.972565	1.972576	2.637382
292	Ti	1.971981	1.972565	5.899232	2.620661
293	Ti	1.970514	1.972786	9.841312	2.647822
294	Ti	1.971997	5.917663	5.899328	2.620710
295	Ti	1.970592	5.918026	9.841440	2.647704
296	Ti	1.972565	5.917695	1.972576	2.637386
297	Ti	1.972013	9.862935	5.899168	2.620724
298	Ti	1.970592	9.863188	9.841568	2.647747
299	Ti	1.972565	9.862825	1.972576	2.637385
300	Ti	1.970514	13.808271	9.841280	2.648119
301	Ti	1.972565	13.807955	1.972576	2.637380
302	Ti	1.971981	13.807955	5.899072	2.620548
303	Ti	5.917695	1.972565	1.972576	2.637385
304	Ti	5.917143	1.972470	5.899168	2.620699
305	Ti	5.915722	1.972865	9.841248	2.647832

```
Ti        5.917064       5.917711       5.899360        2.620593
Ti        5.915691       5.918090       9.841344        2.647739
Ti        5.917695       5.917695       1.972576        2.637383
Ti        5.917048       9.862825       5.899136        2.620639
Ti        5.915675       9.863125       9.841152        2.647964
Ti        5.917695       9.862825       1.972576        2.637384
Ti        5.915659      13.808318       9.841184        2.647758
Ti        5.917695      13.807955       1.972576        2.637377
Ti        5.917111      13.808065       5.899040        2.620695
Ti        9.862383       1.972549       5.899136        2.620622
Ti        9.861010       1.973038       9.841856        2.647649
Ti        9.862399       5.917553       5.898912        2.620849
Ti        9.861010       5.917506       9.840128        2.648914
Ti        9.862825       5.917695       1.972576        2.637383
Ti        9.862399       9.862935       5.898624        2.620944
Ti        9.861026       9.863677       9.839712        2.649433
Ti        9.862825       9.862825       1.972576        2.637389
Ti        9.860994      13.808207       9.841600        2.647551
Ti        9.862825      13.807955       1.972576        2.637370
Ti        9.862430      13.808002       5.898944        2.620666
Ti        9.862825       1.972565       1.972576        2.637383
Ti       13.807497       1.972470       5.898976        2.620486
Ti       13.806014       1.972817       9.841248        2.647693
Ti       13.807576       5.917711       5.899136        2.620587
Ti       13.806109       5.918090       9.841376        2.647545
Ti       13.807955       5.917695       1.972576        2.637384
Ti       13.807592       9.862841       5.898944        2.620502
Ti       13.806124       9.863204       9.841472        2.647635
Ti       13.807955       9.862825       1.972576        2.637378
Ti       13.806014      13.808286       9.841440        2.647733
Ti       13.807955      13.807955       1.972576        2.637369
Ti       13.807513      13.808065       5.898912        2.620520
Ti       13.807955       1.972565       1.972576        2.637369
C         9.626717       7.738088      19.264672        4.174112
O         8.448906       7.733828      19.289600        2.175888
O        10.804701       7.741576      19.290880        2.175083
```

1.2 TiO_2–Pd interface

1.2.1 CH_2O

```
      356
jmolscript: load "" {1 1 1} spacegroup "x,y,z" unitcell [{      15.780000
       0.000000      0.000000 }, {       0.000000     15.780000       0.000000
   }, {       0.000000      0.000000     32.000000 }]
C         8.428619      11.159853      17.737664        3.983540
H         7.411582      10.755885      17.951712        1.005389
H         8.654541      12.164770      18.153856        0.979182
O        15.708327       1.963947       7.856608        1.904658
O        15.706875       9.853884       7.857440        1.904868
O         0.062110       5.907811      11.689696        2.233792
O         0.061005      13.798931      11.686720        2.237461
O         0.101844       1.963600      11.690880        2.239086
O         0.102696       9.853269      11.693280        2.242986
O         0.081235       3.936731       5.902240        1.940377
O         0.079894      11.826321       5.903200        1.938252
O         0.080588       7.880500       5.902816        1.939313
O         0.082261      -0.009831       5.902272        1.940244
O         0.082151      -0.006060       9.812704        1.982479
O         0.081961       7.883609       9.813856        1.981444
O         0.081993       1.962495       0.004992        1.690762
O         0.081993       1.962495       3.950112        1.911936
O         0.081993       3.935106       1.977504        1.897493
O         0.081993       5.907606       0.004992        1.691127
O         0.081993       5.907606       3.950112        1.911980
```

O	0.081993	7.879901	1.977504	1.897537
O	0.081993	9.852495	0.004992	1.690754
O	0.081993	9.852495	3.950112	1.911856
O	0.081993	11.825106	1.977504	1.897438
O	0.081993	13.797606	0.004992	1.691169
O	0.081993	13.797606	3.950112	1.912039
O	0.081993	-0.010099	1.977504	1.897565
O	0.083429	3.934569	9.812992	1.981626
O	0.085165	11.823007	9.814176	1.980673
O	0.236132	5.909137	7.859264	1.904363
O	0.235990	13.798395	7.859328	1.904482
O	2.055519	15.751470	11.688416	2.234676
O	2.054319	3.952858	11.690496	2.237906
O	2.053941	7.860539	11.689472	2.238663
O	2.054840	11.847261	11.689216	2.242831
O	2.054603	3.935106	0.004992	1.690695
O	2.054603	3.935106	3.950112	1.911475
O	2.054603	7.879901	0.004992	1.690701
O	2.054603	7.879901	3.950112	1.911439
O	2.054603	11.825106	0.004992	1.690695
O	2.054603	11.825106	3.950112	1.911390
O	2.054603	-0.010099	0.004992	1.690740
O	2.054603	-0.010099	3.950112	1.911444
O	2.054619	3.782987	7.856512	1.904731
O	2.055392	11.671977	7.857984	1.903895
O	2.054745	0.144845	7.858208	1.904642
O	2.054619	8.036470	7.858048	1.903953
O	3.873738	5.909215	7.858080	1.904451
O	3.873548	13.798237	7.858528	1.904536
O	4.009146	1.962953	11.688736	2.234793
O	4.002092	9.853632	11.692896	2.238176
O	4.024373	3.936258	9.812512	1.982555
O	4.027482	11.826005	9.815392	1.981781
O	4.043499	5.908221	11.686784	2.239035
O	4.047428	13.798237	11.687936	2.239868
O	4.028208	-0.009184	9.812608	1.981311
O	4.026314	7.882173	9.813376	1.981792
O	4.027103	1.962495	0.004992	1.690738
O	4.027103	1.962495	3.950112	1.911925
O	4.027103	3.935106	1.977504	1.897477
O	4.027103	5.907606	0.004992	1.691134
O	4.027103	5.907606	3.950112	1.911941
O	4.027103	7.879901	1.977504	1.897388
O	4.027103	9.852495	0.004992	1.690746
O	4.027103	9.852495	3.950112	1.911688
O	4.027103	11.825106	1.977504	1.897462
O	4.027103	13.797606	0.004992	1.691184
O	4.027103	13.797606	3.950112	1.911950
O	4.027103	-0.010099	1.977504	1.897395
O	4.028618	7.881289	5.902848	1.939127
O	4.027624	-0.009610	5.902368	1.939474
O	4.028176	3.936084	5.902112	1.940420
O	4.029391	11.825769	5.902976	1.939803
O	4.181369	1.963821	7.857760	1.904755
O	4.183136	9.854436	7.859136	1.904127
O	5.999161	3.920068	11.686464	2.237581
O	6.001781	11.803724	11.695936	2.233664
O	6.000250	0.008584	11.688512	2.240476
O	6.000645	7.899515	11.687552	2.233919
O	5.999698	3.935106	0.004992	1.690877
O	5.999698	3.935106	3.950112	1.911521
O	5.999698	7.879901	0.004992	1.690881
O	5.999698	7.879901	3.950112	1.911854
O	5.999698	11.825106	0.004992	1.690886
O	5.999698	11.825106	3.950112	1.911545
O	5.999698	-0.010099	0.004992	1.690933
O	5.999698	-0.010099	3.950112	1.911431
O	5.999966	4.090602	7.860224	1.904231
O	6.000850	11.982022	7.863776	1.903845
O	6.000818	7.727450	7.862304	1.903995
O	6.000597	15.617166	7.858688	1.904163
O	7.818753	1.963458	7.856640	1.904494

O	7.815755	9.854736	7.855840	1.906013
O	7.957602	5.908363	11.684608	2.240227
O	7.956465	13.797006	11.693024	2.231389
O	7.987726	1.963663	11.689088	2.243850
O	7.994969	9.858839	11.717056	2.248250
O	7.971583	3.939193	5.903232	1.938417
O	7.971235	11.827931	5.904096	1.938523
O	7.972103	7.879569	5.904192	1.937993
O	7.972403	-0.012151	5.903008	1.939891
O	7.972561	-0.006833	9.814688	1.982768
O	7.977121	7.896849	9.821088	1.976723
O	7.971993	1.962495	0.004992	1.690764
O	7.971993	1.962495	3.950112	1.911702
O	7.971993	3.935106	1.977504	1.897445
O	7.971993	5.907606	0.004992	1.691112
O	7.971993	5.907606	3.950112	1.911984
O	7.971993	7.879901	1.977504	1.897573
O	7.971993	9.852495	0.004992	1.690759
O	7.971993	9.852495	3.950112	1.911695
O	7.971993	11.825106	1.977504	1.897465
O	7.971993	13.797606	0.004992	1.691180
O	7.971993	13.797606	3.950112	1.911906
O	7.971993	-0.010099	1.977504	1.897559
O	7.975338	3.932029	9.813664	1.981355
O	7.978384	11.816411	9.820992	1.976845
O	8.126321	5.911204	7.866944	1.903161
O	8.127221	13.797716	7.865568	1.903319
O	9.415200	10.296055	17.729504	2.390076
O	9.944903	15.758665	11.690848	2.240034
O	9.944998	3.952148	11.692416	2.241644
O	9.942299	7.868776	11.708736	2.222092
O	9.939396	11.839119	11.711136	2.247275
O	9.944603	3.935106	0.004992	1.690684
O	9.944603	3.935106	3.950112	1.911277
O	9.944603	7.879901	0.004992	1.690676
O	9.944603	7.879901	3.950112	1.911347
O	9.944603	11.825106	0.004992	1.690682
O	9.944603	11.825106	3.950112	1.911336
O	9.944603	-0.010099	0.004992	1.690742
O	9.944603	-0.010099	3.950112	1.910986
O	9.944872	3.783113	7.856384	1.904720
O	9.944240	11.671046	7.857888	1.904539
O	9.944540	0.145665	7.858176	1.904211
O	9.944209	8.037638	7.859360	1.904460
O	11.762664	5.910383	7.861952	1.904437
O	11.762570	13.797716	7.862720	1.903727
O	11.900345	1.964137	11.689440	2.237470
O	11.891240	9.855462	11.709632	2.215725
O	11.915320	3.936637	9.813504	1.981883
O	11.905931	11.820609	9.820480	1.977975
O	11.932331	5.910462	11.688480	2.237952
O	11.934619	13.796943	11.685856	2.235662
O	11.917119	-0.007590	9.812992	1.982488
O	11.911154	7.887144	9.819200	1.977913
O	11.917103	1.962495	0.004992	1.690701
O	11.917103	1.962495	3.950112	1.911843
O	11.917103	3.935106	1.977504	1.897471
O	11.917103	5.907606	0.004992	1.691075
O	11.917103	5.907606	3.950112	1.911923
O	11.917103	7.879901	1.977504	1.897437
O	11.917103	9.852495	0.004992	1.690678
O	11.917103	9.852495	3.950112	1.911850
O	11.917103	11.825106	1.977504	1.897480
O	11.917103	13.797606	0.004992	1.691129
O	11.917103	13.797606	3.950112	1.912008
O	11.917103	-0.010099	1.977504	1.897402
O	11.916898	7.880642	5.903616	1.938115
O	11.916993	-0.010888	5.902720	1.939115
O	11.917750	3.937741	5.902528	1.939397
O	11.917750	11.826463	5.904000	1.938495
O	12.071211	1.964121	7.857504	1.904506
O	12.072536	9.854168	7.858496	1.905193

167	O	13.890282	3.917480	11.689856	2.233897
168	O	13.889824	11.802714	11.687392	2.240575
169	O	13.890171	0.010715	11.687904	2.237239
170	O	13.888041	7.902987	11.689536	2.236769
171	O	13.889698	3.935106	0.004992	1.690832
172	O	13.889698	3.935106	3.950112	1.911470
173	O	13.889698	7.879901	0.004992	1.690822
174	O	13.889698	7.879901	3.950112	1.911728
175	O	13.889698	11.825106	0.004992	1.690815
176	O	13.889698	11.825106	3.950112	1.911530
177	O	13.889698	-0.010099	0.004992	1.690878
178	O	13.889698	-0.010099	3.950112	1.911459
179	O	13.889430	4.090965	7.859648	1.904259
180	O	13.887899	11.980886	7.865152	1.904102
181	O	13.888404	7.727419	7.861920	1.904617
182	O	13.889619	15.617040	7.858240	1.904251
183	Pd	0.049912	0.034527	15.762816	2.920096
184	Pd	0.055404	7.920471	15.762144	2.914787
185	Pd	0.092313	1.961249	13.817120	3.508863
186	Pd	0.083965	9.854515	13.812256	3.496389
187	Pd	0.120796	3.889612	15.759456	2.917906
188	Pd	0.085401	5.901767	13.819616	3.500793
189	Pd	0.114263	11.790011	15.763232	2.908786
190	Pd	0.080588	13.805796	13.814208	3.494542
191	Pd	2.055550	-0.005775	13.820224	3.500811
192	Pd	2.047502	7.871111	13.816768	3.494371
193	Pd	2.056671	3.930104	13.817184	3.506478
194	Pd	2.049459	11.834621	13.813696	3.494014
195	Pd	2.008841	5.937335	15.785312	2.867050
196	Pd	2.007342	13.834768	15.772576	2.873956
197	Pd	2.104626	1.921278	15.785088	2.858259
198	Pd	2.095205	9.824738	15.773504	2.862314
199	Pd	4.016042	5.897807	13.813728	3.506951
200	Pd	4.025178	13.807579	13.814944	3.494016
201	Pd	4.023253	1.964815	13.820064	3.502302
202	Pd	4.022780	9.852811	13.813664	3.495027
203	Pd	3.991109	3.979022	15.762688	2.919035
204	Pd	3.985981	11.874955	15.765856	2.910155
205	Pd	4.064865	15.728131	15.768256	2.902861
206	Pd	4.040658	7.828916	15.764448	2.924001
207	Pd	5.992266	0.000252	13.815712	3.495210
208	Pd	5.973393	7.865951	13.816640	3.498437
209	Pd	5.993086	3.936637	13.814496	3.495100
210	Pd	5.979294	11.824206	13.825824	3.465589
211	Pd	6.033625	5.867399	15.774496	2.879350
212	Pd	6.045413	13.761280	15.767072	2.885705
213	Pd	5.955104	2.005354	15.773568	2.873365
214	Pd	5.929161	9.867849	15.766976	2.842837
215	Pd	7.930602	0.042559	15.766176	2.912660
216	Pd	7.928093	7.893235	15.763968	2.816576
217	Pd	7.968190	1.971963	13.813248	3.497902
218	Pd	7.965113	9.813393	13.828352	3.435965
219	Pd	8.003001	3.897755	15.762464	2.911159
220	Pd	7.974234	5.911519	13.812192	3.480589
221	Pd	8.010512	11.787013	15.790176	3.274590
222	Pd	7.969405	13.821497	13.825504	3.469266
223	Pd	9.944288	-0.002115	13.811520	3.494974
224	Pd	9.959736	7.872658	13.846688	3.492848
225	Pd	9.946481	3.935769	13.811968	3.496800
226	Pd	9.984022	11.851695	13.824064	3.442485
227	Pd	9.913028	5.922739	15.781568	2.849391
228	Pd	9.918724	13.864923	15.763104	2.846780
229	Pd	9.980203	1.929010	15.775008	2.863251
230	Pd	10.024245	9.794709	15.857088	3.158876
231	Pd	11.933751	5.894714	13.816256	3.495050
232	Pd	11.938769	13.830649	13.813984	3.502935
233	Pd	11.924694	1.968192	13.817760	3.496199
234	Pd	11.906641	9.841402	13.856480	3.490215
235	Pd	11.886380	3.968560	15.763200	2.917505
236	Pd	11.904953	11.887800	15.766752	2.830820
237	Pd	11.960483	15.748361	15.766656	2.920142
238	Pd	11.974842	7.812063	15.775584	2.891501

Pd	13.901612	0.006975	13.815520	3.508959
Pd	13.897257	7.872042	13.818016	3.487311
Pd	13.896420	3.929599	13.820384	3.501672
Pd	13.884096	11.824885	13.814368	3.476527
Pd	13.940020	5.865584	15.780320	2.868683
Pd	13.938537	13.773005	15.775840	2.876090
Pd	13.852284	1.998300	15.785568	2.867729
Pd	13.862667	9.881594	15.776960	2.848624
Sr	2.054840	1.961517	5.890464	1.598591
Sr	2.054304	9.851454	5.891008	1.598290
Sr	2.054635	5.906754	5.891072	1.598090
Sr	2.054477	13.796375	5.891264	1.598072
Sr	2.054966	1.962874	9.828000	1.612443
Sr	2.053846	9.852574	9.830976	1.612242
Sr	2.054509	5.907196	9.829760	1.612445
Sr	2.055061	13.797211	9.828832	1.612719
Sr	2.054603	1.962495	1.977504	1.567502
Sr	2.054603	5.907606	1.977504	1.567620
Sr	2.054603	9.852495	1.977504	1.567495
Sr	2.054603	13.797606	1.977504	1.567616
Sr	5.999856	5.907101	5.891488	1.597800
Sr	5.999714	13.796233	5.891488	1.598368
Sr	5.999367	1.961675	5.890752	1.598338
Sr	5.999982	9.850570	5.890976	1.598848
Sr	5.999998	5.908742	9.830656	1.611102
Sr	6.001434	13.796596	9.831808	1.610909
Sr	6.000124	1.962637	9.828160	1.612611
Sr	6.002538	9.852069	9.834624	1.609634
Sr	5.999698	1.962495	1.977504	1.567595
Sr	5.999698	5.907606	1.977504	1.567754
Sr	5.999698	9.852495	1.977504	1.567613
Sr	5.999698	13.797606	1.977504	1.567768
Sr	9.945156	1.962117	5.891104	1.598421
Sr	9.945156	9.851138	5.891680	1.598467
Sr	9.944493	5.906785	5.892224	1.597319
Sr	9.945124	13.795539	5.892192	1.597866
Sr	9.945045	1.963805	9.830432	1.612032
Sr	9.945061	9.852511	9.840096	1.611384
Sr	9.946276	5.910257	9.834784	1.610987
Sr	9.945408	13.794103	9.835872	1.609959
Sr	9.944603	1.962495	1.977504	1.567491
Sr	9.944603	5.907606	1.977504	1.567624
Sr	9.944603	9.852495	1.977504	1.567492
Sr	9.944603	13.797606	1.977504	1.567632
Sr	13.889225	5.907290	5.891168	1.597984
Sr	13.889698	13.796044	5.891552	1.597839
Sr	13.889682	1.961359	5.890432	1.598634
Sr	13.889509	9.851075	5.890912	1.598271
Sr	13.888057	5.908963	9.831200	1.611271
Sr	13.888325	13.797732	9.831008	1.611676
Sr	13.890282	1.962716	9.828928	1.612341
Sr	13.888088	9.851422	9.832160	1.610873
Sr	13.889698	1.962495	1.977504	1.567603
Sr	13.889698	5.907606	1.977504	1.567762
Sr	13.889698	9.852495	1.977504	1.567606
Sr	13.889698	13.797606	1.977504	1.567750
Ti	0.082009	3.933275	7.853440	2.606233
Ti	0.082214	11.822360	7.856032	2.604928
Ti	0.082529	7.878370	7.855072	2.606146
Ti	0.082719	-0.012166	7.853472	2.606611
Ti	0.084439	-0.009089	11.746208	2.814830
Ti	0.081409	7.879743	11.748960	2.814991
Ti	0.082750	3.935406	11.746752	2.815852
Ti	0.081535	11.827031	11.750176	2.815470
Ti	0.081993	3.935106	0.004992	2.388702
Ti	0.081993	3.935106	3.950112	2.605091
Ti	0.081993	7.879901	0.004992	2.388773
Ti	0.081993	7.879901	3.950112	2.604897
Ti	0.081993	11.825106	0.004992	2.388695
Ti	0.081993	11.825106	3.950112	2.604583
Ti	0.081993	-0.010099	0.004992	2.388833
Ti	0.081993	-0.010099	3.950112	2.605250

```
Ti      4.027845       7.878244       7.855296        2.605525
Ti      4.027877      -0.012766       7.854432        2.605827
Ti      4.027813       3.933102       7.853536        2.606854
Ti      4.028681      11.822202       7.854080        2.605682
Ti      4.027119       3.934143      11.746240        2.814996
Ti      4.025604      11.827773      11.747456        2.814628
Ti      4.026693      -0.008395      11.747520        2.815433
Ti      4.025336       7.878986      11.748192        2.814455
Ti      4.027103       3.935106       0.004992        2.388640
Ti      4.027103       3.935106       3.950112        2.605305
Ti      4.027103       7.879901       0.004992        2.388753
Ti      4.027103       7.879901       3.950112        2.605043
Ti      4.027103      11.825106       0.004992        2.388646
Ti      4.027103      11.825106       3.950112        2.604822
Ti      4.027103      -0.010099       0.004992        2.388808
Ti      4.027103      -0.010099       3.950112        2.605180
Ti      7.972845       3.932565       7.855840        2.604885
Ti      7.973208      11.820924       7.855840        2.603901
Ti      7.973713       7.878859       7.856640        2.604697
Ti      7.973650      -0.013034       7.853440        2.605821
Ti      7.970620      -0.006833      11.745568        2.814533
Ti      7.972529       7.879191      11.756640        2.809491
Ti      7.971030       3.936842      11.749376        2.816343
Ti      7.971425      11.826731      11.755488        2.818688
Ti      7.971993       3.935106       0.004992        2.388694
Ti      7.971993       3.935106       3.950112        2.604466
Ti      7.971993       7.879901       0.004992        2.388755
Ti      7.971993       7.879901       3.950112        2.604445
Ti      7.971993      11.825106       0.004992        2.388698
Ti      7.971993      11.825106       3.950112        2.604113
Ti      7.971993      -0.010099       0.004992        2.388842
Ti      7.971993      -0.010099       3.950112        2.604943
Ti     11.916567       7.878512       7.856704        2.605753
Ti     11.917040      -0.012656       7.854912        2.605759
Ti     11.917545       3.933055       7.854720        2.605852
Ti     11.917009      11.821761       7.856288        2.604908
Ti     11.918713       3.936084      11.748288        2.815387
Ti     11.919549      11.823907      11.755488        2.808548
Ti     11.919328      -0.007133      11.747456        2.815377
Ti     11.916804       7.882757      11.756448        2.807917
Ti     11.917103       3.935106       0.004992        2.388578
Ti     11.917103       3.935106       3.950112        2.604934
Ti     11.917103       7.879901       0.004992        2.388660
Ti     11.917103       7.879901       3.950112        2.604674
Ti     11.917103      11.825106       0.004992        2.388554
Ti     11.917103      11.825106       3.950112        2.604350
Ti     11.917103      -0.010099       0.004992        2.388748
Ti     11.917103      -0.010099       3.950112        2.605065
```

1.2.2 CHO

```
      355
jmolscript: load "" {1 1 1} spacegroup "x,y,z" unitcell [{      15.780000
       0.000000      0.000000 }, {      0.000000    15.780000      0.000000
    }, {      0.000000      0.000000    32.000000 }]
C      10.394665       9.371963      17.559232        3.937415
H       9.854720       9.904711      18.371360        0.929525
O      15.709242       1.964436       7.856256        1.904715
O      15.707917       9.853221       7.857824        1.904290
O       0.060216       5.911961      11.687872        2.235572
O       0.063088      13.802734      11.685920        2.236673
O       0.096637       1.967103      11.692896        2.237023
O       0.103327       9.858949      11.696064        2.244901
O       0.078458       3.938183       5.903008        1.939016
O       0.081125      11.825879       5.902848        1.938832
O       0.081377       7.879932       5.903744        1.939204
O       0.080825      -0.010115       5.902304        1.940127
```

O	0.078947	-0.003519	9.812608	1.982015
O	0.071026	7.887728	9.817472	1.980579
O	0.081993	1.962495	0.004992	1.690963
O	0.081993	1.962495	3.950112	1.911609
O	0.081993	3.935106	1.977504	1.897369
O	0.081993	5.907606	0.004992	1.691369
O	0.081993	5.907606	3.950112	1.911681
O	0.081993	7.879901	1.977504	1.897463
O	0.081993	9.852495	0.004992	1.691034
O	0.081993	9.852495	3.950112	1.911302
O	0.081993	11.825106	1.977504	1.897366
O	0.081993	13.797606	0.004992	1.691408
O	0.081993	13.797606	3.950112	1.911915
O	0.081993	-0.010099	1.977504	1.897438
O	0.083950	3.934775	9.814208	1.981444
O	0.080399	11.824001	9.813440	1.982189
O	0.233481	5.909468	7.862176	1.904378
O	0.232708	13.798679	7.860032	1.904419
O	2.051637	15.757955	11.688224	2.238529
O	2.051242	3.958129	11.693024	2.236000
O	2.050863	7.863442	11.687072	2.237779
O	2.052568	11.848713	11.686240	2.241869
O	2.054603	3.935106	0.004992	1.690895
O	2.054603	3.935106	3.950112	1.911205
O	2.054603	7.879901	0.004992	1.690935
O	2.054603	7.879901	3.950112	1.911283
O	2.054603	11.825106	0.004992	1.690964
O	2.054603	11.825106	3.950112	1.911428
O	2.054603	-0.010099	0.004992	1.690950
O	2.054603	-0.010099	3.950112	1.911380
O	2.053451	3.785243	7.855200	1.904896
O	2.053657	11.673665	7.858720	1.904276
O	2.053341	0.142683	7.857408	1.904290
O	2.052883	8.032667	7.863136	1.903185
O	3.875694	5.909799	7.858720	1.905238
O	3.874874	13.798616	7.859072	1.904560
O	4.004869	1.966977	11.688000	2.239150
O	4.003717	9.856551	11.695552	2.238885
O	4.020902	3.938846	9.812544	1.982252
O	4.027387	11.825106	9.813312	1.981635
O	4.045093	5.912261	11.690048	2.239076
O	4.042268	13.802529	11.686848	2.238659
O	4.024452	-0.006265	9.811616	1.982549
O	4.025936	7.884856	9.815040	1.981150
O	4.027103	1.962495	0.004992	1.690955
O	4.027103	1.962495	3.950112	1.911530
O	4.027103	3.935106	1.977504	1.897353
O	4.027103	5.907606	0.004992	1.691394
O	4.027103	5.907606	3.950112	1.911652
O	4.027103	7.879901	1.977504	1.897290
O	4.027103	9.852495	0.004992	1.691033
O	4.027103	9.852495	3.950112	1.911335
O	4.027103	11.825106	1.977504	1.897354
O	4.027103	13.797606	0.004992	1.691424
O	4.027103	13.797606	3.950112	1.911878
O	4.027103	-0.010099	1.977504	1.897324
O	4.027529	7.880595	5.903680	1.938179
O	4.027261	-0.009594	5.902304	1.939963
O	4.028097	3.936921	5.902144	1.939894
O	4.025872	11.826037	5.902112	1.940128
O	4.178260	1.964326	7.857152	1.904740
O	4.178970	9.853616	7.857152	1.905414
O	5.995532	3.920841	11.685824	2.237429
O	5.997110	11.809642	11.692576	2.237510
O	5.995816	0.011393	11.686656	2.238812
O	5.999698	7.906474	11.688704	2.240725
O	5.999698	3.935106	0.004992	1.691111
O	5.999698	3.935106	3.950112	1.911551
O	5.999698	7.879901	0.004992	1.691175
O	5.999698	7.879901	3.950112	1.911274
O	5.999698	11.825106	0.004992	1.691213
O	5.999698	11.825106	3.950112	1.911613

O	5.999698	-0.010099	0.004992	1.691169
O	5.999698	-0.010099	3.950112	1.911475
O	5.999177	4.088346	7.861248	1.903786
O	5.998877	11.977872	7.856832	1.904469
O	6.001308	7.730022	7.864768	1.903871
O	5.999114	15.619265	7.858720	1.904583
O	7.820110	1.963931	7.858944	1.904554
O	7.819684	9.853821	7.860896	1.905147
O	7.950216	5.913145	11.688736	2.236025
O	7.953436	13.801772	11.692352	2.236923
O	7.984270	1.966456	11.686176	2.241731
O	7.996752	9.857498	11.691616	2.236240
O	7.970825	3.936652	5.902528	1.939010
O	7.973208	11.824522	5.902656	1.938786
O	7.972924	7.881447	5.904288	1.937437
O	7.971835	-0.008474	5.902240	1.940158
O	7.972608	-0.009042	9.813280	1.981991
O	7.983528	7.884067	9.819584	1.978983
O	7.971993	1.962495	0.004992	1.691038
O	7.971993	1.962495	3.950112	1.911922
O	7.971993	3.935106	1.977504	1.897347
O	7.971993	5.907606	0.004992	1.691465
O	7.971993	5.907606	3.950112	1.911737
O	7.971993	7.879901	1.977504	1.897452
O	7.971993	9.852495	0.004992	1.691152
O	7.971993	9.852495	3.950112	1.911639
O	7.971993	11.825106	1.977504	1.897358
O	7.971993	13.797606	0.004992	1.691507
O	7.971993	13.797606	3.950112	1.911878
O	7.971993	-0.010099	1.977504	1.897446
O	7.974833	3.938609	9.813632	1.982386
O	7.967890	11.830361	9.814496	1.981744
O	8.125343	5.909736	7.859968	1.904555
O	8.124286	13.798679	7.856960	1.905130
O	9.941794	15.759817	11.695008	2.238597
O	9.939427	3.950602	11.696736	2.244623
O	9.947239	7.872342	11.716960	2.239344
O	9.941305	11.838298	11.691104	2.235206
O	9.944603	3.935106	0.004992	1.690963
O	9.944603	3.935106	3.950112	1.910987
O	9.944603	7.879901	0.004992	1.691039
O	9.944603	7.879901	3.950112	1.911234
O	9.944603	11.825106	0.004992	1.691082
O	9.944603	11.825106	3.950112	1.911320
O	9.944603	-0.010099	0.004992	1.691032
O	9.944603	-0.010099	3.950112	1.911321
O	9.944351	3.783081	7.855872	1.904934
O	9.944351	11.672782	7.861280	1.904497
O	9.944382	0.142762	7.857824	1.904558
O	9.946323	8.034387	7.861504	1.904769
O	11.241135	8.518738	17.833984	2.401433
O	11.763012	5.909689	7.861632	1.905324
O	11.764653	13.796549	7.864832	1.903401
O	11.899177	1.967514	11.687456	2.237864
O	11.889709	9.851722	11.716832	2.238453
O	11.908267	3.946357	9.817792	1.980103
O	11.914484	11.814912	9.819328	1.978599
O	11.927471	5.912371	11.715648	2.223397
O	11.927834	13.799247	11.688864	2.239874
O	11.914058	-0.007969	9.814976	1.981084
O	11.917498	7.881526	9.826048	1.974388
O	11.917103	1.962495	0.004992	1.690933
O	11.917103	1.962495	3.950112	1.911578
O	11.917103	3.935106	1.977504	1.897373
O	11.917103	5.907606	0.004992	1.691378
O	11.917103	5.907606	3.950112	1.911713
O	11.917103	7.879901	1.977504	1.897295
O	11.917103	9.852495	0.004992	1.691040
O	11.917103	9.852495	3.950112	1.911370
O	11.917103	11.825106	1.977504	1.897361
O	11.917103	13.797606	0.004992	1.691427
O	11.917103	13.797606	3.950112	1.911865

159	O	11.917103	-0.010099	1.977504	1.897279
160	O	11.917072	7.880327	5.904352	1.937645
161	O	11.916598	-0.010036	5.903680	1.938141
162	O	11.918823	3.936573	5.903680	1.939052
163	O	11.915873	11.824648	5.904224	1.937714
164	O	12.068733	1.964720	7.862944	1.903752
165	O	12.071021	9.851691	7.860928	1.905748
166	O	13.886116	3.921914	11.687328	2.234436
167	O	13.885674	11.813192	11.688448	2.235786
168	O	13.886416	0.008884	11.690368	2.238269
169	O	13.886937	7.907689	11.715808	2.222611
170	O	13.889698	3.935106	0.004992	1.691072
171	O	13.889698	3.935106	3.950112	1.911461
172	O	13.889698	7.879901	0.004992	1.691126
173	O	13.889698	7.879901	3.950112	1.911220
174	O	13.889698	11.825106	0.004992	1.691174
175	O	13.889698	11.825106	3.950112	1.911639
176	O	13.889698	-0.010099	0.004992	1.691137
177	O	13.889698	-0.010099	3.950112	1.911319
178	O	13.887883	4.089182	7.864736	1.903115
179	O	13.888088	11.978077	7.860416	1.904218
180	O	13.887931	7.726787	7.860704	1.905372
181	O	13.888104	15.618934	7.858336	1.904833
182	Pd	0.065566	0.002904	15.761088	2.915463
183	Pd	0.068974	7.896344	15.774976	2.897493
184	Pd	0.079894	1.973336	13.822560	3.508714
185	Pd	0.071436	9.846972	13.811872	3.494091
186	Pd	0.093165	3.925638	15.765216	2.933111
187	Pd	0.071262	5.911314	13.819424	3.492997
188	Pd	0.114468	11.841754	15.759520	2.938508
189	Pd	0.086743	13.812834	13.813216	3.506886
190	Pd	2.046256	0.003251	13.816704	3.505877
191	Pd	2.054903	7.878765	13.816000	3.490996
192	Pd	2.045419	3.941986	13.822816	3.507162
193	Pd	2.052410	11.839166	13.812320	3.494295
194	Pd	2.034863	5.924680	15.774784	2.873888
195	Pd	2.041064	13.822901	15.781088	2.881986
196	Pd	2.056008	1.963569	15.779456	2.868236
197	Pd	2.056118	9.840203	15.770176	2.867232
198	Pd	4.017856	5.905334	13.815584	3.503818
199	Pd	4.015268	13.812849	13.814176	3.506353
200	Pd	4.012018	1.972090	13.816032	3.506549
201	Pd	4.020129	9.862816	13.818080	3.503376
202	Pd	4.015552	3.954752	15.761344	2.915296
203	Pd	4.001603	11.849139	15.758080	2.918972
204	Pd	4.023048	-0.006422	15.756704	2.936644
205	Pd	4.029439	7.869470	15.762144	2.921326
206	Pd	5.981867	0.002872	13.813472	3.506975
207	Pd	5.976975	7.883388	13.814240	3.489765
208	Pd	5.985054	3.929378	13.813312	3.506496
209	Pd	5.977290	11.848208	13.819232	3.501303
210	Pd	6.006783	5.901436	15.773344	2.870028
211	Pd	5.990498	13.805228	15.784864	2.866277
212	Pd	5.970884	1.976382	15.781568	2.882304
213	Pd	5.965582	9.869664	15.775072	2.871404
214	Pd	7.944141	0.016285	15.758240	2.920198
215	Pd	7.946193	7.897480	15.762784	2.914566
216	Pd	7.957349	1.964058	13.812768	3.495351
217	Pd	7.934642	9.869380	13.817568	3.450027
218	Pd	7.954935	3.902189	15.759680	2.938423
219	Pd	7.952031	5.892931	13.816256	3.502089
220	Pd	7.955203	11.845257	15.760256	2.943876
221	Pd	7.946208	13.821860	13.819136	3.501691
222	Pd	9.933715	-0.003819	13.818048	3.502665
223	Pd	9.913990	7.858188	13.836448	3.423731
224	Pd	9.948406	3.943864	13.812032	3.496801
225	Pd	9.931222	11.871783	13.818496	3.451790
226	Pd	9.878106	5.883005	15.767296	2.822425
227	Pd	9.927861	13.836661	15.776256	2.871759
228	Pd	9.953519	1.958046	15.771328	2.866965
229	Pd	9.909493	9.890147	15.792384	3.652632
230	Pd	11.915462	5.902982	13.854048	3.498878

231	Pd	11.918965	13.824463	13.815104	3.491246
232	Pd	11.916662	1.961044	13.816960	3.491742
233	Pd	11.936071	9.886217	13.834912	3.426038
234	Pd	11.900203	3.944795	15.775520	2.898105
235	Pd	11.899319	11.855845	15.763104	2.915352
236	Pd	11.929364	-0.010036	15.763136	2.921375
237	Pd	11.965106	7.838636	15.838880	2.940496
238	Pd	13.894243	0.004766	13.815968	3.504357
239	Pd	13.885721	7.883104	13.855712	3.500196
240	Pd	13.888341	3.940108	13.819680	3.493570
241	Pd	13.904910	11.846677	13.816160	3.501514
242	Pd	13.918070	5.876235	15.777088	2.906831
243	Pd	13.898566	13.792288	15.774240	2.869289
244	Pd	13.874660	1.982394	15.777088	2.873331
245	Pd	13.910985	9.920697	15.766176	2.829472
246	Sr	2.053246	1.964547	5.891040	1.597766
247	Sr	2.053483	9.855068	5.891776	1.597176
248	Sr	2.053341	5.909973	5.891872	1.597061
249	Sr	2.053341	13.799279	5.891232	1.597448
250	Sr	2.050785	1.968145	9.828032	1.612618
251	Sr	2.051558	9.857545	9.831712	1.611779
252	Sr	2.051794	5.912419	9.829376	1.612320
253	Sr	2.052142	13.802829	9.829024	1.612671
254	Sr	2.054603	1.962495	1.977504	1.567400
255	Sr	2.054603	5.907606	1.977504	1.567543
256	Sr	2.054603	9.852495	1.977504	1.567409
257	Sr	2.054603	13.797606	1.977504	1.567539
258	Sr	5.998767	5.910068	5.892160	1.596903
259	Sr	5.999051	13.799405	5.891008	1.597922
260	Sr	5.999161	1.965320	5.890784	1.597617
261	Sr	5.999225	9.855588	5.891904	1.597423
262	Sr	5.997457	5.912324	9.831520	1.611296
263	Sr	5.996668	13.801582	9.829760	1.611434
264	Sr	5.996558	1.967782	9.828608	1.612422
265	Sr	5.998183	9.857734	9.831520	1.610860
266	Sr	5.999698	1.962495	1.977504	1.567510
267	Sr	5.999698	5.907606	1.977504	1.567650
268	Sr	5.999698	9.852495	1.977504	1.567468
269	Sr	5.999698	13.797606	1.977504	1.567643
270	Sr	9.942694	1.964326	5.891232	1.597980
271	Sr	9.943025	9.855352	5.892480	1.597379
272	Sr	9.943010	5.910620	5.892480	1.597595
273	Sr	9.943846	13.799736	5.892064	1.597220
274	Sr	9.940942	1.967229	9.830496	1.611309
275	Sr	9.943341	9.855951	9.839744	1.611188
276	Sr	9.942647	5.915101	9.837280	1.608967
277	Sr	9.941084	13.801062	9.832512	1.611116
278	Sr	9.944603	1.962495	1.977504	1.567405
279	Sr	9.944603	5.907606	1.977504	1.567519
280	Sr	9.944603	9.852495	1.977504	1.567396
281	Sr	9.944603	13.797606	1.977504	1.567512
282	Sr	13.887868	5.909910	5.891936	1.597545
283	Sr	13.888688	13.799263	5.891840	1.597367
284	Sr	13.888167	1.964815	5.891200	1.597386
285	Sr	13.887820	9.855730	5.892032	1.597825
286	Sr	13.883197	5.914376	9.834784	1.609402
287	Sr	13.886116	13.801330	9.830720	1.611020
288	Sr	13.887568	1.967940	9.829440	1.612592
289	Sr	13.885264	9.856030	9.836640	1.609044
290	Sr	13.889698	1.962495	1.977504	1.567530
291	Sr	13.889698	5.907606	1.977504	1.567697
292	Sr	13.889698	9.852495	1.977504	1.567489
293	Sr	13.889698	13.797606	1.977504	1.567643
294	Ti	0.079200	3.941039	7.855008	2.606015
295	Ti	0.079421	11.829509	7.855744	2.606026
296	Ti	0.076817	7.885487	7.854976	2.607092
297	Ti	0.079216	-0.005097	7.854048	2.607133
298	Ti	0.079231	-0.003156	11.747360	2.814577
299	Ti	0.079452	7.882205	11.749696	2.812744
300	Ti	0.078285	3.940029	11.748768	2.813810
301	Ti	0.079705	11.831513	11.748928	2.816420
302	Ti	0.081993	3.935106	0.004992	2.388865

```
Ti      0.081993      3.935106      3.950112      2.604397
Ti      0.081993      7.879901      0.004992      2.388991
Ti      0.081993      7.879901      3.950112      2.604376
Ti      0.081993     11.825106      0.004992      2.388973
Ti      0.081993     11.825106      3.950112      2.604747
Ti      0.081993     -0.010099      0.004992      2.389017
Ti      0.081993     -0.010099      3.950112      2.605088
Ti      4.024168      7.885787      7.856096      2.606082
Ti      4.025241     -0.004829      7.853760      2.607086
Ti      4.024610      3.940408      7.854080      2.607369
Ti      4.025052     11.829556      7.854272      2.606450
Ti      4.021139      3.939161     11.747360      2.814425
Ti      4.022969     11.831023     11.748224      2.817030
Ti      4.021912     -0.003835     11.746400      2.815780
Ti      4.022417      7.884240     11.750400      2.813703
Ti      4.027103      3.935106      0.004992      2.388825
Ti      4.027103      3.935106      3.950112      2.605063
Ti      4.027103      7.879901      0.004992      2.388983
Ti      4.027103      7.879901      3.950112      2.604564
Ti      4.027103     11.825106      0.004992      2.388926
Ti      4.027103     11.825106      3.950112      2.605074
Ti      4.027103     -0.010099      0.004992      2.388995
Ti      4.027103     -0.010099      3.950112      2.605145
Ti      7.970809      3.940866      7.855552      2.606141
Ti      7.970652     11.829824      7.856096      2.606414
Ti      7.970020      7.885440      7.857280      2.605089
Ti      7.969989     -0.005397      7.854304      2.606569
Ti      7.966738     -0.004103     11.747872      2.816739
Ti      7.967306      7.885471     11.754784      2.810128
Ti      7.966344      3.938672     11.748864      2.816261
Ti      7.968537     11.830187     11.750400      2.813804
Ti      7.971993      3.935106      0.004992      2.388972
Ti      7.971993      3.935106      3.950112      2.604764
Ti      7.971993      7.879901      0.004992      2.389128
Ti      7.971993      7.879901      3.950112      2.604113
Ti      7.971993     11.825106      0.004992      2.389118
Ti      7.971993     11.825106      3.950112      2.605042
Ti      7.971993     -0.010099      0.004992      2.389123
Ti      7.971993     -0.010099      3.950112      2.605179
Ti     11.913521      7.886686      7.856864      2.604397
Ti     11.914768     -0.004024      7.856192      2.605544
Ti     11.913206      3.942033      7.855200      2.606943
Ti     11.914058     11.829319      7.857376      2.605122
Ti     11.915857      3.937725     11.750208      2.812184
Ti     11.913537     11.831355     11.754688      2.809253
Ti     11.914074     -0.003898     11.750240      2.814065
Ti     11.914973      7.883451     11.761952      2.813226
Ti     11.917103      3.935106      0.004992      2.388790
Ti     11.917103      3.935106      3.950112      2.604328
Ti     11.917103      7.879901      0.004992      2.388980
Ti     11.917103      7.879901      3.950112      2.604134
Ti     11.917103     11.825106      0.004992      2.388933
Ti     11.917103     11.825106      3.950112      2.604266
Ti     11.917103     -0.010099      0.004992      2.388980
Ti     11.917103     -0.010099      3.950112      2.604433
```

1.2.3 HCOO

```
      356
jmolscript: load "" {1 1 1} spacegroup "x,y,z" unitcell [{     15.780000      0.000000      0.000000 }, {      0.000000     15.780000      0.000000 }, {      0.000000      0.000000     32.000000 }]
C      10.089337      7.720523     18.144256      4.138707
H       9.789265      8.025313     19.165280      0.922450
O       0.066513     13.795144     11.683104      2.238724
O       0.065913      5.903393     11.691200      2.238067
O       0.081141     11.824348      5.901440      1.940494
```

O	0.081993	1.962495	0.004992	1.690872
O	0.081993	1.962495	3.950112	1.911735
O	0.081993	3.935106	1.977504	1.897476
O	0.081993	5.907606	0.004992	1.691319
O	0.081993	5.907606	3.950112	1.911509
O	0.081993	7.879901	1.977504	1.897512
O	0.081993	9.852495	0.004992	1.690944
O	0.081993	9.852495	3.950112	1.911659
O	0.081993	11.825106	1.977504	1.897435
O	0.081993	13.797606	0.004992	1.691281
O	0.081993	13.797606	3.950112	1.911865
O	0.081993	-0.010099	1.977504	1.897596
O	0.073487	7.879948	5.903008	1.939735
O	0.084754	-0.010178	9.811424	1.982878
O	0.080762	3.935343	5.901984	1.939847
O	0.081693	-0.009137	5.901760	1.941144
O	0.092550	7.876177	9.812064	1.985892
O	0.089299	11.823907	9.808736	1.983947
O	0.080383	3.935863	9.812256	1.982423
O	0.100093	1.960192	11.687712	2.235067
O	0.107777	9.851328	11.683840	2.242162
O	0.234870	5.907890	7.855872	1.904104
O	0.235138	13.797685	7.856128	1.904728
O	2.054603	3.935106	0.004992	1.690803
O	2.054603	3.935106	3.950112	1.911771
O	2.054603	7.879901	0.004992	1.690843
O	2.054603	7.879901	3.950112	1.910843
O	2.054603	11.825106	0.004992	1.690833
O	2.054603	11.825106	3.950112	1.911967
O	2.054603	-0.010099	0.004992	1.690815
O	2.054603	-0.010099	3.950112	1.911204
O	2.056939	11.672198	7.853152	1.905198
O	2.055550	8.032336	7.848352	1.905650
O	2.055692	0.141862	7.855744	1.905001
O	2.054998	3.784502	7.856832	1.905110
O	2.056781	11.841912	11.690080	2.234646
O	2.054540	15.747462	11.690336	2.236027
O	2.054698	7.857051	11.689120	2.246229
O	2.053010	3.948093	11.688608	2.234666
O	3.878377	5.907543	7.854400	1.904727
O	3.877840	13.797243	7.855008	1.904776
O	4.009098	9.849087	11.683520	2.239031
O	4.005895	1.957888	11.684096	2.238233
O	4.026330	11.825280	9.810432	1.983437
O	4.025794	3.934995	9.810176	1.984038
O	4.027103	1.962495	0.004992	1.690834
O	4.027103	1.962495	3.950112	1.911548
O	4.027103	3.935106	1.977504	1.897466
O	4.027103	5.907606	0.004992	1.691318
O	4.027103	5.907606	3.950112	1.911547
O	4.027103	7.879901	1.977504	1.897363
O	4.027103	9.852495	0.004992	1.690920
O	4.027103	9.852495	3.950112	1.911700
O	4.027103	11.825106	1.977504	1.897456
O	4.027103	13.797606	0.004992	1.691277
O	4.027103	13.797606	3.950112	1.911980
O	4.027103	-0.010099	1.977504	1.897457
O	4.028839	-0.009263	5.901696	1.941040
O	4.037534	7.880232	5.903008	1.938670
O	4.030685	11.824001	5.901408	1.940386
O	4.028003	3.934317	5.901472	1.940573
O	4.025162	-0.013807	9.811712	1.982598
O	4.014606	7.876066	9.810720	1.984844
O	4.048896	13.794229	11.688384	2.236722
O	4.042331	5.902383	11.689216	2.239752
O	4.180674	1.962227	7.857920	1.904112
O	4.183120	9.851596	7.858976	1.904273
O	5.998972	7.891089	11.673568	2.232721
O	5.999698	3.935106	0.004992	1.691009
O	5.999698	3.935106	3.950112	1.910682
O	5.999698	7.879901	0.004992	1.691054
O	5.999698	7.879901	3.950112	1.911670

O	5.999698	11.825106	0.004992	1.691056
O	5.999698	11.825106	3.950112	1.911458
O	5.999698	-0.010099	0.004992	1.691010
O	5.999698	-0.010099	3.950112	1.911414
O	6.006199	7.728239	7.879168	1.901321
O	6.002791	11.976752	7.860544	1.904660
O	6.001686	4.087225	7.858144	1.904221
O	6.001386	15.618413	7.859456	1.904370
O	6.001560	11.798422	11.682816	2.237919
O	5.999698	0.006754	11.688128	2.233317
O	5.999808	3.911531	11.681088	2.239564
O	7.821105	9.850334	7.863744	1.905172
O	7.822919	1.963000	7.863104	1.903848
O	7.952599	13.792872	11.690784	2.239317
O	7.960568	5.903945	11.708384	2.244132
O	8.010338	7.876966	9.829184	1.970419
O	7.974202	3.935990	5.903808	1.938146
O	7.973634	11.822471	5.903520	1.937780
O	7.971993	1.962495	0.004992	1.690877
O	7.971993	1.962495	3.950112	1.911900
O	7.971993	3.935106	1.977504	1.897488
O	7.971993	5.907606	0.004992	1.691359
O	7.971993	5.907606	3.950112	1.911515
O	7.971993	7.879901	1.977504	1.897569
O	7.971993	9.852495	0.004992	1.691004
O	7.971993	9.852495	3.950112	1.912268
O	7.971993	11.825106	1.977504	1.897467
O	7.971993	13.797606	0.004992	1.691302
O	7.971993	13.797606	3.950112	1.911786
O	7.971993	-0.010099	1.977504	1.897513
O	7.968490	7.879648	5.905696	1.936381
O	7.972151	-0.009784	5.902912	1.938609
O	7.975843	-0.016206	9.813280	1.981413
O	7.971314	3.941386	9.816800	1.981965
O	7.978431	11.817421	9.817696	1.978951
O	7.994921	1.957272	11.683328	2.243378
O	8.001044	9.848124	11.708096	2.235179
O	8.127031	13.795334	7.859584	1.904184
O	8.128373	5.908805	7.861888	1.904595
O	9.674166	6.587535	17.768480	2.455457
O	9.947586	8.036470	7.849952	1.908368
O	9.946860	7.855978	11.762656	2.238319
O	9.944603	3.935106	0.004992	1.690879
O	9.944603	3.935106	3.950112	1.911286
O	9.944603	7.879901	0.004992	1.690972
O	9.944603	7.879901	3.950112	1.910842
O	9.944603	11.825106	0.004992	1.690933
O	9.944603	11.825106	3.950112	1.911536
O	9.944603	-0.010099	0.004992	1.690879
O	9.944603	-0.010099	3.950112	1.910766
O	9.946655	11.669847	7.859008	1.904721
O	9.945897	0.142241	7.855424	1.906074
O	9.945787	3.783192	7.862400	1.904681
O	9.947980	11.846456	11.701856	2.219014
O	9.944493	15.745095	11.692576	2.242699
O	9.942252	3.953395	11.699520	2.198963
O	10.824985	8.558962	17.516224	2.517713
O	11.763848	5.909294	7.861216	1.904692
O	11.766578	13.795665	7.862784	1.902738
O	11.898562	9.847541	11.708576	2.242897
O	11.893323	1.960207	11.687776	2.239100
O	11.888573	7.872216	9.833056	1.969004
O	11.913285	11.814092	9.817792	1.980226
O	11.923384	7.880090	5.905728	1.937166
O	11.918161	-0.009105	5.903360	1.937934
O	11.917103	1.962495	0.004992	1.690807
O	11.917103	1.962495	3.950112	1.911586
O	11.917103	3.935106	1.977504	1.897487
O	11.917103	5.907606	0.004992	1.691313
O	11.917103	5.907606	3.950112	1.911699
O	11.917103	7.879901	1.977504	1.897468
O	11.917103	9.852495	0.004992	1.690921

152	O	11.917103	9.852495	3.950112	1.911698
153	O	11.917103	11.825106	1.977504	1.897451
154	O	11.917103	13.797606	0.004992	1.691252
155	O	11.917103	13.797606	3.950112	1.911895
156	O	11.917103	-0.010099	1.977504	1.897354
157	O	11.917877	11.822376	5.904160	1.937338
158	O	11.916362	3.936368	5.904384	1.938015
159	O	11.916851	3.948456	9.821472	1.978062
160	O	11.914105	-0.009231	9.812832	1.982277
161	O	11.936024	13.795555	11.682720	2.245445
162	O	11.926871	5.907653	11.728384	2.233231
163	O	12.069712	1.965257	7.864608	1.903546
164	O	12.073530	9.849513	7.865856	1.904307
165	O	13.886747	7.726961	7.877888	1.901707
166	O	13.889698	3.935106	0.004992	1.691004
167	O	13.889698	3.935106	3.950112	1.910798
168	O	13.889698	7.879901	0.004992	1.691032
169	O	13.889698	7.879901	3.950112	1.911487
170	O	13.889698	11.825106	0.004992	1.691034
171	O	13.889698	11.825106	3.950112	1.911396
172	O	13.889698	-0.010099	0.004992	1.691002
173	O	13.889698	-0.010099	3.950112	1.911271
174	O	13.895268	7.896217	11.695872	2.208347
175	O	13.890219	11.977225	7.860448	1.904828
176	O	13.890156	15.618918	7.858880	1.904660
177	O	13.888688	4.088850	7.860192	1.904759
178	O	13.890597	11.805302	11.673856	2.242987
179	O	13.889603	0.005144	11.687136	2.232497
180	O	13.885027	3.914481	11.686976	2.238376
181	O	15.709953	9.851233	7.858560	1.902769
182	O	15.710915	1.963095	7.857920	1.903954
183	Pd	0.098830	7.860050	15.760032	2.916290
184	Pd	0.102933	15.737520	15.759392	2.916802
185	Pd	0.086111	13.803681	13.811392	3.508530
186	Pd	0.066087	5.899558	13.816960	3.485614
187	Pd	0.059585	9.842238	13.803488	3.486453
188	Pd	0.088116	1.937689	13.819456	3.508518
189	Pd	0.067933	3.927484	15.761152	2.937101
190	Pd	0.066024	11.855151	15.753696	2.951251
191	Pd	2.037940	1.975956	15.774976	2.879570
192	Pd	2.056955	11.823039	13.820192	3.498636
193	Pd	2.056986	15.755210	13.819040	3.516244
194	Pd	2.018404	9.864693	15.763104	2.872780
195	Pd	2.047423	3.924028	13.821440	3.490362
196	Pd	2.046287	7.873447	13.809728	3.499454
197	Pd	2.078431	13.758787	15.772960	2.881557
198	Pd	2.070588	5.885198	15.770304	2.858476
199	Pd	4.049274	11.794651	15.758176	2.920145
200	Pd	4.037045	3.900784	15.757664	2.930474
201	Pd	4.026109	13.787286	13.815872	3.508559
202	Pd	4.013611	1.942739	13.811936	3.508734
203	Pd	4.013280	9.850413	13.810496	3.501029
204	Pd	4.017635	5.901010	13.811520	3.497988
205	Pd	3.999709	-0.004986	15.761280	2.923614
206	Pd	3.993303	7.897843	15.757920	2.924325
207	Pd	5.990719	11.815543	13.810592	3.495687
208	Pd	5.988983	9.828163	15.786208	2.910887
209	Pd	5.982829	13.817725	15.770176	2.879532
210	Pd	5.994238	15.749986	13.820384	3.496482
211	Pd	5.938298	5.900142	15.779072	2.859116
212	Pd	5.961526	3.905818	13.802528	3.515134
213	Pd	6.001655	1.910863	15.770560	2.898809
214	Pd	5.986758	7.882299	13.804224	3.503402
215	Pd	7.908399	7.852964	15.741664	2.810982
216	Pd	8.004626	15.749481	15.759776	2.898532
217	Pd	7.959432	9.831871	13.832320	3.504003
218	Pd	7.963787	1.959844	13.808416	3.473196
219	Pd	7.965918	13.777692	13.818080	3.484891
220	Pd	7.923154	5.879123	13.822560	3.486369
221	Pd	7.910419	11.837067	15.774688	2.953273
222	Pd	7.873289	3.907649	15.773280	2.898942
223	Pd	9.922622	1.992793	15.763648	2.862813

Pd	9.943593	3.968354	13.872064	3.488625
Pd	9.930748	9.949353	15.835456	2.829490
Pd	9.927387	11.764779	13.848768	3.502165
Pd	9.937897	-0.013082	13.813664	3.495869
Pd	9.941021	7.834660	13.880160	3.379637
Pd	9.955602	13.747662	15.758720	2.867874
Pd	9.925683	5.731280	15.852224	3.043799
Pd	11.977146	11.823275	15.760608	2.899143
Pd	11.982385	3.880917	15.782016	2.925638
Pd	11.941641	5.874121	13.854176	3.494573
Pd	11.918997	9.853711	13.821536	3.470166
Pd	11.918902	1.958219	13.816640	3.478623
Pd	11.909908	13.788075	13.805536	3.474083
Pd	12.015902	7.898600	15.789824	2.993205
Pd	11.877448	-0.005113	15.761056	2.896808
Pd	13.912910	5.882232	15.799616	2.895716
Pd	13.868016	13.821923	15.766944	2.889093
Pd	13.914252	3.904509	13.812032	3.506439
Pd	13.886589	-0.009673	13.818624	3.497528
Pd	13.907182	11.839860	13.792864	3.506564
Pd	13.909202	1.916118	15.782272	2.879978
Pd	13.863235	7.866156	13.855584	3.512552
Pd	13.948179	9.887985	15.778016	2.856244
Sr	2.054603	1.962495	1.977504	1.567511
Sr	2.054603	5.907606	1.977504	1.567567
Sr	2.054603	9.852495	1.977504	1.567479
Sr	2.054603	13.797606	1.977504	1.567596
Sr	2.052583	1.960413	5.890304	1.598579
Sr	2.052946	13.793550	5.890752	1.598656
Sr	2.053341	5.903377	5.891680	1.597871
Sr	2.052757	9.850870	5.890048	1.599291
Sr	2.053814	13.792257	9.826624	1.612561
Sr	2.051542	1.957651	9.826816	1.612551
Sr	2.052394	5.900915	9.828448	1.612556
Sr	2.053183	9.848109	9.826880	1.613526
Sr	5.999698	1.962495	1.977504	1.567583
Sr	5.999698	5.907606	1.977504	1.567717
Sr	5.999698	9.852495	1.977504	1.567607
Sr	5.999698	13.797606	1.977504	1.567709
Sr	5.998167	13.794055	5.891584	1.597975
Sr	5.998972	1.959371	5.891360	1.597401
Sr	5.998341	1.956909	9.829632	1.612210
Sr	5.999067	9.849324	5.891264	1.597643
Sr	5.999509	13.791468	9.829408	1.612175
Sr	5.998057	5.906328	5.893312	1.596976
Sr	6.000171	9.845600	9.831456	1.609935
Sr	6.000803	5.900047	9.836608	1.610027
Sr	9.944603	1.962495	1.977504	1.567501
Sr	9.944603	5.907606	1.977504	1.567586
Sr	9.944603	9.852495	1.977504	1.567472
Sr	9.944603	13.797606	1.977504	1.567572
Sr	9.943088	13.793819	5.892480	1.597090
Sr	9.942678	1.959923	5.891680	1.597629
Sr	9.942946	5.904276	5.893376	1.597525
Sr	9.941937	9.850428	5.891648	1.598831
Sr	9.944004	9.845647	9.838880	1.608312
Sr	9.943657	5.901925	9.842240	1.609974
Sr	9.942000	13.789590	9.832928	1.612873
Sr	9.942441	1.957556	9.829856	1.611889
Sr	13.889698	1.962495	1.977504	1.567590
Sr	13.889698	5.907606	1.977504	1.567752
Sr	13.889698	9.852495	1.977504	1.567627
Sr	13.889698	13.797606	1.977504	1.567719
Sr	13.887331	9.849955	5.891552	1.597797
Sr	13.887473	13.794166	5.891680	1.597597
Sr	13.887710	1.959418	5.891680	1.597297
Sr	13.887536	5.905539	5.893184	1.597317
Sr	13.885721	9.844953	9.836224	1.609511
Sr	13.886621	13.792777	9.829088	1.612444
Sr	13.886873	1.958961	9.830848	1.611683
Sr	13.883496	5.901294	9.839040	1.609649
Ti	0.081993	3.935106	0.004992	2.388795

```
Ti       0.081993      3.935106      3.950112       2.605165
Ti       0.081993      7.879901      0.004992       2.388902
Ti       0.081993      7.879901      3.950112       2.604195
Ti       0.081993     11.825106      0.004992       2.388835
Ti       0.081993     11.825106      3.950112       2.605430
Ti       0.081993     -0.010099      0.004992       2.388869
Ti       0.081993     -0.010099      3.950112       2.605344
Ti       0.084549     11.823591     11.743584       2.817484
Ti       0.076281     11.817705      7.854240       2.607065
Ti       0.077133      3.929015      7.854528       2.606335
Ti       0.078032     -0.017405      7.852544       2.606425
Ti       0.081314     -0.013665     11.744096       2.815577
Ti       0.077827      7.872390      7.853728       2.605389
Ti       0.080431      3.929220     11.747456       2.815022
Ti       0.080415      7.875041     11.742176       2.814765
Ti       4.027103      3.935106      0.004992       2.388714
Ti       4.027103      3.935106      3.950112       2.605454
Ti       4.027103      7.879901      0.004992       2.388869
Ti       4.027103      7.879901      3.950112       2.604906
Ti       4.027103     11.825106      0.004992       2.388760
Ti       4.027103     11.825106      3.950112       2.605641
Ti       4.027103     -0.010099      0.004992       2.388814
Ti       4.027103     -0.010099      3.950112       2.605410
Ti       4.027703     11.820356     11.744640       2.815543
Ti       4.026409     -0.015322     11.744416       2.815924
Ti       4.022259      3.928968     11.744032       2.816714
Ti       4.022716     -0.016885      7.852352       2.606619
Ti       4.023427     11.817042      7.853504       2.606469
Ti       4.024500      3.927989      7.853312       2.606519
Ti       4.022006      7.873131      7.855584       2.606804
Ti       4.023427      7.876192     11.745408       2.815564
Ti       7.971993      3.935106      0.004992       2.388810
Ti       7.971993      3.935106      3.950112       2.604439
Ti       7.971993      7.879901      0.004992       2.388970
Ti       7.971993      7.879901      3.950112       2.603514
Ti       7.971993     11.825106      0.004992       2.388883
Ti       7.971993     11.825106      3.950112       2.604777
Ti       7.971993     -0.010099      0.004992       2.388887
Ti       7.971993     -0.010099      3.950112       2.604870
Ti       7.972987     11.819457     11.753760       2.809691
Ti       7.975070      3.924091     11.751392       2.810571
Ti       7.970431     -0.017532     11.749184       2.814672
Ti       7.973382      7.873999     11.764416       2.806066
Ti       7.967922     11.816632      7.856736       2.605048
Ti       7.969137     15.761837      7.855520       2.605468
Ti       7.968632      3.929078      7.856320       2.605728
Ti       7.971283      7.872121      7.858816       2.604325
Ti      11.917103      3.935106      0.004992       2.388682
Ti      11.917103      3.935106      3.950112       2.604039
Ti      11.917103      7.879901      0.004992       2.388861
Ti      11.917103      7.879901      3.950112       2.603883
Ti      11.917103     11.825106      0.004992       2.388741
Ti      11.917103     11.825106      3.950112       2.604364
Ti      11.917103     -0.010099      0.004992       2.388791
Ti      11.917103     -0.010099      3.950112       2.604700
Ti      11.911612     -0.017279      7.856352       2.605187
Ti      11.912480     11.815401      7.857184       2.604627
Ti      11.910144      7.872626      7.857280       2.604501
Ti      11.913600      3.928794      7.856576       2.605464
Ti      11.916015     -0.013886     11.749056       2.813837
Ti      11.916267      7.872263     11.766176       2.806472
Ti      11.913427     11.824427     11.753152       2.812147
Ti      11.909829      3.926679     11.756128       2.806125
```

1.2.4 CH_3O

```
        357
```

```
jmolscript: load "" {1 1 1} spacegroup "x,y,z" unitcell [{      15.780000
        0.000000      0.000000 }, {       0.000000    15.780000      0.000000
    }, {       0.000000      0.000000    32.000000 }]
C        8.498177      9.445608     18.359040        3.911533
H        7.709571     10.139534     18.010976        1.063539
H        8.048573      8.685691     19.017824        0.960760
H        9.257526     10.012915     18.919552        0.965503
O       15.715870      1.964026      7.858336        1.905001
O       15.714182      9.854121      7.859040        1.905392
O        0.063483      5.907701     11.689568        2.237046
O        0.065392     13.798663     11.691776        2.235079
O        0.101749      1.963016     11.689696        2.238154
O        0.103469      9.853742     11.692736        2.238067
O        0.081930      3.936495      5.902624        1.939366
O        0.080951     11.825863      5.902432        1.939714
O        0.080604      7.881431      5.903552        1.938062
O        0.082324     -0.009200      5.902464        1.939510
O        0.083808     -0.009294      9.813728        1.981707
O        0.085023      7.882710      9.814208        1.981225
O        0.081993      1.962495      0.004992        1.691048
O        0.081993      1.962495      3.950112        1.911598
O        0.081993      3.935106      1.977504        1.897370
O        0.081993      5.907606      0.004992        1.691486
O        0.081993      5.907606      3.950112        1.911412
O        0.081993      7.879901      1.977504        1.897411
O        0.081993      9.852495      0.004992        1.691137
O        0.081993      9.852495      3.950112        1.911675
O        0.081993     11.825106      1.977504        1.897380
O        0.081993     13.797606      0.004992        1.691481
O        0.081993     13.797606      3.950112        1.911613
O        0.081993     -0.010099      1.977504        1.897471
O        0.088210      3.936147      9.813184        1.982064
O        0.082151     11.826637      9.813824        1.981843
O        0.232187      5.909768      7.858688        1.905425
O        0.232376     13.798853      7.858336        1.905227
O        2.055661     15.756409     11.691136        2.237673
O        2.057538      3.953931     11.693184        2.233779
O        2.056639      7.858961     11.687264        2.241140
O        2.054824     11.844831     11.687072        2.240425
O        2.054603      3.935106      0.004992        1.690999
O        2.054603      3.935106      3.950112        1.911477
O        2.054603      7.879901      0.004992        1.691063
O        2.054603      7.879901      3.950112        1.911009
O        2.054603     11.825106      0.004992        1.691058
O        2.054603     11.825106      3.950112        1.911301
O        2.054603     -0.010099      0.004992        1.691018
O        2.054603     -0.010099      3.950112        1.910868
O        2.056718      3.789646      7.856384        1.905735
O        2.055313     11.677516      7.856544        1.905152
O        2.056039      0.140600      7.857536        1.904973
O        2.057049      8.031026      7.859552        1.904115
O        3.880570      5.910336      7.860704        1.905091
O        3.880886     13.799784      7.860736        1.904637
O        4.013438      1.965478     11.693088        2.235400
O        4.003970      9.855052     11.693056        2.244007
O        4.027656      3.935911      9.814656        1.980646
O        4.025005     11.822045      9.811008        1.983570
O        4.048738      5.909484     11.691360        2.233908
O        4.042331     13.800099     11.683584        2.235341
O        4.026898     -0.002556      9.813120        1.982559
O        4.032831      7.884745      9.816096        1.982787
O        4.027103      1.962495      0.004992        1.691039
O        4.027103      1.962495      3.950112        1.911431
O        4.027103      3.935106      1.977504        1.897372
O        4.027103      5.907606      0.004992        1.691513
O        4.027103      5.907606      3.950112        1.911576
O        4.027103      7.879901      1.977504        1.897324
O        4.027103      9.852495      0.004992        1.691150
O        4.027103      9.852495      3.950112        1.911225
O        4.027103     11.825106      1.977504        1.897323
O        4.027103     13.797606      0.004992        1.691501
```

O	4.027103	13.797606	3.950112	1.911759
O	4.027103	-0.010099	1.977504	1.897323
O	4.029960	7.879901	5.903104	1.939580
O	4.028287	-0.009216	5.902432	1.939706
O	4.030133	3.938751	5.902656	1.938846
O	4.028760	11.827504	5.902304	1.939069
O	4.177850	1.965399	7.856224	1.905696
O	4.179443	9.853569	7.856192	1.904988
O	6.003706	3.922151	11.689312	2.235706
O	6.000850	11.811172	11.679232	2.238825
O	6.000992	0.004860	11.687936	2.235648
O	6.000235	7.903066	11.710144	2.219742
O	5.999698	3.935106	0.004992	1.691229
O	5.999698	3.935106	3.950112	1.911171
O	5.999698	7.879901	0.004992	1.691325
O	5.999698	7.879901	3.950112	1.911249
O	5.999698	11.825106	0.004992	1.691319
O	5.999698	11.825106	3.950112	1.911314
O	5.999698	-0.010099	0.004992	1.691251
O	5.999698	-0.010099	3.950112	1.911312
O	6.002381	4.087367	7.862752	1.903860
O	6.001828	11.976263	7.860800	1.904299
O	6.002396	7.731821	7.862912	1.904772
O	6.001371	15.624709	7.859360	1.904965
O	7.825649	1.966504	7.862464	1.904791
O	7.822919	9.852811	7.860736	1.905466
O	7.961404	5.912876	11.712512	2.229405
O	7.961862	13.798127	11.683072	2.243610
O	7.987394	1.964673	11.687104	2.238626
O	7.990045	9.852211	11.704000	2.241898
O	7.971772	3.937410	5.903424	1.939243
O	7.972845	11.825280	5.903520	1.938039
O	7.972498	7.881116	5.904352	1.937451
O	7.972703	-0.007890	5.903328	1.937871
O	7.976064	-0.006596	9.813440	1.982356
O	7.975417	7.880248	9.824448	1.975240
O	7.971993	1.962495	0.004992	1.691108
O	7.971993	1.962495	3.950112	1.911733
O	7.971993	3.935106	1.977504	1.897393
O	7.971993	5.907606	0.004992	1.691582
O	7.971993	5.907606	3.950112	1.911363
O	7.971993	7.879901	1.977504	1.897456
O	7.971993	9.852495	0.004992	1.691258
O	7.971993	9.852495	3.950112	1.911689
O	7.971993	11.825106	1.977504	1.897347
O	7.971993	13.797606	0.004992	1.691569
O	7.971993	13.797606	3.950112	1.911549
O	7.971993	-0.010099	1.977504	1.897404
O	7.978163	3.946515	9.817856	1.980553
O	7.979204	11.818904	9.816832	1.979878
O	8.124854	5.910541	7.860256	1.905803
O	8.122534	13.798269	7.861472	1.904550
O	9.946323	15.756661	11.693056	2.239675
O	9.946686	3.943817	11.694400	2.241761
O	9.942315	7.866977	11.715264	2.235267
O	9.943641	11.833564	11.702880	2.218954
O	9.944603	3.935106	0.004992	1.691074
O	9.944603	3.935106	3.950112	1.910875
O	9.944603	7.879901	0.004992	1.691181
O	9.944603	7.879901	3.950112	1.911008
O	9.944603	11.825106	0.004992	1.691172
O	9.944603	11.825106	3.950112	1.911039
O	9.944603	-0.010099	0.004992	1.691098
O	9.944603	-0.010099	3.950112	1.910882
O	9.946055	3.787689	7.856320	1.905282
O	9.946055	11.676080	7.858080	1.905053
O	9.946229	0.140442	7.858016	1.905130
O	9.944777	8.032667	7.860864	1.905250
O	9.135989	8.774169	17.267104	2.482516
O	11.769245	5.910099	7.862496	1.904650
O	11.769639	13.797874	7.861760	1.904803
O	11.904779	1.962306	11.687904	2.239372

O	11.891840	9.851833	11.705120	2.234549
O	11.914579	3.935895	9.813440	1.982302
O	11.915162	11.820751	9.817984	1.978934
O	11.934240	5.907953	11.690592	2.234438
O	11.931069	13.796486	11.693696	2.232884
O	11.918129	-0.010178	9.814656	1.981603
O	11.907667	7.885913	9.821504	1.976590
O	11.917103	1.962495	0.004992	1.691037
O	11.917103	1.962495	3.950112	1.911597
O	11.917103	3.935106	1.977504	1.897354
O	11.917103	5.907606	0.004992	1.691513
O	11.917103	5.907606	3.950112	1.911751
O	11.917103	7.879901	1.977504	1.897312
O	11.917103	9.852495	0.004992	1.691157
O	11.917103	9.852495	3.950112	1.911423
O	11.917103	11.825106	1.977504	1.897372
O	11.917103	13.797606	0.004992	1.691508
O	11.917103	13.797606	3.950112	1.911763
O	11.917103	-0.010099	1.977504	1.897316
O	11.917498	7.880248	5.904416	1.937009
O	11.918050	-0.009941	5.902912	1.939064
O	11.919281	3.937220	5.903008	1.938619
O	11.918066	11.826447	5.903264	1.938364
O	12.067471	1.963600	7.859264	1.905243
O	12.068623	9.853490	7.858784	1.905543
O	13.892128	3.917637	11.684256	2.236101
O	13.888404	11.805255	11.691456	2.233349
O	13.891465	0.005428	11.692160	2.234800
O	13.890313	7.901488	11.688160	2.237849
O	13.889698	3.935106	0.004992	1.691167
O	13.889698	3.935106	3.950112	1.911052
O	13.889698	7.879901	0.004992	1.691239
O	13.889698	7.879901	3.950112	1.911166
O	13.889698	11.825106	0.004992	1.691239
O	13.889698	11.825106	3.950112	1.911211
O	13.889698	-0.010099	0.004992	1.691195
O	13.889698	-0.010099	3.950112	1.911174
O	13.891087	4.085600	7.861728	1.904148
O	13.889887	11.975773	7.860832	1.904876
O	13.888941	7.733147	7.864864	1.904891
O	13.891024	15.622926	7.859232	1.905473
Pd	0.102570	-0.000631	15.757344	2.926789
Pd	0.096637	7.868113	15.760736	2.918018
Pd	0.092629	1.968018	13.817472	3.500403
Pd	0.085370	9.850823	13.815360	3.495994
Pd	0.073061	3.942523	15.759264	2.917168
Pd	0.084991	5.901215	13.816704	3.500296
Pd	0.087421	11.829793	15.763360	2.924977
Pd	0.088684	13.803729	13.820288	3.507533
Pd	2.059448	0.000394	13.819328	3.504422
Pd	2.054083	7.868381	13.811328	3.491592
Pd	2.066359	3.942475	13.825184	3.495319
Pd	2.048765	11.833785	13.815392	3.494237
Pd	2.066722	5.899874	15.773664	2.876205
Pd	2.057538	13.791041	15.780416	2.876327
Pd	2.057475	1.985187	15.782656	2.862079
Pd	2.054588	9.853553	15.768960	2.874932
Pd	4.050458	5.908805	13.825312	3.487662
Pd	4.013706	13.811555	13.815296	3.504144
Pd	4.033384	1.966156	13.823008	3.506053
Pd	4.028571	9.848330	13.807584	3.491686
Pd	4.046639	3.932613	15.761792	2.923160
Pd	4.018961	11.833454	15.758944	2.946852
Pd	4.019245	-0.001215	15.758176	2.919841
Pd	4.011670	7.885755	15.770752	2.909188
Pd	5.992455	-0.002130	13.816832	3.502289
Pd	6.011864	7.889558	13.853408	3.496278
Pd	6.000976	3.933449	13.820352	3.493886
Pd	5.972509	11.847466	13.802752	3.497965
Pd	6.001891	5.913729	15.781088	2.938417
Pd	5.988952	13.805149	15.774368	2.887287
Pd	6.013127	1.949982	15.780544	2.873179

Pd	5.969811	9.890210	15.758144	2.877703
Pd	7.982613	15.760922	15.759360	2.919707
Pd	7.913954	7.861138	15.841152	3.018403
Pd	7.970399	1.953122	13.815584	3.488444
Pd	7.966896	9.875250	13.824192	3.423840
Pd	7.959653	3.929046	15.772256	2.921194
Pd	7.989635	5.889538	13.844736	3.490319
Pd	7.942642	11.864619	15.750656	2.921865
Pd	7.965918	13.808463	13.806336	3.485750
Pd	9.953109	-0.014565	13.813728	3.496424
Pd	9.966285	7.884524	13.846720	3.384070
Pd	9.948012	3.938688	13.812000	3.505370
Pd	9.964928	11.834227	13.844320	3.491072
Pd	9.981828	5.850041	15.763744	2.773191
Pd	9.951736	13.799184	15.775040	2.876567
Pd	9.939222	1.976587	15.768352	2.870855
Pd	9.976053	9.965496	15.815680	3.093306
Pd	11.932331	5.893562	13.817920	3.502092
Pd	11.938012	13.802198	13.827968	3.492742
Pd	11.927960	1.954400	13.814432	3.495154
Pd	11.952529	9.861174	13.829600	3.491982
Pd	11.947685	3.906102	15.764512	2.938685
Pd	11.956743	11.837399	15.781216	2.962150
Pd	11.911312	0.000757	15.763552	2.910509
Pd	11.942162	7.885582	15.760320	2.795880
Pd	13.900618	-0.009216	13.822080	3.502139
Pd	13.896215	7.877913	13.812800	3.502145
Pd	13.898645	3.924076	13.813664	3.501338
Pd	13.911380	11.839844	13.823840	3.499692
Pd	13.884128	5.912813	15.771264	2.886757
Pd	13.901833	13.814664	15.783552	2.867518
Pd	13.900854	1.950550	15.779680	2.881818
Pd	13.919033	9.850365	15.783744	2.872996
Sr	2.055361	1.961722	5.891360	1.597348
Sr	2.054745	9.851249	5.891776	1.597634
Sr	2.055140	5.905255	5.892192	1.596757
Sr	2.055361	13.795002	5.891968	1.597030
Sr	2.056986	1.963442	9.829056	1.611325
Sr	2.055108	9.852211	9.830688	1.611764
Sr	2.056450	5.906422	9.829344	1.610948
Sr	2.054808	13.797417	9.829056	1.611923
Sr	2.054603	1.962495	1.977504	1.567425
Sr	2.054603	5.907606	1.977504	1.567549
Sr	2.054603	9.852495	1.977504	1.567407
Sr	2.054603	13.797606	1.977504	1.567546
Sr	6.000582	5.906170	5.892800	1.597383
Sr	6.000377	13.795933	5.892192	1.596986
Sr	6.000834	1.960507	5.891648	1.596974
Sr	6.001323	9.851202	5.891840	1.597630
Sr	6.004416	5.909263	9.834560	1.609234
Sr	6.001639	13.796770	9.829728	1.610903
Sr	6.000455	1.962716	9.829376	1.610921
Sr	6.003675	9.849702	9.836000	1.609240
Sr	5.999698	1.962495	1.977504	1.567535
Sr	5.999698	5.907606	1.977504	1.567683
Sr	5.999698	9.852495	1.977504	1.567516
Sr	5.999698	13.797606	1.977504	1.567656
Sr	9.945503	1.960602	5.891584	1.597350
Sr	9.945392	9.850886	5.892448	1.597290
Sr	9.946181	5.906202	5.893088	1.596834
Sr	9.945566	13.794876	5.892800	1.596659
Sr	9.946876	1.961975	9.831136	1.610834
Sr	9.945187	9.851722	9.838368	1.610849
Sr	9.945219	5.909768	9.836800	1.608616
Sr	9.946513	13.794797	9.832672	1.610551
Sr	9.944603	1.962495	1.977504	1.567438
Sr	9.944603	5.907606	1.977504	1.567535
Sr	9.944603	9.852495	1.977504	1.567399
Sr	9.944603	13.797606	1.977504	1.567520
Sr	13.889856	5.906770	5.893056	1.596423
Sr	13.889808	13.795681	5.891904	1.597276
Sr	13.890014	1.961044	5.891648	1.597084

287	Sr	13.890392	9.850744	5.891840	1.597228
288	Sr	13.889540	5.907795	9.832768	1.610547
289	Sr	13.890250	13.796280	9.830432	1.610275
290	Sr	13.891592	1.961249	9.829472	1.611083
291	Sr	13.888925	9.851028	9.833440	1.609550
292	Sr	13.889698	1.962495	1.977504	1.567533
293	Sr	13.889698	5.907606	1.977504	1.567655
294	Sr	13.889698	9.852495	1.977504	1.567520
295	Sr	13.889698	13.797606	1.977504	1.567681
296	Ti	0.082892	3.931824	7.854848	2.606931
297	Ti	0.082640	11.820908	7.854528	2.606781
298	Ti	0.083902	7.876713	7.856480	2.606393
299	Ti	0.083476	-0.013807	7.853920	2.607004
300	Ti	0.084723	-0.008458	11.747744	2.815191
301	Ti	0.083886	7.879049	11.750368	2.814927
302	Ti	0.087027	3.935327	11.748000	2.815141
303	Ti	0.083508	11.826195	11.747968	2.815193
304	Ti	0.081993	3.935106	0.004992	2.389032
305	Ti	0.081993	3.935106	3.950112	2.604818
306	Ti	0.081993	7.879901	0.004992	2.389185
307	Ti	0.081993	7.879901	3.950112	2.604424
308	Ti	0.081993	11.825106	0.004992	2.389107
309	Ti	0.081993	11.825106	3.950112	2.604920
310	Ti	0.081993	-0.010099	0.004992	2.389131
311	Ti	0.081993	-0.010099	3.950112	2.604951
312	Ti	4.029391	7.876776	7.853984	2.606592
313	Ti	4.027845	-0.013555	7.853952	2.607254
314	Ti	4.029281	3.931981	7.855232	2.606356
315	Ti	4.029281	11.820119	7.855072	2.607071
316	Ti	4.029833	3.937599	11.750176	2.813096
317	Ti	4.025904	11.829335	11.745568	2.816853
318	Ti	4.028097	-0.006612	11.746656	2.813972
319	Ti	4.029313	7.878780	11.746688	2.813071
320	Ti	4.027103	3.935106	0.004992	2.388987
321	Ti	4.027103	3.935106	3.950112	2.604929
322	Ti	4.027103	7.879901	0.004992	2.389187
323	Ti	4.027103	7.879901	3.950112	2.604969
324	Ti	4.027103	11.825106	0.004992	2.389084
325	Ti	4.027103	11.825106	3.950112	2.604994
326	Ti	4.027103	-0.010099	0.004992	2.389111
327	Ti	4.027103	-0.010099	3.950112	2.605115
328	Ti	7.973934	3.932329	7.854656	2.606363
329	Ti	7.973445	11.820135	7.856512	2.605588
330	Ti	7.974707	7.876697	7.856992	2.604902
331	Ti	7.974234	-0.013776	7.856224	2.606073
332	Ti	7.972861	-0.008900	11.748928	2.815312
333	Ti	7.974881	7.879475	11.760192	2.810388
334	Ti	7.972703	3.933260	11.749696	2.814261
335	Ti	7.975764	11.825958	11.752576	2.810576
336	Ti	7.971993	3.935106	0.004992	2.389115
337	Ti	7.971993	3.935106	3.950112	2.604438
338	Ti	7.971993	7.879901	0.004992	2.389328
339	Ti	7.971993	7.879901	3.950112	2.604080
340	Ti	7.971993	11.825106	0.004992	2.389245
341	Ti	7.971993	11.825106	3.950112	2.604461
342	Ti	7.971993	-0.010099	0.004992	2.389221
343	Ti	7.971993	-0.010099	3.950112	2.604626
344	Ti	11.917640	7.877423	7.857792	2.605638
345	Ti	11.917782	-0.013571	7.855104	2.606358
346	Ti	11.917908	3.932029	7.855872	2.606396
347	Ti	11.918287	11.820546	7.856224	2.605785
348	Ti	11.922374	3.933638	11.748256	2.815203
349	Ti	11.919234	11.827410	11.753664	2.808926
350	Ti	11.920812	-0.009878	11.749152	2.815169
351	Ti	11.915336	7.881479	11.758176	2.809813
352	Ti	11.917103	3.935106	0.004992	2.388985
353	Ti	11.917103	3.935106	3.950112	2.604752
354	Ti	11.917103	7.879901	0.004992	2.389190
355	Ti	11.917103	7.879901	3.950112	2.604208
356	Ti	11.917103	11.825106	0.004992	2.389084
357	Ti	11.917103	11.825106	3.950112	2.604563
358	Ti	11.917103	-0.010099	0.004992	2.389114

```
Ti      11.917103      -0.010099      3.950112       2.604954
```

1.2.5 COOH

```
      356
jmolscript: load "" {1 1 1} spacegroup "x,y,z" unitcell [{      15.780000       0.000000      0.000000 }, {       0.000000     15.780000      0.000000 }, {      0.000000      0.000000     32.000000 }]
C        9.465128       8.371827     17.121344        4.145937
H        8.310505       9.524066     18.079264        0.916711
O        0.075839       5.897175     11.691424        2.241048
O        0.067870      13.789953     11.689408        2.236633
O        0.081993       1.962495      0.004992        1.690390
O        0.081993       1.962495      3.950112        1.911851
O        0.081993       3.935106      1.977504        1.897499
O        0.081993       5.907606      0.004992        1.690749
O        0.081993       5.907606      3.950112        1.911674
O        0.081993       7.879901      1.977504        1.897608
O        0.081993       9.852495      0.004992        1.690383
O        0.081993       9.852495      3.950112        1.911845
O        0.081993      11.825106      1.977504        1.897532
O        0.081993      13.797606      0.004992        1.690806
O        0.081993      13.797606      3.950112        1.911795
O        0.081993      -0.010099      1.977504        1.897599
O        0.091966       3.930908      9.812256        1.982668
O        0.083981      11.818762      9.814304        1.981108
O        0.081362       3.934364      5.902656        1.939119
O        0.083271      11.822644      5.902752        1.938782
O        0.088620       7.876792      9.814848        1.983185
O        0.083997      -0.016317      9.812224        1.982489
O        0.084249       7.878844      5.902912        1.939877
O        0.083271      -0.011425      5.902240        1.940055
O        0.104448       1.953438     11.686336        2.237314
O        0.103943       9.848093     11.696672        2.238867
O        0.238878       5.904876      7.858368        1.903962
O        0.236684      13.793803      7.857888        1.904456
O        2.059022       7.857020     11.688384        2.243930
O        2.056907      15.742680     11.689344        2.236242
O        2.059022       3.938893     11.693696        2.238605
O        2.057081      11.834274     11.689152        2.236620
O        2.054603       3.935106      0.004992        1.690305
O        2.054603       3.935106      3.950112        1.911575
O        2.054603       7.879901      0.004992        1.690303
O        2.054603       7.879901      3.950112        1.911131
O        2.054603      11.825106      0.004992        1.690345
O        2.054603      11.825106      3.950112        1.911606
O        2.054603      -0.010099      0.004992        1.690372
O        2.054603      -0.010099      3.950112        1.911178
O        2.059148       3.781282      7.854560        1.905698
O        2.058249      11.670257      7.859136        1.904108
O        2.058059       0.136970      7.857120        1.904607
O        2.060111       8.027933      7.857440        1.904519
O        3.882527       5.904829      7.857792        1.904036
O        3.881580      13.793629      7.859584        1.903766
O        4.014748       1.953674     11.687104        2.235326
O        4.013517       9.844274     11.694464        2.238222
O        4.028918       7.874741      9.815040        1.982582
O        4.029502      -0.012687      9.811616        1.983243
O        4.027829       3.931555      9.812928        1.981840
O        4.032090      11.814123      9.812704        1.981181
O        4.027103       1.962495      0.004992        1.690369
O        4.027103       1.962495      3.950112        1.911477
O        4.027103       3.935106      1.977504        1.897489
O        4.027103       5.907606      0.004992        1.690757
O        4.027103       5.907606      3.950112        1.911948
O        4.027103       7.879901      1.977504        1.897489
O        4.027103       9.852495      0.004992        1.690375
```

62	O	4.027103	9.852495	3.950112	1.911254
63	O	4.027103	11.825106	1.977504	1.897488
64	O	4.027103	13.797606	0.004992	1.690821
65	O	4.027103	13.797606	3.950112	1.912285
66	O	4.027103	-0.010099	1.977504	1.897487
67	O	4.032295	7.877676	5.902912	1.940466
68	O	4.029991	-0.012387	5.902400	1.939777
69	O	4.032532	3.934238	5.902304	1.939085
70	O	4.028429	11.823623	5.902208	1.939440
71	O	4.044367	5.899037	11.693440	2.237859
72	O	4.045203	13.789527	11.680960	2.237142
73	O	4.183136	1.959797	7.857408	1.904598
74	O	4.184809	9.848124	7.856800	1.904631
75	O	6.001718	-0.004718	11.691776	2.234026
76	O	6.006263	7.887207	11.691936	2.225462
77	O	6.003769	3.912588	11.686848	2.236921
78	O	6.002444	11.803314	11.691232	2.242870
79	O	5.999698	3.935106	0.004992	1.690450
80	O	5.999698	3.935106	3.950112	1.911451
81	O	5.999698	7.879901	0.004992	1.690452
82	O	5.999698	7.879901	3.950112	1.911064
83	O	5.999698	11.825106	0.004992	1.690521
84	O	5.999698	11.825106	3.950112	1.911450
85	O	5.999698	-0.010099	0.004992	1.690538
86	O	5.999698	-0.010099	3.950112	1.911420
87	O	6.005852	4.082980	7.863424	1.904182
88	O	6.004258	11.972428	7.856128	1.905504
89	O	6.006389	7.725115	7.864736	1.903024
90	O	6.003943	15.616251	7.859840	1.904915
91	O	7.826501	1.960255	7.864800	1.903748
92	O	7.826801	9.848203	7.865088	1.903087
93	O	7.963614	5.902903	11.706112	2.244272
94	O	7.965570	13.789763	11.696640	2.237962
95	O	7.971993	1.962495	0.004992	1.690336
96	O	7.971993	1.962495	3.950112	1.911905
97	O	7.971993	3.935106	1.977504	1.897565
98	O	7.971993	5.907606	0.004992	1.690681
99	O	7.971993	5.907606	3.950112	1.911349
100	O	7.971993	7.879901	1.977504	1.897618
101	O	7.971993	9.852495	0.004992	1.690330
102	O	7.971993	9.852495	3.950112	1.911905
103	O	7.971993	11.825106	1.977504	1.897497
104	O	7.971993	13.797606	0.004992	1.690780
105	O	7.971993	13.797606	3.950112	1.911545
106	O	7.971993	-0.010099	1.977504	1.897598
107	O	7.981193	3.937441	9.820736	1.978095
108	O	7.972529	11.825816	9.815232	1.981147
109	O	7.973713	3.932076	5.904192	1.938452
110	O	7.975843	11.821050	5.903264	1.938494
111	O	7.983623	7.867435	9.819456	1.980321
112	O	7.976364	15.761632	9.816384	1.979315
113	O	7.975575	7.879617	5.904832	1.937868
114	O	7.973871	-0.008868	5.903424	1.938658
115	O	7.990392	1.955079	11.691584	2.241376
116	O	7.989272	9.842633	11.682304	2.245931
117	O	8.130945	5.904119	7.858112	1.904865
118	O	8.128515	13.794481	7.856992	1.904450
119	O	8.975443	8.835601	18.306656	2.272744
120	O	9.952935	7.842155	11.694560	2.248033
121	O	9.946986	15.748345	11.694688	2.240961
122	O	9.948075	3.934538	11.710048	2.202932
123	O	9.944966	11.818100	11.693888	2.225385
124	O	9.944603	3.935106	0.004992	1.690241
125	O	9.944603	3.935106	3.950112	1.911290
126	O	9.944603	7.879901	0.004992	1.690225
127	O	9.944603	7.879901	3.950112	1.911042
128	O	9.944603	11.825106	0.004992	1.690299
129	O	9.944603	11.825106	3.950112	1.911276
130	O	9.944603	-0.010099	0.004992	1.690340
131	O	9.944603	-0.010099	3.950112	1.911102
132	O	9.948785	3.779610	7.858976	1.905711
133	O	9.948675	11.669278	7.862016	1.904416

O	9.948375	0.137665	7.859104	1.905173
O	9.951215	8.028438	7.863360	1.904455
O	10.368896	7.442400	17.267488	2.413854
O	11.770097	5.904687	7.861184	1.903957
O	11.770334	13.792493	7.862272	1.903411
O	11.902160	1.953611	11.689216	2.243708
O	11.899114	9.838199	11.704480	2.240843
O	11.927755	7.871269	9.824160	1.974611
O	11.919407	-0.016443	9.815552	1.980254
O	11.912606	3.935753	9.820192	1.978558
O	11.919486	11.813807	9.818912	1.978695
O	11.917103	1.962495	0.004992	1.690261
O	11.917103	1.962495	3.950112	1.911658
O	11.917103	3.935106	1.977504	1.897516
O	11.917103	5.907606	0.004992	1.690626
O	11.917103	5.907606	3.950112	1.911927
O	11.917103	7.879901	1.977504	1.897487
O	11.917103	9.852495	0.004992	1.690237
O	11.917103	9.852495	3.950112	1.911498
O	11.917103	11.825106	1.977504	1.897530
O	11.917103	13.797606	0.004992	1.690714
O	11.917103	13.797606	3.950112	1.912202
O	11.917103	-0.010099	1.977504	1.897414
O	11.919612	7.878559	5.904832	1.937727
O	11.918476	-0.011709	5.903712	1.938115
O	11.919960	3.933512	5.904384	1.937591
O	11.918113	11.821445	5.904288	1.938126
O	11.943093	5.898596	11.709024	2.239763
O	11.929033	13.787885	11.692096	2.238338
O	12.072820	1.959766	7.863520	1.904064
O	12.074872	9.847493	7.863520	1.904170
O	13.892128	-0.004450	11.693504	2.234044
O	13.904863	7.885692	11.708096	2.205047
O	13.892018	3.905171	11.679008	2.241952
O	13.888057	11.800410	11.689536	2.235966
O	13.889698	3.935106	0.004992	1.690416
O	13.889698	3.935106	3.950112	1.911459
O	13.889698	7.879901	0.004992	1.690403
O	13.889698	7.879901	3.950112	1.910961
O	13.889698	11.825106	0.004992	1.690464
O	13.889698	11.825106	3.950112	1.911251
O	13.889698	-0.010099	0.004992	1.690500
O	13.889698	-0.010099	3.950112	1.911198
O	13.892223	4.082917	7.864576	1.903838
O	13.892396	11.972570	7.859424	1.905211
O	13.894164	7.723947	7.861024	1.904767
O	13.892460	15.615588	7.858464	1.904819
O	15.715696	1.959497	7.858336	1.903685
O	15.715050	9.848661	7.859520	1.903298
Pd	0.060232	5.904355	13.810432	3.480379
Pd	0.086664	13.792367	13.818720	3.509916
Pd	0.088873	3.891017	15.754880	2.950788
Pd	0.080210	11.830282	15.757536	2.939048
Pd	0.087705	7.883167	15.765216	2.900439
Pd	0.098515	15.759628	15.757216	2.919606
Pd	0.101939	1.929247	13.815648	3.512882
Pd	0.054394	9.836810	13.820320	3.477156
Pd	2.072119	1.954984	15.784128	2.871764
Pd	2.050942	9.859107	15.771680	2.863860
Pd	2.026420	5.891558	15.771872	2.865472
Pd	2.056939	13.776398	15.786016	2.876336
Pd	2.054603	3.919310	13.822688	3.493682
Pd	2.053467	11.821350	13.819808	3.491080
Pd	2.035257	7.871159	13.810816	3.491587
Pd	2.060994	15.751201	13.817088	3.518433
Pd	4.023395	1.939141	13.819200	3.502557
Pd	4.016262	9.853616	13.817120	3.501646
Pd	3.989831	7.861770	15.766368	2.916279
Pd	4.013517	15.741733	15.761312	2.932304
Pd	4.010803	5.894382	13.816512	3.500474
Pd	4.025794	13.797006	13.813504	3.500877
Pd	4.026819	3.929899	15.762752	2.904906

206	Pd	4.033147	11.825832	15.761632	2.924321
207	Pd	5.980983	1.930667	15.777088	2.880024
208	Pd	5.972762	9.839461	15.760320	2.933771
209	Pd	5.970126	3.911562	13.813088	3.505093
210	Pd	5.987989	11.824964	13.807680	3.502285
211	Pd	5.983618	5.918510	15.779936	2.872852
212	Pd	6.004921	13.794008	15.780576	2.868485
213	Pd	5.951459	7.868839	13.828224	3.464160
214	Pd	6.004038	15.755857	13.822688	3.500577
215	Pd	7.936456	5.877040	13.821376	3.475661
216	Pd	7.966107	13.792683	13.822688	3.494993
217	Pd	7.899073	3.919847	15.783168	2.923298
218	Pd	7.949554	11.814707	15.763392	2.959581
219	Pd	7.965034	7.886386	15.787872	3.244257
220	Pd	7.984869	-0.006722	15.764416	2.900684
221	Pd	7.961326	1.968744	13.819936	3.468334
222	Pd	7.958485	9.860843	13.801408	3.435402
223	Pd	9.943309	1.988974	15.757120	2.859617
224	Pd	9.930780	9.852480	15.781600	3.259429
225	Pd	9.945361	5.790234	15.828736	2.888135
226	Pd	9.940611	13.791199	15.783648	2.860209
227	Pd	9.935435	4.002786	13.873504	3.509218
228	Pd	9.957606	11.841470	13.833216	3.458352
229	Pd	9.945156	7.867955	13.808288	3.317099
230	Pd	9.939380	-0.011141	13.817728	3.489588
231	Pd	11.917829	1.964720	13.813664	3.471308
232	Pd	11.944608	9.877223	13.820832	3.471227
233	Pd	12.007996	7.859403	15.825280	2.906534
234	Pd	11.900156	-0.007669	15.762336	2.907861
235	Pd	11.940505	5.880464	13.829696	3.485143
236	Pd	11.926966	13.804234	13.820640	3.495665
237	Pd	11.979813	3.905440	15.777280	2.919422
238	Pd	11.921680	11.830771	15.772832	2.904164
239	Pd	13.894795	1.935291	15.778272	2.886818
240	Pd	13.894984	9.886391	15.795264	2.887866
241	Pd	13.926039	3.893036	13.798784	3.506026
242	Pd	13.912895	11.842259	13.818208	3.509148
243	Pd	13.940604	5.847658	15.785120	2.878287
244	Pd	13.885990	13.806332	15.788736	2.847671
245	Pd	13.831391	7.874993	13.877760	3.517314
246	Pd	13.886731	15.760086	13.823392	3.499962
247	Sr	2.054730	1.954732	9.828352	1.612590
248	Sr	2.052757	9.845331	9.830880	1.611885
249	Sr	2.052631	1.959119	5.890656	1.598116
250	Sr	2.052126	9.849071	5.890848	1.598398
251	Sr	2.053956	5.898958	9.830784	1.611516
252	Sr	2.055029	13.788895	9.828288	1.612638
253	Sr	2.053073	5.902825	5.891456	1.598133
254	Sr	2.054177	13.792951	5.891232	1.597684
255	Sr	2.054603	1.962495	1.977504	1.567509
256	Sr	2.054603	5.907606	1.977504	1.567629
257	Sr	2.054603	9.852495	1.977504	1.567532
258	Sr	2.054603	13.797606	1.977504	1.567632
259	Sr	5.999777	5.899495	9.835744	1.610880
260	Sr	6.000818	13.789495	9.830048	1.611102
261	Sr	5.997031	5.903061	5.892608	1.597680
262	Sr	5.998120	13.793803	5.891488	1.598081
263	Sr	5.999903	1.954905	9.830112	1.611021
264	Sr	6.000329	9.843501	9.833120	1.610610
265	Sr	5.999225	1.959087	5.891456	1.597540
266	Sr	5.999225	9.849261	5.891520	1.597986
267	Sr	5.999698	1.962495	1.977504	1.567622
268	Sr	5.999698	5.907606	1.977504	1.567756
269	Sr	5.999698	9.852495	1.977504	1.567616
270	Sr	5.999698	13.797606	1.977504	1.567737
271	Sr	9.944067	1.957320	9.832320	1.611974
272	Sr	9.945408	9.844164	9.835104	1.610524
273	Sr	9.943136	1.959071	5.891776	1.597614
274	Sr	9.942994	9.849040	5.892128	1.597527
275	Sr	9.945503	5.898122	9.837888	1.610842
276	Sr	9.944761	13.789164	9.831520	1.611003
277	Sr	9.944240	5.903330	5.892544	1.597893

Sr	9.944524	13.793629	5.892192	1.597635
Sr	9.944603	1.962495	1.977504	1.567531
Sr	9.944603	5.907606	1.977504	1.567639
Sr	9.944603	9.852495	1.977504	1.567545
Sr	9.944603	13.797606	1.977504	1.567619
Sr	13.885942	5.901436	9.838752	1.610877
Sr	13.888199	13.789779	9.830016	1.610963
Sr	13.887520	5.903140	5.892768	1.597500
Sr	13.888215	13.793345	5.891616	1.597699
Sr	13.889777	1.955284	9.831104	1.611516
Sr	13.888751	9.842601	9.836288	1.610354
Sr	13.888325	1.959150	5.891424	1.597273
Sr	13.888294	9.848708	5.892128	1.597765
Sr	13.889698	1.962495	1.977504	1.567623
Sr	13.889698	5.907606	1.977504	1.567749
Sr	13.889698	9.852495	1.977504	1.567597
Sr	13.889698	13.797606	1.977504	1.567754
Ti	0.076991	3.927784	7.855552	2.606356
Ti	0.077290	11.816427	7.855520	2.605590
Ti	0.076202	7.870606	7.854208	2.605611
Ti	0.079089	15.761143	7.853376	2.606024
Ti	0.086743	3.924549	11.747328	2.817821
Ti	0.085180	11.818526	11.749472	2.815009
Ti	0.075223	7.873415	11.746688	2.812905
Ti	0.083287	15.760669	11.745248	2.816256
Ti	0.081993	3.935106	0.004992	2.388121
Ti	0.081993	3.935106	3.950112	2.605063
Ti	0.081993	7.879901	0.004992	2.388188
Ti	0.081993	7.879901	3.950112	2.604963
Ti	0.081993	11.825106	0.004992	2.388170
Ti	0.081993	11.825106	3.950112	2.605126
Ti	0.081993	-0.010099	0.004992	2.388282
Ti	0.081993	-0.010099	3.950112	2.605290
Ti	4.020697	7.872642	7.852928	2.606090
Ti	4.023963	15.762374	7.853792	2.606321
Ti	4.022827	3.926679	7.855264	2.605911
Ti	4.024421	11.815512	7.854656	2.605506
Ti	4.021565	7.873115	11.745472	2.814383
Ti	4.029249	-0.017453	11.745152	2.815172
Ti	4.027466	3.924249	11.748864	2.816385
Ti	4.027040	11.821571	11.748000	2.817169
Ti	4.027103	3.935106	0.004992	2.388061
Ti	4.027103	3.935106	3.950112	2.605320
Ti	4.027103	7.879901	0.004992	2.388160
Ti	4.027103	7.879901	3.950112	2.605165
Ti	4.027103	11.825106	0.004992	2.388128
Ti	4.027103	11.825106	3.950112	2.605284
Ti	4.027103	-0.010099	0.004992	2.388252
Ti	4.027103	-0.010099	3.950112	2.605447
Ti	7.967937	3.928683	7.855648	2.605344
Ti	7.968237	11.816948	7.855584	2.605324
Ti	7.967464	7.870307	7.856096	2.605065
Ti	7.969941	15.761474	7.855360	2.605201
Ti	7.976238	3.923839	11.754016	2.808254
Ti	7.978415	11.819252	11.749664	2.814386
Ti	7.969989	7.872800	11.751232	2.816407
Ti	7.973271	-0.015780	11.751008	2.814644
Ti	7.971993	3.935106	0.004992	2.388030
Ti	7.971993	3.935106	3.950112	2.604455
Ti	7.971993	7.879901	0.004992	2.388097
Ti	7.971993	7.879901	3.950112	2.604093
Ti	7.971993	11.825106	0.004992	2.388134
Ti	7.971993	11.825106	3.950112	2.605102
Ti	7.971993	-0.010099	0.004992	2.388239
Ti	7.971993	-0.010099	3.950112	2.604646
Ti	11.911407	7.872926	7.856736	2.604804
Ti	11.913537	-0.016490	7.856096	2.604699
Ti	11.912622	3.927942	7.857216	2.604821
Ti	11.913821	11.816238	7.856224	2.604402
Ti	11.917971	7.873699	11.758880	2.808565
Ti	11.917703	-0.014139	11.750656	2.814936
Ti	11.914105	3.925906	11.755616	2.808847

```
Ti      11.919691      11.823102      11.753248        2.812666
Ti      11.917103       3.935106       0.004992        2.387900
Ti      11.917103       3.935106       3.950112        2.604480
Ti      11.917103       7.879901       0.004992        2.387979
Ti      11.917103       7.879901       3.950112        2.604286
Ti      11.917103      11.825106       0.004992        2.387963
Ti      11.917103      11.825106       3.950112        2.604435
Ti      11.917103      -0.010099       0.004992        2.388128
Ti      11.917103      -0.010099       3.950112        2.604607
```

1.2.6 CO

```
       354
jmolscript: load "" {1 1 1} spacegroup "x,y,z" unitcell [{      15.780000
       0.000000      0.000000 }, {       0.000000     15.780000      0.000000
    }, {       0.000000      0.000000     32.000000 }]
C        8.961983       8.942037      17.221216        4.137329
O       15.712493       1.964200       7.856960        1.905310
O       15.711436       9.854215       7.858144        1.905426
O        0.060343       5.910478      11.686752        2.237301
O        0.062520      13.801314      11.690016        2.236479
O        0.100550       1.965746      11.689664        2.239269
O        0.101355       9.856472      11.694272        2.241607
O        0.081046       3.937126       5.902688        1.940149
O        0.082166      11.826211       5.902976        1.939736
O        0.081993       7.880879       5.903584        1.938836
O        0.081283      -0.009815       5.902496        1.940344
O        0.082356      -0.006549       9.812576        1.982545
O        0.082372       7.885771       9.814944        1.980680
O        0.081993       1.962495       0.004992        1.689932
O        0.081993       1.962495       3.950112        1.911568
O        0.081993       3.935106       1.977504        1.897370
O        0.081993       5.907606       0.004992        1.690277
O        0.081993       5.907606       3.950112        1.911714
O        0.081993       7.879901       1.977504        1.897450
O        0.081993       9.852495       0.004992        1.689867
O        0.081993       9.852495       3.950112        1.911428
O        0.081993      11.825106       1.977504        1.897398
O        0.081993      13.797606       0.004992        1.690304
O        0.081993      13.797606       3.950112        1.911745
O        0.081993      -0.010099       1.977504        1.897465
O        0.085575       3.935721       9.812384        1.982705
O        0.077590      11.825974       9.814336        1.981265
O        0.232140       5.909752       7.859968        1.904753
O        0.232124      13.798789       7.859200        1.905288
O        2.053593      15.757482      11.689760        2.239453
O        2.055156       3.957624      11.690464        2.235606
O        2.054761       7.862637      11.689664        2.240960
O        2.052868      11.847640      11.686432        2.240259
O        2.054603       3.935106       0.004992        1.689834
O        2.054603       3.935106       3.950112        1.911364
O        2.054603       7.879901       0.004992        1.689783
O        2.054603       7.879901       3.950112        1.911105
O        2.054603      11.825106       0.004992        1.689803
O        2.054603      11.825106       3.950112        1.911449
O        2.054603      -0.010099       0.004992        1.689884
O        2.054603      -0.010099       3.950112        1.911201
O        2.055282       3.787405       7.856704        1.905167
O        2.054193      11.676159       7.860384        1.904488
O        2.054572       0.141294       7.856960        1.904683
O        2.055503       8.032115       7.860992        1.903731
O        3.877256       5.910210       7.860320        1.905260
O        3.877761      13.799010       7.860544        1.904706
O        4.009950       1.967024      11.690080        2.236294
O        4.001540       9.855793      11.696256        2.240287
O        4.027277       3.938578       9.814336        1.980717
O        4.027703      11.822913       9.813312        1.982431
```

O	4.046118	5.911709	11.692096	2.238525
O	4.041589	13.801567	11.682976	2.239538
O	4.024547	-0.004040	9.812448	1.983107
O	4.029818	7.884556	9.817088	1.980954
O	4.027103	1.962495	0.004992	1.689857
O	4.027103	1.962495	3.950112	1.911603
O	4.027103	3.935106	1.977504	1.897370
O	4.027103	5.907606	0.004992	1.690215
O	4.027103	5.907606	3.950112	1.911856
O	4.027103	7.879901	1.977504	1.897343
O	4.027103	9.852495	0.004992	1.689776
O	4.027103	9.852495	3.950112	1.911270
O	4.027103	11.825106	1.977504	1.897350
O	4.027103	13.797606	0.004992	1.690246
O	4.027103	13.797606	3.950112	1.911789
O	4.027103	-0.010099	1.977504	1.897323
O	4.027798	7.880422	5.903552	1.940118
O	4.028019	-0.009468	5.902752	1.940132
O	4.029028	3.937615	5.902848	1.939438
O	4.026977	11.826258	5.903072	1.939077
O	4.177739	1.965004	7.857472	1.905509
O	4.178749	9.853395	7.857664	1.905043
O	5.999793	3.922671	11.691872	2.238870
O	5.998641	11.811977	11.689344	2.240367
O	5.998372	0.007432	11.686720	2.238735
O	6.001544	7.903508	11.702592	2.227091
O	5.999698	3.935106	0.004992	1.689940
O	5.999698	3.935106	3.950112	1.911438
O	5.999698	7.879901	0.004992	1.689863
O	5.999698	7.879901	3.950112	1.911173
O	5.999698	11.825106	0.004992	1.689904
O	5.999698	11.825106	3.950112	1.911511
O	5.999698	-0.010099	0.004992	1.689998
O	5.999698	-0.010099	3.950112	1.911371
O	6.000471	4.087967	7.861568	1.904053
O	6.000061	11.976831	7.859392	1.904643
O	6.000519	7.730291	7.861888	1.904154
O	5.999998	15.621695	7.860320	1.904855
O	7.821767	1.965115	7.860544	1.904863
O	7.820678	9.853237	7.861792	1.905057
O	7.955582	5.913318	11.702816	2.226787
O	7.958280	13.801740	11.691872	2.240512
O	7.985579	1.966614	11.689792	2.241890
O	7.988167	9.852685	11.698816	2.247325
O	7.970730	3.936952	5.903360	1.940400
O	7.972719	11.824932	5.904096	1.938484
O	7.972024	7.881163	5.904480	1.939452
O	7.971709	-0.009373	5.903488	1.938819
O	7.975023	-0.006486	9.814880	1.981057
O	7.972987	7.883136	9.820384	1.980877
O	7.971993	1.962495	0.004992	1.689829
O	7.971993	1.962495	3.950112	1.911582
O	7.971993	3.935106	1.977504	1.897392
O	7.971993	5.907606	0.004992	1.690141
O	7.971993	5.907606	3.950112	1.911507
O	7.971993	7.879901	1.977504	1.897491
O	7.971993	9.852495	0.004992	1.689714
O	7.971993	9.852495	3.950112	1.911408
O	7.971993	11.825106	1.977504	1.897398
O	7.971993	13.797606	0.004992	1.690190
O	7.971993	13.797606	3.950112	1.911715
O	7.971993	-0.010099	1.977504	1.897445
O	7.974344	3.940266	9.817024	1.981317
O	7.971535	11.824222	9.818208	1.979953
O	8.123607	5.909894	7.861664	1.904329
O	8.122613	13.798253	7.861792	1.904844
O	8.932869	8.960200	18.402272	2.301215
O	9.944588	15.758492	11.694144	2.241340
O	9.943972	3.948913	11.696224	2.240401
O	9.940658	7.865004	11.699808	2.245506
O	9.941432	11.835757	11.697408	2.229070
O	9.944603	3.935106	0.004992	1.689758

O	9.944603	3.935106	3.950112	1.911128
O	9.944603	7.879901	0.004992	1.689670
O	9.944603	7.879901	3.950112	1.910984
O	9.944603	11.825106	0.004992	1.689706
O	9.944603	11.825106	3.950112	1.911334
O	9.944603	-0.010099	0.004992	1.689819
O	9.944603	-0.010099	3.950112	1.911138
O	9.943783	3.785685	7.856416	1.905293
O	9.944051	11.675164	7.861600	1.905051
O	9.944446	0.141910	7.857760	1.905031
O	9.943767	8.032541	7.861728	1.904520
O	11.766199	5.909799	7.859776	1.905177
O	11.766041	13.798190	7.859168	1.904838
O	11.900771	1.965068	11.686272	2.239110
O	11.887800	9.853521	11.699008	2.227298
O	11.911154	3.938483	9.813056	1.982382
O	11.916630	11.826921	9.817472	1.979476
O	11.935045	5.910841	11.688800	2.240969
O	11.930674	13.798553	11.694784	2.236611
O	11.915036	-0.011346	9.814336	1.981778
O	11.913395	7.883293	9.818368	1.979009
O	11.917103	1.962495	0.004992	1.689830
O	11.917103	1.962495	3.950112	1.911776
O	11.917103	3.935106	1.977504	1.897348
O	11.917103	5.907606	0.004992	1.690183
O	11.917103	5.907606	3.950112	1.911911
O	11.917103	7.879901	1.977504	1.897330
O	11.917103	9.852495	0.004992	1.689732
O	11.917103	9.852495	3.950112	1.911617
O	11.917103	11.825106	1.977504	1.897391
O	11.917103	13.797606	0.004992	1.690218
O	11.917103	13.797606	3.950112	1.911875
O	11.917103	-0.010099	1.977504	1.897323
O	11.916188	7.881447	5.904032	1.938730
O	11.917261	-0.009421	5.902816	1.939923
O	11.917687	3.936416	5.902880	1.939256
O	11.915667	11.824932	5.903456	1.938850
O	12.067234	1.963553	7.859808	1.904921
O	12.067708	9.853789	7.860640	1.905497
O	13.889745	3.918995	11.682176	2.238533
O	13.886479	11.807464	11.694848	2.236439
O	13.889430	0.009784	11.690016	2.236093
O	13.889619	7.905812	11.692352	2.239507
O	13.889698	3.935106	0.004992	1.689976
O	13.889698	3.935106	3.950112	1.911325
O	13.889698	7.879901	0.004992	1.689919
O	13.889698	7.879901	3.950112	1.911233
O	13.889698	11.825106	0.004992	1.689947
O	13.889698	11.825106	3.950112	1.911380
O	13.889698	-0.010099	0.004992	1.690029
O	13.889698	-0.010099	3.950112	1.911351
O	13.889256	4.086736	7.861312	1.903977
O	13.888688	11.976641	7.858720	1.904863
O	13.888767	7.731411	7.861728	1.904756
O	13.889240	15.621080	7.859360	1.904875
Pd	0.097410	0.017547	15.755520	2.919105
Pd	0.095516	7.898916	15.759936	2.904806
Pd	0.089646	1.967703	13.816064	3.508500
Pd	0.080131	9.860764	13.815456	3.495711
Pd	0.065061	3.920494	15.760448	2.925197
Pd	0.085733	5.905728	13.813216	3.497033
Pd	0.076975	11.824175	15.758656	2.918585
Pd	0.078127	13.810119	13.818624	3.504958
Pd	2.051700	0.003109	13.816416	3.507182
Pd	2.048244	7.879948	13.815552	3.492605
Pd	2.056891	3.939272	13.821504	3.503205
Pd	2.044851	11.844831	13.813664	3.494455
Pd	2.028740	5.892946	15.774304	2.871776
Pd	2.037372	13.798853	15.783488	2.873225
Pd	2.077800	1.991452	15.781568	2.857997
Pd	2.071867	9.874619	15.769824	2.860160
Pd	4.029186	5.910004	13.819200	3.493607

Pd	4.012049	13.817978	13.810336	3.506622
Pd	4.021991	1.972358	13.820928	3.504286
Pd	4.025431	9.862169	13.814720	3.501906
Pd	4.044382	3.962610	15.766848	2.899989
Pd	4.019292	11.866702	15.760896	2.926541
Pd	4.007047	15.758034	15.760768	2.923285
Pd	3.998257	7.862559	15.768768	2.898253
Pd	5.989362	0.004213	13.813312	3.498051
Pd	5.976707	7.896975	13.840480	3.463235
Pd	5.993481	3.943154	13.819328	3.493772
Pd	5.978158	11.852169	13.809408	3.503634
Pd	6.024709	5.939608	15.770944	2.879488
Pd	6.001118	13.824448	15.773600	2.872320
Pd	5.973156	1.948451	15.775232	2.871719
Pd	5.943285	9.874461	15.761472	2.822097
Pd	7.979315	0.015070	15.759456	2.906242
Pd	7.992333	7.912502	15.838560	3.360344
Pd	7.965018	1.966456	13.815712	3.494377
Pd	7.963756	9.887622	13.814528	3.404726
Pd	7.948370	3.912209	15.769056	2.899182
Pd	7.977169	5.894209	13.841312	3.463014
Pd	7.943589	11.845951	15.759776	2.849860
Pd	7.966738	13.808257	13.817024	3.497192
Pd	9.943073	-0.002683	13.815424	3.495548
Pd	9.971177	7.873936	13.815968	3.408950
Pd	9.944540	3.945347	13.814464	3.502048
Pd	9.953977	11.862331	13.832608	3.464708
Pd	9.953361	5.862980	15.762624	2.826619
Pd	9.935467	13.809646	15.782816	2.851462
Pd	9.959926	1.987112	15.770240	2.860626
Pd	9.964707	9.878769	15.816064	3.414234
Pd	11.939038	5.893798	13.809344	3.504770
Pd	11.929238	13.815122	13.822976	3.502717
Pd	11.926066	1.963316	13.813696	3.494727
Pd	11.939716	9.869980	13.834496	3.463319
Pd	11.947433	3.938956	15.760384	2.927397
Pd	11.933025	11.853826	15.769888	2.907027
Pd	11.909703	-0.006659	15.758496	2.919675
Pd	11.930974	7.855126	15.760256	2.854955
Pd	13.892270	-0.001625	13.818432	3.505419
Pd	13.890960	7.885424	13.817344	3.495382
Pd	13.902937	3.926506	13.809824	3.506577
Pd	13.895963	11.844626	13.823072	3.500197
Pd	13.911790	5.914707	15.774816	2.870405
Pd	13.908113	13.823122	15.786400	2.848262
Pd	13.878983	1.958424	15.783552	2.873734
Pd	13.887962	9.855273	15.782016	2.851741
Sr	2.054461	1.963600	5.890720	1.597621
Sr	2.054035	9.854058	5.891360	1.597385
Sr	2.054225	5.908142	5.891392	1.597264
Sr	2.054446	13.798079	5.891104	1.597423
Sr	2.054698	1.966046	9.827744	1.612064
Sr	2.053325	9.855809	9.831136	1.611440
Sr	2.054714	5.910825	9.828992	1.611560
Sr	2.053499	13.801156	9.828736	1.612355
Sr	2.054603	1.962495	1.977504	1.567450
Sr	2.054603	5.907606	1.977504	1.567599
Sr	2.054603	9.852495	1.977504	1.567477
Sr	2.054603	13.797606	1.977504	1.567598
Sr	5.999004	5.908348	5.891552	1.597934
Sr	5.999446	13.798332	5.891776	1.597445
Sr	5.999446	1.963142	5.890848	1.597634
Sr	5.999808	9.854720	5.891520	1.597614
Sr	6.000408	5.911598	9.831904	1.610589
Sr	5.999461	13.800020	9.830592	1.610608
Sr	5.998799	1.966409	9.828800	1.611579
Sr	6.000345	9.855872	9.833856	1.609798
Sr	5.999698	1.962495	1.977504	1.567578
Sr	5.999698	5.907606	1.977504	1.567755
Sr	5.999698	9.852495	1.977504	1.567573
Sr	5.999698	13.797606	1.977504	1.567685
Sr	9.944872	1.963064	5.891008	1.597905

Sr	9.945392	9.854484	5.892032	1.597382
Sr	9.945408	5.908332	5.891936	1.597557
Sr	9.944461	13.798142	5.891808	1.597338
Sr	9.944098	1.965273	9.830112	1.611154
Sr	9.943294	9.855099	9.836256	1.611925
Sr	9.944509	5.912529	9.834048	1.610142
Sr	9.944398	13.799358	9.832640	1.610276
Sr	9.944603	1.962495	1.977504	1.567463
Sr	9.944603	5.907606	1.977504	1.567610
Sr	9.944603	9.852495	1.977504	1.567482
Sr	9.944603	13.797606	1.977504	1.567576
Sr	13.889130	5.908726	5.891680	1.597112
Sr	13.888830	13.798253	5.891104	1.597736
Sr	13.888941	1.963442	5.890720	1.597607
Sr	13.888877	9.853711	5.891584	1.597413
Sr	13.888546	5.910983	9.830560	1.611404
Sr	13.887363	13.799342	9.830432	1.610803
Sr	13.889730	1.965036	9.828256	1.612007
Sr	13.888215	9.856235	9.831808	1.610583
Sr	13.889698	1.962495	1.977504	1.567562
Sr	13.889698	5.907606	1.977504	1.567710
Sr	13.889698	9.852495	1.977504	1.567538
Sr	13.889698	13.797606	1.977504	1.567708
Ti	0.082009	3.937284	7.853920	2.606960
Ti	0.081519	11.826936	7.854528	2.606562
Ti	0.081661	7.882631	7.855488	2.606434
Ti	0.081488	-0.008142	7.853408	2.607463
Ti	0.081472	-0.005744	11.746624	2.815910
Ti	0.082293	7.882789	11.750208	2.814590
Ti	0.084281	3.938372	11.746336	2.816307
Ti	0.080273	11.829856	11.748832	2.815005
Ti	0.081993	3.935106	0.004992	2.387551
Ti	0.081993	3.935106	3.950112	2.604576
Ti	0.081993	7.879901	0.004992	2.387563
Ti	0.081993	7.879901	3.950112	2.604353
Ti	0.081993	11.825106	0.004992	2.387513
Ti	0.081993	11.825106	3.950112	2.604612
Ti	0.081993	-0.010099	0.004992	2.387687
Ti	0.081993	-0.010099	3.950112	2.604932
Ti	4.027561	7.882931	7.853664	2.606515
Ti	4.026946	-0.007748	7.853952	2.607315
Ti	4.027450	3.937789	7.854816	2.606487
Ti	4.027450	11.826715	7.855488	2.606731
Ti	4.026409	3.939083	11.749664	2.815040
Ti	4.023569	11.832302	11.748640	2.817377
Ti	4.025431	-0.003377	11.746528	2.815148
Ti	4.026125	7.882757	11.748320	2.812090
Ti	4.027103	3.935106	0.004992	2.387416
Ti	4.027103	3.935106	3.950112	2.604788
Ti	4.027103	7.879901	0.004992	2.387430
Ti	4.027103	7.879901	3.950112	2.604443
Ti	4.027103	11.825106	0.004992	2.387370
Ti	4.027103	11.825106	3.950112	2.604456
Ti	4.027103	-0.010099	0.004992	2.387582
Ti	4.027103	-0.010099	3.950112	2.604878
Ti	7.972671	3.937978	7.853472	2.606644
Ti	7.972372	11.827047	7.856288	2.605488
Ti	7.972435	7.883057	7.854272	2.605879
Ti	7.972245	-0.007669	7.855552	2.606529
Ti	7.969910	-0.004545	11.750048	2.815939
Ti	7.969389	7.882347	11.751072	2.818880
Ti	7.970068	3.939067	11.747968	2.813342
Ti	7.973350	11.830234	11.753248	2.813970
Ti	7.971993	3.935106	0.004992	2.387401
Ti	7.971993	3.935106	3.950112	2.604321
Ti	7.971993	7.879901	0.004992	2.387357
Ti	7.971993	7.879901	3.950112	2.603890
Ti	7.971993	11.825106	0.004992	2.387339
Ti	7.971993	11.825106	3.950112	2.604266
Ti	7.971993	-0.010099	0.004992	2.387561
Ti	7.971993	-0.010099	3.950112	2.604476
Ti	11.916535	7.882915	7.856192	2.605928

```
Ti      11.916488      -0.007843       7.854528        2.606487
Ti      11.916330       3.937789       7.855328        2.607117
Ti      11.916472      11.827142       7.856256        2.606058
Ti      11.919739       3.936589      11.748096        2.815298
Ti      11.915194      11.827962      11.753792        2.809351
Ti      11.916772      -0.006296      11.748608        2.815509
Ti      11.917340       7.886686      11.753344        2.812698
Ti      11.917103       3.935106       0.004992        2.387378
Ti      11.917103       3.935106       3.950112        2.604600
Ti      11.917103       7.879901       0.004992        2.387379
Ti      11.917103       7.879901       3.950112        2.604261
Ti      11.917103      11.825106       0.004992        2.387315
Ti      11.917103      11.825106       3.950112        2.604404
Ti      11.917103      -0.010099       0.004992        2.387546
Ti      11.917103      -0.010099       3.950112        2.604772
```

1.2.7 CH_2OH

```
       357
jmolscript: load "" {1 1 1} spacegroup "x,y,z" unitcell [{      15.780000
       0.000000      0.000000 }, {      0.000000     15.780000      0.000000
    }, {      0.000000      0.000000     32.000000 }]
C        9.737507       9.348703      17.763904        3.875969
H        9.644468       7.391668      17.645728        0.974155
H        9.135389      10.146966      18.213088        0.977498
H       10.766773       9.283453      18.143872        0.997600
O       15.711720       1.964610       7.859264        1.905316
O       15.710126       9.854200       7.858624        1.905263
O        0.066450       5.909768      11.693280        2.237693
O        0.065945      13.799594      11.690976        2.236241
O        0.104732       1.963931      11.689344        2.236320
O        0.106720       9.855131      11.694720        2.238152
O        0.082482       3.936763       5.902496        1.939327
O        0.081693      11.826116       5.902464        1.939311
O        0.082103       7.881258       5.903424        1.938443
O        0.083366      -0.008695       5.902656        1.939354
O        0.082403      -0.008742       9.813856        1.981165
O        0.083950       7.882867       9.815136        1.980791
O        0.081993       1.962495       0.004992        1.691546
O        0.081993       1.962495       3.950112        1.912073
O        0.081993       3.935106       1.977504        1.897797
O        0.081993       5.907606       0.004992        1.691986
O        0.081993       5.907606       3.950112        1.912226
O        0.081993       7.879901       1.977504        1.897869
O        0.081993       9.852495       0.004992        1.691634
O        0.081993       9.852495       3.950112        1.911923
O        0.081993      11.825106       1.977504        1.897800
O        0.081993      13.797606       0.004992        1.691976
O        0.081993      13.797606       3.950112        1.912318
O        0.081993      -0.010099       1.977504        1.897893
O        0.088273       3.938688       9.814144        1.981036
O        0.084028      11.826005       9.813728        1.981142
O        0.235864       5.910115       7.859072        1.905019
O        0.235895      13.799105       7.858880        1.904778
O        2.057870      15.754389      11.689504        2.235980
O        2.058911       3.955446      11.693120        2.237963
O        2.058596       7.861659      11.688480        2.235109
O        2.057302      11.847198      11.686208        2.242960
O        2.054603       3.935106       0.004992        1.691462
O        2.054603       3.935106       3.950112        1.911874
O        2.054603       7.879901       0.004992        1.691519
O        2.054603       7.879901       3.950112        1.911538
O        2.054603      11.825106       0.004992        1.691514
O        2.054603      11.825106       3.950112        1.911755
O        2.054603      -0.010099       0.004992        1.691483
O        2.054603      -0.010099       3.950112        1.911482
O        2.056671       3.785164       7.856832        1.904691
```

O	2.055739	11.674375	7.856800	1.904387
O	2.056323	0.144371	7.860000	1.904486
O	2.056181	8.034892	7.861568	1.903645
O	3.875757	5.909973	7.860416	1.905114
O	3.877130	13.800052	7.859744	1.904640
O	4.012554	1.965068	11.693824	2.237263
O	4.008010	9.857450	11.694080	2.244465
O	4.026804	3.937000	9.815584	1.980212
O	4.026235	11.825374	9.812736	1.982382
O	4.049606	5.908458	11.695680	2.237439
O	4.047491	13.800951	11.687552	2.237932
O	4.031395	-0.003977	9.813920	1.981569
O	4.034599	7.883120	9.816416	1.981472
O	4.027103	1.962495	0.004992	1.691476
O	4.027103	1.962495	3.950112	1.911823
O	4.027103	3.935106	1.977504	1.897823
O	4.027103	5.907606	0.004992	1.691925
O	4.027103	5.907606	3.950112	1.911949
O	4.027103	7.879901	1.977504	1.897751
O	4.027103	9.852495	0.004992	1.691551
O	4.027103	9.852495	3.950112	1.911716
O	4.027103	11.825106	1.977504	1.897790
O	4.027103	13.797606	0.004992	1.691931
O	4.027103	13.797606	3.950112	1.912224
O	4.027103	-0.010099	1.977504	1.897750
O	4.027703	7.880863	5.903264	1.939177
O	4.027435	-0.008363	5.902624	1.939558
O	4.029518	3.937284	5.902944	1.939136
O	4.029107	11.826652	5.902688	1.938768
O	4.181511	1.965352	7.858016	1.905118
O	4.181826	9.853947	7.858880	1.904448
O	6.004653	3.918332	11.689824	2.233344
O	6.003564	11.810667	11.682816	2.234706
O	6.003596	0.007985	11.691392	2.237533
O	5.997915	7.903066	11.710976	2.215824
O	5.999698	3.935106	0.004992	1.691650
O	5.999698	3.935106	3.950112	1.911679
O	5.999698	7.879901	0.004992	1.691733
O	5.999698	7.879901	3.950112	1.911770
O	5.999698	11.825106	0.004992	1.691730
O	5.999698	11.825106	3.950112	1.911779
O	5.999698	-0.010099	0.004992	1.691684
O	5.999698	-0.010099	3.950112	1.911602
O	6.001718	4.090555	7.862912	1.903293
O	6.001671	11.979545	7.861696	1.903698
O	6.001213	7.728507	7.860032	1.904377
O	6.001891	15.620496	7.857952	1.904498
O	7.821894	1.965920	7.863488	1.904125
O	7.819100	9.852985	7.861216	1.905173
O	7.962856	5.911898	11.711008	2.230907
O	7.962414	13.798300	11.687680	2.239890
O	7.992270	1.965746	11.686176	2.237198
O	8.001533	9.851486	11.705984	2.241393
O	7.971567	3.936337	5.903520	1.938940
O	7.971677	11.825422	5.903456	1.938456
O	7.972308	7.881384	5.903872	1.938380
O	7.973334	-0.007953	5.903360	1.938578
O	7.974581	-0.009074	9.814240	1.981556
O	7.969941	7.879727	9.823200	1.975630
O	7.971993	1.962495	0.004992	1.691555
O	7.971993	1.962495	3.950112	1.911997
O	7.971993	3.935106	1.977504	1.897807
O	7.971993	5.907606	0.004992	1.692011
O	7.971993	5.907606	3.950112	1.912081
O	7.971993	7.879901	1.977504	1.897881
O	7.971993	9.852495	0.004992	1.691673
O	7.971993	9.852495	3.950112	1.911806
O	7.971993	11.825106	1.977504	1.897796
O	7.971993	13.797606	0.004992	1.692012
O	7.971993	13.797606	3.950112	1.912112
O	7.971993	-0.010099	1.977504	1.897866
O	7.978384	3.947178	9.817088	1.979683

O	7.979962	11.819899	9.816576	1.980300
O	8.126905	5.910588	7.859488	1.905467
O	8.125990	13.798332	7.861472	1.903919
O	9.031320	8.137399	17.896640	2.411402
O	9.948296	15.758255	11.694048	2.235297
O	9.950410	3.951722	11.689760	2.247671
O	9.944193	7.868176	11.707584	2.240158
O	9.947759	11.837020	11.695232	2.240697
O	9.944603	3.935106	0.004992	1.691555
O	9.944603	3.935106	3.950112	1.911677
O	9.944603	7.879901	0.004992	1.691651
O	9.944603	7.879901	3.950112	1.911642
O	9.944603	11.825106	0.004992	1.691643
O	9.944603	11.825106	3.950112	1.911704
O	9.944603	-0.010099	0.004992	1.691574
O	9.944603	-0.010099	3.950112	1.911382
O	9.946039	3.784675	7.857472	1.904250
O	9.945850	11.673397	7.856896	1.904306
O	9.946024	0.144182	7.859360	1.904368
O	9.943799	8.035428	7.861664	1.904869
O	11.765221	5.909799	7.860736	1.904652
O	11.765631	13.798663	7.859104	1.904547
O	11.906547	1.963174	11.687040	2.240839
O	11.896179	9.853111	11.693536	2.242761
O	11.918397	3.935074	9.812544	1.982507
O	11.916188	11.825311	9.815200	1.980554
O	11.941326	5.907953	11.685664	2.240301
O	11.936229	13.797937	11.692512	2.234259
O	11.920212	-0.009957	9.814048	1.981079
O	11.910050	7.884225	9.817472	1.979307
O	11.917103	1.962495	0.004992	1.691554
O	11.917103	1.962495	3.950112	1.912034
O	11.917103	3.935106	1.977504	1.897779
O	11.917103	5.907606	0.004992	1.692036
O	11.917103	5.907606	3.950112	1.912242
O	11.917103	7.879901	1.977504	1.897748
O	11.917103	9.852495	0.004992	1.691677
O	11.917103	9.852495	3.950112	1.912047
O	11.917103	11.825106	1.977504	1.897789
O	11.917103	13.797606	0.004992	1.692025
O	11.917103	13.797606	3.950112	1.912367
O	11.917103	-0.010099	1.977504	1.897739
O	11.916251	7.880674	5.903872	1.937888
O	11.917056	-0.009752	5.902336	1.939884
O	11.918318	3.937220	5.902528	1.939026
O	11.917924	11.825911	5.902624	1.939293
O	12.070769	1.963695	7.857632	1.904360
O	12.071448	9.854089	7.858528	1.904923
O	13.895205	3.917985	11.688448	2.237493
O	13.892160	11.807054	11.690944	2.233378
O	13.893580	0.007922	11.692864	2.236899
O	13.890361	7.902940	11.687552	2.240401
O	13.889698	3.935106	0.004992	1.691686
O	13.889698	3.935106	3.950112	1.911603
O	13.889698	7.879901	0.004992	1.691774
O	13.889698	7.879901	3.950112	1.911742
O	13.889698	11.825106	0.004992	1.691759
O	13.889698	11.825106	3.950112	1.911920
O	13.889698	-0.010099	0.004992	1.691706
O	13.889698	-0.010099	3.950112	1.911707
O	13.891418	4.089466	7.859872	1.904131
O	13.890377	11.979561	7.859808	1.904241
O	13.889367	7.729644	7.863040	1.903649
O	13.890913	15.619502	7.857664	1.904779
Pd	0.059980	15.760291	15.765792	2.910959
Pd	0.058433	7.868034	15.763168	2.918375
Pd	0.093181	1.966488	13.819424	3.498197
Pd	0.093039	9.856598	13.816896	3.503441
Pd	0.116614	3.956646	15.763904	2.910702
Pd	0.092218	5.905144	13.818304	3.503164
Pd	0.130311	11.839829	15.764352	2.914255
Pd	0.085417	13.804975	13.820064	3.501121

Pd	2.055582	-0.002698	13.819776	3.500281
Pd	2.068537	7.876871	13.817888	3.491588
Pd	2.063850	3.942822	13.821760	3.495265
Pd	2.054461	11.834653	13.811424	3.491430
Pd	2.079757	5.948003	15.775200	2.867260
Pd	2.070573	13.834263	15.776480	2.887440
Pd	2.048496	1.939851	15.775200	2.869797
Pd	2.058990	9.816612	15.766912	2.869399
Pd	4.055634	5.919157	13.823744	3.483718
Pd	4.022369	13.807532	13.814560	3.506398
Pd	4.028618	1.968886	13.821312	3.504286
Pd	4.048753	9.841134	13.811808	3.479177
Pd	3.998699	3.922340	15.765504	2.922007
Pd	3.981657	11.834258	15.759968	2.938120
Pd	4.060147	0.002225	15.763296	2.908091
Pd	4.072834	7.894813	15.771328	2.897139
Pd	5.993418	-0.001641	13.817376	3.507660
Pd	6.052151	7.877960	13.865056	3.503504
Pd	5.998341	3.933449	13.822880	3.499151
Pd	5.986979	11.846267	13.813312	3.498857
Pd	5.995264	5.848147	15.784224	2.877844
Pd	5.991224	13.765288	15.778400	2.869517
Pd	6.017198	1.998395	15.780128	2.869779
Pd	5.988904	9.941321	15.773664	2.870495
Pd	7.935904	15.762279	15.766176	2.923933
Pd	7.909378	7.893314	15.819936	2.910000
Pd	7.969137	1.950156	13.818208	3.493683
Pd	7.956213	9.885523	13.819456	3.461026
Pd	8.020295	3.935406	15.773344	2.919117
Pd	7.979441	5.883115	13.841312	3.486863
Pd	8.014457	11.851001	15.755360	2.921856
Pd	7.969910	13.826310	13.815872	3.492136
Pd	9.958742	-0.012261	13.819552	3.499429
Pd	9.959941	7.863537	13.829216	3.427012
Pd	9.944477	3.908596	13.801152	3.490521
Pd	9.952162	11.860011	13.812064	3.467606
Pd	10.002847	5.888559	15.758400	2.975665
Pd	9.965259	13.853830	15.778976	2.867742
Pd	9.938891	1.922998	15.775008	2.867548
Pd	9.948675	9.855730	15.816384	3.400543
Pd	11.926918	5.897523	13.806880	3.483697
Pd	11.939306	13.813575	13.826464	3.493632
Pd	11.938075	1.954416	13.814208	3.500247
Pd	11.933436	9.868749	13.812288	3.458525
Pd	11.904905	3.903593	15.756224	2.942544
Pd	11.883634	11.811188	15.764480	2.923981
Pd	11.970566	0.014218	15.766656	2.918176
Pd	11.949011	7.889416	15.741856	2.951493
Pd	13.904389	-0.007117	13.820992	3.507477
Pd	13.918181	7.882741	13.810752	3.487276
Pd	13.903537	3.929283	13.815936	3.501502
Pd	13.904926	11.832018	13.823712	3.492845
Pd	13.876648	5.883557	15.768640	2.874518
Pd	13.882013	13.758740	15.785664	2.856906
Pd	13.918165	2.002703	15.785248	2.876273
Pd	13.920800	9.886785	15.769504	2.868916
Sr	2.056465	1.962385	5.891360	1.597849
Sr	2.056797	9.851943	5.891456	1.597822
Sr	2.056623	5.906217	5.891712	1.597769
Sr	2.056513	13.796328	5.891392	1.597722
Sr	2.058959	1.964736	9.829728	1.611231
Sr	2.059195	9.854200	9.831744	1.611012
Sr	2.059053	5.908032	9.831264	1.610836
Sr	2.057507	13.798521	9.829440	1.611761
Sr	2.054603	1.962495	1.977504	1.567706
Sr	2.054603	5.907606	1.977504	1.567841
Sr	2.054603	9.852495	1.977504	1.567711
Sr	2.054603	13.797606	1.977504	1.567844
Sr	6.002633	5.907259	5.892224	1.597820
Sr	6.001639	13.796249	5.891712	1.597721
Sr	6.001686	1.961785	5.891488	1.597356
Sr	6.002286	9.851407	5.891744	1.598016

Sr	6.006357	5.910699	9.834912	1.609028
Sr	6.002444	13.799184	9.829568	1.611310
Sr	6.001986	1.964215	9.829984	1.611217
Sr	6.004953	9.851044	9.834848	1.609145
Sr	5.999698	1.962495	1.977504	1.567829
Sr	5.999698	5.907606	1.977504	1.567962
Sr	5.999698	9.852495	1.977504	1.567802
Sr	5.999698	13.797606	1.977504	1.567958
Sr	9.947318	1.962306	5.891104	1.597679
Sr	9.947570	9.851754	5.891968	1.597942
Sr	9.947444	5.907085	5.891936	1.597767
Sr	9.947097	13.795697	5.891744	1.597842
Sr	9.949637	1.963632	9.830176	1.610906
Sr	9.947775	9.853000	9.838144	1.611060
Sr	9.948043	5.909247	9.835200	1.608677
Sr	9.948943	13.797022	9.831520	1.610823
Sr	9.944603	1.962495	1.977504	1.567737
Sr	9.944603	5.907606	1.977504	1.567845
Sr	9.944603	9.852495	1.977504	1.567694
Sr	9.944603	13.797606	1.977504	1.567839
Sr	13.892002	5.907495	5.892096	1.597443
Sr	13.891702	13.796454	5.891328	1.598293
Sr	13.891860	1.961991	5.891264	1.597806
Sr	13.891481	9.852038	5.891520	1.597770
Sr	13.894180	5.908789	9.832352	1.610796
Sr	13.893785	13.798332	9.829728	1.611114
Sr	13.894180	1.962622	9.829792	1.611353
Sr	13.892491	9.852322	9.831968	1.610410
Sr	13.889698	1.962495	1.977504	1.567797
Sr	13.889698	5.907606	1.977504	1.567951
Sr	13.889698	9.852495	1.977504	1.567791
Sr	13.889698	13.797606	1.977504	1.567950
Ti	0.086380	3.933859	7.855072	2.605887
Ti	0.086080	11.822928	7.854752	2.606155
Ti	0.088068	7.879191	7.856032	2.605692
Ti	0.086474	-0.011220	7.854560	2.605965
Ti	0.086632	-0.007180	11.749024	2.814941
Ti	0.090814	7.881384	11.750400	2.815028
Ti	0.088352	3.935832	11.749632	2.814919
Ti	0.085196	11.828972	11.748896	2.815345
Ti	0.081993	3.935106	0.004992	2.389821
Ti	0.081993	3.935106	3.950112	2.605433
Ti	0.081993	7.879901	0.004992	2.389980
Ti	0.081993	7.879901	3.950112	2.605030
Ti	0.081993	11.825106	0.004992	2.389898
Ti	0.081993	11.825106	3.950112	2.605398
Ti	0.081993	-0.010099	0.004992	2.389915
Ti	0.081993	-0.010099	3.950112	2.605395
Ti	4.033100	7.878291	7.854880	2.605923
Ti	4.031017	-0.011267	7.854304	2.606170
Ti	4.032169	3.934569	7.855104	2.605741
Ti	4.031995	11.823039	7.855456	2.606177
Ti	4.030543	3.938546	11.750400	2.813951
Ti	4.028555	11.828372	11.747872	2.815869
Ti	4.029107	-0.006060	11.748416	2.815619
Ti	4.036492	7.881495	11.749312	2.811906
Ti	4.027103	3.935106	0.004992	2.389672
Ti	4.027103	3.935106	3.950112	2.605254
Ti	4.027103	7.879901	0.004992	2.389857
Ti	4.027103	7.879901	3.950112	2.605071
Ti	4.027103	11.825106	0.004992	2.389761
Ti	4.027103	11.825106	3.950112	2.605349
Ti	4.027103	-0.010099	0.004992	2.389816
Ti	4.027103	-0.010099	3.950112	2.605405
Ti	7.977027	3.934506	7.855392	2.606020
Ti	7.976743	11.822344	7.855936	2.604845
Ti	7.978179	7.879159	7.856224	2.604830
Ti	7.976806	-0.011377	7.855072	2.605129
Ti	7.975512	-0.005996	11.748160	2.814816
Ti	7.976585	7.880295	11.758592	2.811944
Ti	7.973303	3.933891	11.750688	2.814991
Ti	7.973161	11.832049	11.750656	2.813697

```
Ti       7.971993       3.935106       0.004992        2.389829
Ti       7.971993       3.935106       3.950112        2.604861
Ti       7.971993       7.879901       0.004992        2.390013
Ti       7.971993       7.879901       3.950112        2.604833
Ti       7.971993      11.825106       0.004992        2.389939
Ti       7.971993      11.825106       3.950112        2.604912
Ti       7.971993      -0.010099       0.004992        2.389940
Ti       7.971993      -0.010099       3.950112        2.605075
Ti      11.921553       7.878733       7.857024        2.605909
Ti      11.921143      -0.011598       7.853952        2.606024
Ti      11.921774       3.934459       7.855200        2.606291
Ti      11.921190      11.823118       7.855264        2.605762
Ti      11.924189       3.934159      11.747392        2.818644
Ti      11.922816      11.829430      11.750400        2.814027
Ti      11.922532      -0.008931      11.748192        2.815879
Ti      11.922595       7.882520      11.753440        2.812506
Ti      11.917103       3.935106       0.004992        2.389802
Ti      11.917103       3.935106       3.950112        2.605341
Ti      11.917103       7.879901       0.004992        2.390013
Ti      11.917103       7.879901       3.950112        2.604915
Ti      11.917103      11.825106       0.004992        2.389903
Ti      11.917103      11.825106       3.950112        2.605356
Ti      11.917103      -0.010099       0.004992        2.389926
Ti      11.917103      -0.010099       3.950112        2.605565
```

1.2.8 CH_3OH

```
       358
jmolscript: load "" {1 1 1} spacegroup "x,y,z" unitcell [{      15.780000
       0.000000      0.000000 }, {      0.000000     15.780000      0.000000
    }, {      0.000000      0.000000     32.000000 }]
C        8.799733      10.085108      18.740448        3.850228
H        7.822067      10.166659      18.237952        1.057436
H        8.684807       9.636404      19.737760        0.963069
H        9.318532       8.404176      17.777536        0.959769
H        9.250457      11.079643      18.823360        0.972283
O       15.710678       1.961991       7.858464        1.904781
O       15.709511       9.852275       7.858080        1.904977
O        0.063514       5.908064      11.688768        2.234145
O        0.064509      13.797164      11.690976        2.235517
O        0.101860       1.962290      11.690880        2.238011
O        0.101923       9.852385      11.691264        2.238082
O        0.082703       3.934869       5.902336        1.939476
O        0.081314      11.825216       5.902304        1.939547
O        0.077669       7.879443       5.902496        1.939086
O        0.081788      -0.009941       5.902560        1.939399
O        0.084723      -0.009657       9.814240        1.981845
O        0.090593       7.881715       9.813248        1.981558
O        0.081993       1.962495       0.004992        1.691431
O        0.081993       1.962495       3.950112        1.911805
O        0.081993       3.935106       1.977504        1.897396
O        0.081993       5.907606       0.004992        1.691883
O        0.081993       5.907606       3.950112        1.911725
O        0.081993       7.879901       1.977504        1.897426
O        0.081993       9.852495       0.004992        1.691556
O        0.081993       9.852495       3.950112        1.911810
O        0.081993      11.825106       1.977504        1.897353
O        0.081993      13.797606       0.004992        1.691885
O        0.081993      13.797606       3.950112        1.911848
O        0.081993      -0.010099       1.977504        1.897473
O        0.081346       3.934175       9.814112        1.981489
O        0.084770      11.824364       9.813248        1.982043
O        0.234049       5.907196       7.858656        1.904667
O        0.235012      13.797180       7.858432        1.904687
O        2.055676      15.751486      11.691616        2.237618
O        2.055171       3.953111      11.690912        2.234994
O        2.055045       7.860760      11.694496        2.241040
```

O	2.055645	11.843379	11.692096	2.233790
O	2.054603	3.935106	0.004992	1.691389
O	2.054603	3.935106	3.950112	1.911319
O	2.054603	7.879901	0.004992	1.691507
O	2.054603	7.879901	3.950112	1.911191
O	2.054603	11.825106	0.004992	1.691478
O	2.054603	11.825106	3.950112	1.911533
O	2.054603	-0.010099	0.004992	1.691409
O	2.054603	-0.010099	3.950112	1.911264
O	2.055014	3.782529	7.859136	1.904468
O	2.054950	11.671551	7.856736	1.905109
O	2.055219	0.141452	7.857888	1.904604
O	2.054635	8.031736	7.851456	1.905792
O	3.876357	5.906912	7.857760	1.904776
O	3.875473	13.797275	7.857472	1.904939
O	4.009067	1.962101	11.691008	2.234488
O	4.008877	9.852038	11.689792	2.238349
O	4.028161	3.934727	9.813504	1.981445
O	4.025162	11.826258	9.812992	1.982222
O	4.046844	5.907543	11.688032	2.236287
O	4.046702	13.797401	11.692736	2.237104
O	4.026109	-0.011504	9.814208	1.981180
O	4.017493	7.880232	9.812288	1.982879
O	4.027103	1.962495	0.004992	1.691441
O	4.027103	1.962495	3.950112	1.911770
O	4.027103	3.935106	1.977504	1.897385
O	4.027103	5.907606	0.004992	1.691945
O	4.027103	5.907606	3.950112	1.911726
O	4.027103	7.879901	1.977504	1.897296
O	4.027103	9.852495	0.004992	1.691615
O	4.027103	9.852495	3.950112	1.911866
O	4.027103	11.825106	1.977504	1.897332
O	4.027103	13.797606	0.004992	1.691941
O	4.027103	13.797606	3.950112	1.911783
O	4.027103	-0.010099	1.977504	1.897326
O	4.031506	7.879522	5.902336	1.939287
O	4.028019	-0.009752	5.902528	1.939378
O	4.027056	3.934601	5.902368	1.939343
O	4.028524	11.824948	5.902080	1.939382
O	4.179964	1.961801	7.858944	1.904488
O	4.180627	9.852148	7.858496	1.904671
O	5.999998	3.915349	11.689888	2.238472
O	6.000929	11.803377	11.683968	2.237552
O	6.001292	0.006580	11.689248	2.234077
O	6.000613	7.899452	11.675552	2.239177
O	5.999698	3.935106	0.004992	1.691681
O	5.999698	3.935106	3.950112	1.911248
O	5.999698	7.879901	0.004992	1.691834
O	5.999698	7.879901	3.950112	1.911631
O	5.999698	11.825106	0.004992	1.691814
O	5.999698	11.825106	3.950112	1.911539
O	5.999698	-0.010099	0.004992	1.691691
O	5.999698	-0.010099	3.950112	1.911394
O	6.000187	4.085395	7.858304	1.904821
O	6.000613	11.976121	7.860928	1.904261
O	6.001102	7.727734	7.867264	1.903236
O	6.000361	15.616882	7.860416	1.904568
O	7.821262	1.961186	7.852896	1.905690
O	7.819905	9.851643	7.852800	1.906627
O	7.954950	5.905160	11.678176	2.241076
O	7.957317	13.795870	11.682752	2.239877
O	7.991749	1.960870	11.696992	2.238472
O	7.998014	9.849655	11.708000	2.242829
O	7.972735	3.937978	5.902688	1.938785
O	7.972198	11.827457	5.902912	1.938829
O	7.968979	7.876129	5.902880	1.938746
O	7.971882	-0.014328	5.902688	1.938886
O	7.976648	-0.003961	9.814304	1.981231
O	7.988862	7.890584	9.816768	1.979178
O	7.971993	1.962495	0.004992	1.691586
O	7.971993	1.962495	3.950112	1.911603
O	7.971993	3.935106	1.977504	1.897369

O	7.971993	5.907606	0.004992	1.692083
O	7.971993	5.907606	3.950112	1.911876
O	7.971993	7.879901	1.977504	1.897445
O	7.971993	9.852495	0.004992	1.691820
O	7.971993	9.852495	3.950112	1.911631
O	7.971993	11.825106	1.977504	1.897359
O	7.971993	13.797606	0.004992	1.692077
O	7.971993	13.797606	3.950112	1.911976
O	7.971993	-0.010099	1.977504	1.897444
O	7.972419	3.924454	9.813568	1.981626
O	7.976017	11.812151	9.816736	1.979658
O	8.123970	5.907006	7.866112	1.903817
O	8.124349	13.795712	7.865856	1.903867
O	9.945866	15.750223	11.695264	2.239210
O	9.945471	3.952196	11.691840	2.237203
O	9.944351	7.864752	11.713248	2.245042
O	9.944777	11.836799	11.701664	2.216373
O	9.944603	3.935106	0.004992	1.691539
O	9.944603	3.935106	3.950112	1.910973
O	9.944603	7.879901	0.004992	1.691721
O	9.944603	7.879901	3.950112	1.910950
O	9.944603	11.825106	0.004992	1.691686
O	9.944603	11.825106	3.950112	1.911237
O	9.944603	-0.010099	0.004992	1.691561
O	9.944603	-0.010099	3.950112	1.911040
O	9.945187	3.782561	7.858688	1.904289
O	9.945250	11.671630	7.858144	1.904859
O	9.945298	0.139874	7.856448	1.904936
O	9.945345	8.031562	7.850688	1.906475
O	9.721364	9.287209	17.955680	2.464520
O	11.766152	5.907590	7.863968	1.904301
O	11.766310	13.795886	7.863520	1.904122
O	11.900061	1.961880	11.694880	2.234982
O	11.889331	9.850476	11.708768	2.230456
O	11.918681	3.930025	9.814400	1.981579
O	11.915068	11.816332	9.817152	1.979301
O	11.934730	5.906359	11.685760	2.235642
O	11.933909	13.795996	11.686592	2.239554
O	11.914784	-0.006896	9.814752	1.981222
O	11.902822	7.887160	9.818272	1.978808
O	11.917103	1.962495	0.004992	1.691471
O	11.917103	1.962495	3.950112	1.911521
O	11.917103	3.935106	1.977504	1.897356
O	11.917103	5.907606	0.004992	1.691973
O	11.917103	5.907606	3.950112	1.911700
O	11.917103	7.879901	1.977504	1.897332
O	11.917103	9.852495	0.004992	1.691658
O	11.917103	9.852495	3.950112	1.911668
O	11.917103	11.825106	1.977504	1.897348
O	11.917103	13.797606	0.004992	1.691973
O	11.917103	13.797606	3.950112	1.911812
O	11.917103	-0.010099	1.977504	1.897313
O	11.920985	7.877913	5.903072	1.938437
O	11.918539	-0.012498	5.902816	1.938551
O	11.917103	3.936873	5.902656	1.938549
O	11.917624	11.826305	5.903136	1.938474
O	12.069459	1.961533	7.856448	1.905421
O	12.070580	9.851975	7.856256	1.905585
O	13.890913	3.916107	11.692384	2.238273
O	13.889698	11.802730	11.685792	2.237572
O	13.890487	0.007227	11.690016	2.234099
O	13.890156	7.900367	11.680928	2.237397
O	13.889698	3.935106	0.004992	1.691584
O	13.889698	3.935106	3.950112	1.911217
O	13.889698	7.879901	0.004992	1.691690
O	13.889698	7.879901	3.950112	1.911649
O	13.889698	11.825106	0.004992	1.691676
O	13.889698	11.825106	3.950112	1.911466
O	13.889698	-0.010099	0.004992	1.691600
O	13.889698	-0.010099	3.950112	1.911380
O	13.889903	4.086073	7.858144	1.904908
O	13.889619	11.976389	7.860800	1.904277

183	O	13.888783	7.727529	7.867168	1.903838
184	O	13.890093	15.616914	7.860512	1.904544
185	Pd	0.112922	0.010194	15.762208	2.919837
186	Pd	0.113821	7.897764	15.762976	2.915475
187	Pd	0.082908	1.967135	13.818816	3.495352
188	Pd	0.088005	9.854578	13.817632	3.492378
189	Pd	0.054283	3.920620	15.761632	2.914959
190	Pd	0.084186	5.902998	13.818624	3.496296
191	Pd	0.054315	11.810004	15.764160	2.912663
192	Pd	0.086727	13.804328	13.819232	3.500573
193	Pd	2.058911	-0.011551	13.819168	3.495921
194	Pd	2.054304	7.876429	13.819712	3.498524
195	Pd	2.057428	3.936747	13.819840	3.499852
196	Pd	2.054398	11.828925	13.822976	3.496841
197	Pd	2.041380	5.875588	15.771392	2.871035
198	Pd	2.039297	13.767908	15.774784	2.868726
199	Pd	2.073744	1.993061	15.771488	2.868170
200	Pd	2.072072	9.883835	15.779296	2.868707
201	Pd	4.028413	5.901578	13.815744	3.500683
202	Pd	4.031111	13.797590	13.819776	3.500395
203	Pd	4.031758	1.961422	13.820416	3.497415
204	Pd	4.018677	9.856409	13.816416	3.493715
205	Pd	4.062245	3.953679	15.762208	2.916165
206	Pd	4.060383	11.839908	15.762848	2.916613
207	Pd	4.004270	15.751864	15.762528	2.915794
208	Pd	4.001776	7.866867	15.758112	2.922007
209	Pd	5.998672	-0.007369	13.820096	3.495467
210	Pd	5.983019	7.882315	13.800608	3.482696
211	Pd	6.001481	3.929536	13.815872	3.500681
212	Pd	5.992944	11.828672	13.811136	3.497220
213	Pd	6.011675	5.937651	15.771040	2.902310
214	Pd	6.018555	13.833853	15.777760	2.893377
215	Pd	5.984376	1.932103	15.775424	2.868503
216	Pd	5.986175	9.819421	15.780960	2.882722
217	Pd	8.007230	0.000521	15.764736	2.913405
218	Pd	7.972561	7.863064	15.722976	3.027542
219	Pd	7.975906	1.958929	13.823808	3.498304
220	Pd	7.964324	9.861585	13.824896	3.472861
221	Pd	7.945861	3.921362	15.758304	2.916404
222	Pd	7.973019	5.901215	13.803712	3.482554
223	Pd	7.911808	11.824806	15.755872	2.943931
224	Pd	7.974612	13.797353	13.809536	3.481210
225	Pd	9.948438	-0.013429	13.819456	3.497088
226	Pd	9.953961	7.876887	13.828000	3.469680
227	Pd	9.947633	3.929110	13.818752	3.488733
228	Pd	9.954829	11.820609	13.849696	3.483937
229	Pd	9.921754	5.883952	15.785536	2.904934
230	Pd	9.929028	13.757793	15.773792	2.887875
231	Pd	9.964849	1.992525	15.781376	2.862518
232	Pd	9.997182	9.955176	15.828160	2.958983
233	Pd	11.920622	5.904734	13.815552	3.493107
234	Pd	11.928149	13.800115	13.813280	3.488796
235	Pd	11.917703	1.959702	13.824768	3.497788
236	Pd	11.946975	9.857592	13.837024	3.489355
237	Pd	11.944229	3.956440	15.763296	2.907328
238	Pd	11.993920	11.872762	15.768288	2.939842
239	Pd	11.888115	15.748014	15.765472	2.910870
240	Pd	11.908203	7.838747	15.751616	2.874151
241	Pd	13.896278	-0.002683	13.820864	3.498016
242	Pd	13.896326	7.881337	13.809760	3.491722
243	Pd	13.887000	3.935911	13.818688	3.499487
244	Pd	13.902180	11.835615	13.813376	3.501844
245	Pd	13.918497	5.937493	15.775936	2.881728
246	Pd	13.920595	13.842863	15.782368	2.888452
247	Pd	13.871930	1.935370	15.775328	2.866985
248	Pd	13.878100	9.821724	15.781888	2.857252
249	Sr	2.054919	1.962022	5.891072	1.598027
250	Sr	2.055093	9.852401	5.890912	1.598811
251	Sr	2.055093	5.907259	5.891904	1.597662
252	Sr	2.054950	13.796754	5.891456	1.598092
253	Sr	2.055534	1.961407	9.829696	1.611577
254	Sr	2.055187	9.851628	9.829216	1.611942

Sr	2.055140	5.906580	9.829600	1.612287
Sr	2.055692	13.796896	9.830464	1.611444
Sr	2.054603	1.962495	1.977504	1.567434
Sr	2.054603	5.907606	1.977504	1.567509
Sr	2.054603	9.852495	1.977504	1.567385
Sr	2.054603	13.797606	1.977504	1.567527
Sr	6.000692	5.907795	5.891872	1.597380
Sr	6.000676	13.796659	5.891616	1.597708
Sr	6.000077	1.962164	5.891104	1.598226
Sr	5.999998	9.851896	5.890944	1.598466
Sr	6.000692	5.905302	9.830080	1.611188
Sr	6.001260	13.795854	9.830880	1.611306
Sr	6.001671	1.961091	9.829632	1.611195
Sr	6.002207	9.852780	9.830496	1.611170
Sr	5.999698	1.962495	1.977504	1.567492
Sr	5.999698	5.907606	1.977504	1.567665
Sr	5.999698	9.852495	1.977504	1.567498
Sr	5.999698	13.797606	1.977504	1.567662
Sr	9.944998	1.962054	5.891936	1.597782
Sr	9.945093	9.852622	5.891808	1.598700
Sr	9.944856	5.907464	5.892896	1.596759
Sr	9.944982	13.797117	5.892608	1.596990
Sr	9.945818	1.960807	9.831424	1.611281
Sr	9.946465	9.851517	9.835744	1.612168
Sr	9.946024	5.908079	9.834624	1.611172
Sr	9.946765	13.795775	9.832640	1.611349
Sr	9.944603	1.962495	1.977504	1.567371
Sr	9.944603	5.907606	1.977504	1.567510
Sr	9.944603	9.852495	1.977504	1.567345
Sr	9.944603	13.797606	1.977504	1.567518
Sr	13.889872	5.908000	5.891872	1.597572
Sr	13.889793	13.796912	5.891840	1.597561
Sr	13.890124	1.962164	5.891328	1.598039
Sr	13.890045	9.852069	5.890880	1.598274
Sr	13.889745	5.905728	9.830528	1.610960
Sr	13.890219	13.796312	9.831904	1.610622
Sr	13.889950	1.961706	9.830400	1.611610
Sr	13.889950	9.852985	9.830432	1.611062
Sr	13.889698	1.962495	1.977504	1.567501
Sr	13.889698	5.907606	1.977504	1.567672
Sr	13.889698	9.852495	1.977504	1.567516
Sr	13.889698	13.797606	1.977504	1.567662
Ti	0.082798	3.933686	7.854336	2.605928
Ti	0.083382	11.823702	7.854560	2.606196
Ti	0.083618	7.879301	7.854880	2.605810
Ti	0.083097	-0.011220	7.854432	2.605712
Ti	0.083823	-0.009010	11.748192	2.814735
Ti	0.083460	7.879775	11.747936	2.815288
Ti	0.082782	3.935043	11.748320	2.814416
Ti	0.083776	11.824806	11.747840	2.815200
Ti	0.081993	3.935106	0.004992	2.389528
Ti	0.081993	3.935106	3.950112	2.605052
Ti	0.081993	7.879901	0.004992	2.389726
Ti	0.081993	7.879901	3.950112	2.604660
Ti	0.081993	11.825106	0.004992	2.389631
Ti	0.081993	11.825106	3.950112	2.605042
Ti	0.081993	-0.010099	0.004992	2.389623
Ti	0.081993	-0.010099	3.950112	2.605017
Ti	4.028082	7.879222	7.854624	2.606285
Ti	4.028208	-0.011598	7.854208	2.605948
Ti	4.028555	3.933844	7.854240	2.606019
Ti	4.028413	11.823844	7.854528	2.605747
Ti	4.027450	3.933717	11.747968	2.814748
Ti	4.027940	11.824270	11.747424	2.814713
Ti	4.028129	-0.010367	11.748256	2.814437
Ti	4.025431	7.879222	11.746592	2.815008
Ti	4.027103	3.935106	0.004992	2.389514
Ti	4.027103	3.935106	3.950112	2.604973
Ti	4.027103	7.879901	0.004992	2.389790
Ti	4.027103	7.879901	3.950112	2.605342
Ti	4.027103	11.825106	0.004992	2.389657
Ti	4.027103	11.825106	3.950112	2.605242

```
Ti       4.027103      -0.010099       0.004992        2.389635
Ti       4.027103      -0.010099       3.950112        2.605043
Ti       7.973602       3.933875       7.855168        2.605627
Ti       7.973965      11.823165       7.855360        2.605125
Ti       7.974723       7.880485       7.855616        2.605990
Ti       7.973587      -0.010588       7.855136        2.605808
Ti       7.972861      -0.010336      11.749664        2.815074
Ti       7.971441       7.879254      11.751424        2.814820
Ti       7.972845       3.932755      11.748480        2.817108
Ti       7.976411      11.825548      11.751520        2.810887
Ti       7.971993       3.935106       0.004992        2.389737
Ti       7.971993       3.935106       3.950112        2.604823
Ti       7.971993       7.879901       0.004992        2.390037
Ti       7.971993       7.879901       3.950112        2.605015
Ti       7.971993      11.825106       0.004992        2.389927
Ti       7.971993      11.825106       3.950112        2.604740
Ti       7.971993      -0.010099       0.004992        2.389830
Ti       7.971993      -0.010099       3.950112        2.605218
Ti      11.917245       7.880122       7.855424        2.606174
Ti      11.918145      -0.010446       7.855264        2.605524
Ti      11.918082       3.934364       7.855264        2.605368
Ti      11.918161      11.823796       7.855744        2.605084
Ti      11.917514       3.934206      11.749280        2.815767
Ti      11.916251      11.824348      11.752928        2.808316
Ti      11.918902      -0.009547      11.749920        2.814055
Ti      11.919502       7.880295      11.752736        2.813805
Ti      11.917103       3.935106       0.004992        2.389546
Ti      11.917103       3.935106       3.950112        2.604648
Ti      11.917103       7.879901       0.004992        2.389830
Ti      11.917103       7.879901       3.950112        2.605142
Ti      11.917103      11.825106       0.004992        2.389698
Ti      11.917103      11.825106       3.950112        2.604569
Ti      11.917103      -0.010099       0.004992        2.389673
Ti      11.917103      -0.010099       3.950112        2.605106
```

1.2.9 $CH_2O(OH)$

```
      358
jmolscript: load "" {1 1 1} spacegroup "x,y,z" unitcell [{      15.780000       0.000000       0.000000 }, {       0.000000      15.780000       0.000000 }, {       0.000000       0.000000      32.000000 }]
C        8.163215       9.691145      18.245696        3.948597
H        6.677370      11.111487      18.207360        0.859858
H        7.567220       9.011848      18.880544        0.942628
H        8.813540      10.355641      18.841280        0.945660
O        0.063215       5.904702      11.686272        2.232910
O        0.065329      13.793945      11.690656        2.232605
O        0.084928       7.879443       5.902912        1.937548
O        0.083208      11.825153       5.902752        1.937992
O        0.082924       3.935500       5.901184        1.940740
O        0.081993       1.962495       0.004992        1.691918
O        0.081993       1.962495       3.950112        1.911643
O        0.081993       3.935106       1.977504        1.897346
O        0.081993       5.907606       0.004992        1.692378
O        0.081993       5.907606       3.950112        1.911510
O        0.081993       7.879901       1.977504        1.897325
O        0.081993       9.852495       0.004992        1.692123
O        0.081993       9.852495       3.950112        1.911172
O        0.081993      11.825106       1.977504        1.897230
O        0.081993      13.797606       0.004992        1.692440
O        0.081993      13.797606       3.950112        1.911667
O        0.081993      -0.010099       1.977504        1.897434
O        0.079515      11.821019       9.813216        1.980940
O        0.082514      -0.010967       5.901088        1.940827
O        0.084218      -0.012087       9.811008        1.982597
O        0.086427       7.882173       9.811936        1.981802
O        0.090577       3.931587       9.810432        1.983003
```

O	0.105805	1.958882	11.687776	2.236853
O	0.108330	9.849734	11.692960	2.240840
O	0.236306	5.907385	7.858272	1.904668
O	0.235169	13.795902	7.857504	1.905068
O	2.054603	3.935106	0.004992	1.691913
O	2.054603	3.935106	3.950112	1.911319
O	2.054603	7.879901	0.004992	1.692031
O	2.054603	7.879901	3.950112	1.911130
O	2.054603	11.825106	0.004992	1.692059
O	2.054603	11.825106	3.950112	1.911033
O	2.054603	-0.010099	0.004992	1.691989
O	2.054603	-0.010099	3.950112	1.911290
O	2.056402	11.843584	11.678912	2.247557
O	2.058454	11.673429	7.863648	1.903422
O	2.056386	15.750160	11.684896	2.240198
O	2.056765	0.139953	7.854816	1.904948
O	2.059637	7.854274	11.684224	2.240076
O	2.058170	3.784044	7.854240	1.905430
O	2.060663	8.030994	7.864320	1.904018
O	2.060063	3.952322	11.689088	2.235516
O	3.879197	5.908079	7.858848	1.905302
O	3.878976	13.796628	7.858624	1.904326
O	3.997563	9.851265	11.688896	2.214958
O	4.011181	1.961486	11.691936	2.234415
O	4.041258	11.822013	9.814880	1.981995
O	4.029139	-0.003440	9.808448	1.984252
O	4.027103	1.962495	0.004992	1.691992
O	4.027103	1.962495	3.950112	1.912141
O	4.027103	3.935106	1.977504	1.897273
O	4.027103	5.907606	0.004992	1.692503
O	4.027103	5.907606	3.950112	1.912123
O	4.027103	7.879901	1.977504	1.897230
O	4.027103	9.852495	0.004992	1.692272
O	4.027103	9.852495	3.950112	1.911224
O	4.027103	11.825106	1.977504	1.897243
O	4.027103	13.797606	0.004992	1.692591
O	4.027103	13.797606	3.950112	1.912103
O	4.027103	-0.010099	1.977504	1.897190
O	4.029565	3.931761	9.811552	1.981690
O	4.029107	-0.011346	5.900672	1.939967
O	4.029991	11.823749	5.903456	1.937241
O	4.029881	7.880674	5.903488	1.937899
O	4.031127	3.937710	5.901056	1.939681
O	4.047791	7.878070	9.819072	1.979324
O	4.047365	13.794876	11.669856	2.241817
O	4.055239	5.907653	11.684160	2.237622
O	4.182852	1.963048	7.852320	1.905946
O	4.184509	9.851675	7.860448	1.903789
O	5.999698	3.935106	0.004992	1.692222
O	5.999698	3.935106	3.950112	1.911291
O	5.999698	7.879901	0.004992	1.692415
O	5.999698	7.879901	3.950112	1.911515
O	5.999698	11.825106	0.004992	1.692464
O	5.999698	11.825106	3.950112	1.911299
O	5.999698	-0.010099	0.004992	1.692323
O	5.999698	-0.010099	3.950112	1.911432
O	6.007777	7.907153	11.725856	2.234012
O	6.003327	11.797696	11.709696	2.244773
O	5.999477	0.007290	11.686560	2.244169
O	6.002838	15.616567	7.857088	1.904327
O	6.004732	11.977541	7.861952	1.905469
O	6.003801	4.088645	7.860416	1.903537
O	6.004827	7.726235	7.860192	1.905454
O	6.004180	3.913787	11.688608	2.233830
O	7.181794	10.582762	17.558144	2.560505
O	7.819321	9.851138	7.853824	1.907210
O	7.823598	1.962843	7.852000	1.904617
O	7.964797	5.901499	11.701920	2.206986
O	7.956166	13.799421	11.686080	2.220637
O	7.972419	3.943501	5.903008	1.939174
O	7.971993	1.962495	0.004992	1.692051
O	7.971993	1.962495	3.950112	1.911302

101	O	7.971993	3.935106	1.977504	1.897272
102	O	7.971993	5.907606	0.004992	1.692538
103	O	7.971993	5.907606	3.950112	1.911682
104	O	7.971993	7.879901	1.977504	1.897427
105	O	7.971993	9.852495	0.004992	1.692392
106	O	7.971993	9.852495	3.950112	1.911132
107	O	7.971993	11.825106	1.977504	1.897294
108	O	7.971993	13.797606	0.004992	1.692642
109	O	7.971993	13.797606	3.950112	1.911814
110	O	7.971993	-0.010099	1.977504	1.897310
111	O	7.975512	7.875972	5.905952	1.935672
112	O	7.973776	15.761821	5.902976	1.937686
113	O	7.974502	11.827757	5.905792	1.935413
114	O	7.970036	7.905101	9.834176	1.968700
115	O	7.974092	-0.002320	9.810848	1.985993
116	O	7.977958	3.928747	9.813344	1.984611
117	O	7.976443	11.792189	9.832672	1.968120
118	O	7.992901	1.959403	11.685536	2.244436
119	O	7.995189	9.848756	11.764192	2.235425
120	O	8.126700	13.791830	7.876992	1.902055
121	O	8.127710	5.911519	7.876736	1.901675
122	O	8.879658	8.993227	17.333248	2.548457
123	O	9.947254	7.861280	11.709344	2.244026
124	O	9.944872	11.829082	11.720032	2.235331
125	O	9.944603	3.935106	0.004992	1.692010
126	O	9.944603	3.935106	3.950112	1.910566
127	O	9.944603	7.879901	0.004992	1.692178
128	O	9.944603	7.879901	3.950112	1.910774
129	O	9.944603	11.825106	0.004992	1.692222
130	O	9.944603	11.825106	3.950112	1.910698
131	O	9.944603	-0.010099	0.004992	1.692102
132	O	9.944603	-0.010099	3.950112	1.910777
133	O	9.944682	8.033503	7.865632	1.904781
134	O	9.946339	3.783870	7.853632	1.905005
135	O	9.944824	11.669862	7.863552	1.905093
136	O	9.946276	0.138643	7.855808	1.904495
137	O	9.949243	15.750381	11.686816	2.240034
138	O	9.951547	3.942554	11.689312	2.242835
139	O	11.767872	5.908000	7.860032	1.903684
140	O	11.767209	13.794087	7.861472	1.903818
141	O	11.899761	9.845584	11.704000	2.207229
142	O	11.904432	1.957304	11.686624	2.237104
143	O	11.911659	7.880611	9.818176	1.980130
144	O	11.916961	3.929157	9.808320	1.983475
145	O	11.909166	11.817816	9.820576	1.978531
146	O	11.917103	1.962495	0.004992	1.691924
147	O	11.917103	1.962495	3.950112	1.911940
148	O	11.917103	3.935106	1.977504	1.897253
149	O	11.917103	5.907606	0.004992	1.692422
150	O	11.917103	5.907606	3.950112	1.911508
151	O	11.917103	7.879901	1.977504	1.897246
152	O	11.917103	9.852495	0.004992	1.692172
153	O	11.917103	9.852495	3.950112	1.911137
154	O	11.917103	11.825106	1.977504	1.897303
155	O	11.917103	13.797606	0.004992	1.692498
156	O	11.917103	13.797606	3.950112	1.911581
157	O	11.917103	-0.010099	1.977504	1.897253
158	O	11.915320	7.880153	5.904000	1.937160
159	O	11.918161	-0.013460	5.901312	1.939448
160	O	11.915494	11.823922	5.903936	1.937319
161	O	11.919754	-0.012387	9.810848	1.983171
162	O	11.919281	3.936652	5.901056	1.939534
163	O	11.937065	13.789984	11.684800	2.237032
164	O	11.943803	5.904024	11.676608	2.244800
165	O	12.069727	1.960570	7.854080	1.905348
166	O	12.071448	9.851186	7.859456	1.904775
167	O	13.891071	11.798154	11.692544	2.235282
168	O	13.890929	7.729012	7.861440	1.904412
169	O	13.889698	3.935106	0.004992	1.692026
170	O	13.889698	3.935106	3.950112	1.911384
171	O	13.889698	7.879901	0.004992	1.692139
172	O	13.889698	7.879901	3.950112	1.911255

173	O	13.889698	11.825106	0.004992	1.692160
174	O	13.889698	11.825106	3.950112	1.911278
175	O	13.889698	-0.010099	0.004992	1.692099
176	O	13.889698	-0.010099	3.950112	1.911480
177	O	13.889351	11.974258	7.861600	1.904627
178	O	13.894432	7.900699	11.681152	2.245551
179	O	13.891544	15.617308	7.856768	1.904887
180	O	13.892507	4.085426	7.858112	1.903991
181	O	13.892744	0.002888	11.686880	2.235797
182	O	13.894969	3.908580	11.679296	2.236199
183	O	15.714134	9.851943	7.855040	1.905596
184	O	15.714040	1.961517	7.855968	1.904972
185	Pd	0.087216	3.904856	15.756832	2.933354
186	Pd	0.100377	5.890611	13.817728	3.502601
187	Pd	0.088905	9.847556	13.813216	3.500415
188	Pd	0.094775	11.787439	15.761152	2.898064
189	Pd	0.073361	13.798016	13.823200	3.502253
190	Pd	0.095437	1.954432	13.815840	3.519264
191	Pd	0.090530	7.903965	15.761568	2.899309
192	Pd	0.098152	0.008332	15.756160	2.935475
193	Pd	2.052568	11.825485	13.801536	3.471031
194	Pd	2.061105	7.862701	13.813824	3.479159
195	Pd	2.111348	9.851012	15.747808	2.892668
196	Pd	2.087773	1.959434	15.779488	2.879759
197	Pd	2.028503	13.794829	15.766848	2.910705
198	Pd	2.053941	-0.006202	13.809792	3.512357
199	Pd	2.053215	3.925165	13.817120	3.516510
200	Pd	2.024921	5.895108	15.779616	2.899089
201	Pd	4.002802	7.799896	15.787904	2.981787
202	Pd	4.011876	13.821749	13.793216	3.503206
203	Pd	4.001477	11.904085	15.772128	3.017421
204	Pd	4.019813	15.754610	15.750016	2.954557
205	Pd	4.077647	9.838230	13.839072	3.504808
206	Pd	4.035514	1.966014	13.824544	3.494069
207	Pd	4.026519	3.946310	15.760832	2.932846
208	Pd	4.018203	5.881790	13.812512	3.501100
209	Pd	5.819743	9.857845	15.801120	2.919463
210	Pd	5.982735	11.843884	13.818368	3.481712
211	Pd	5.967302	13.825047	15.778848	2.956453
212	Pd	6.014721	-0.013713	13.805248	3.489921
213	Pd	6.007241	3.940850	13.821792	3.485541
214	Pd	6.000739	5.877687	15.793856	2.953923
215	Pd	5.991887	7.855931	13.852192	3.496446
216	Pd	5.996652	1.950913	15.771008	2.871784
217	Pd	7.986179	7.766742	15.819936	2.984625
218	Pd	7.985264	11.932410	15.769152	2.926600
219	Pd	7.977910	3.900611	15.767840	2.919442
220	Pd	7.980987	13.776098	13.833056	3.514767
221	Pd	7.983623	9.852069	13.886048	3.306403
222	Pd	7.976411	1.958077	13.807680	3.499784
223	Pd	7.973697	-0.000126	15.758528	2.930212
224	Pd	7.973476	5.914612	13.860640	3.510917
225	Pd	9.958916	1.960318	15.768288	2.870107
226	Pd	9.945424	3.932439	13.807776	3.489009
227	Pd	9.974080	13.835068	15.796416	2.941828
228	Pd	9.946907	15.755588	13.812000	3.488809
229	Pd	9.967721	11.852326	13.844000	3.501141
230	Pd	9.979524	7.843796	13.821600	3.439735
231	Pd	10.111240	9.840219	15.827264	3.010140
232	Pd	9.999991	5.847484	15.770656	2.851751
233	Pd	11.928307	15.761869	15.756864	2.957819
234	Pd	11.929522	1.946605	13.817056	3.495592
235	Pd	11.955149	5.863848	13.792800	3.514465
236	Pd	11.942036	13.817631	13.814336	3.501396
237	Pd	11.968751	7.805167	15.760224	2.908479
238	Pd	11.947906	3.925259	15.752384	2.957523
239	Pd	11.892928	9.867360	13.861888	3.500292
240	Pd	11.944466	11.876912	15.791424	2.974242
241	Pd	13.925345	5.893451	15.768672	2.898293
242	Pd	13.905636	7.867671	13.805664	3.473858
243	Pd	13.912169	3.908753	13.811008	3.510788
244	Pd	13.901864	-0.005902	13.815936	3.517941

245	Pd	13.876948	1.947836	15.780896	2.886587
246	Pd	13.920769	13.806190	15.781856	2.874917
247	Pd	13.895063	11.827725	13.827872	3.482816
248	Pd	13.869784	9.842191	15.765408	2.882300
249	Sr	2.054603	1.962495	1.977504	1.567301
250	Sr	2.054603	5.907606	1.977504	1.567442
251	Sr	2.054603	9.852495	1.977504	1.567298
252	Sr	2.054603	13.797606	1.977504	1.567431
253	Sr	2.055014	9.849860	5.892992	1.596529
254	Sr	2.057538	13.793456	9.828032	1.612684
255	Sr	2.058895	9.848393	9.832096	1.613598
256	Sr	2.056134	5.905081	5.891808	1.597630
257	Sr	2.056607	1.960270	5.890816	1.598598
258	Sr	2.055960	13.794939	5.891680	1.597749
259	Sr	2.059858	5.903708	9.829472	1.611520
260	Sr	2.059038	1.959860	9.827328	1.612451
261	Sr	5.999698	1.962495	1.977504	1.567393
262	Sr	5.999698	5.907606	1.977504	1.567615
263	Sr	5.999698	9.852495	1.977504	1.567357
264	Sr	5.999698	13.797606	1.977504	1.567570
265	Sr	5.999714	13.794560	5.892096	1.597632
266	Sr	6.001860	1.959355	9.828032	1.612652
267	Sr	5.999319	5.905586	5.892128	1.598108
268	Sr	5.999414	1.959560	5.890784	1.598900
269	Sr	5.999935	9.849781	5.893056	1.598411
270	Sr	6.004337	13.791310	9.835424	1.609603
271	Sr	6.003753	9.847730	9.841152	1.608159
272	Sr	6.005679	5.907196	9.836160	1.608892
273	Sr	9.944603	1.962495	1.977504	1.567266
274	Sr	9.944603	5.907606	1.977504	1.567469
275	Sr	9.944603	9.852495	1.977504	1.567296
276	Sr	9.944603	13.797606	1.977504	1.567440
277	Sr	9.946655	9.848440	9.844896	1.608618
278	Sr	9.948264	5.907653	9.840672	1.609351
279	Sr	9.945014	9.849939	5.894240	1.597111
280	Sr	9.946434	1.959639	5.892032	1.597988
281	Sr	9.944509	13.794450	5.893760	1.596705
282	Sr	9.945014	5.905444	5.893888	1.596894
283	Sr	9.947602	13.788343	9.837952	1.608886
284	Sr	9.948327	1.958235	9.828416	1.613336
285	Sr	13.891765	9.848535	9.830880	1.612079
286	Sr	13.892460	5.904355	9.829344	1.613017
287	Sr	13.889698	1.962495	1.977504	1.567430
288	Sr	13.889698	5.907606	1.977504	1.567574
289	Sr	13.889698	9.852495	1.977504	1.567377
290	Sr	13.889698	13.797606	1.977504	1.567585
291	Sr	13.889761	13.794813	5.891712	1.597675
292	Sr	13.889398	5.905649	5.892128	1.597363
293	Sr	13.890014	1.960255	5.890528	1.598361
294	Sr	13.891244	13.792903	9.829152	1.611481
295	Sr	13.889966	9.849923	5.892640	1.597112
296	Sr	13.892633	1.956468	9.825824	1.613163
297	Ti	0.081993	3.935106	0.004992	2.390157
298	Ti	0.081993	3.935106	3.950112	2.605454
299	Ti	0.081993	7.879901	0.004992	2.390373
300	Ti	0.081993	7.879901	3.950112	2.604988
301	Ti	0.081993	11.825106	0.004992	2.390326
302	Ti	0.081993	11.825106	3.950112	2.604773
303	Ti	0.081993	-0.010099	0.004992	2.390320
304	Ti	0.081993	-0.010099	3.950112	2.605639
305	Ti	0.082940	11.819520	7.855904	2.605520
306	Ti	0.083713	3.930277	7.852480	2.606596
307	Ti	0.084360	-0.015243	7.852192	2.606112
308	Ti	0.083161	11.822423	11.749408	2.814089
309	Ti	0.088289	7.875293	11.748640	2.813876
310	Ti	0.083776	7.875151	7.856416	2.605828
311	Ti	0.085307	-0.013981	11.744160	2.816529
312	Ti	0.086853	3.930766	11.743072	2.815212
313	Ti	4.023348	11.821050	11.749664	2.812948
314	Ti	4.027103	3.935106	0.004992	2.390227
315	Ti	4.027103	3.935106	3.950112	2.605561
316	Ti	4.027103	7.879901	0.004992	2.390520

```
Ti       4.027103      7.879901      3.950112        2.604809
Ti       4.027103     11.825106      0.004992        2.390474
Ti       4.027103     11.825106      3.950112        2.604611
Ti       4.027103     -0.010099      0.004992        2.390449
Ti       4.027103     -0.010099      3.950112        2.606149
Ti       4.029265     -0.015622      7.854080        2.607156
Ti       4.029833     -0.009863     11.744256        2.817079
Ti       4.030780     11.819110      7.856864        2.605094
Ti       4.029865      3.930325      7.854368        2.606620
Ti       4.028397      7.878654     11.753632        2.809134
Ti       4.030275      7.874977      7.856640        2.605687
Ti       4.032532      3.932423     11.747392        2.815499
Ti       7.971993      3.935106      0.004992        2.390325
Ti       7.971993      3.935106      3.950112        2.604267
Ti       7.971993      7.879901      0.004992        2.390638
Ti       7.971993      7.879901      3.950112        2.603946
Ti       7.971993     11.825106      0.004992        2.390631
Ti       7.971993     11.825106      3.950112        2.603515
Ti       7.971993     -0.010099      0.004992        2.390534
Ti       7.971993     -0.010099      3.950112        2.605130
Ti       7.975196      3.931003     11.743264        2.812748
Ti       7.976664     -0.013492     11.744416        2.815636
Ti       7.973097     11.817105      7.858848        2.603717
Ti       7.973634      3.930340      7.853952        2.605249
Ti       7.974013     -0.015717      7.855776        2.606428
Ti       7.973634      7.876934      7.858528        2.603535
Ti       7.974802      7.876808     11.768800        2.806383
Ti       7.977611     11.818825     11.767936        2.806914
Ti      11.927676      7.880485     11.754016        2.810501
Ti      11.917103      3.935106      0.004992        2.390141
Ti      11.917103      3.935106      3.950112        2.605451
Ti      11.917103      7.879901      0.004992        2.390400
Ti      11.917103      7.879901      3.950112        2.604359
Ti      11.917103     11.825106      0.004992        2.390336
Ti      11.917103     11.825106      3.950112        2.604205
Ti      11.917103     -0.010099      0.004992        2.390339
Ti      11.917103     -0.010099      3.950112        2.605880
Ti      11.916456      7.876035      7.857568        2.605409
Ti      11.917940     -0.015512      7.854144        2.605962
Ti      11.917955      3.930782      7.854432        2.606695
Ti      11.917009     11.819125      7.857056        2.604996
Ti      11.925435     11.819488     11.755808        2.807448
Ti      11.920859     -0.014502     11.745696        2.815640
Ti      11.924678      3.926001     11.743808        2.817275
```

1.2.10 CH(OH)(OH)

```
      358
jmolscript: load "" {1 1 1} spacegroup "x,y,z" unitcell [{     15.780000
       0.000000      0.000000 }, {      0.000000    15.780000      0.000000
    }, {      0.000000      0.000000    32.000000 }]
C        9.973796      9.483054     17.833216        4.028141
H        9.460489     10.381867     18.213696        0.953446
H        9.755117      7.568735     17.850976        0.938346
H       11.826084     10.048199     17.765152        0.981394
O        0.062315     13.797385     11.691776        2.238763
O        0.062631      5.907211     11.693728        2.236309
O        0.081141     11.825469      5.902592        1.939570
O        0.081993      1.962495      0.004992        1.690765
O        0.081993      1.962495      3.950112        1.911872
O        0.081993      3.935106      1.977504        1.897484
O        0.081993      5.907606      0.004992        1.691143
O        0.081993      5.907606      3.950112        1.911777
O        0.081993      7.879901      1.977504        1.897562
O        0.081993      9.852495      0.004992        1.690781
O        0.081993      9.852495      3.950112        1.911700
O        0.081993     11.825106      1.977504        1.897478
```

19	O	0.081993	13.797606	0.004992	1.691191
20	O	0.081993	13.797606	3.950112	1.911950
21	O	0.081993	-0.010099	1.977504	1.897565
22	O	0.082577	7.881195	5.903392	1.939091
23	O	0.079310	-0.010588	9.814368	1.981102
24	O	0.081835	3.935516	5.902944	1.939619
25	O	0.082861	-0.008758	5.903008	1.939404
26	O	0.075618	7.881069	9.815872	1.980642
27	O	0.081804	11.825406	9.813920	1.981665
28	O	0.084234	3.937284	9.814560	1.981187
29	O	0.099509	1.961438	11.690624	2.235788
30	O	0.099019	9.852969	11.694112	2.239531
31	O	0.235043	5.908742	7.858912	1.904577
32	O	0.235106	13.798348	7.858336	1.904702
33	O	2.054603	3.935106	0.004992	1.690680
34	O	2.054603	3.935106	3.950112	1.911369
35	O	2.054603	7.879901	0.004992	1.690702
36	O	2.054603	7.879901	3.950112	1.911217
37	O	2.054603	11.825106	0.004992	1.690725
38	O	2.054603	11.825106	3.950112	1.911468
39	O	2.054603	-0.010099	0.004992	1.690727
40	O	2.054603	-0.010099	3.950112	1.911289
41	O	2.054225	11.672971	7.857248	1.904593
42	O	2.054083	8.033456	7.862912	1.904351
43	O	2.055140	0.143330	7.860512	1.905015
44	O	2.055171	3.783839	7.857664	1.904864
45	O	2.052804	11.843553	11.687456	2.243126
46	O	2.053625	15.750839	11.688160	2.234962
47	O	2.052173	7.859339	11.687648	2.233700
48	O	2.053735	3.951107	11.691136	2.240268
49	O	3.874921	5.908584	7.859808	1.904424
50	O	3.875410	13.798363	7.860096	1.904082
51	O	4.003686	9.852574	11.694048	2.240209
52	O	4.006747	1.961643	11.691904	2.237469
53	O	4.023569	11.822849	9.813216	1.982007
54	O	4.025178	3.935280	9.814656	1.981207
55	O	4.027103	1.962495	0.004992	1.690720
56	O	4.027103	1.962495	3.950112	1.911562
57	O	4.027103	3.935106	1.977504	1.897474
58	O	4.027103	5.907606	0.004992	1.691123
59	O	4.027103	5.907606	3.950112	1.911912
60	O	4.027103	7.879901	1.977504	1.897417
61	O	4.027103	9.852495	0.004992	1.690741
62	O	4.027103	9.852495	3.950112	1.911392
63	O	4.027103	11.825106	1.977504	1.897462
64	O	4.027103	13.797606	0.004992	1.691170
65	O	4.027103	13.797606	3.950112	1.912075
66	O	4.027103	-0.010099	1.977504	1.897408
67	O	4.026677	-0.009784	5.902816	1.939768
68	O	4.025936	7.880248	5.903200	1.939037
69	O	4.027845	11.825990	5.902624	1.939360
70	O	4.028192	3.936447	5.902848	1.939650
71	O	4.030086	-0.007874	9.813952	1.981582
72	O	4.030354	7.881258	9.815808	1.981075
73	O	4.043751	13.797353	11.688864	2.238075
74	O	4.043704	5.906249	11.694080	2.236222
75	O	4.180453	1.963616	7.858176	1.904837
76	O	4.180138	9.852780	7.858112	1.904473
77	O	5.996021	7.898774	11.699808	2.234841
78	O	5.999698	3.935106	0.004992	1.690864
79	O	5.999698	3.935106	3.950112	1.911439
80	O	5.999698	7.879901	0.004992	1.690897
81	O	5.999698	7.879901	3.950112	1.911465
82	O	5.999698	11.825106	0.004992	1.690922
83	O	5.999698	11.825106	3.950112	1.911482
84	O	5.999698	-0.010099	0.004992	1.690921
85	O	5.999698	-0.010099	3.950112	1.911422
86	O	5.999651	7.728129	7.858944	1.904947
87	O	5.999777	11.978077	7.860416	1.904346
88	O	6.000140	4.088677	7.860896	1.904267
89	O	6.000455	15.618602	7.857792	1.905128
90	O	5.997583	11.805286	11.685504	2.235757

O	5.998704	0.005775	11.692608	2.238651
O	5.998104	3.914639	11.690368	2.234862
O	7.818911	9.852306	7.860224	1.904463
O	7.820489	1.963458	7.861728	1.903871
O	7.954887	13.795239	11.689472	2.240349
O	7.954887	5.906549	11.699232	2.234088
O	7.968427	7.878149	9.818976	1.978574
O	7.971661	3.935674	5.903104	1.939248
O	7.971819	11.824932	5.903136	1.939062
O	7.971993	1.962495	0.004992	1.690792
O	7.971993	1.962495	3.950112	1.911746
O	7.971993	3.935106	1.977504	1.897489
O	7.971993	5.907606	0.004992	1.691169
O	7.971993	5.907606	3.950112	1.911851
O	7.971993	7.879901	1.977504	1.897568
O	7.971993	9.852495	0.004992	1.690817
O	7.971993	9.852495	3.950112	1.911699
O	7.971993	11.825106	1.977504	1.897481
O	7.971993	13.797606	0.004992	1.691227
O	7.971993	13.797606	3.950112	1.911871
O	7.971993	-0.010099	1.977504	1.897535
O	7.973019	7.880232	5.903616	1.938378
O	7.973145	-0.009105	5.902976	1.939304
O	7.971693	-0.011661	9.814560	1.981338
O	7.974597	3.939477	9.815200	1.981084
O	7.974202	11.822613	9.815040	1.981479
O	7.987110	1.961896	11.687648	2.237148
O	7.994385	9.850981	11.697760	2.240344
O	8.125185	13.797464	7.859232	1.903866
O	8.125406	5.908553	7.859168	1.905008
O	9.248200	8.367171	18.154656	2.318875
O	9.943767	8.033503	7.862272	1.905032
O	9.941495	7.867734	11.702176	2.235827
O	9.944603	3.935106	0.004992	1.690719
O	9.944603	3.935106	3.950112	1.911311
O	9.944603	7.879901	0.004992	1.690746
O	9.944603	7.879901	3.950112	1.911374
O	9.944603	11.825106	0.004992	1.690781
O	9.944603	11.825106	3.950112	1.911209
O	9.944603	-0.010099	0.004992	1.690777
O	9.944603	-0.010099	3.950112	1.911158
O	9.944572	11.672340	7.858176	1.904481
O	9.945077	0.142904	7.859904	1.904858
O	9.944856	3.782718	7.857536	1.904768
O	9.943609	11.835316	11.691936	2.243208
O	9.943262	15.753663	11.694688	2.234390
O	9.943562	3.950491	11.691776	2.246991
O	11.296492	9.341571	18.216864	2.297025
O	11.764132	5.908016	7.860768	1.904363
O	11.764716	13.797306	7.861664	1.903158
O	11.893260	9.849718	11.697664	2.240458
O	11.898720	1.961091	11.690016	2.237828
O	11.914452	7.880311	9.817824	1.978895
O	11.914263	11.819346	9.815456	1.981667
O	11.915415	7.880059	5.903616	1.938228
O	11.916914	-0.010620	5.902912	1.939676
O	11.917103	1.962495	0.004992	1.690750
O	11.917103	1.962495	3.950112	1.911530
O	11.917103	3.935106	1.977504	1.897458
O	11.917103	5.907606	0.004992	1.691147
O	11.917103	5.907606	3.950112	1.912121
O	11.917103	7.879901	1.977504	1.897414
O	11.917103	9.852495	0.004992	1.690769
O	11.917103	9.852495	3.950112	1.911731
O	11.917103	11.825106	1.977504	1.897486
O	11.917103	13.797606	0.004992	1.691204
O	11.917103	13.797606	3.950112	1.912143
O	11.917103	-0.010099	1.977504	1.897427
O	11.917356	11.825469	5.903136	1.939191
O	11.917908	3.935942	5.902592	1.939264
O	11.914894	3.933433	9.813504	1.981965
O	11.917577	-0.009279	9.814944	1.981131

163	O	11.930516	13.794150	11.688704	2.237259
164	O	11.933515	5.903629	11.690560	2.236949
165	O	12.070327	1.962511	7.857920	1.904427
166	O	12.070580	9.852227	7.858240	1.904662
167	O	13.888404	7.727434	7.859520	1.904865
168	O	13.889698	3.935106	0.004992	1.690862
169	O	13.889698	3.935106	3.950112	1.911344
170	O	13.889698	7.879901	0.004992	1.690887
171	O	13.889698	7.879901	3.950112	1.911504
172	O	13.889698	11.825106	0.004992	1.690914
173	O	13.889698	11.825106	3.950112	1.911446
174	O	13.889698	-0.010099	0.004992	1.690918
175	O	13.889698	-0.010099	3.950112	1.911259
176	O	13.885721	7.899184	11.695808	2.238013
177	O	13.889067	11.978630	7.860032	1.904635
178	O	13.889808	15.617908	7.858208	1.905094
179	O	13.889966	4.088756	7.860448	1.904398
180	O	13.887662	11.807606	11.688864	2.236518
181	O	13.887978	0.005365	11.692768	2.237718
182	O	13.889856	3.914403	11.689568	2.236825
183	O	15.708785	9.853079	7.859584	1.904333
184	O	15.710363	1.963553	7.860032	1.904310
185	Pd	0.036199	7.876950	15.767872	2.907434
186	Pd	0.034116	15.754042	15.769888	2.898595
187	Pd	0.077354	13.799010	13.818496	3.497626
188	Pd	0.080210	5.902525	13.821312	3.501481
189	Pd	0.090041	9.848093	13.815488	3.496558
190	Pd	0.080005	1.961722	13.820416	3.495941
191	Pd	0.123526	3.940219	15.765760	2.914773
192	Pd	0.135235	11.835205	15.763040	2.909699
193	Pd	2.041411	1.916623	15.770656	2.873421
194	Pd	2.050658	11.830708	13.811488	3.496650
195	Pd	2.049364	-0.010131	13.819520	3.496459
196	Pd	2.051526	9.800800	15.773984	2.866952
197	Pd	2.050090	3.936005	13.816576	3.499622
198	Pd	2.056734	7.871080	13.820128	3.493995
199	Pd	2.061562	13.845987	15.774496	2.883322
200	Pd	2.059874	5.950638	15.779488	2.859774
201	Pd	3.966934	11.825532	15.762144	2.921261
202	Pd	3.976292	3.921440	15.765216	2.915016
203	Pd	4.021359	13.802798	13.815936	3.500598
204	Pd	4.021880	1.962748	13.820320	3.498432
205	Pd	4.026756	9.844858	13.814176	3.490991
206	Pd	4.029391	5.902825	13.821952	3.495612
207	Pd	4.066538	-0.003266	15.767936	2.907056
208	Pd	4.070625	7.883262	15.767456	2.910735
209	Pd	5.984234	11.830408	13.816736	3.494130
210	Pd	5.983192	9.916988	15.775648	2.853496
211	Pd	5.983650	13.749745	15.778464	2.868731
212	Pd	5.988952	-0.004892	13.818944	3.506619
213	Pd	5.981204	5.843602	15.783648	2.868248
214	Pd	5.993575	3.931335	13.820800	3.499911
215	Pd	6.003233	2.007263	15.779424	2.868567
216	Pd	6.002775	7.871364	13.832608	3.503505
217	Pd	7.913354	7.869691	15.779264	2.875387
218	Pd	7.922018	15.759912	15.765600	2.909617
219	Pd	7.946382	9.858760	13.815552	3.454184
220	Pd	7.963409	1.954179	13.818272	3.492907
221	Pd	7.959795	13.814995	13.815968	3.493259
222	Pd	7.962698	5.895566	13.829472	3.493800
223	Pd	8.006046	11.840207	15.752352	2.935902
224	Pd	8.012484	3.932313	15.767456	2.925340
225	Pd	9.934346	1.917144	15.773920	2.864384
226	Pd	9.938923	3.920352	13.807104	3.484479
227	Pd	9.918172	9.831287	15.822944	3.353938
228	Pd	9.931537	11.845099	13.810976	3.454478
229	Pd	9.943688	-0.011314	13.821920	3.501415
230	Pd	9.938733	7.851418	13.827104	3.450248
231	Pd	9.955618	13.847250	15.774528	2.872269
232	Pd	9.952967	5.913176	15.776960	2.957048
233	Pd	11.867807	11.807527	15.759168	3.011294
234	Pd	11.876265	3.921803	15.760704	2.919361

235	Pd	11.918192	5.914644	13.819552	3.489243
236	Pd	11.912543	9.858981	13.821280	3.444608
237	Pd	11.922500	1.959103	13.817376	3.495414
238	Pd	11.921680	13.816431	13.816928	3.478743
239	Pd	11.953350	7.862180	15.762592	2.903026
240	Pd	11.965643	0.002967	15.765024	2.911758
241	Pd	13.890298	5.857788	15.775168	2.871998
242	Pd	13.869500	13.739977	15.775776	2.863338
243	Pd	13.888483	3.935848	13.816928	3.497558
244	Pd	13.895505	-0.009673	13.818624	3.507384
245	Pd	13.890676	11.815938	13.817120	3.480743
246	Pd	13.900192	2.010404	15.776928	2.872093
247	Pd	13.902164	7.878686	13.821888	3.489566
248	Pd	13.906157	9.910724	15.770016	2.894212
249	Sr	2.054603	1.962495	1.977504	1.567518
250	Sr	2.054603	5.907606	1.977504	1.567650
251	Sr	2.054603	9.852495	1.977504	1.567514
252	Sr	2.054603	13.797606	1.977504	1.567632
253	Sr	2.052710	1.960207	5.891040	1.597924
254	Sr	2.053136	13.794592	5.891328	1.597733
255	Sr	2.053309	5.904971	5.891520	1.597781
256	Sr	2.053120	9.850444	5.891456	1.597614
257	Sr	2.052015	13.795381	9.829792	1.611936
258	Sr	2.052520	1.960917	9.829312	1.611992
259	Sr	2.052694	5.905002	9.829952	1.611275
260	Sr	2.051495	9.851123	9.831232	1.611345
261	Sr	5.999698	1.962495	1.977504	1.567604
262	Sr	5.999698	5.907606	1.977504	1.567748
263	Sr	5.999698	9.852495	1.977504	1.567566
264	Sr	5.999698	13.797606	1.977504	1.567753
265	Sr	5.997899	13.795050	5.891296	1.598039
266	Sr	5.998499	1.960255	5.891104	1.597671
267	Sr	5.997110	1.959529	9.829568	1.611964
268	Sr	5.999019	9.850097	5.891840	1.597918
269	Sr	5.996716	13.796107	9.829536	1.611541
270	Sr	5.998704	5.905712	5.891680	1.597877
271	Sr	5.997852	9.848566	9.832480	1.610032
272	Sr	5.998799	5.906785	9.832192	1.610363
273	Sr	9.944603	1.962495	1.977504	1.567500
274	Sr	9.944603	5.907606	1.977504	1.567622
275	Sr	9.944603	9.852495	1.977504	1.567494
276	Sr	9.944603	13.797606	1.977504	1.567647
277	Sr	9.943562	13.794971	5.891424	1.597660
278	Sr	9.943451	1.961138	5.891328	1.597975
279	Sr	9.943751	5.905523	5.891648	1.597740
280	Sr	9.943499	9.849750	5.891808	1.597585
281	Sr	9.942047	9.849939	9.836640	1.611731
282	Sr	9.942505	5.906643	9.833984	1.609767
283	Sr	9.942189	13.794040	9.831328	1.611014
284	Sr	9.942915	1.961359	9.830272	1.611392
285	Sr	13.889698	1.962495	1.977504	1.567575
286	Sr	13.889698	5.907606	1.977504	1.567732
287	Sr	13.889698	9.852495	1.977504	1.567599
288	Sr	13.889698	13.797606	1.977504	1.567746
289	Sr	13.887994	9.850192	5.891168	1.597900
290	Sr	13.888041	13.795160	5.891488	1.598012
291	Sr	13.888530	1.960697	5.891584	1.597786
292	Sr	13.887489	5.905712	5.891904	1.597772
293	Sr	13.886148	9.849766	9.832416	1.610447
294	Sr	13.887363	13.795807	9.830624	1.611375
295	Sr	13.887520	1.959750	9.830144	1.611403
296	Sr	13.886400	5.907369	9.832096	1.610793
297	Ti	0.081993	3.935106	0.004992	2.388618
298	Ti	0.081993	3.935106	3.950112	2.604737
299	Ti	0.081993	7.879901	0.004992	2.388717
300	Ti	0.081993	7.879901	3.950112	2.604622
301	Ti	0.081993	11.825106	0.004992	2.388682
302	Ti	0.081993	11.825106	3.950112	2.604964
303	Ti	0.081993	-0.010099	0.004992	2.388758
304	Ti	0.081993	-0.010099	3.950112	2.604796
305	Ti	0.079231	11.825990	11.748800	2.816625
306	Ti	0.078158	11.820104	7.854880	2.605850

```
Ti       0.078221       3.930766       7.854720        2.605922
Ti       0.078569      -0.015386       7.854880        2.606239
Ti       0.082293      -0.009784      11.749504        2.814359
Ti       0.079768       7.875214       7.855136        2.606308
Ti       0.081267       3.933528      11.748672        2.815507
Ti       0.081914       7.878102      11.750080        2.814356
Ti       4.027103       3.935106       0.004992        2.388523
Ti       4.027103       3.935106       3.950112        2.604820
Ti       4.027103       7.879901       0.004992        2.388658
Ti       4.027103       7.879901       3.950112        2.604691
Ti       4.027103      11.825106       0.004992        2.388590
Ti       4.027103      11.825106       3.950112        2.604881
Ti       4.027103      -0.010099       0.004992        2.388691
Ti       4.027103      -0.010099       3.950112        2.604889
Ti       4.023884      11.825216      11.747872        2.816044
Ti       4.024452      -0.009563      11.748416        2.815599
Ti       4.025241       3.933765      11.748576        2.814914
Ti       4.023064      -0.014802       7.854208        2.606008
Ti       4.024437      11.819110       7.854912        2.605946
Ti       4.024326       3.929914       7.854400        2.606250
Ti       4.024878       7.875198       7.855008        2.605690
Ti       4.025241       7.877881      11.749856        2.813991
Ti       7.971993       3.935106       0.004992        2.388649
Ti       7.971993       3.935106       3.950112        2.604666
Ti       7.971993       7.879901       0.004992        2.388758
Ti       7.971993       7.879901       3.950112        2.604611
Ti       7.971993      11.825106       0.004992        2.388726
Ti       7.971993      11.825106       3.950112        2.604693
Ti       7.971993      -0.010099       0.004992        2.388798
Ti       7.971993      -0.010099       3.950112        2.604759
Ti       7.967054      11.826400      11.749152        2.815353
Ti       7.968695       3.932770      11.749376        2.814966
Ti       7.970257      -0.009216      11.748512        2.815114
Ti       7.969405       7.877597      11.754976        2.812551
Ti       7.968963      11.819741       7.855136        2.605129
Ti       7.969121      -0.015212       7.854624        2.605615
Ti       7.968742       3.931035       7.855136        2.605747
Ti       7.970604       7.875088       7.856096        2.605489
Ti      11.917103       3.935106       0.004992        2.388562
Ti      11.917103       3.935106       3.950112        2.604917
Ti      11.917103       7.879901       0.004992        2.388688
Ti      11.917103       7.879901       3.950112        2.604578
Ti      11.917103      11.825106       0.004992        2.388624
Ti      11.917103      11.825106       3.950112        2.604722
Ti      11.917103      -0.010099       0.004992        2.388732
Ti      11.917103      -0.010099       3.950112        2.604939
Ti      11.913095      -0.013587       7.854368        2.605615
Ti      11.913790      11.819914       7.855168        2.605478
Ti      11.914137       7.876445       7.856736        2.605178
Ti      11.913774       3.931350       7.855328        2.605813
Ti      11.916456      -0.009279      11.748608        2.815057
Ti      11.915936       7.881211      11.754624        2.812881
Ti      11.914610      11.825800      11.749472        2.814565
Ti      11.916804       3.935074      11.748576        2.816768
```

1.2.11 COH

```
       355
jmolscript: load "" {1 1 1} spacegroup "x,y,z" unitcell [{      15.780000
       0.000000      0.000000 }, {       0.000000     15.780000      0.000000
    }, {       0.000000      0.000000     32.000000 }]
C        7.972214       9.865482      16.615360        3.945883
H        7.944662      10.752713      18.328384        0.848493
O       15.713030       1.966314       7.858112        1.904945
O       15.711515       9.856393       7.859936        1.905142
O        0.064461       5.914881      11.691840        2.239348
O        0.064461      13.803334      11.692672        2.238899
```

O	0.099067	1.968965	11.693088	2.241912
O	0.098578	9.859360	11.695424	2.244788
O	0.081740	3.937852	5.903008	1.938893
O	0.081851	11.827362	5.903328	1.938724
O	0.082577	7.881873	5.903264	1.938623
O	0.082498	-0.008553	5.902944	1.939056
O	0.081441	-0.003614	9.814336	1.981505
O	0.081030	7.886513	9.816832	1.979496
O	0.081993	1.962495	0.004992	1.690985
O	0.081993	1.962495	3.950112	1.911662
O	0.081993	3.935106	1.977504	1.897383
O	0.081993	5.907606	0.004992	1.691370
O	0.081993	5.907606	3.950112	1.911937
O	0.081993	7.879901	1.977504	1.897503
O	0.081993	9.852495	0.004992	1.691038
O	0.081993	9.852495	3.950112	1.911626
O	0.081993	11.825106	1.977504	1.897416
O	0.081993	13.797606	0.004992	1.691436
O	0.081993	13.797606	3.950112	1.911988
O	0.081993	-0.010099	1.977504	1.897461
O	0.082624	3.938578	9.814048	1.981528
O	0.082340	11.829035	9.817152	1.979398
O	0.232692	5.911535	7.860640	1.905059
O	0.232660	13.801046	7.860608	1.904848
O	2.053530	15.758823	11.690592	2.239403
O	2.053372	3.959802	11.690848	2.239754
O	2.055692	7.875735	11.694752	2.236709
O	2.055534	11.843789	11.696256	2.236880
O	2.054603	3.935106	0.004992	1.690904
O	2.054603	3.935106	3.950112	1.911346
O	2.054603	7.879901	0.004992	1.690966
O	2.054603	7.879901	3.950112	1.911305
O	2.054603	11.825106	0.004992	1.690996
O	2.054603	11.825106	3.950112	1.911354
O	2.054603	-0.010099	0.004992	1.690975
O	2.054603	-0.010099	3.950112	1.911227
O	2.055140	3.789535	7.858592	1.904753
O	2.053972	11.678888	7.858880	1.905115
O	2.055077	0.143440	7.860192	1.904524
O	2.054130	8.034292	7.860224	1.904696
O	3.877982	5.911898	7.858880	1.905714
O	3.878030	13.800904	7.858784	1.905851
O	4.006937	1.969107	11.688640	2.237321
O	4.023553	9.859281	11.700544	2.228086
O	4.026930	3.944511	9.815584	1.980346
O	4.022133	11.830376	9.815872	1.979291
O	4.041858	5.914723	11.698016	2.238370
O	4.041952	13.803508	11.698688	2.238822
O	4.028224	-0.009247	9.815776	1.980324
O	4.024657	7.885329	9.815904	1.979044
O	4.027103	1.962495	0.004992	1.690965
O	4.027103	1.962495	3.950112	1.911589
O	4.027103	3.935106	1.977504	1.897373
O	4.027103	5.907606	0.004992	1.691375
O	4.027103	5.907606	3.950112	1.911884
O	4.027103	7.879901	1.977504	1.897296
O	4.027103	9.852495	0.004992	1.691041
O	4.027103	9.852495	3.950112	1.911512
O	4.027103	11.825106	1.977504	1.897360
O	4.027103	13.797606	0.004992	1.691470
O	4.027103	13.797606	3.950112	1.911898
O	4.027103	-0.010099	1.977504	1.897314
O	4.026188	7.882725	5.902944	1.938764
O	4.026961	-0.007480	5.903328	1.938590
O	4.027750	3.936621	5.903328	1.938574
O	4.026756	11.826810	5.903040	1.938893
O	4.176445	1.966314	7.862464	1.905020
O	4.176272	9.856425	7.859680	1.906152
O	5.998720	3.923634	11.693120	2.239270
O	6.002554	11.821129	11.680704	2.243379
O	5.998925	0.015528	11.693984	2.239313
O	6.002554	7.898474	11.683392	2.240836

O	5.999698	3.935106	0.004992	1.691110
O	5.999698	3.935106	3.950112	1.911418
O	5.999698	7.879901	0.004992	1.691188
O	5.999698	7.879901	3.950112	1.911204
O	5.999698	11.825106	0.004992	1.691245
O	5.999698	11.825106	3.950112	1.911253
O	5.999698	-0.010099	0.004992	1.691205
O	5.999698	-0.010099	3.950112	1.911365
O	5.999682	4.088061	7.859648	1.904569
O	5.999004	11.977983	7.858912	1.903950
O	5.999288	7.735356	7.856928	1.904342
O	5.999730	15.625356	7.858144	1.904629
O	7.823045	1.966614	7.860992	1.904771
O	7.824276	9.856519	7.865216	1.902339
O	7.954019	5.913208	11.697408	2.228164
O	7.954288	13.805401	11.695136	2.230318
O	7.988625	1.969581	11.686592	2.242956
O	7.988593	9.859486	11.641728	2.260732
O	7.971677	3.934080	5.902208	1.940885
O	7.971519	11.824412	5.902112	1.941049
O	7.972577	7.884808	5.902016	1.941146
O	7.972593	-0.004482	5.902112	1.940906
O	7.971220	-0.006217	9.814336	1.981720
O	7.971362	7.868255	9.808256	1.987654
O	7.971993	1.962495	0.004992	1.691040
O	7.971993	1.962495	3.950112	1.911467
O	7.971993	3.935106	1.977504	1.897418
O	7.971993	5.907606	0.004992	1.691428
O	7.971993	5.907606	3.950112	1.911364
O	7.971993	7.879901	1.977504	1.897460
O	7.971993	9.852495	0.004992	1.691128
O	7.971993	9.852495	3.950112	1.911230
O	7.971993	11.825106	1.977504	1.897363
O	7.971993	13.797606	0.004992	1.691548
O	7.971993	13.797606	3.950112	1.911293
O	7.971993	-0.010099	1.977504	1.897477
O	7.972324	3.942633	9.814592	1.981215
O	7.972671	11.847214	9.807680	1.988428
O	7.973003	9.834348	17.979808	2.365189
O	8.122187	5.909768	7.851040	1.905633
O	8.122250	13.803160	7.851136	1.905430
O	9.944304	15.761537	11.692960	2.237715
O	9.944288	3.957624	11.693472	2.238542
O	9.940769	7.864310	11.682496	2.240672
O	9.940737	11.854804	11.681216	2.242439
O	9.944603	3.935106	0.004992	1.690942
O	9.944603	3.935106	3.950112	1.911633
O	9.944603	7.879901	0.004992	1.691022
O	9.944603	7.879901	3.950112	1.911495
O	9.944603	11.825106	0.004992	1.691079
O	9.944603	11.825106	3.950112	1.911600
O	9.944603	-0.010099	0.004992	1.691038
O	9.944603	-0.010099	3.950112	1.911581
O	9.944856	3.789236	7.858784	1.904850
O	9.945582	11.679977	7.857504	1.904366
O	9.944919	0.144150	7.860256	1.904594
O	9.945377	8.033535	7.858944	1.904209
O	11.767698	5.911709	7.858400	1.906103
O	11.767746	13.801062	7.858368	1.905969
O	11.901560	1.969107	11.688768	2.236798
O	11.885685	9.859155	11.701024	2.226719
O	11.915746	3.944621	9.815456	1.980240
O	11.920496	11.830282	9.815680	1.979854
O	11.935298	5.914675	11.698048	2.238580
O	11.935440	13.803508	11.698688	2.238463
O	11.917151	-0.009326	9.815744	1.980329
O	11.920938	7.885203	9.815776	1.979457
O	11.917103	1.962495	0.004992	1.690958
O	11.917103	1.962495	3.950112	1.911616
O	11.917103	3.935106	1.977504	1.897369
O	11.917103	5.907606	0.004992	1.691370
O	11.917103	5.907606	3.950112	1.912130

O	11.917103	7.879901	1.977504	1.897299
O	11.917103	9.852495	0.004992	1.691035
O	11.917103	9.852495	3.950112	1.911828
O	11.917103	11.825106	1.977504	1.897350
O	11.917103	13.797606	0.004992	1.691464
O	11.917103	13.797606	3.950112	1.912166
O	11.917103	-0.010099	1.977504	1.897312
O	11.917277	7.882804	5.902816	1.939071
O	11.916693	-0.007211	5.903360	1.938779
O	11.917450	3.936447	5.903296	1.938872
O	11.918240	11.826715	5.902880	1.939114
O	12.067297	1.966346	7.862560	1.904977
O	12.067865	9.856456	7.859872	1.905420
O	13.889840	3.925496	11.689984	2.239371
O	13.887473	11.809973	11.695488	2.237109
O	13.889872	0.013255	11.691264	2.239236
O	13.887410	7.909520	11.695712	2.237377
O	13.889698	3.935106	0.004992	1.691067
O	13.889698	3.935106	3.950112	1.911430
O	13.889698	7.879901	0.004992	1.691131
O	13.889698	7.879901	3.950112	1.911450
O	13.889698	11.825106	0.004992	1.691160
O	13.889698	11.825106	3.950112	1.911496
O	13.889698	-0.010099	0.004992	1.691139
O	13.889698	-0.010099	3.950112	1.911343
O	13.889524	4.089135	7.860256	1.904214
O	13.890282	11.979592	7.859936	1.904822
O	13.890108	7.733557	7.858208	1.904993
O	13.889556	15.623983	7.859008	1.904571
Pd	0.080604	0.013381	15.756224	2.920763
Pd	0.079358	7.900588	15.757920	2.920018
Pd	0.080478	1.972484	13.815712	3.502306
Pd	0.079626	9.862847	13.816608	3.490454
Pd	0.079910	3.933165	15.755328	2.919575
Pd	0.078095	5.919520	13.817152	3.500453
Pd	0.079247	11.824617	15.759072	2.918720
Pd	0.078348	13.805638	13.818496	3.499448
Pd	2.048307	-0.001894	13.817664	3.502869
Pd	2.054982	7.885455	13.820800	3.490866
Pd	2.048733	3.947809	13.817216	3.503473
Pd	2.055424	11.840744	13.822368	3.490344
Pd	2.038871	5.920798	15.780832	2.855969
Pd	2.039391	13.805827	15.782016	2.855756
Pd	2.060568	1.973257	15.783968	2.863040
Pd	2.059700	9.862879	15.780384	2.859504
Pd	4.025036	5.911251	13.820064	3.505524
Pd	4.024594	13.815958	13.820832	3.505850
Pd	4.007583	1.972689	13.818304	3.499454
Pd	4.022543	9.862532	13.836416	3.477132
Pd	4.013690	3.956204	15.760672	2.913998
Pd	4.033368	11.843979	15.764864	2.905914
Pd	4.015363	-0.010257	15.761472	2.913830
Pd	4.032279	7.882378	15.764864	2.887379
Pd	5.991398	0.008442	13.817248	3.497574
Pd	5.981946	7.873273	13.810656	3.460697
Pd	5.992644	3.939619	13.816608	3.496182
Pd	5.978853	11.854378	13.805952	3.457606
Pd	6.023652	5.904687	15.769472	2.861659
Pd	6.025372	13.818483	15.769408	2.899876
Pd	5.968043	1.973715	15.776512	2.871369
Pd	6.046691	9.867250	15.788800	3.176549
Pd	7.969799	0.038014	15.765792	2.926254
Pd	7.970888	7.930618	15.815200	3.131536
Pd	7.970857	1.973305	13.812064	3.495240
Pd	7.972545	9.859107	13.738816	3.364475
Pd	7.969673	3.907065	15.767104	2.919695
Pd	7.969405	5.892741	13.837312	3.473599
Pd	7.972024	11.804040	15.795488	3.263006
Pd	7.969721	13.836598	13.831392	3.470868
Pd	9.948106	0.007259	13.816416	3.496950
Pd	9.958143	7.869660	13.810528	3.461327
Pd	9.946465	3.941528	13.816704	3.497292

Pd	9.963713	11.858465	13.806368	3.459208
Pd	9.915726	5.904450	15.769888	2.863355
Pd	9.915521	13.819572	15.769440	2.899553
Pd	9.971571	1.973494	15.776768	2.870500
Pd	9.897390	9.866335	15.788768	3.174732
Pd	11.917182	5.910983	13.820096	3.506638
Pd	11.918902	13.817520	13.820864	3.507571
Pd	11.930674	1.973699	13.818944	3.499259
Pd	11.914973	9.862484	13.835872	3.477518
Pd	11.925987	3.955983	15.760768	2.914813
Pd	11.907478	11.845825	15.765344	2.904665
Pd	11.924425	-0.009326	15.761600	2.915725
Pd	11.907399	7.880169	15.765312	2.888434
Pd	13.891071	0.001215	13.817984	3.503466
Pd	13.883134	7.887144	13.821184	3.492271
Pd	13.890471	3.945931	13.816768	3.502161
Pd	13.883307	11.839055	13.821728	3.490096
Pd	13.901281	5.920151	15.781856	2.855642
Pd	13.901154	13.806853	15.782880	2.855213
Pd	13.879236	1.972989	15.784672	2.862211
Pd	13.878037	9.862847	15.781312	2.858345
Sr	2.054051	1.964547	5.891424	1.596975
Sr	2.054398	9.854752	5.891616	1.597138
Sr	2.054067	5.909815	5.891808	1.597015
Sr	2.054177	13.799121	5.891776	1.596982
Sr	2.053909	1.968334	9.830400	1.610940
Sr	2.055361	9.858508	9.832896	1.609361
Sr	2.054777	5.915543	9.832160	1.610073
Sr	2.054808	13.801898	9.832288	1.610052
Sr	2.054603	1.962495	1.977504	1.567427
Sr	2.054603	5.907606	1.977504	1.567545
Sr	2.054603	9.852495	1.977504	1.567413
Sr	2.054603	13.797606	1.977504	1.567543
Sr	5.998436	5.909342	5.891680	1.597726
Sr	5.998420	13.799941	5.891840	1.597589
Sr	5.999019	1.964310	5.891552	1.597099
Sr	5.998499	9.854515	5.891680	1.596787
Sr	5.997804	5.912924	9.829632	1.612491
Sr	5.997852	13.804218	9.830048	1.612800
Sr	5.998467	1.968555	9.829792	1.610952
Sr	5.998609	9.858729	9.829920	1.613597
Sr	5.999698	1.962495	1.977504	1.567529
Sr	5.999698	5.907606	1.977504	1.567655
Sr	5.999698	9.852495	1.977504	1.567529
Sr	5.999698	13.797606	1.977504	1.567646
Sr	9.945108	1.964531	5.890912	1.597505
Sr	9.946150	9.854705	5.890720	1.597557
Sr	9.945976	5.909310	5.890848	1.598203
Sr	9.946087	13.799736	5.890944	1.598271
Sr	9.945377	1.968492	9.829280	1.610305
Sr	9.944950	9.858429	9.827904	1.613084
Sr	9.944666	5.912608	9.828320	1.611812
Sr	9.944840	13.804912	9.828576	1.611370
Sr	9.944603	1.962495	1.977504	1.567466
Sr	9.944603	5.907606	1.977504	1.567568
Sr	9.944603	9.852495	1.977504	1.567469
Sr	9.944603	13.797606	1.977504	1.567556
Sr	13.890029	5.909784	5.891712	1.597388
Sr	13.890045	13.799405	5.891744	1.597325
Sr	13.890045	1.964452	5.891648	1.596715
Sr	13.889824	9.854468	5.891648	1.597108
Sr	13.888814	5.914881	9.832000	1.609796
Sr	13.888862	13.802229	9.832128	1.609806
Sr	13.890014	1.968602	9.830848	1.611290
Sr	13.888799	9.858839	9.832480	1.610069
Sr	13.889698	1.962495	1.977504	1.567506
Sr	13.889698	5.907606	1.977504	1.567657
Sr	13.889698	9.852495	1.977504	1.567497
Sr	13.889698	13.797606	1.977504	1.567654
Ti	0.082324	3.939603	7.855648	2.606201
Ti	0.082040	11.829051	7.855936	2.605936
Ti	0.081882	7.884619	7.855840	2.606488

```
Ti       0.082245      -0.006107       7.855552        2.606787
Ti       0.081267      -0.003551      11.749504        2.816563
Ti       0.080983       7.887728      11.752320        2.814994
Ti       0.081267       3.942428      11.749440        2.816968
Ti       0.081188      11.831497      11.752736        2.814771
Ti       0.081993       3.935106       0.004992        2.388820
Ti       0.081993       3.935106       3.950112        2.604698
Ti       0.081993       7.879901       0.004992        2.388964
Ti       0.081993       7.879901       3.950112        2.604708
Ti       0.081993      11.825106       0.004992        2.388921
Ti       0.081993      11.825106       3.950112        2.604604
Ti       0.081993      -0.010099       0.004992        2.388981
Ti       0.081993      -0.010099       3.950112        2.604771
Ti       4.026472       7.884540       7.855648        2.606166
Ti       4.026677      -0.005965       7.855712        2.605508
Ti       4.026551       3.939524       7.855648        2.606695
Ti       4.026441      11.829225       7.855712        2.607355
Ti       4.024799       3.941197      11.751360        2.815886
Ti       4.027671      11.830424      11.752384        2.810589
Ti       4.025036      -0.002036      11.751584        2.816413
Ti       4.028255       7.888990      11.752640        2.810465
Ti       4.027103       3.935106       0.004992        2.388750
Ti       4.027103       3.935106       3.950112        2.604560
Ti       4.027103       7.879901       0.004992        2.388948
Ti       4.027103       7.879901       3.950112        2.604579
Ti       4.027103      11.825106       0.004992        2.388902
Ti       4.027103      11.825106       3.950112        2.604558
Ti       4.027103      -0.010099       0.004992        2.388961
Ti       4.027103      -0.010099       3.950112        2.604632
Ti       7.972151       3.939177       7.852672        2.606579
Ti       7.972166      11.830156       7.852096        2.607325
Ti       7.972009       7.883136       7.851680        2.607242
Ti       7.972214      -0.005965       7.852768        2.607037
Ti       7.971204      -0.001957      11.746432        2.813815
Ti       7.971314       7.879348      11.738080        2.820667
Ti       7.971125       3.942144      11.746752        2.812811
Ti       7.971677      11.838866      11.737600        2.821990
Ti       7.971993       3.935106       0.004992        2.388886
Ti       7.971993       3.935106       3.950112        2.605105
Ti       7.971993       7.879901       0.004992        2.389064
Ti       7.971993       7.879901       3.950112        2.604768
Ti       7.971993      11.825106       0.004992        2.389076
Ti       7.971993      11.825106       3.950112        2.605020
Ti       7.971993      -0.010099       0.004992        2.389089
Ti       7.971993      -0.010099       3.950112        2.604812
Ti      11.917608       7.884256       7.855488        2.605862
Ti      11.917782      -0.005949       7.855648        2.605739
Ti      11.917608       3.939682       7.855552        2.607251
Ti      11.917624      11.829493       7.855392        2.606419
Ti      11.917166       3.941307      11.751072        2.815378
Ti      11.914815      11.830597      11.751744        2.811634
Ti      11.917308      -0.001815      11.751360        2.816085
Ti      11.914042       7.888864      11.752064        2.810524
Ti      11.917103       3.935106       0.004992        2.388742
Ti      11.917103       3.935106       3.950112        2.604570
Ti      11.917103       7.879901       0.004992        2.388943
Ti      11.917103       7.879901       3.950112        2.604908
Ti      11.917103      11.825106       0.004992        2.388896
Ti      11.917103      11.825106       3.950112        2.604887
Ti      11.917103      -0.010099       0.004992        2.388954
Ti      11.917103      -0.010099       3.950112        2.604654
```

1.2.12 CHOH

```
      356
jmolscript: load "" {1 1 1} spacegroup "x,y,z" unitcell [{      15.780000
       0.000000      0.000000 }, {      0.000000    15.780000      0.000000
```

```
     }, {      0.000000     0.000000    32.000000 }]
C       8.970820    10.829814    17.319520       3.958759
H       7.609432     9.516839    17.615936       1.003259
H       9.631591    11.481354    17.914016       0.971248
O      15.705866     1.965494     7.857408       1.904537
O      15.704319     9.855304     7.858496       1.904265
O       0.057976     5.912166    11.690848       2.236987
O       0.058149    13.802229    11.687296       2.239069
O       0.097536     1.967798    11.689472       2.238180
O       0.098972     9.856504    11.693024       2.239816
O       0.080178     3.937615     5.902176       1.940431
O       0.081930    11.826353     5.902752       1.939426
O       0.079799     7.881573     5.902432       1.940375
O       0.081346    -0.008837     5.902112       1.940317
O       0.083224    -0.005176     9.812256       1.982738
O       0.075113     7.884524     9.814464       1.980821
O       0.081993     1.962495     0.004992       1.690630
O       0.081993     1.962495     3.950112       1.911847
O       0.081993     3.935106     1.977504       1.897476
O       0.081993     5.907606     0.004992       1.691012
O       0.081993     5.907606     3.950112       1.912007
O       0.081993     7.879901     1.977504       1.897582
O       0.081993     9.852495     0.004992       1.690634
O       0.081993     9.852495     3.950112       1.911709
O       0.081993    11.825106     1.977504       1.897467
O       0.081993    13.797606     0.004992       1.691036
O       0.081993    13.797606     3.950112       1.912064
O       0.081993    -0.010099     1.977504       1.897546
O       0.079926     3.938940     9.812704       1.982001
O       0.078158    11.826810     9.813888       1.981144
O       0.234459     5.910683     7.858752       1.904799
O       0.235564    13.799910     7.858624       1.904451
O       2.052568    15.755210    11.690880       2.235295
O       2.050390     3.956677    11.689504       2.237645
O       2.049080     7.865099    11.686624       2.238829
O       2.052000    11.850464    11.688448       2.240419
O       2.054603     3.935106     0.004992       1.690548
O       2.054603     3.935106     3.950112       1.911536
O       2.054603     7.879901     0.004992       1.690566
O       2.054603     7.879901     3.950112       1.911454
O       2.054603    11.825106     0.004992       1.690531
O       2.054603    11.825106     3.950112       1.911540
O       2.054603    -0.010099     0.004992       1.690577
O       2.054603    -0.010099     3.950112       1.911545
O       2.052757     3.783413     7.856896       1.904774
O       2.053515    11.672703     7.860704       1.903535
O       2.053641     0.147369     7.857056       1.904728
O       2.051211     8.037780     7.857760       1.904037
O       3.870818     5.910004     7.857728       1.904252
O       3.870345    13.799878     7.858816       1.904698
O       4.006921     1.965809    11.689952       2.235229
O       4.002487     9.857798    11.687360       2.245682
O       4.022685     3.936258     9.812448       1.982429
O       4.028918    11.830108     9.815872       1.980810
O       4.038781     5.910778    11.685088       2.239658
O       4.044351    13.800936    11.694112       2.237531
O       4.026472    -0.007653     9.814528       1.980139
O       4.021028     7.884603     9.810880       1.983729
O       4.027103     1.962495     0.004992       1.690572
O       4.027103     1.962495     3.950112       1.911879
O       4.027103     3.935106     1.977504       1.897477
O       4.027103     5.907606     0.004992       1.690977
O       4.027103     5.907606     3.950112       1.911977
O       4.027103     7.879901     1.977504       1.897388
O       4.027103     9.852495     0.004992       1.690561
O       4.027103     9.852495     3.950112       1.911616
O       4.027103    11.825106     1.977504       1.897485
O       4.027103    13.797606     0.004992       1.690990
O       4.027103    13.797606     3.950112       1.912227
O       4.027103    -0.010099     1.977504       1.897420
O       4.026015     7.881731     5.902240       1.939563
O       4.027151    -0.008963     5.902144       1.939979
```

74	O	4.026882	3.936163	5.901984	1.940582
75	O	4.026015	11.826984	5.902816	1.940677
76	O	4.180595	1.964689	7.857248	1.904955
77	O	4.180453	9.855951	7.858016	1.904088
78	O	5.995958	3.924486	11.686880	2.238125
79	O	6.000503	11.809042	11.701600	2.231554
80	O	5.996873	0.009594	11.694752	2.238017
81	O	5.993607	7.899058	11.681792	2.242108
82	O	5.999698	3.935106	0.004992	1.690712
83	O	5.999698	3.935106	3.950112	1.911646
84	O	5.999698	7.879901	0.004992	1.690725
85	O	5.999698	7.879901	3.950112	1.911827
86	O	5.999698	11.825106	0.004992	1.690693
87	O	5.999698	11.825106	3.950112	1.911285
88	O	5.999698	-0.010099	0.004992	1.690739
89	O	5.999698	-0.010099	3.950112	1.911709
90	O	5.998167	4.092085	7.859456	1.904130
91	O	5.998104	11.983348	7.859072	1.904121
92	O	5.997978	7.728444	7.860032	1.904252
93	O	5.998530	15.617214	7.858720	1.904217
94	O	8.211865	10.101046	18.161824	2.363183
95	O	7.815613	1.964531	7.859904	1.904085
96	O	7.814635	9.856172	7.859008	1.904763
97	O	7.955392	5.913318	11.687008	2.243239
98	O	7.951526	13.798379	11.699808	2.229068
99	O	7.982423	1.966220	11.689056	2.241144
100	O	7.983575	9.858871	11.692704	2.241368
101	O	7.970778	3.936431	5.902720	1.939471
102	O	7.971188	11.827220	5.903136	1.940194
103	O	7.970588	7.881589	5.903008	1.939103
104	O	7.970730	-0.008600	5.902752	1.940921
105	O	7.968064	-0.009531	9.815808	1.981267
106	O	7.973397	7.886718	9.815072	1.981041
107	O	7.971993	1.962495	0.004992	1.690608
108	O	7.971993	1.962495	3.950112	1.911704
109	O	7.971993	3.935106	1.977504	1.897464
110	O	7.971993	5.907606	0.004992	1.690985
111	O	7.971993	5.907606	3.950112	1.912059
112	O	7.971993	7.879901	1.977504	1.897535
113	O	7.971993	9.852495	0.004992	1.690597
114	O	7.971993	9.852495	3.950112	1.911629
115	O	7.971993	11.825106	1.977504	1.897469
116	O	7.971993	13.797606	0.004992	1.691008
117	O	7.971993	13.797606	3.950112	1.911986
118	O	7.971993	-0.010099	1.977504	1.897574
119	O	7.972182	3.938325	9.813664	1.981315
120	O	7.967843	11.828215	9.816704	1.981706
121	O	8.125043	5.910809	7.859872	1.904440
122	O	8.126037	13.799910	7.859584	1.904007
123	O	9.940437	-0.017137	11.688960	2.242953
124	O	9.941510	3.953221	11.692672	2.239948
125	O	9.937029	7.877976	11.701056	2.234568
126	O	9.939759	11.852532	11.690432	2.241503
127	O	9.944603	3.935106	0.004992	1.690559
128	O	9.944603	3.935106	3.950112	1.911522
129	O	9.944603	7.879901	0.004992	1.690576
130	O	9.944603	7.879901	3.950112	1.911473
131	O	9.944603	11.825106	0.004992	1.690553
132	O	9.944603	11.825106	3.950112	1.911571
133	O	9.944603	-0.010099	0.004992	1.690596
134	O	9.944603	-0.010099	3.950112	1.911393
135	O	9.942852	3.783381	7.856640	1.904678
136	O	9.942221	11.672860	7.860704	1.904005
137	O	9.942157	0.148237	7.858304	1.904256
138	O	9.942457	8.038869	7.858112	1.904603
139	O	11.759698	5.911472	7.858400	1.904708
140	O	11.759856	13.799941	7.858848	1.904403
141	O	11.899288	1.968429	11.683680	2.240389
142	O	11.883918	9.859249	11.697760	2.232439
143	O	11.912780	3.943611	9.813728	1.981447
144	O	11.914673	11.827252	9.815552	1.980273
145	O	11.925924	5.914944	11.695104	2.232741

O	11.933672	13.803760	11.688576	2.240443
O	11.912653	-0.006833	9.811552	1.983458
O	11.909987	7.885487	9.817088	1.978343
O	11.917103	1.962495	0.004992	1.690590
O	11.917103	1.962495	3.950112	1.912006
O	11.917103	3.935106	1.977504	1.897469
O	11.917103	5.907606	0.004992	1.690992
O	11.917103	5.907606	3.950112	1.912092
O	11.917103	7.879901	1.977504	1.897432
O	11.917103	9.852495	0.004992	1.690593
O	11.917103	9.852495	3.950112	1.911993
O	11.917103	11.825106	1.977504	1.897439
O	11.917103	13.797606	0.004992	1.691028
O	11.917103	13.797606	3.950112	1.912247
O	11.917103	-0.010099	1.977504	1.897380
O	11.915809	7.882347	5.902720	1.939697
O	11.916078	-0.008174	5.902336	1.939735
O	11.916693	3.937299	5.902208	1.940404
O	11.915178	11.826495	5.902912	1.939139
O	12.070075	1.966172	7.858656	1.904217
O	12.070690	9.855683	7.858688	1.904865
O	13.886226	3.925275	11.690080	2.236559
O	13.885611	11.807164	11.690144	2.242564
O	13.887726	0.014265	11.683136	2.238502
O	13.882360	7.906443	11.694784	2.233016
O	13.889698	3.935106	0.004992	1.690724
O	13.889698	3.935106	3.950112	1.911680
O	13.889698	7.879901	0.004992	1.690746
O	13.889698	7.879901	3.950112	1.911639
O	13.889698	11.825106	0.004992	1.690727
O	13.889698	11.825106	3.950112	1.911485
O	13.889698	-0.010099	0.004992	1.690760
O	13.889698	-0.010099	3.950112	1.911677
O	13.887378	4.092669	7.859552	1.904579
O	13.887410	11.983206	7.858304	1.904655
O	13.886211	7.727971	7.859360	1.904528
O	13.888120	15.618145	7.858656	1.903873
Pd	0.068611	-0.000252	15.760288	2.923450
Pd	0.082593	7.875861	15.760000	2.924910
Pd	0.083918	1.971995	13.817568	3.501946
Pd	0.076438	9.855004	13.815744	3.496736
Pd	0.079831	3.944101	15.755360	2.915045
Pd	0.071515	5.911882	13.819136	3.506014
Pd	0.078695	11.839119	15.757792	2.912490
Pd	0.077212	13.814380	13.811968	3.502609
Pd	2.054745	-0.001562	13.822656	3.501196
Pd	2.039344	7.869707	13.813472	3.506579
Pd	2.046903	3.937899	13.818688	3.501828
Pd	2.038776	11.843868	13.814176	3.497720
Pd	2.054856	5.913523	15.784928	2.870352
Pd	2.041443	13.815090	15.777664	2.868863
Pd	2.052868	1.958266	15.780032	2.856685
Pd	2.044993	9.852922	15.772512	2.862921
Pd	4.011670	5.906075	13.811520	3.504195
Pd	4.025036	13.808668	13.823168	3.494928
Pd	4.014716	1.965225	13.821440	3.502158
Pd	3.993792	9.855336	13.801536	3.493304
Pd	4.011907	3.946310	15.760864	2.923247
Pd	3.999930	11.833280	15.763776	2.899654
Pd	4.033731	-0.011393	15.766304	2.891170
Pd	4.011513	7.881715	15.755232	2.931521
Pd	5.989962	-0.004308	13.821952	3.494323
Pd	5.983744	7.879569	13.801952	3.483851
Pd	5.978095	3.936510	13.812576	3.505073
Pd	5.968801	11.825059	13.832896	3.451501
Pd	5.988747	5.909847	15.769280	2.879555
Pd	6.021064	13.766109	15.769568	2.877435
Pd	5.982719	1.982820	15.778016	2.873675
Pd	5.962284	9.841039	15.739328	2.946593
Pd	7.952189	0.016695	15.765856	2.910439
Pd	7.945893	7.859387	15.743424	2.972190
Pd	7.955361	1.975088	13.815616	3.495357

Pd	7.970841	9.845868	13.817408	3.420749
Pd	7.964276	3.939051	15.758176	2.911363
Pd	7.960000	5.891321	13.807552	3.489927
Pd	8.007088	11.817516	15.848384	3.303937
Pd	7.975891	13.831675	13.834784	3.459942
Pd	9.944998	0.013855	13.804928	3.499418
Pd	9.951294	7.859718	13.829568	3.459801
Pd	9.943751	3.940108	13.813856	3.499700
Pd	9.954355	11.838156	13.816832	3.429953
Pd	9.942678	5.895329	15.778272	2.854719
Pd	9.955318	13.854777	15.743840	2.867912
Pd	9.943846	1.962906	15.768032	2.866731
Pd	9.929360	9.874682	15.835904	3.335562
Pd	11.920733	5.907622	13.826752	3.500218
Pd	11.925546	13.817647	13.808096	3.496576
Pd	11.928970	1.979569	13.809984	3.501712
Pd	11.927408	9.851975	13.829344	3.464090
Pd	11.917640	3.929015	15.760000	2.921882
Pd	11.934935	11.841091	15.745696	2.890478
Pd	11.932284	0.016711	15.757472	2.932366
Pd	11.894838	7.898726	15.773248	2.906794
Pd	13.895694	0.009231	13.810464	3.504696
Pd	13.886984	7.879191	13.825888	3.495243
Pd	13.889272	3.947193	13.818848	3.505130
Pd	13.892396	11.833106	13.811360	3.497231
Pd	13.877737	5.916885	15.787648	2.842406
Pd	13.885011	13.809504	15.772576	2.878871
Pd	13.893690	1.972216	15.784032	2.874736
Pd	13.903521	9.853916	15.777568	2.863566
Sr	2.053704	1.963679	5.890304	1.598640
Sr	2.053278	9.853632	5.890752	1.598422
Sr	2.053420	5.908679	5.890656	1.598544
Sr	2.053483	13.798931	5.890560	1.598667
Sr	2.052205	1.965999	9.827936	1.612561
Sr	2.049775	9.855793	9.830368	1.612216
Sr	2.050785	5.911156	9.829024	1.613148
Sr	2.051984	13.801630	9.828736	1.612100
Sr	2.054603	1.962495	1.977504	1.567491
Sr	2.054603	5.907606	1.977504	1.567625
Sr	2.054603	9.852495	1.977504	1.567516
Sr	2.054603	13.797606	1.977504	1.567639
Sr	5.998814	5.909263	5.890912	1.598325
Sr	5.998294	13.799121	5.890528	1.599058
Sr	5.998341	1.964010	5.890464	1.598693
Sr	5.998609	9.852417	5.890912	1.598663
Sr	5.997126	5.911314	9.830208	1.611879
Sr	5.997489	13.800446	9.830688	1.611380
Sr	5.995753	1.965793	9.829184	1.612163
Sr	5.998199	9.856677	9.831968	1.611427
Sr	5.999698	1.962495	1.977504	1.567603
Sr	5.999698	5.907606	1.977504	1.567734
Sr	5.999698	9.852495	1.977504	1.567591
Sr	5.999698	13.797606	1.977504	1.567782
Sr	9.944209	1.964073	5.890560	1.598571
Sr	9.944272	9.853553	5.890880	1.598445
Sr	9.943467	5.908789	5.891008	1.598411
Sr	9.944114	13.798206	5.890688	1.598748
Sr	9.941921	1.966961	9.829632	1.611923
Sr	9.940059	9.856819	9.834624	1.613460
Sr	9.941542	5.913097	9.831904	1.610978
Sr	9.941510	13.799799	9.832160	1.611465
Sr	9.944603	1.962495	1.977504	1.567505
Sr	9.944603	5.907606	1.977504	1.567615
Sr	9.944603	9.852495	1.977504	1.567509
Sr	9.944603	13.797606	1.977504	1.567647
Sr	13.888230	5.909026	5.890624	1.598593
Sr	13.888246	13.798995	5.890944	1.598490
Sr	13.888309	1.963616	5.890112	1.598499
Sr	13.887962	9.853442	5.890784	1.598596
Sr	13.885280	5.912229	9.829952	1.611894
Sr	13.885800	13.800588	9.830304	1.611975
Sr	13.887583	1.967608	9.828704	1.613040

290	Sr	13.884522	9.856156	9.831296	1.611042
291	Sr	13.889698	1.962495	1.977504	1.567594
292	Sr	13.889698	5.907606	1.977504	1.567756
293	Sr	13.889698	9.852495	1.977504	1.567582
294	Sr	13.889698	13.797606	1.977504	1.567729
295	Ti	0.079878	3.937694	7.853600	2.606875
296	Ti	0.078947	11.827189	7.854464	2.605807
297	Ti	0.079799	7.882457	7.853792	2.607049
298	Ti	0.080226	-0.007716	7.853824	2.606443
299	Ti	0.081519	-0.003787	11.746688	2.815448
300	Ti	0.076265	7.884067	11.748448	2.813470
301	Ti	0.078774	3.939698	11.747136	2.815557
302	Ti	0.077669	11.830708	11.748864	2.815265
303	Ti	0.081993	3.935106	0.004992	2.388466
304	Ti	0.081993	3.935106	3.950112	2.604982
305	Ti	0.081993	7.879901	0.004992	2.388562
306	Ti	0.081993	7.879901	3.950112	2.604849
307	Ti	0.081993	11.825106	0.004992	2.388461
308	Ti	0.081993	11.825106	3.950112	2.604726
309	Ti	0.081993	-0.010099	0.004992	2.388584
310	Ti	0.081993	-0.010099	3.950112	2.605220
311	Ti	4.025368	7.882220	7.854912	2.606718
312	Ti	4.025352	-0.008285	7.854240	2.605770
313	Ti	4.025131	3.937110	7.853472	2.606618
314	Ti	4.025083	11.826621	7.853056	2.605860
315	Ti	4.021186	3.937457	11.746752	2.814885
316	Ti	4.022417	11.831860	11.747552	2.812856
317	Ti	4.023427	-0.005413	11.750048	2.814349
318	Ti	4.021265	7.881652	11.746272	2.817178
319	Ti	4.027103	3.935106	0.004992	2.388357
320	Ti	4.027103	3.935106	3.950112	2.605205
321	Ti	4.027103	7.879901	0.004992	2.388478
322	Ti	4.027103	7.879901	3.950112	2.605072
323	Ti	4.027103	11.825106	0.004992	2.388321
324	Ti	4.027103	11.825106	3.950112	2.604615
325	Ti	4.027103	-0.010099	0.004992	2.388494
326	Ti	4.027103	-0.010099	3.950112	2.605284
327	Ti	7.970368	3.937236	7.854496	2.606174
328	Ti	7.969736	11.826621	7.853472	2.606212
329	Ti	7.970794	7.882378	7.855488	2.605992
330	Ti	7.970399	-0.008237	7.852672	2.606237
331	Ti	7.967101	-0.004892	11.746976	2.812726
332	Ti	7.969705	7.886765	11.750752	2.813263
333	Ti	7.967006	3.938609	11.748672	2.816416
334	Ti	7.966880	11.831323	11.749024	2.816906
335	Ti	7.971993	3.935106	0.004992	2.388436
336	Ti	7.971993	3.935106	3.950112	2.604733
337	Ti	7.971993	7.879901	0.004992	2.388526
338	Ti	7.971993	7.879901	3.950112	2.604698
339	Ti	7.971993	11.825106	0.004992	2.388414
340	Ti	7.971993	11.825106	3.950112	2.604457
341	Ti	7.971993	-0.010099	0.004992	2.388544
342	Ti	7.971993	-0.010099	3.950112	2.604713
343	Ti	11.914437	7.882236	7.855008	2.606410
344	Ti	11.914689	-0.008411	7.854752	2.606378
345	Ti	11.915068	3.937268	7.853888	2.606829
346	Ti	11.914579	11.826510	7.855488	2.606086
347	Ti	11.914468	3.941402	11.748160	2.814005
348	Ti	11.910160	11.827504	11.751424	2.811291
349	Ti	11.917608	-0.002446	11.746560	2.816576
350	Ti	11.912227	7.885629	11.752928	2.810024
351	Ti	11.917103	3.935106	0.004992	2.388372
352	Ti	11.917103	3.935106	3.950112	2.604974
353	Ti	11.917103	7.879901	0.004992	2.388497
354	Ti	11.917103	7.879901	3.950112	2.604841
355	Ti	11.917103	11.825106	0.004992	2.388378
356	Ti	11.917103	11.825106	3.950112	2.604622
357	Ti	11.917103	-0.010099	0.004992	2.388529
358	Ti	11.917103	-0.010099	3.950112	2.605044

1.2.13 CO_2

```
      355
jmolscript: load "" {1 1 1} spacegroup "x,y,z" unitcell [{      15.780000
       0.000000      0.000000 }, {       0.000000     15.780000      0.000000
    }, {      0.000000      0.000000     32.000000 }]
C        7.816481       7.745266      18.903136        4.160677
O        0.062741       5.908600      11.692512        2.236702
O        0.062726      13.798600      11.693184        2.236979
O        0.081993       1.962495       0.004992        1.690444
O        0.081993       1.962495       3.950112        1.911596
O        0.081993       3.935106       1.977504        1.897231
O        0.081993       5.907606       0.004992        1.690831
O        0.081993       5.907606       3.950112        1.911661
O        0.081993       7.879901       1.977504        1.897323
O        0.081993       9.852495       0.004992        1.690437
O        0.081993       9.852495       3.950112        1.911492
O        0.081993      11.825106       1.977504        1.897225
O        0.081993      13.797606       0.004992        1.690836
O        0.081993      13.797606       3.950112        1.911731
O        0.081993      -0.010099       1.977504        1.897311
O        0.081504       3.936510       9.815104        1.981352
O        0.081804      11.827220       9.815200        1.981411
O        0.082056       3.935516       5.903104        1.939154
O        0.081946      11.825926       5.903104        1.939113
O        0.081898       7.881053       9.815264        1.981270
O        0.081851      -0.009105       9.815360        1.981316
O        0.082277       7.881321       5.903200        1.939345
O        0.082166      -0.008726       5.903168        1.939329
O        0.098483       1.963537      11.692448        2.237345
O        0.098483       9.853474      11.692096        2.237055
O        0.234696       5.908758       7.859040        1.904680
O        0.234712      13.799010       7.858880        1.904921
O        2.053120       7.863237      11.692864        2.236878
O        2.053152      15.753190      11.692960        2.237374
O        2.053199       3.953663      11.692480        2.236707
O        2.053215      11.843442      11.692832        2.236501
O        2.054603       3.935106       0.004992        1.690381
O        2.054603       3.935106       3.950112        1.911222
O        2.054603       7.879901       0.004992        1.690405
O        2.054603       7.879901       3.950112        1.911180
O        2.054603      11.825106       0.004992        1.690379
O        2.054603      11.825106       3.950112        1.911235
O        2.054603      -0.010099       0.004992        1.690401
O        2.054603      -0.010099       3.950112        1.911123
O        2.054872       3.784470       7.859552        1.904659
O        2.054872      11.674549       7.859424        1.904673
O        2.054777       0.143677       7.859552        1.905008
O        2.054619       8.033614       7.859488        1.905047
O        3.875600       5.908916       7.859744        1.904656
O        3.875694      13.798931       7.860064        1.904566
O        4.007615       1.963584      11.692544        2.236838
O        4.008199       9.853553      11.693152        2.236178
O        4.025289       7.881794       9.814720        1.981135
O        4.026204      -0.008316       9.815264        1.981099
O        4.026930       3.936353       9.815136        1.981202
O        4.026661      11.825721       9.815232        1.981177
O        4.027103       1.962495       0.004992        1.690425
O        4.027103       1.962495       3.950112        1.911408
O        4.027103       3.935106       1.977504        1.897221
O        4.027103       5.907606       0.004992        1.690849
O        4.027103       5.907606       3.950112        1.911867
O        4.027103       7.879901       1.977504        1.897178
O        4.027103       9.852495       0.004992        1.690429
O        4.027103       9.852495       3.950112        1.911399
O        4.027103      11.825106       1.977504        1.897215
O        4.027103      13.797606       0.004992        1.690838
O        4.027103      13.797606       3.950112        1.911765
O        4.027103      -0.010099       1.977504        1.897155
O        4.027135       7.880721       5.902944        1.939468
```

O	4.027119	-0.009358	5.903168	1.939212
O	4.027482	3.936431	5.902976	1.939279
O	4.027561	11.826163	5.903008	1.939272
O	4.043799	5.908616	11.692736	2.236520
O	4.043341	13.798600	11.692608	2.236546
O	4.179680	1.963931	7.858944	1.904679
O	4.179601	9.853979	7.858784	1.904921
O	5.998341	0.008679	11.692512	2.236690
O	5.997931	7.898427	11.689184	2.239115
O	5.998341	3.917574	11.692224	2.236577
O	5.998325	11.808758	11.692256	2.237165
O	5.999698	3.935106	0.004992	1.690601
O	5.999698	3.935106	3.950112	1.911125
O	5.999698	7.879901	0.004992	1.690635
O	5.999698	7.879901	3.950112	1.911257
O	5.999698	11.825106	0.004992	1.690595
O	5.999698	11.825106	3.950112	1.911087
O	5.999698	-0.010099	0.004992	1.690603
O	5.999698	-0.010099	3.950112	1.911245
O	5.999777	4.088409	7.859360	1.905112
O	5.999745	11.978519	7.859552	1.905001
O	5.999556	7.729659	7.859392	1.904744
O	5.999651	15.619707	7.859680	1.904858
O	6.638883	7.740911	18.923840	2.166336
O	7.820694	1.963726	7.859904	1.904507
O	7.820757	9.854168	7.859616	1.904375
O	7.953041	5.908789	11.689024	2.238089
O	7.952583	13.798711	11.692448	2.237584
O	7.971993	1.962495	0.004992	1.690507
O	7.971993	1.962495	3.950112	1.911653
O	7.971993	3.935106	1.977504	1.897234
O	7.971993	5.907606	0.004992	1.690924
O	7.971993	5.907606	3.950112	1.911673
O	7.971993	7.879901	1.977504	1.897310
O	7.971993	9.852495	0.004992	1.690527
O	7.971993	9.852495	3.950112	1.911515
O	7.971993	11.825106	1.977504	1.897238
O	7.971993	13.797606	0.004992	1.690896
O	7.971993	13.797606	3.950112	1.911763
O	7.971993	-0.010099	1.977504	1.897303
O	7.971646	3.935863	9.814368	1.981835
O	7.971709	11.827915	9.814240	1.981826
O	7.972072	3.935721	5.902848	1.939593
O	7.972103	11.825911	5.902784	1.939518
O	7.971393	7.880958	9.813216	1.982420
O	7.971740	-0.008868	9.815232	1.981291
O	7.972024	7.881274	5.902720	1.939398
O	7.972040	-0.008805	5.903264	1.939337
O	7.988373	1.963584	11.691968	2.237761
O	7.988862	9.853616	11.687680	2.238957
O	8.124664	5.908663	7.858720	1.904765
O	8.124601	13.799215	7.858880	1.904909
O	8.994537	7.748895	18.912480	2.166458
O	9.943136	7.863095	11.689152	2.239492
O	9.943025	15.753206	11.692576	2.236645
O	9.943120	3.952732	11.692352	2.236497
O	9.943230	11.844421	11.692320	2.236696
O	9.944603	3.935106	0.004992	1.690435
O	9.944603	3.935106	3.950112	1.911237
O	9.944603	7.879901	0.004992	1.690472
O	9.944603	7.879901	3.950112	1.911121
O	9.944603	11.825106	0.004992	1.690431
O	9.944603	11.825106	3.950112	1.911292
O	9.944603	-0.010099	0.004992	1.690439
O	9.944603	-0.010099	3.950112	1.911068
O	9.944856	3.784360	7.859136	1.904734
O	9.944872	11.674581	7.859040	1.904741
O	9.944903	0.143472	7.859392	1.904885
O	9.945077	8.033424	7.859296	1.904819
O	11.765884	5.908884	7.859584	1.904570
O	11.765757	13.798931	7.859936	1.904565
O	11.897931	1.963616	11.692768	2.236645

139	O	11.897694	9.853458	11.692544	2.235841
140	O	11.917482	7.881826	9.814560	1.981365
141	O	11.916614	-0.008237	9.815168	1.981155
142	O	11.916188	3.936384	9.815040	1.981381
143	O	11.916109	11.825706	9.815040	1.981471
144	O	11.917103	1.962495	0.004992	1.690423
145	O	11.917103	1.962495	3.950112	1.911381
146	O	11.917103	3.935106	1.977504	1.897217
147	O	11.917103	5.907606	0.004992	1.690850
148	O	11.917103	5.907606	3.950112	1.911808
149	O	11.917103	7.879901	1.977504	1.897179
150	O	11.917103	9.852495	0.004992	1.690430
151	O	11.917103	9.852495	3.950112	1.911431
152	O	11.917103	11.825106	1.977504	1.897218
153	O	11.917103	13.797606	0.004992	1.690838
154	O	11.917103	13.797606	3.950112	1.911849
155	O	11.917103	-0.010099	1.977504	1.897159
156	O	11.917640	7.880674	5.903008	1.939413
157	O	11.917529	-0.009263	5.903168	1.939267
158	O	11.917135	3.936447	5.902912	1.939396
159	O	11.917230	11.825974	5.902912	1.939377
160	O	11.933404	5.908742	11.692256	2.235923
161	O	11.933657	13.798569	11.692384	2.236815
162	O	12.069727	1.963884	7.859200	1.904698
163	O	12.069822	9.853931	7.858720	1.904795
164	O	13.888199	0.008632	11.692512	2.236886
165	O	13.888483	7.898537	11.692320	2.237552
166	O	13.888152	3.918111	11.692320	2.236960
167	O	13.888183	11.808221	11.692320	2.236912
168	O	13.889698	3.935106	0.004992	1.690547
169	O	13.889698	3.935106	3.950112	1.911117
170	O	13.889698	7.879901	0.004992	1.690570
171	O	13.889698	7.879901	3.950112	1.911406
172	O	13.889698	11.825106	0.004992	1.690546
173	O	13.889698	11.825106	3.950112	1.911110
174	O	13.889698	-0.010099	0.004992	1.690567
175	O	13.889698	-0.010099	3.950112	1.911311
176	O	13.889777	4.088377	7.859648	1.904846
177	O	13.889808	11.978488	7.859968	1.904813
178	O	13.890061	7.729659	7.859552	1.904819
179	O	13.889872	15.619549	7.859616	1.904741
180	O	15.710647	1.963853	7.859936	1.904476
181	O	15.710742	9.854184	7.860096	1.904455
182	Pd	0.075476	5.912419	13.820832	3.495279
183	Pd	0.075823	13.800273	13.820768	3.495444
184	Pd	0.077338	3.940108	15.763456	2.907225
185	Pd	0.077148	11.828325	15.763648	2.907688
186	Pd	0.077180	7.883988	15.763296	2.910096
187	Pd	0.077969	-0.006107	15.765536	2.906361
188	Pd	0.078285	1.967198	13.820256	3.494848
189	Pd	0.078695	9.855273	13.820544	3.494439
190	Pd	2.049648	1.966851	15.772416	2.868320
191	Pd	2.048638	9.856488	15.776128	2.864068
192	Pd	2.049333	5.912166	15.775616	2.864166
193	Pd	2.050043	13.801440	15.772224	2.868654
194	Pd	2.050327	3.942286	13.820608	3.495058
195	Pd	2.050769	11.828830	13.821088	3.495664
196	Pd	2.051794	7.882741	13.821152	3.495763
197	Pd	2.050311	-0.006991	13.821120	3.494561
198	Pd	4.021880	1.967466	13.820128	3.495829
199	Pd	4.020539	9.855872	13.821984	3.496763
200	Pd	4.024989	7.884998	15.763360	2.871174
201	Pd	4.023695	-0.005050	15.763840	2.908191
202	Pd	4.023379	5.911630	13.822048	3.495942
203	Pd	4.024452	13.800651	13.820608	3.495280
204	Pd	4.023048	3.940219	15.762976	2.906899
205	Pd	4.023648	11.827441	15.763296	2.906895
206	Pd	5.995106	1.968050	15.774496	2.868784
207	Pd	6.000724	9.855131	15.778432	2.846460
208	Pd	5.994380	3.940029	13.820800	3.496181
209	Pd	5.994143	11.826510	13.821024	3.496254
210	Pd	5.999193	5.912908	15.779328	2.841945

Pd	5.994427	13.801267	15.774784	2.868428
Pd	5.993528	7.886134	13.815264	3.494026
Pd	5.994238	-0.004261	13.820800	3.496603
Pd	7.965634	5.905413	13.815072	3.496038
Pd	7.965381	13.802009	13.820224	3.493954
Pd	7.967969	3.941023	15.762880	2.923350
Pd	7.967622	11.828120	15.762368	2.922452
Pd	7.968569	7.885250	15.732352	2.984173
Pd	7.967164	-0.005918	15.763200	2.909685
Pd	7.968237	1.965857	13.819936	3.492470
Pd	7.968679	9.862437	13.813312	3.495930
Pd	9.938638	1.967229	15.774784	2.868842
Pd	9.936335	9.856582	15.777856	2.840404
Pd	9.937281	5.912813	15.778656	2.836720
Pd	9.939159	13.801314	15.774688	2.868609
Pd	9.940469	3.941734	13.820928	3.496473
Pd	9.940359	11.830487	13.820640	3.496883
Pd	9.946055	7.882962	13.813984	3.492541
Pd	9.940264	-0.007054	13.820928	3.496028
Pd	11.909860	1.966472	13.820704	3.495505
Pd	11.910949	9.857419	13.821696	3.497887
Pd	11.910776	7.884793	15.762560	2.879411
Pd	11.911359	-0.005555	15.764416	2.907208
Pd	11.914010	5.910020	13.821952	3.497272
Pd	11.912780	13.801362	13.820544	3.495154
Pd	11.912385	3.939130	15.763456	2.907021
Pd	11.912574	11.828956	15.763328	2.907701
Pd	13.885721	1.966172	15.772224	2.869653
Pd	13.884869	9.856283	15.775296	2.865689
Pd	13.884222	3.938893	13.820448	3.495529
Pd	13.884412	11.826968	13.820672	3.495525
Pd	13.884412	5.912403	15.775520	2.864668
Pd	13.885169	13.802529	15.772928	2.868753
Pd	13.883686	7.885660	13.820032	3.495555
Pd	13.884664	-0.004624	13.820800	3.494856
Sr	2.052063	1.961864	9.831104	1.611275
Sr	2.052489	9.851991	9.831072	1.611426
Sr	2.053467	1.960886	5.891200	1.597706
Sr	2.053672	9.850760	5.891200	1.597733
Sr	2.052757	5.906864	9.831136	1.611319
Sr	2.052631	13.797006	9.831200	1.611211
Sr	2.053988	5.905744	5.891328	1.597699
Sr	2.053688	13.795507	5.891328	1.597620
Sr	2.054603	1.962495	1.977504	1.567300
Sr	2.054603	5.907606	1.977504	1.567428
Sr	2.054603	9.852495	1.977504	1.567298
Sr	2.054603	13.797606	1.977504	1.567423
Sr	5.997015	5.906738	9.830880	1.611811
Sr	5.997252	13.797133	9.831072	1.611544
Sr	5.998593	5.906123	5.891520	1.597866
Sr	5.998609	13.795886	5.891488	1.597801
Sr	5.997631	1.961564	9.830848	1.611323
Sr	5.997536	9.852085	9.830880	1.611724
Sr	5.998909	1.960633	5.891264	1.597634
Sr	5.998767	9.850334	5.891424	1.597635
Sr	5.999698	1.962495	1.977504	1.567383
Sr	5.999698	5.907606	1.977504	1.567532
Sr	5.999698	9.852495	1.977504	1.567373
Sr	5.999698	13.797606	1.977504	1.567532
Sr	9.942473	1.962022	9.830880	1.611524
Sr	9.942931	9.852480	9.830848	1.611749
Sr	9.943278	1.961217	5.891136	1.597751
Sr	9.943341	9.850996	5.891232	1.597738
Sr	9.943120	5.906123	9.830752	1.611524
Sr	9.942836	13.796833	9.830816	1.611560
Sr	9.943799	5.905760	5.891392	1.597679
Sr	9.943578	13.795349	5.891264	1.597655
Sr	9.944603	1.962495	1.977504	1.567297
Sr	9.944603	5.907606	1.977504	1.567417
Sr	9.944603	9.852495	1.977504	1.567293
Sr	9.944603	13.797606	1.977504	1.567426
Sr	13.887410	5.906991	9.830944	1.611540

283	Sr	13.887820	13.797164	9.831136	1.611279
284	Sr	13.888357	5.906249	5.891392	1.597903
285	Sr	13.888499	13.796075	5.891424	1.597826
286	Sr	13.887899	1.961391	9.830944	1.611199
287	Sr	13.887773	9.851706	9.831072	1.611433
288	Sr	13.888830	1.960539	5.891296	1.597629
289	Sr	13.888609	9.850318	5.891296	1.597613
290	Sr	13.889698	1.962495	1.977504	1.567385
291	Sr	13.889698	5.907606	1.977504	1.567541
292	Sr	13.889698	9.852495	1.977504	1.567388
293	Sr	13.889698	13.797606	1.977504	1.567539
294	Ti	0.079263	3.931382	7.854816	2.605868
295	Ti	0.079310	11.821366	7.854848	2.605909
296	Ti	0.080178	7.875861	7.854912	2.606596
297	Ti	0.080083	-0.014186	7.854848	2.606656
298	Ti	0.079831	3.936573	11.749408	2.814447
299	Ti	0.079926	11.826179	11.749504	2.814299
300	Ti	0.080368	7.881384	11.749664	2.814449
301	Ti	0.080162	-0.008663	11.749920	2.814363
302	Ti	0.081993	3.935106	0.004992	2.388293
303	Ti	0.081993	3.935106	3.950112	2.604395
304	Ti	0.081993	7.879901	0.004992	2.388395
305	Ti	0.081993	7.879901	3.950112	2.604436
306	Ti	0.081993	11.825106	0.004992	2.388290
307	Ti	0.081993	11.825106	3.950112	2.604345
308	Ti	0.081993	-0.010099	0.004992	2.388402
309	Ti	0.081993	-0.010099	3.950112	2.604412
310	Ti	4.024358	7.876350	7.854784	2.606156
311	Ti	4.024216	-0.013681	7.854784	2.605983
312	Ti	4.025005	3.930893	7.854816	2.606619
313	Ti	4.025068	11.820830	7.854880	2.606610
314	Ti	4.024784	7.881321	11.749408	2.814243
315	Ti	4.024705	-0.008553	11.749664	2.814263
316	Ti	4.025115	3.936731	11.749536	2.814101
317	Ti	4.025162	11.826037	11.749696	2.813893
318	Ti	4.027103	3.935106	0.004992	2.388248
319	Ti	4.027103	3.935106	3.950112	2.604423
320	Ti	4.027103	7.879901	0.004992	2.388378
321	Ti	4.027103	7.879901	3.950112	2.604594
322	Ti	4.027103	11.825106	0.004992	2.388241
323	Ti	4.027103	11.825106	3.950112	2.604328
324	Ti	4.027103	-0.010099	0.004992	2.388355
325	Ti	4.027103	-0.010099	3.950112	2.604394
326	Ti	7.969168	3.931382	7.854592	2.606025
327	Ti	7.969216	11.821429	7.854592	2.606039
328	Ti	7.970210	7.875861	7.854880	2.606896
329	Ti	7.970005	-0.014123	7.854848	2.606736
330	Ti	7.969863	3.935422	11.748416	2.814478
331	Ti	7.969784	11.827394	11.748320	2.814954
332	Ti	7.970446	7.881274	11.747680	2.814011
333	Ti	7.969973	-0.008490	11.749568	2.814571
334	Ti	7.971993	3.935106	0.004992	2.388394
335	Ti	7.971993	3.935106	3.950112	2.604463
336	Ti	7.971993	7.879901	0.004992	2.388522
337	Ti	7.971993	7.879901	3.950112	2.604548
338	Ti	7.971993	11.825106	0.004992	2.388390
339	Ti	7.971993	11.825106	3.950112	2.604489
340	Ti	7.971993	-0.010099	0.004992	2.388468
341	Ti	7.971993	-0.010099	3.950112	2.604405
342	Ti	11.914310	7.876398	7.854688	2.605947
343	Ti	11.914200	-0.013665	7.854688	2.605904
344	Ti	11.915099	3.930877	7.854720	2.606552
345	Ti	11.915115	11.820877	7.854752	2.606640
346	Ti	11.915904	7.881305	11.749056	2.814523
347	Ti	11.915147	-0.008600	11.749472	2.814345
348	Ti	11.914941	3.936510	11.749376	2.813969
349	Ti	11.914941	11.826274	11.749344	2.814111
350	Ti	11.917103	3.935106	0.004992	2.388242
351	Ti	11.917103	3.935106	3.950112	2.604359
352	Ti	11.917103	7.879901	0.004992	2.388376
353	Ti	11.917103	7.879901	3.950112	2.604601
354	Ti	11.917103	11.825106	0.004992	2.388239

```
Ti      11.917103      11.825106       3.950112         2.604398
Ti      11.917103      -0.010099       0.004992         2.388358
Ti      11.917103      -0.010099       3.950112         2.604481
```
